# Supplementary material for: Enantioselective synthesis of cis-hydrobenzofurans bearing all-carbon quaternary stereocenters and application to total synthesis of (‒)-morphine
Source: Nat Commun. 2019 Jun 7;10:2507. doi: 10.1038/s41467-019-10398-4 (PMC6555830; doi:10.1038/s41467-019-10398-4)
Supplement: Supplementary file 1 — Supplementary Information [file 41467_2019_10398_MOESM1_ESM.pdf]

Supplementary Information

Enantioselective synthesis of *cis*-hydrobenzofurans bearing all-carbon quaternary stereocenters and application to total synthesis of (–)-morphine

Zhang *et al.*

Supplementary Note 1

General Information

All moisture- or oxygen-sensitive reactions were carried out under argon atmosphere in oven-dried flasks. In addition to commercially available extra dry solvents, all solvents were purified and dried by standard techniques, and distilled prior to use. Unless otherwise noted, all reagents were analytically pure and used without further purification. All reactions were monitored by thin-layer chromatography (TLC). Silica gel (200-300 mesh), petroleum ether (bp. 60-90 ℃), ethyl acetate, dichloromethane and methanol were used for product purification by flash column chromatography.

NMR spectra were recorded in $CDCl_3$ solution on Bruker AM-400 MHz or Varian Mercury-600 MHz instruments and calibrated by using residual undeuterated solvent $CHCl_3$ (7.26 ppm) or tetramethylsilane (0.00 ppm) as internal reference for ^1H NMR and the deuterated solvent $CDCl_3$ (77.00 ppm) as internal standard for ^{13}C NMR.

High-resolution mass spectra (**HRMS**) were measured by means of the ESI technique on Fourier transform ion cyclotron resonance mass analyzer.

IR spectra were recorded on a Nicolet FT-170SX spectrometer.

The **MS** data were obtained with Shimadzu GCMS-QP2010SE by means of EI (70 eV) technique.

Chiral high performance liquid chromatography (**HPLC**) analysis data were recorded on a Waters e-2695 instrument equipment with Waters 2998UV/Visible detector.

Optical rotations were detected on RUDOLPH A21202-J APTV/GW.

The X-ray single-crystal determination was performed on an Agilent SuperNova single crystal X-ray diffractometer.

The following abbreviations were used: **PE**: petroleumether; **EtOAc**: ethyl acetate; **DCM**: dichloromethane; **THF**: tetrahydrofuran; **DMF**: *N*,*N*-dimethylformamide; **DDQ**: dichlorodicyanobenzoquinone; **Pd(dppf)Cl$_2$**: [1,1′-bis(diphenylphosphino)ferrocene]dichloropalladium(II); **PTSA**: *p*-toluenesulfonic acid; **LiHMDS**: lithium bis(trimethylsilyl)amide; **DMPU**: *N*,*N*'-Dimethylpropyleneurea; **PPA**: polyphosphoric acids; **Ts**: *p*-toluenesulfonic; **LiDBB**: lithium 4,4′-di-*tert*-butylbiphenylide; **HMPA**: hexamethylphosphoramide.

Supplementary Note 2

Preparation of the Substrates

General Procedure for the Synthesis of Aryl Bromides

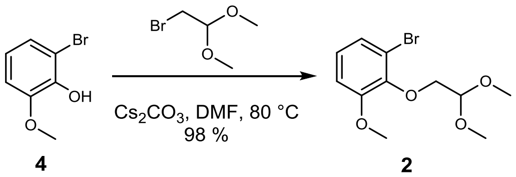

To a stirred solution of 2-bromo-6-methoxyphenol **4** (commercially available) (19.5 g, 96 mmol, 1 eq.) in dry DMF (100 mL) at room temperature was added Cs_2CO_3 (37.4 g, 115 mmol, 1.2 eq.) and bromoacetaldehyde dimethyl acetal (17.0 mL, 144 mmol, 1.5 eq.). The reaction was stirred at 80 ℃ for 8 h. After cooled to room temperature, the reaction mixture was poured into water and extracted with EtOAc. The combined organic layer was washed with brine, dried with Na_2SO_4 and concentrated *in vacuum*. The crude product was purified by column chromatography on silica gel (petroleum ether: ethyl acetate = 30:1) to give product **2** (27.4 g, 98% yield) as a colorless oil.

^{1}H NMR (400 MHz, $CDCl_3$) δ 7.12 (dd, *J* = 8.0, 1.4 Hz, 1H), 6.92 (t, *J* = 8.1 Hz, 1H), 6.84 (dd, *J* = 8.2, 1.2 Hz, 1H), 4.82 (t, *J* = 5.4 Hz, 1H), 4.05 (d, *J* = 5.4 Hz, 2H), 3.85 (s, 3H), 3.46 (s, 6H); **^{13}C NMR** (100 MHz, $CDCl_3$) δ 153.53, 145.29, 124.94, 124.69, 117.56, 111.51, 102.28, 71.61, 55.95, 53.71; **HRMS ESI** Calcd for $C_{11}H_{15}BrO_4Na$ $[M+Na]^+$: 313.0046, Found: 313.0047; **IR (neat)**: 2938, 2834, 1583, 1573, 1478, 1263, 1134, 1081, 1036, 770 cm^{-1}; **EI MS** m/z (%): 75 (100), 88 (8), 180 (17), 201 (11), 203 (9), 261 (5), 290 (18), 292 (18).

Following the above-mentioned method for the synthesis of compound **2**, a series of aryl bromides (**2a**, **2e-2k**) could be analogously obtained. The detailed analytic data were as follows:

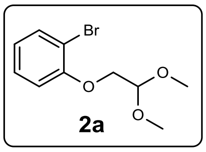

Compound **2a** (95% yield, colorless oil): **^{1}H NMR** (400 MHz, $CDCl_3$) δ 7.52 (dd, *J* = 7.9, 1.6 Hz, 1H), 7.27 – 7.17 (m, 1H), 6.89 (dd, *J* = 8.2, 1.2 Hz, 1H), 6.83 (td, *J* = 7.7, 1.3 Hz, 1H), 4.74 (t, *J* = 5.1 Hz, 1H), 4.04 (d, *J* = 5.2 Hz, 2H), 3.50 (s, 6H); **^{13}C NMR** (100 MHz, $CDCl_3$) δ 154.87, 133.28, 128.36, 122.23, 113.48, 112.21, 102.50, 69.36, 54.80; **HRMS ESI** Calcd for $C_{10}H_{13}BrO_3Na$ $[M+Na]^+$: 282.9940, Found: 282.9938; **IR (neat)**: 2990, 2936, 2833, 1586, 1573, 1480, 1281, 1250, 1137, 1079, 748 cm^{-1}; **EI MS** m/z (%): 75 (100), 119 (5), 197 (3), 199 (3), 260 (2), 262 (3).

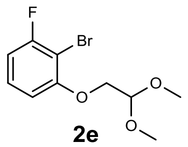

2e

Compound **2e** (90% yield, colorless oil): **^{1}H NMR** (400 MHz, $CDCl_3$) δ 7.21 (td, *J* = 8.4, 6.4 Hz, 1H), 6.77 (td, *J* = 8.2, 1.2 Hz, 1H), 6.69 (d, *J* = 8.4 Hz, 1H), 4.75 (t, *J* = 5.1 Hz, 1H), 4.06 (d, *J* = 5.1 Hz, 2H), 3.51 (s, 6H); **^{13}C NMR** (100 MHz, $CDCl_3$) δ 160.16 (d, *J* = 246.1 Hz), 156.59 (d, *J* = 4.0 Hz), 128.44 (d, *J* = 9.8 Hz), 109.10 (d, *J* = 22.8 Hz), 108.57 (d, *J* = 2.9 Hz), 102.51, 99.96 (d, *J* = 22.6 Hz) , 69.83, 54.96; **^{19}F NMR** (376 MHz, $CDCl_3$) δ -105.12; **HRMS ESI** Calcd for $C_{10}H_{12}BrFO_3Na$ $[M+Na]^+$: 300.9846, Found: 300.9844; **IR (neat)**: 2955, 2930, 1598, 1466, 1247, 1137, 1087, 770 cm^{-1}; **EI MS** m/z (%): 75 (100), 190 (5), 192 (5), 278 (1), 280 (1).

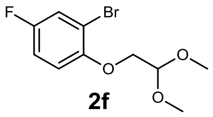

2f

Compound **2f** (91% yield, colorless oil): **^{1}H NMR** (400 MHz, $CDCl_3$) δ 7.34 – 7.24 (m, 1H), 7.01 – 6.92 (m, 1H), 6.87 (dd, *J* = 9.1, 4.8 Hz, 1H), 4.73 (t, *J* = 5.1 Hz, 1H), 4.01 (d, *J* = 5.2 Hz, 2H), 3.50 (s, 6H); **^{13}C NMR** (100 MHz, $CDCl_3$) δ 156.88 (d, *J* = 243.5 Hz), 151.69 (d, *J* = 2.7 Hz), 120.39 (d, *J* = 25.8 Hz), 114.68 (d, *J* = 22.7 Hz), 114.46 (d, *J* = 8.5 Hz), 112.54 (d, *J* = 9.8 Hz), 102.49, 70.20, 54.80; **^{19}F NMR** (376 MHz, $CDCl_3$) δ -121.05; **HRMS ESI** Calcd for $C_{10}H_{12}BrFO_3Na$ $[M+Na]^+$: 300.9846, Found: 300.9840; **IR (neat)**: 2933, 2835, 1592, 1492, 1291, 1263, 1192, 1137, 1079, 863, 777 cm^{-1}; **EI MS** m/z (%): 75 (100), 168 (10), 188 (5), 190 (5), 278 (1), 280 (2).

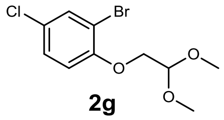

2g

Compound **2g** (93% yield, colorless oil): **^{1}H NMR** (400 MHz, $CDCl_3$) δ 7.53 (d, *J* = 2.5 Hz, 1H), 7.21 (dd, *J* = 8.8, 2.5 Hz, 1H), 6.83 (d, *J* = 8.8 Hz, 1H), 4.73 (t, *J* = 5.1 Hz, 1H), 4.02 (d, *J* = 5.1 Hz, 2H), 3.50 (s, 6H); **^{13}C NMR** (100 MHz, $CDCl_3$) δ 153.87, 132.83, 128.24, 126.51, 114.20, 112.79, 102.46, 69.75, 54.94; **HRMS ESI** Calcd for $C_{10}H_{12}BrClO_3Na$ $[M+Na]^+$: 316.9551, Found: 316.9543; **IR (neat)**: 2954, 2928, 2838, 1477, 1289, 1264, 1137, 1099, 1081, 869, 721 cm^{-1}; **EI MS** m/z (%): 75 (100), 184 (4), 205 (3), 207 (3), 294 (2), 296 (2).

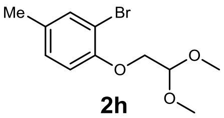

2h

Compound **2h** (92% yield, colorless oil): **^{1}H NMR** (400 MHz, $CDCl_3$) δ 7.32 (d, *J* = 1.6 Hz, 1H), 6.99 (ddd, *J* = 8.3, 2.1, 0.6 Hz, 1H), 6.77 (d, *J* = 8.3 Hz, 1H), 4.71 (t, *J* = 5.2 Hz, 1H), 3.99 (d, *J* = 5.2 Hz, 2H), 3.47 (s, 6H), 2.23 (s, 3H); **^{13}C NMR** (100 MHz, $CDCl_3$) δ 152.68, 133.50, 131.81, 128.66, 113.48, 111.81, 102.38, 69.46, 54.53, 19.91; **HRMS ESI** Calcd for $C_{11}H_{15}BrO_3Na$ $[M+Na]^+$: 297.0097, Found: 297.0091; **IR (neat)**: 2953, 2928, 2834, 1497, 1457, 1288, 1256, 1138, 1099, 1081, 861, 803 cm^{-1}; **EI MS** m/z (%): 75 (100), 149 (4), 164 (4), 262 (4), 274 (4), 276 (4).

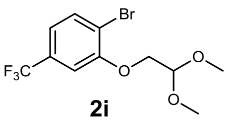

2i

Compound **2i** (83% yield, white solid): **^{1}H NMR** (400 MHz, $CDCl_3$) δ 7.65 (d, *J* = 8.6 Hz, 1H), 7.11 (d, *J* = 6.7 Hz, 2H), 4.76 (t, *J* = 5.1 Hz, 1H), 4.09 (d, *J* = 5.1 Hz, 2H), 3.51 (s, 6H); **^{13}C NMR** (100 MHz, $CDCl_3$) δ 155.29, 133.79, 130.87 (q, *J* = 32.8 Hz), 123.57 (q, *J* = 272.4 Hz), 118.87 (q, *J* = 3.9 Hz) , 116.36, 110.05 (q, *J* = 3.5 Hz), 102.36, 69.64, 54.88; **^{19}F NMR** (376 MHz, $CDCl_3$) δ -62.76; **HRMS ESI** Calcd for $C_{11}H_{12}BrF_3O_3Na$ $[M+Na]^+$: 350.9814, Found: 350.9823; **IR (neat)**: 2956, 2928, 2840, 1490, 1421, 1334, 1251, 1172, 1136, 1032, 905 cm^{-1}; **EI MS** m/z (%): 55 (34), 57 (34), 75 (100), 265 (4), 267 (4); **Mp**: 44−45 ℃.

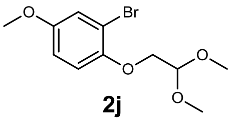

2j

Compound **2j** (88% yield, colorless oil): **^{1}H NMR** (400 MHz, $CDCl_3$) δ 7.10 (d, *J* = 2.9 Hz, 1H), 6.87 (d, *J* = 9.0 Hz, 1H), 6.78 (dd, *J* = 9.0, 3.0 Hz, 1H), 4.72 (t, *J* = 5.2 Hz, 1H), 3.99 (d, *J* = 5.2 Hz, 2H), 3.74 (s, 3H), 3.48 (s, 6H); ^{13}C NMR (100 MHz, $CDCl_3$) δ 154.47, 149.34, 118.71, 115.38, 113.62, 112.97, 102.47, 70.35, 55.72, 54.57; **HRMS ESI** Calcd for $C_{11}H_{15}BrO_4Na$ $[M+Na]^+$: 313.0046, Found: 313.0036; **IR (neat)**: 2957, 2930, 2835, 1605, 1494, 1440, 1276, 1220, 1136, 1080, 1041, 860, 768 cm^{-1}; **EI MS** m/z (%): 75 (100), 201 (6), 203 (6), 290 (5), 292 (5).

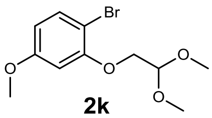

2k

Compound **2k** (88% yield, colorless oil): **^{1}H NMR** (600 MHz, $CDCl_3$) δ 7.38 (dd, *J* = 8.7, 0.6 Hz, 1H), 6.48 (d, *J* = 2.7 Hz, 1H), 6.40 (dd, *J* = 8.7, 2.6 Hz, 1H), 4.73 (t, *J* = 5.1 Hz, 1H), 4.01 (d, *J* = 5.1 Hz, 2H), 3.76 (s, 3H), 3.50 (d, *J* = 0.5 Hz, 6H); **^{13}C NMR** (150 MHz, $CDCl_3$) δ 160.01, 155.55, 133.09, 106.82, 102.90, 102.49, 101.13, 69.44, 55.42, 54.81; **HRMS ESI** Calcd for $C_{11}H_{15}BrO_4Na$ $[M+Na]^+$: 313.0046, Found: 313.0050; **IR (neat)**: 2954, 2934, 2835, 1595, 1581, 1489, 1306, 1207, 1136, 1079, 1023 cm^{-1}; **EI MS** m/z (%):75 (100), 149 (25), 175 (3), 180 (3), 290 (5), 292 (5).

General Procedure for the Synthesis of Acetylenic Amides

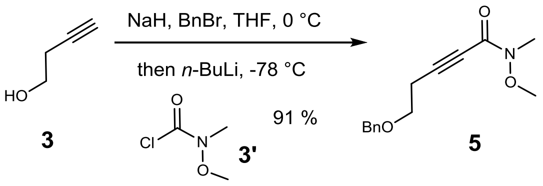

To a stirred solution of 3-butyn-1-ol **3** (1.4 mL, 18.6 mmol, 1.1 eq.) in dry THF (15 mL) at 0 ℃ was slowly added sodium hydride (60%, 1.0 g, 25.4 mmol, 1.5 eq.) and benzyl bromide (2.0 mL, 16.9 mmol, 1 eq.). The solution was warmed to room temperature and stirred for 24 h. Then the reaction was cooled to −78 ℃ and *n*-BuLi (8.1 mL, 20.3 mmol, 2.5 M in hexane, 1.2 eq) was added. After stirred at –78 ℃ for 50 min, a solution of *N*-methoxy-*N*-methylcarbamoyl chloride **3'** (CAS: 30289-28-2, 2.6 mL, 25.4 mmol, 1.5 eq.) in dry THF (5 mL) was added dropwise to the mixture. When the starting material disappeared (monitored by TLC, about 10 min), the reaction was quenched with saturated $NaHCO_3$ solution and extracted with EtOAc. The combined organic extract was washed with saturated $NaHCO_3$ solution and brine, dried with Na_2SO_4 and concentrated *in vacuo*. The crude product was purified by column chromatography on silica gel (petroleum ether: ethyl acetate = 2:1) to give product **5** (3.8 g, 91% yield) as a colorless oil.

The *N*-methoxy-*N*-methylcarbamoyl chloride **3'** was commercially available and also could be prepared according to the reported literature procedure [1-3].

^{1}H NMR (400 MHz, $CDCl_3$) δ 7.40 – 7.27 (m, 5H), 4.55 (s, 2H), 3.73 (s, 3H), 3.66 (t, *J* = 6.8 Hz, 2H), 3.22 (brs, 3H), 2.69 (t, *J* = 6.8 Hz, 2H); **^{13}C NMR** (100 MHz, $CDCl_3$) δ 154.10, 137.56, 128.17, 127.50, 127.41, 89.89, 73.71, 72.76, 67.07, 61.77, 32.07, 20.23; **HRMS ESI** Calcd for $C_{14}H_{17}NO_3Na$ $[M+Na]^+$: 270.1101, Found: 270.1096; **IR (neat)**: 2931, 2865, 2238, 1639, 1455, 1382, 1101 cm^{-1}; **EI MS** m/z (%): 91 (100), 129 (14), 159 (8), 187 (10), 216 (3).

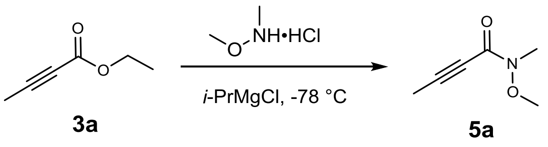

To a stirred solution of ethyl 2-butynoate **3a** (2.0 mL, 17.2 mmol, 1 eq.) and *N*,*O*-dimethylhydroxylamine hydrochloride (5.0 g, 51.6 mmol, 3 eq.) in dry THF (20 mL) at −78 ℃ was added dropwise *i*-PrMgCl (34.4 mL, 68.8 mmol, 2 M in THF, 4 eq.). The mixture was stirred at −78 ℃ for 1 h and then warmed to room temperature. Finally, the reaction was quenched with saturated NH_4Cl solution and extracted with EtOAc. The combined organic extract was washed with brine, dried with Na_2SO_4 and concentrated *in vacuo*. The crude product was purified by column chromatography on silica gel (petroleum ether: ethyl acetate = 2:1) to give product **5a** (1.8 g, 83% yield) as a colorless oil.

^{1}H NMR (400 MHz, $CDCl_3$) δ 3.61 (s, 3H), 3.14 (br, 3H), 1.88 (d, J = 4.8 Hz, 3H); **^{13}C NMR** (100 MHz, $CDCl_3$) δ 154.08, 89.02, 72.01, 61.64, 31.83, 3.62; **HRMS ESI** Calcd for $C_6H_9NO_2Na$ $[M+Na]^+$: 150.0525, Found: 150.0530; **IR (neat)**: 2924, 2243, 1639, 1459, 1378, 1200, 1160, 978, 724 cm^{-1}; **EI MS** m/z (%): 56 (11), 67 (100), 82 (9), 97 (7), 127 (4).

Following the above-mentioned method for the synthesis of **5a**, a series of acetylenic amides (**5b-5c**) could be analogously obtained. The detailed analytic data were as follows:

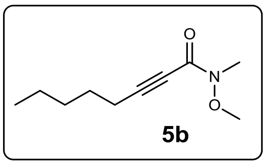

Compound **5b** (87% yield, colorless oil): **^{1}H NMR** (400 MHz, $CDCl_3$) δ 3.61 (s, 3H), 3.02 (br, 3H) , 2.11 (t, J = 7.2 Hz, 2H), 1.36 – 1.29 (m, 2H), 1.18 – 1.04 (m, 4H), 0.64 (t, J = 7.2 Hz, 3H); **^{13}C NMR** (100 MHz, $CDCl_3$) δ 153.82, 92.53, 72.57, 61.19, 31.48, 30.21, 26.72, 21.37, 18.12, 13.15; **HRMS ESI** Calcd for $C_{10}H_{17}NO_2Na$ $[M+Na]^+$: 206.1151, Found: 206.1146; **IR (neat)**: 2957, 2931, 2863, 2236, 1647, 1460, 1378, 1194, 1156, 980, 723 cm^{-1}; **EI MS** m/z (%): 67 (58), 79 (12), 93 (13), 123 (100), 149 (6), 183 (2).

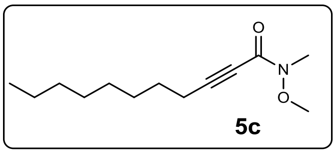

Compound **5c** (88% yield, colorless oil): **^{1}H NMR** (400 MHz, $CDCl_3$) δ 3.71 (s, 3H), 3.18 (br, 3H), 2.32 (t, J = 7.1 Hz, 2H), 1.57 – 1.50 (m, 2H), 1.43 – 1.31 (m, 2H), 1.30 – 1.17 (m, 8H), 0.82 (t, J = 6.8 Hz, 3H); **^{13}C NMR** (150 MHz, $CDCl_3$) δ 154.62, 93.49, 73.16, 61.68, 32.26, 31.64, 28.96, 28.84, 28.69, 27.63, 22.46, 18.83, 13.87; **HRMS ESI** Calcd for $C_{13}H_{23}NO_2Na$ $[M+Na]^+$: 248.1621, Found: 248.1620; **IR (neat)**: 2929, 2857, 2236, 1650, 1461, 1412, 1379, 1197, 1156, 983, 724 cm^{-1}; **EI MS** m/z (%): 55 (48), 67 (40), 81 (75), 95 (60), 75 (100), 226 (12).

General Procedure for the Synthesis of Suzuki Coupling Products

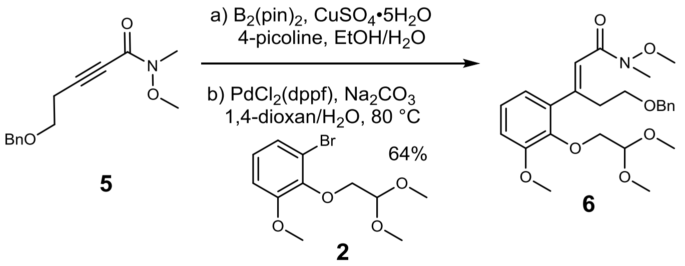

Copper-catalyzed boron addition to acetylenic amide was based on the procedure reported by Santos[4].

Alkynamide **5** (2.0 g, 8.1 mmol, 1 eq.), bis(pinacolato)diboron ($B_2(pin)_2$, 2.46 g, 9.7 mmol, 1.2 eq.) and 4-picoline (118 uL, 1.21 mmol, 0.15 eq.) were suspended in 10% ethanol in H_2O (20 mL) and stirred at room temperature. Then a solution of $CuSO_4$•$5H_2O$ (61 mg, 0.243 mmol, 0.3 eq.) in 0.5 mL H_2O was added in one portion. After stirred at 30 ℃ for 3 h, the green mixture was extracted with EtOAc. The combined organic layer was washed with saturated $CuSO_4$ solution and brine, dried with Na_2SO_4 and concentrated *in vacuum*. The crude product was directly used to the next step.

Next, the crude product was dissolved in a degassed dioxane/H_2O (v:v = 2:1, 25 mL) solution at room temperature. Aryl bromide **2** (2.59 g, 8.9 mmol, 1.1 eq.), Na_2CO_3 (1.72 g, 16.2 mmol, 2 eq.), and Pd(dppf)Cl_2 (178 mg, 0.243 mmol, 0.03 eq.) were added sequentially. The mixture was stirred at 80 ℃ for 2 h. After cooled to room temperature, the reaction mixture was extracted with EtOAc. The combined organic layer was washed with brine, dried with Na_2SO_4 and concentrated *in vacuum*. The crude product was purified by column chromatography on silica gel (petroleum ether: ethyl acetate = 1:1) to give product **6** (2.4 g, 64% yield) as a colorless oil.

^{1}H NMR (400 MHz, $CDCl_3$) δ 7.29 – 7.18 (m, 5H), 7.03 (t, *J* = 7.9 Hz, 1H), 6.89 (dd, *J* = 8.2, 1.4 Hz, 1H), 6.79 (dd, *J* = 7.7, 1.5 Hz, 1H), 6.40 (s, 1H), 4.67 (t, *J* = 5.3 Hz, 1H), 4.38 (s, 2H), 3.94 (d, *J* = 5.4 Hz, 2H), 3.86 (s, 3H), 3.63 (s, 3H), 3.54 (t, *J* = 6.7 Hz, 2H), 3.46 – 3.39 (m, 2H), 3.39 (s, 6H), 3.23 (s, 3H); **^{13}C NMR** (100 MHz, $CDCl_3$) δ 166.98, 152.57, 152.37, 144.72, 138.52, 136.84, 127.91, 127.35, 127.01, 123.96, 121.39, 120.16, 111.72, 102.26, 72.16, 71.82, 68.65, 61.31, 55.58, 53.52, 32.18, 31.85; **HRMS ESI** Calcd for $C_{25}H_{33}NO_7Na$ $[M+Na]^+$: 482.2155, Found: 482.2152; **IR (neat)**: 2936, 2836, 1649, 1576, 1469, 1263, 1133, 1070 cm^{-1}; **EI MS** m/z (%): 91 (100), 175 (14), 201 (24), 245 (34), 277 (7), 399 (9), 428 (2), 460 (1).

Following the above-mentioned method for the synthesis of compound **6**, a series of coupling products (**6a-6c** and **6e-6l**) could be analogously obtained. The detailed analytic data were as follows:

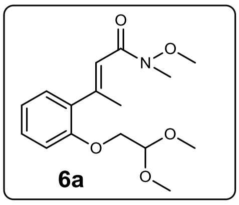

Compound **6a** (58% yield, colorless oil): **^{1}H NMR** (400 MHz, $CDCl_3$) δ 7.31 – 7.23 (m, 1H), 7.18 (dd, J = 7.5, 1.4 Hz, 1H), 6.96 (t, J = 7.5 Hz, 1H), 6.89 (d, J = 8.2 Hz, 1H), 6.33 (s, 1H), 4.69 (t, J = 5.2 Hz, 1H), 4.01 (d, J = 5.2 Hz, 2H), 3.69 (s, 3H), 3.44 (s, 6H), 3.25 (s, 3H), 2.48 (s, 3H); **^{13}C NMR** (100 MHz, $CDCl_3$) δ 167.65, 155.19, 152.72, 133.83, 128.97, 128.88, 120.88, 117.74, 112.03, 102.28, 68.10, 61.26, 54.19, 32.06, 19.43; **HRMS ESI** Calcd for $C_{16}H_{23}NO_5Na$ $[M+Na]^+$: 332.1468, Found: 332.1478; **IR (neat)**: 2936, 2834, 1650, 1489, 1447, 1382, 1367, 1258, 1135, 1077, 980, 756, 739 cm^{-1}; **EI MS** m/z (%):75 (42), 131 (41), 161 (100), 185 (39), 217 (25), 249 (72).

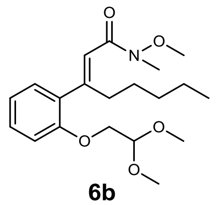

6b

Compound **6b** (62% yield, colorless oil): **^{1}H NMR** (400 MHz, $CDCl_3$) δ 7.31 – 7.24 (m, 1H), 7.13 (dd, J = 7.5, 1.7 Hz, 1H), 6.96 (t, J = 7.4 Hz, 1H), 6.88 (d, J = 8.1 Hz, 1H), 6.23 (s, 1H), 4.67 (t, J = 5.2 Hz, 1H), 4.00 (d, J = 5.2 Hz, 2H), 3.68 (s, 3H), 3.44 (s, 6H), 3.25 (s, 3H), 2.99 (d, J = 7.2 Hz, 2H), 1.34 – 1.21 (m, 6H), 0.81 (t, J = 7.0 Hz, 3H); **^{13}C NMR** (100 MHz, $CDCl_3$) δ 167.56, 157.41, 155.29, 132.56, 129.75, 128.88, 120.86, 118.01, 111.89, 102.48, 68.26, 61.36, 54.41, 32.17, 31.87, 31.84, 28.08, 22.43, 13.97; **HRMS ESI** Calcd for $C_{20}H_{31}NO_5Na$ $[M+Na]^+$: 388.2094, Found: 388.2099; **IR (neat)**: 2956, 2871, 1651, 1489, 1447, 1379, 1253, 1136, 1078, 999, 753 cm^{-1}; **EI MS** m/z (%): 75 (36), 131 (26), 217 (100), 241 (31), 273 (16), 305 (42).

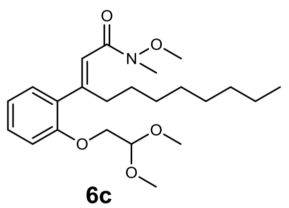

6c

Compound **6c** (64% yield, colorless oil): **^{1}H NMR** (600 MHz, $CDCl_3$) δ 7.26 (t, J = 7.9 Hz, 1H), 7.13 (dd, J = 7.4, 1.4 Hz, 1H), 6.95 (t, J = 7.4 Hz, 1H), 6.88 (d, J = 8.2 Hz, 1H), 6.23 (s, 1H), 4.67 (t, J = 5.1 Hz, 1H), 4.00 (d, J = 5.1 Hz, 2H), 3.68 (s, 3H), 3.43 (s, 6H), 3.25 (s, 3H), 3.00 (s, 2H), 1.33 – 1.18 (m, 12H), 0.84 (t, J = 7.1 Hz, 3H); **^{13}C NMR** (150 MHz, $CDCl_3$) δ 167.54, 156.99, 155.35, 132.63, 129.67, 128.80, 120.84, 118.13, 112.03, 102.51, 68.40, 61.27, 54.30, 32.33, 31.89, 31.71, 29.64, 29.30, 29.13, 28.36, 22.49, 13.91; **HRMS ESI** Calcd for $C_{23}H_{37}NO_5Na$ $[M+Na]^+$: 430.2564, Found: 430.2559; **IR (neat)**: 2926, 2855, 1650, 1489, 1447, 1379, 1265, 1135, 1109, 1079, 994, 739 cm^{-1}; **EI MS** m/z (%): 131 (17), 259 (100), 283 (28), 315 (13), 347 (34).

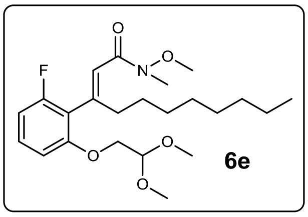

Compound **6e** (73% yield, colorless oil): **^{1}H NMR** (600 MHz, $CDCl_3$) δ 7.17 (dd, *J* = 14.9, 8.3 Hz, 1H), 6.70 (t, *J* = 8.6 Hz, 1H), 6.65 (d, *J* = 8.3 Hz, 1H), 6.20 (s, 1H), 4.61 (t, *J* = 5.1 Hz, 1H), 3.96 (d, *J* = 5.1 Hz, 2H), 3.66 (s, 3H), 3.41 (s, 6H), 3.22 (s, 3H), 2.90 (s, 2H), 1.32 – 1.15 (m, 12H), 0.82 (t, *J* = 7.1 Hz, 3H); **^{13}C NMR** (150 MHz, $CDCl_3$) δ 166.97, 159.91 (d, *J* = 244.4 Hz), 156.71 (d, *J* = 5.6 Hz), 148.95, 128.69 (d, *J* = 10.3 Hz), 120.37 (d, *J* = 16.9 Hz), 108.52, 108.37, 107.44, 102.56, 69.01, 61.42, 54.62, 32.23, 31.74, 29.65, 29.35, 29.16, 28.13, 22.53, 13.95; **^{19}F NMR** (376 MHz, $CDCl_3$) δ -114.28; **HRMS ESI** Calcd for $C_{23}H_{36}FNO_5Na$ $[M+Na]^+$: 448.2470, Found: 448.2468; **IR (neat)**: 2927, 2855, 1654, 1462, 1381, 1273, 1238, 1137, 1099, 996 cm^{-1}; **EI MS** m/z (%):75 (98), 149 (73), 277 (60), 301 (64), 333 (50), 365 (100).

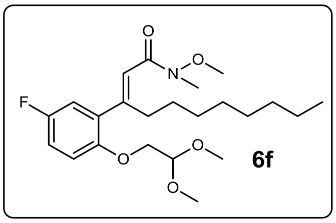

Compound **6f** (67% yield, colorless oil): **^{1}H NMR** (600 MHz, $CDCl_3$) δ 6.95 (td, *J* = 8.5, 3.1 Hz, 1H), 6.86 (dd, *J* = 8.7, 3.1 Hz, 1H), 6.82 (dd, *J* = 9.0, 4.4 Hz, 1H), 6.23 (s, 1H), 4.64 (t, *J* = 5.2 Hz, 1H), 3.95 (d, *J* = 5.2 Hz, 2H), 3.69 (s, 3H), 3.43 (s, 6H), 3.25 (s, 3H), 2.98 (s, 2H), 1.32 – 1.19 (m, 12H), 0.85 (t, *J* = 7.1 Hz, 3H); **^{13}C NMR** (150 MHz, $CDCl_3$) δ 167.26, 157.03 (d, *J* = 240.2 Hz), 155.55, 151.64, 134.05, 118.83, 116.45 (d, *J* = 23.4 Hz), 114.64 (d, *J* = 22.8 Hz), 113.55, 102.52, 69.30, 61.40, 54.39, 32.36, 31.76, 31.75, 29.65, 29.33, 29.16, 28.38, 22.55, 13.96; **^{19}F NMR** (564 MHz, $CDCl_3$) δ -123.28; **HRMS ESI** Calcd for $C_{23}H_{36}FNO_5Na$ $[M+Na]^+$: 448.2470, Found: 448.2475; **IR (neat)**: 2927, 2855, 1652, 1492, 1461, 1379, 1260, 1186, 1136, 1079, 999, 870 cm^{-1}; **EI MS** m/z (%): 149 (26), 277 (100), 301 (29), 333 (23), 365 (50).

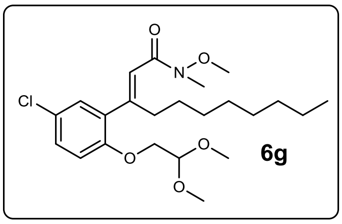

Compound **6g** (61% yield, colorless oil): **^{1}H NMR** (600 MHz, $CDCl_3$) δ 7.20 (dd, *J* = 8.7, 2.6 Hz, 1H), 7.08 (d, *J* = 2.6 Hz, 1H), 6.79 (d, *J* = 8.8 Hz, 1H), 6.19 (s, 1H), 4.63 (t, *J* = 5.1 Hz, 1H), 3.95 (d, *J* = 5.2 Hz, 2H), 3.67 (s, 3H), 3.41 (s, 6H), 3.23 (s, 3H), 2.94 (s, 2H), 1.29 – 1.17 (m, 12H), 0.83 (t, *J* = 7.1 Hz, 3H); **^{13}C NMR** (150 MHz, $CDCl_3$) δ 167.22, 155.59, 154.11, 134.21, 129.44, 128.44, 125.86, 118.79, 113.39, 102.46, 68.84, 61.44, 54.50, 32.29, 31.78, 29.70, 29.35, 29.20, 28.39, 22.58,

14.00; **HRMS ESI** Calcd for $C_{23}H_{36}ClNO_5Na$ $[M+Na]^+$: 464.2174, Found: 464.2175; **IR (neat)**: 2927, 2855, 1653, 1484, 1460, 1379, 1258, 1137, 1079, 996 cm^{-1}; **EI MS** m/z (%): 289 (31), 293 (100), 317 (29), 349 (21), 381 (53).

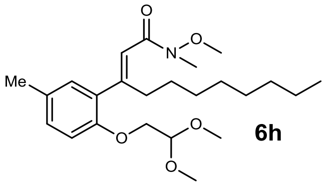

6h

Compound **6h** (57% yield, colorless oil): **^{1}H NMR** (600 MHz, $CDCl_3$) δ 7.05 (dd, *J* = 8.3, 2.1 Hz, 1H), 6.92 (d, *J* = 2.0 Hz, 1H), 6.77 (d, *J* = 8.3 Hz, 1H), 6.20 (s, 1H), 4.64 (t, *J* = 5.2 Hz, 1H), 3.95 (d, *J* = 5.2 Hz, 2H), 3.67 (s, 3H), 3.42 (s, 6H), 3.24 (s, 3H), 2.97 (s, 2H), 2.28 (s, 3H), 1.30 – 1.18 (m, 12H), 0.84 (t, *J* = 7.1 Hz, 3H); **^{13}C NMR** (150 MHz, $CDCl_3$) δ 167.67, 157.37, 153.38, 132.61, 130.38, 130.21, 129.16, 118.00, 112.36, 102.66, 68.79, 61.41, 54.38, 32.41, 32.08, 31.82, 29.78, 29.41, 29.25, 28.49, 22.60, 20.41, 14.01; **HRMS ESI** Calcd for $C_{24}H_{39}NO_5Na$ $[M+Na]^+$: 444.2720, Found: 444.2711; **IR (neat)**: 2926, 2855, 1652, 1496, 1460, 1378, 1235, 1137, 1080, 999 cm^{-1}; **EI MS** m/z (%): 145 (11), 273 (100), 297 (33), 329 (12), 361 (24).

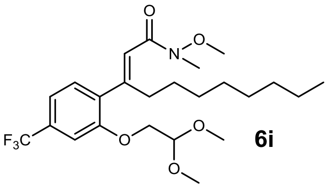

6i

Compound **6i** (51% yield, colorless oil): **^{1}H NMR** (600 MHz, $CDCl_3$) δ 7.22 – 7.16 (m, 2H), 7.06 (s, 1H), 6.19 (s, 1H), 4.64 (t, *J* = 5.1 Hz, 1H), 4.00 (d, *J* = 5.2 Hz, 2H), 3.65 (s, 3H), 3.40 (s, 6H), 3.22 (s, 3H), 2.95 (s, 2H), 1.27 – 1.14 (m, 12H), 0.80 (t, *J* = 7.1 Hz, 3H); **^{13}C NMR** (150 MHz, $CDCl_3$) δ 167.10, 155.55, 155.25, 136.18, 130.99 (q, *J* = 32.4 Hz),130.00, 123.80 (q, *J* = 272.2 Hz), 119.06, 117.68, 108.75, 102.25, 68.54, 61.33, 54.31, 32.14, 31.70, 31.65, 29.59, 29.25, 29.10, 28.26, 22.48, 13.87; **^{19}F NMR** (564 MHz, $CDCl_3$) δ -62.69; **HRMS ESI** Calcd for $C_{24}H_{36}F_3NO_5Na$ $[M+Na]^+$: 498.2438, Found: 498.2435; **IR (neat)**: 2928, 2856, 1652, 1421, 1333, 1117, 1132, 1081, 740 cm^{-1}; **EI MS** m/z (%): 75 (97), 89 (90), 199 (41), 327 (100), 383 (37), 415 (85).

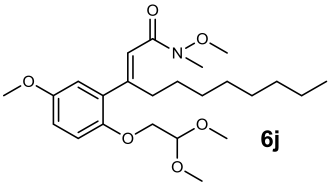

6j

Compound **6j** (88% yield, colorless oil): **^{1}H NMR** (400 MHz, $CDCl_3$) δ 6.77 (dt, *J* = 8.9, 5.9 Hz, 2H), 6.69 (d, *J* = 2.9 Hz, 1H), 6.21 (s, 1H), 4.61 (t, *J* = 5.2 Hz, 1H), 3.91 (d, *J* = 5.2 Hz, 2H), 3.74 (s, 3H), 3.65 (s, 3H), 3.39 (s, 6H), 3.22 (s, 3H), 2.95

(d, J = 7.1 Hz, 2H), 1.31 – 1.15 (m, 12H), 0.82 (t, J = 6.9 Hz, 3H); **^{13}C NMR** (150 MHz, $CDCl_3$) δ 167.50, 156.68, 153.79, 149.61, 133.82, 118.29, 115.85, 113.87, 113.01, 102.58, 69.42, 61.33, 55.58, 54.25, 32.32, 31.91, 31.74, 29.68, 29.32, 29.16, 28.39, 22.52, 13.93; **HRMS ESI** Calcd for $C_{24}H_{39}NO_6Na$ $[M+Na]^+$: 460.2670, Found: 460.2677; **IR (neat)**: 2928, 2855, 1651, 1494, 1462, 1379, 1279, 1221, 1136, 1079, 998, 737 cm^{-1}; **EI MS** m/z (%): 289 (100), 313 (30), 345 (11), 377 (16).

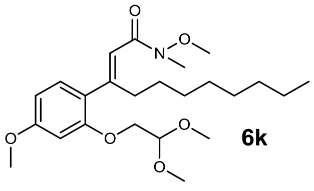

Compound **6k** (59% yield, colorless oil): **^{1}H NMR** (600 MHz, $CDCl_3$) δ 7.05 (d, J = 8.3 Hz, 1H), 6.47 (dd, J = 8.3, 2.3 Hz, 1H), 6.44 (d, J = 2.3 Hz, 1H), 6.20 (s, 1H), 4.65 (t, J = 5.2 Hz, 1H), 3.95 (d, J = 5.2 Hz, 2H), 3.79 (s, 3H), 3.66 (s, 3H), 3.41 (s, 6H), 3.23 (s, 3H), 2.97 (s, 2H), 1.31 – 1.15 (m, 12H), 0.83 (t, J = 7.1 Hz, 3H); **^{13}C NMR** (150 MHz, $CDCl_3$) δ 167.78, 160.52, 156.96, 156.51, 130.29, 125.33, 117.79, 104.99, 102.48, 99.73, 68.47, 61.32, 55.30, 54.38, 32.43, 31.95, 31.78, 29.71, 29.39, 29.20, 28.55, 22.56, 13.98; **HRMS ESI** Calcd for $C_{24}H_{39}NO_6Na$ $[M+Na]^+$: 460.2670, Found: 460.2659; **IR (neat)**: 2927, 2855, 1649, 1608, 1503, 1461, 1301, 1203, 1135, 1079, 737 cm^{-1}; **EI MS** m/z (%): 289 (100), 313 (38), 345 (14), 377 (6).

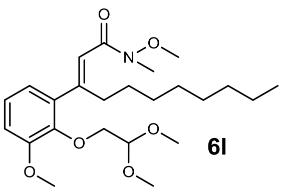

Compound **6l** (69% yield, colorless oil): **^{1}H NMR** (600 MHz, $CDCl_3$) δ 7.00 (t, J = 7.9 Hz, 1H), 6.85 (d, J = 8.2 Hz, 1H), 6.72 (dd, J = 7.7, 1.2 Hz, 1H), 6.25 (s, 1H), 4.66 (t, J = 5.3 Hz, 1H), 3.91 (d, J = 5.3 Hz, 2H), 3.84 (s, 3H), 3.65 (s, 3H), 3.38 (s, 6H), 3.22 (s, 3H), 3.04 – 2.92 (m, 2H), 1.30 – 1.15 (m, 12H), 0.81 (t, J = 7.1 Hz, 3H); **^{13}C NMR** (150 MHz, $CDCl_3$) δ 167.44, 156.59, 152.57, 144.99, 137.52, 123.84, 121.43, 118.32, 111.79, 102.62, 72.20, 61.37, 55.70, 53.67, 32.28, 31.73, 29.72, 29.32, 29.16, 28.51, 24.65, 22.52, 13.94; **HRMS ESI** Calcd for $C_{24}H_{39}NO_6Na$ $[M+Na]^+$: 460.2670, Found: 460.2669; **IR (neat)**: 2927, 2855, 1652, 1469, 1379, 1265, 1135, 1086, 1000, 746 cm^{-1}; **EI MS** m/z (%): 285 (36), 289 (100), 317 (30), 345 (31), 377 (31).

General Procedure for the Synthesis of Aryloxyacetaldehydes

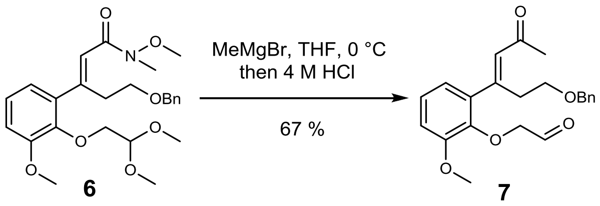

To a stirred solution of **6** (1.0 g, 2.18 mmol, 1 eq.) in dry THF (15 mL) at 0 ℃ was added MeMgBr (2.18 mL, 6.54 mmol, 3 M in Et_2O, 3 eq.). About 0.5 h later, HCl aqueous solution (4 M, 4.5 mL, 18 mmol) was slowly added to the reaction mixture at 0 ℃. Then the reaction was warmed to 40 ℃ and stirred for 4 h. After cooled to room temperature, the mixture was extracted with EtOAc. The combined organic layer was washed with saturated $NaHCO_3$ solution and brine, dried with Na_2SO_4 and concentrated *in vacuum*. The crude product was purified by column chromatography on silica gel (petroleum ether: ethyl acetate = 3:1) to give product **7** (538 mg, 67 % yield) as a colorless oil.

^{1}H NMR (400 MHz, $CDCl_3$) δ 9.85 – 9.79 (m, 1H), 7.32 – 7.22 (m, 3H), 7.20 – 7.15 (m, 2H), 7.06 (t, *J* = 8.0 Hz, 1H), 6.91 (dd, *J* = 8.2, 1.4 Hz, 1H), 6.79 (dd, *J* = 7.7, 1.5 Hz, 1H), 6.31 (d, *J* = 11.9 Hz, 1H), 4.37 (d, *J* = 1.3 Hz, 4H), 3.83 (s, 3H), 3.54 (t, *J* = 6.6 Hz, 2H), 3.35 (t, *J* = 6.6 Hz, 2H), 2.26 (s, 3H); **^{13}C NMR** (100 MHz, $CDCl_3$) δ 200.67, 198.57, 153.67, 151.58, 144.61, 138.35, 136.16, 128.70, 128.16, 127.50, 127.33, 124.40, 121.28, 112.23, 77.53, 72.38, 68.51, 55.59, 32.75, 32.16; **HRMS ESI** Calcd for $C_{22}H_{25}O_5$ $[M+H]^+$: 369.1697, Found: 369.1700; **IR (neat)**: 2938, 2858, 1735, 1684, 1595, 1470, 1359, 1267, 1181, 1095, 1065, 746 cm^{-1}; **EI MS** m/z (%): 91 (100), 161 (11), 201 (8), 247 (6), 279 (11), 309 (19), 368 (1).

Following the above-mentioned method for the synthesis of compound **7**, a series of aryloxyacetaldehydes (**7a-7l**) could be analogously obtained. The detailed analytic data were as follows:

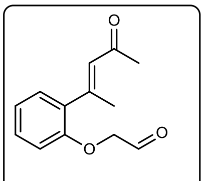

7a

Compound **7a** (60% yield, colorless oil): **^{1}H NMR** (600 MHz, $CDCl_3$) δ 9.85 (t, *J* = 1.0 Hz, 1H), 7.30 (ddd, *J* = 8.2, 7.5, 1.8 Hz, 1H), 7.20 (dd, *J* = 7.5, 1.7 Hz, 1H), 7.02 (td, *J* = 7.5, 1.0 Hz, 1H), 6.76 (d, *J* = 8.3 Hz, 1H), 6.32 (d, *J* = 1.3 Hz, 1H), 4.59 (d, *J* = 1.0 Hz, 2H), 2.50 (d, *J* = 1.3 Hz, 3H), 2.27 (s, 3H); **^{13}C NMR** (150 MHz, $CDCl_3$) δ 198.73, 198.54, 154.55, 153.65, 133.81, 129.56, 129.33, 127.09, 122.04, 112.28, 73.08, 31.98, 20.39; **HRMS ESI** Calcd for $C_{13}H_{14}O_3Na$ $[M+Na]^+$: 241.0835, Found: 241.0841; **IR (neat)**: 2930, 1691, 1599, 1489, 1448, 1248, 1043, 754, 714 cm^{-1}; **EI MS** m/z (%): 115 (14), 131 (20), 175 (5), 159 (100), 218 (1).

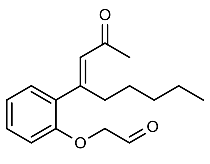

7b

Compound **7b** (58% yield, colorless oil): **^{1}H NMR** (600 MHz, $CDCl_3$) δ 9.83 (t, *J* = 1.1 Hz, 1H), 7.29 (ddd, *J* = 8.2, 7.6, 1.8 Hz, 1H), 7.15 (dd, *J* = 7.5, 1.7 Hz, 1H), 7.02 (td, *J* = 7.5, 0.9 Hz, 1H), 6.75 (d, *J* = 8.2 Hz, 1H), 6.22 (s, 1H), 4.57 (d, *J* = 1.1 Hz, 2H), 3.04 – 3.00 (m, 2H), 2.25 (s, 3H), 1.37 – 1.31 (m, 2H), 1.28 – 1.22 (m, 4H), 0.82 (t, *J* = 7.1 Hz, 3H); **^{13}C NMR** (150 MHz, $CDCl_3$) δ 198.79, 198.47, 158.37, 154.54, 132.60, 129.96, 129.34, 127.13, 121.93, 112.14, 73.08, 32.28, 32.05, 31.86, 28.05, 22.39, 13.92; **HRMS ESI** Calcd for $C_{17}H_{22}O_3Na$ $[M+Na]^+$: 297.1461, Found: 297.1453; **IR (neat)**: 2956, 2929, 2859, 1692, 1600, 1489, 1449, 1248, 1110, 1056, 754 cm^{-1}; **EI MS** m/z (%): 131 (29), 145 (24), 187 (19), 215 (100), 274 (6).

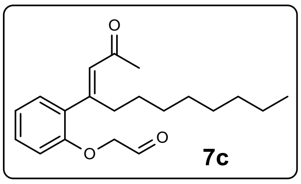

7c

Compound **7c** (67% yield, colorless oil): **^{1}H NMR** (400 MHz, $CDCl_3$) δ 9.83 (s, 1H), 7.29 (dd, *J* = 11.6, 4.1 Hz, 1H), 7.15 (dd, *J* = 7.5, 1.7 Hz, 1H), 7.02 (t, *J* = 7.9 Hz, 1H), 6.75 (d, *J* = 8.2 Hz, 1H), 6.22 (s, 1H), 4.57 (d, *J* = 0.9 Hz, 2H), 3.06 – 2.96 (m, 2H), 2.26 (s, 3H), 1.33 – 1.19 (m, 12H), 0.85 (t, *J* = 6.9 Hz, 3H); **^{13}C NMR** (100 MHz, $CDCl_3$) δ 198.97, 198.58, 158.60, 154.43, 132.47, 129.95, 129.35, 127.04, 121.89, 111.96, 72.94, 32.30, 32.13, 31.79, 29.70, 29.34, 29.20, 28.40, 22.60, 14.05; **HRMS ESI** Calcd for $C_{20}H_{28}O_3Na$ $[M+Na]^+$: 339.1931, Found: 339.1921; **IR (neat)**: 2926, 2855, 1741, 1685, 1597, 1488, 1448, 1246, 752 cm^{-1}; **EI MS** m/z (%): 131 (18), 145 (14), 187 (11), 257 (100) , 316 (3).

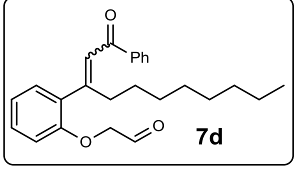

7d

Compound **7d** (51% yield, colorless oil) was obtained as a inseparable mixture of cis- and trans isomers based on the analogous procedure (instead of MeMgBr, PhMgBr was used in the initial Grignard addition). **^{1}H NMR** (600 MHz, $CDCl_3$) δ 9.86 (s, 0.8H), 9.65 (s, 0.2H), 7.99 (d, *J* = 8.0 Hz, 2H), 7.85 (d, *J* = 8.0 Hz, 0.5H), 7.54 (t, *J* = 7.3 Hz, 1H), 7.45 (d, *J* = 7.6 Hz, 1.6H), 7.37 (t, *J* = 7.6 Hz, 0.6H), 7.32 (t, *J* = 7.8 Hz, 1H), 7.20 (s, 0.5H), 7.06 (t, *J* = 7.4 Hz, 1.2H), 6.87 (s, 1.1 *J* = 7.5 Hz, 0.6H), 1.33 – 1.11 (m, 15H), 0.88 (t, *J* = 6.9 Hz, 1H), 0.84 (t, *J* = 7.1 Hz, 3H); **^{13}C NMR** (150 MHz, $CDCl_3$) δ 199.73, 198.92, 191.94, 191.72, 158.77, 154.50, 154.12, 153.82, 139.07, 133.90, 132.56, 130.04, 129.36, 128.83, 128.49, 128.40, 128.25, 124.85, 124.19, 123.32, 121.96, 113.72, 112.04, 73.63, 73.01, 39.57, 32.90, 31.83, 31.79, 29.73, 29.37,

29.35, 29.25, 29.21, 29.08, 28.49, 27.63, 22.61, 14.06; **HRMS ESI** Calcd for $C_{25}H_{30}O_3Na$ $[M+Na]^+$: 401.2087, Found: 401.2080; **IR (neat)**: 2927, 2854, 1739, 1658, 1599, 1484, 1447, 1246, 1042, 751 cm^{-1}; **EI MS** m/z (%): 105 (60), 237 (13), 319 (100), 334 (8), 378 (8).

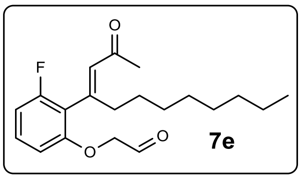

Compound **7e** (69% yield, colorless oil): **^{1}H NMR** (600 MHz, $CDCl_3$) δ 9.80 (s, 1H), 7.22 (td, *J* = 8.4, 6.4 Hz, 1H), 6.80 (t, *J* = 8.5 Hz, 1H), 6.54 (d, *J* = 8.4 Hz, 1H), 6.19 (s, 1H), 4.57 (d, *J* = 0.7 Hz, 2H), 2.95 – 2.91 (m, 2H), 2.26 (s, 3H), 1.36 – 1.20 (m, 12H), 0.85 (t, *J* = 7.1 Hz, 3H); **^{13}C NMR** (150 MHz, $CDCl_3$) δ 198.23, 198.22, 159.94 (d, *J* = 245.7 Hz), 155.69 (d, *J* = 8.0 Hz), 150.49, 129.17 (d, *J* = 10.3 Hz), 128.99, 120.45 (d, *J* = 19.7 Hz), 109.57 (d, *J* = 23.4 Hz), 107.39, 73.20, 32.64, 32.04, 31.81, 29.66, 29.36, 29.19, 28.14, 22.60, 14.02; **^{19}F NMR** (564 MHz, $CDCl_3$) δ -113.28; **HRMS ESI** Calcd for $C_{20}H_{27}FO_3Na$ $[M+Na]^+$: 357.1836, Found: 357.1839; **IR (neat)**: 2927, 2855, 1743, 1688, 1618, 1464, 1274, 1238, 1177, 1095, 782 cm^{-1}; **EI MS** m/z (%): 125 (37), 149 (66), 193 (82), 275 (100), 334 (22).

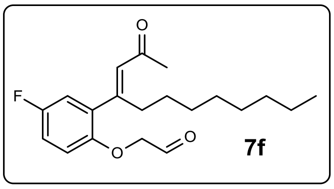

Compound **7f** (71% yield, colorless oil): **^{1}H NMR** (400 MHz, $CDCl_3$) δ 9.81 (s, 1H), 7.00 – 6.94 (m, 1H), 6.89 (dd, *J* = 8.6, 3.1 Hz, 1H), 6.70 (dd, *J* = 9.0, 4.3 Hz, 1H), 6.22 (s, 1H), 4.54 (s, 2H), 3.04 – 2.94 (m, 2H), 2.26 (s, 3H), 1.32 – 1.19 (m, 12H), 0.85 (t, *J* = 6.9 Hz, 3H); **^{13}C NMR** (100 MHz, $CDCl_3$) δ 198.43, 198.41, 157.47 (d, *J* = 241.7 Hz), 156.89 (d, *J* = 1.0 Hz), 150.66 (d, *J* = 2.4 Hz), 133.96 (d, *J* = 7.2 Hz), 127.47, 116.80 (d, *J* = 23.7 Hz), 115.20 (d, *J* = 23.0 Hz), 113.48 (d, *J* = 8.4 Hz), 73.71, 32.13, 32.07, 31.79, 29.66, 29.31, 29.18, 28.37, 22.60, 14.04; **^{19}F NMR** (376 MHz, $CDCl_3$) δ -121.73; **HRMS ESI** Calcd for $C_{20}H_{27}FO_3Na$ $[M+Na]^+$: 357.1836, Found: 357.1828; **IR (neat)**: 2927, 2855, 1741, 1687, 1603, 1492, 1420, 1356, 1261, 1191, 752 cm^{-1}; **EI MS** m/z (%): 149 (18), 193 (15), 205 (15), 275 (100) , 334 (5).

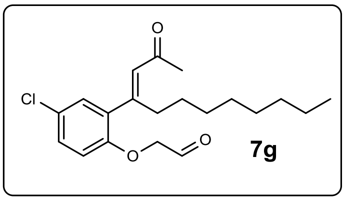

Compound **7g** (70% yield, colorless oil): **^{1}H NMR** (600 MHz, $CDCl_3$) δ 9.81 (s, 1H), 7.24 (dd, *J* = 8.7, 2.2 Hz, 1H), 7.13 (d, *J* = 2.0 Hz, 1H), 6.67 (d, *J* = 8.7 Hz, 1H), 6.20 (s, 1H), 4.57 (s, 2H), 3.02 – 2.94 (m, 2H), 2.26 (s, 3H), 1.31 – 1.19 (m,

12H), 0.85 (t, J = 7.0 Hz, 3H); **^{13}C NMR** (150 MHz, $CDCl_3$) δ 198.34, 197.97, 156.79, 153.15, 134.06, 129.71, 128.87, 127.53, 127.00, 113.32, 73.24, 32.09, 31.80, 29.67, 29.30, 29.19, 28.36, 22.61, 14.04; **HRMS ESI** Calcd for $C_{20}H_{27}ClO_3Na$ $[M+Na]^+$: 373.1541, Found: 373.1541; **IR (neat)**: 2926, 2855, 1741, 1686, 1605, 1483, 1357, 1261, 1236, 1177 cm^{-1}; **EI MS** m/z (%): 209 (19), 291 (100), 293 (37), 350 (7).

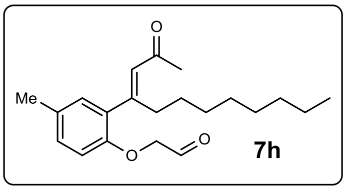

Compound **7h** (72% yield, colorless oil): **^{1}H NMR** (600 MHz, $CDCl_3$) δ 9.82 (s, 1H), 7.08 (d, J = 7.6 Hz, 1H), 6.95 (s, 1H), 6.64 (d, J = 8.3 Hz, 1H), 6.21 (s, 1H), 4.53 (s, 2H), 3.05 – 2.99 (m, 2H), 2.31 (s, 3H), 2.26 (s, 3H), 1.32 – 1.20 (m, 12H), 0.85 (t, J = 6.9 Hz, 3H); **^{13}C NMR** (150 MHz, $CDCl_3$) δ 199.21, 198.51, 158.74, 152.48, 132.37, 131.35, 130.54, 129.62, 126.97, 112.23, 73.32, 32.36, 32.10, 31.82, 29.74, 29.34, 29.22, 28.44, 22.62, 20.44, 14.05; **HRMS ESI** Calcd for $C_{21}H_{30}O_3Na$ $[M+Na]^+$: 353.2087, Found: 353.2091; **IR (neat)**: 2926, 2855, 1741, 1685, 1601, 1495, 1223, 1170 cm^{-1}; **EI MS** m/z (%): 145 (12), 189 (8), 271 (100), 272 (22) , 330 (3).

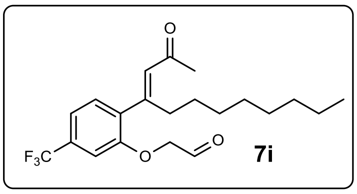

Compound **7i** (52% yield, colorless oil): **^{1}H NMR** (600 MHz, $CDCl_3$) δ 9.80 (s, 1H), 7.22 (td, J = 8.4, 6.4 Hz, 1H), 6.80 (t, J = 8.5 Hz, 1H), 6.54 (d, J = 8.4 Hz, 1H), 6.19 (s, 1H), 4.57 (d, J = 0.7 Hz, 2H), 2.95 – 2.91 (m, 2H), 2.26 (s, 3H), 1.36 – 1.20 (m, 12H), 0.85 (t, J = 7.1 Hz, 3H); **^{13}C NMR** (150 MHz, $CDCl_3$) δ 198.31, 197.05, 156.63, 154.63, 136.12, 131.85, 131.52 (q, J = 33.1 Hz), 127.67, 123.60 (dd, J = 544.3, 272.1 Hz), 118.80, 108.79, 73.02, 32.07, 31.79, 29.66, 29.30, 29.18, 28.32, 22.60, 14.02; **^{19}F NMR** (376 MHz, $CDCl_3$) δ -62.65; **HRMS ESI** Calcd for $C_{21}H_{27}F3O_3Na$ $[M+Na]^+$: 407.1805, Found: 407.1787; **IR (neat)**: 2928, 2856, 1743, 1687, 1605, 1419, 1329, 1171, 1129, 1081 cm^{-1}; **EI MS** m/z (%): 199 (21), 243 (35), 255 (28), 325 (100), 384 (7).

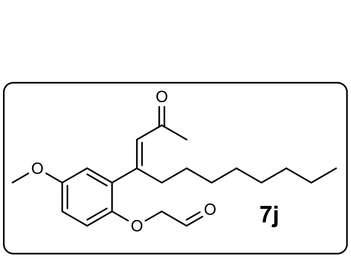

Compound **7j** (67% yield, colorless oil): **^{1}H NMR** (600 MHz, $CDCl_3$) δ 9.82 (s, 1H), 6.80 (dd, J = 8.8, 3.1 Hz, 1H), 6.71 (t, J = 5.7 Hz, 2H), 6.23 (s, 1H), 4.51 (s, 2H), 3.79 (s, 3H), 3.01 – 2.98 (m, 2H), 2.26 (s, 3H), 1.33 – 1.20 (m, 12H), 0.85 (t, J = 7.1 Hz, 3H); **^{13}C NMR** (150 MHz, $CDCl_3$) δ 199.25, 198.51, 158.12, 154.49, 148.70, 133.72, 127.17, 116.06, 113.86, 113.45,

74.07, 55.75, 32.28, 32.10, 31.82, 29.73, 29.34, 29.21, 28.43, 22.62, 14.04; **HRMS ESI** Calcd for $C_{21}H_{30}O_4Na$ $[M+Na]^+$: 369.2036, Found: 369.2020; **IR (neat)**: 2926, 2855, 1740, 1685, 1605, 1493, 1214, 1181, 1040 cm^{-1}; **EI MS** m/z (%): 161 (7), 217 (6), 287 (100), 288 (23), 346 (7).

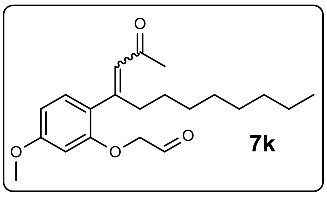

7k

Compound **7k** (48% yield, colorless oil) was obtained as a inseparable mixture of cis- and trans isomers. **^{1}H NMR** (600 MHz, $CDCl_3$) δ 9.81 (s, 0.3H), 9.78 (s, 0.6H), 7.13 (d, *J* = 8.3 Hz, 0.3H), 6.95 (d, *J* = 8.3 Hz, 0.7H), 6.56 (dd, *J* = 8.4, 2.1 Hz, 0.7H), 6.50 (d, *J* = 2.3 Hz, 0.3H), 6.32 (d, *J* = 2.0 Hz, 0.6H), 6.30 (d, *J* = 2.2 Hz, 0.2H), 6.26 (d, *J* = 2.1 Hz, 0.3H), 6.18 (s, 0.7H), 4.55 (s, 1.4H), 4.54 (s, 0.6H), 3.81 (s, 2.1H), 3.78 (s, 0.9H), 2.44 (t, *J* = 7.6 Hz, 1.4H), 2.08 (s, 0.8H), 1.87 (s, 2H), 1.33 – 1.20 (m, 2H), 0.90 – 0.84 (m, 3H); **^{13}C NMR** (150 MHz, $CDCl_3$) δ 199.64, 199.06, 198.82, 198.75, 160.72, 160.03, 154.79, 153.94, 134.65, 134.15, 131.40, 131.09, 130.14, 128.74, 122.14, 105.86, 105.54, 99.98, 99.80, 73.12, 72.82, 55.42, 53.35, 46.58, 39.83, 31.81, 29.95, 29.48, 29.37, 29.20, 29.11, 28.82, 27.59, 22.63, 14.07; **HRMS ESI** Calcd for $C_{21}H_{30}O_4Na$ $[M+Na]^+$: 369.2036, Found: 369.2034; **IR (neat)**: 2926, 2855, 1741, 1678, 1608, 1503, 1460, 1302, 1201, 1042 cm^{-1}; **EI MS** m/z (%): 161 (10), 217 (13), 287 (100), 346 (5).

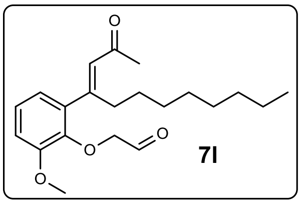

7l

Compound **7l** (74% yield, colorless oil): **^{1}H NMR** (600 MHz, $CDCl_3$) δ 9.90 (s, 1H), 7.06 (t, *J* = 7.9 Hz, 1H), 6.91 (d, *J* = 8.2 Hz, 1H), 6.74 (dd, *J* = 7.7, 1.3 Hz, 1H), 6.20 (s, 1H), 4.40 (d, *J* = 1.2 Hz, 2H), 3.84 (s, 3H), 2.99 – 2.94 (m, 2H), 2.24 (s, 3H), 1.32 – 1.20 (m, 12H), 0.85 (t, *J* = 7.1 Hz, 3H); **^{13}C NMR** (150 MHz, $CDCl_3$) δ 200.75, 198.36, 157.73, 151.70, 144.76, 136.78, 127.15, 124.37, 121.28, 112.22, 77.70, 55.64, 32.70, 32.08, 31.80, 29.72, 29.31, 29.19, 28.42, 22.59, 14.02; **HRMS ESI** Calcd for $C_{21}H_{30}O_4Na$ $[M+Na]^+$: 369.2036, Found: 369.2036; **IR (neat)**: 2926, 2855, 1737, 1686, 1469, 1266, 1181, 1084 cm^{-1}; **EI MS** m/z (%): 161 (8), 217 (11), 287 (100), 346 (3).

Supplementary Note 3

The Conditions Optimization of the Organocatalytic Intramolecular Michael Reaction.

The chiral catalysts **Cat. 5** and **Cat. 6** were prepared according to the literature procedures[5,6]. Others were commercially available.

General procedure: To a stirred solution of the substrate **7** (36.8 mg, 0.1 mmol) in dry dichloromethane (1.0 mL) was added sequentially benzoic acid **A1** (2.4 mg, 20 mol %) and **catalyst** (10 mol %). When the starting material disappeared (monitored by TLC), $Ph_3P=CHCO_2Et$ was added. After stirred at room temperature for 30 min, the reaction mixture was directly purified by flash column chromatography on silica gel to give the product **9**.

Supplementary Table 1. The screening of catalysts for the model reaction

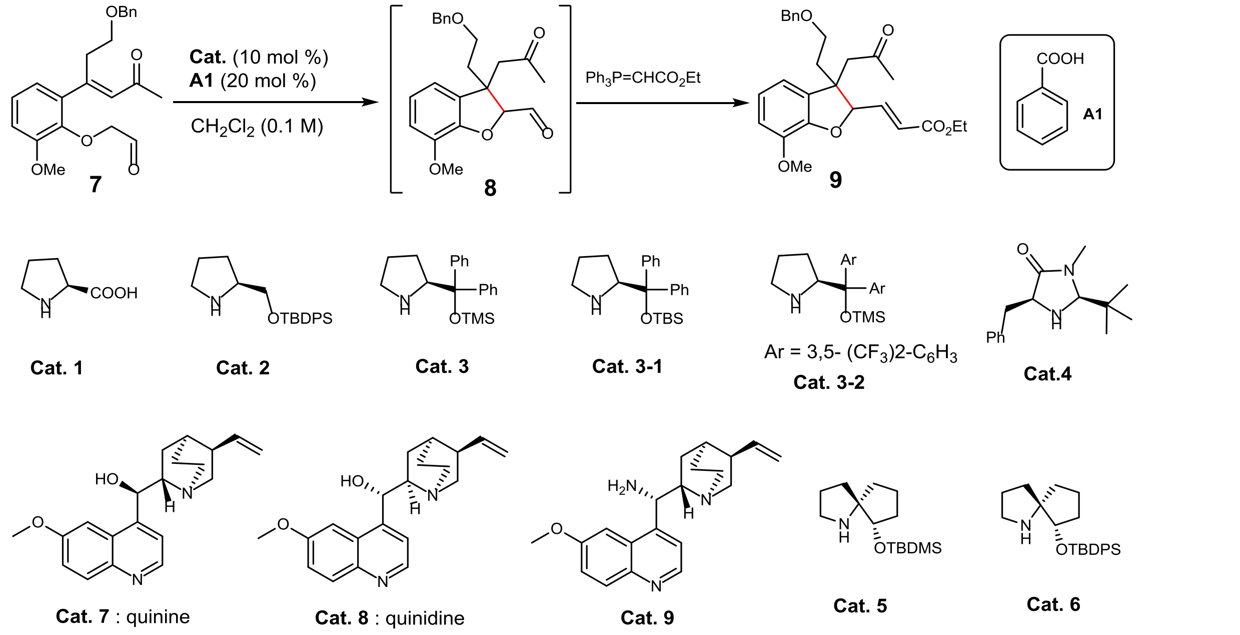

enty	catalyst	*T* [℃]	time	yield[%][a]	dr [%][b]	ee [%][c]
1	1	rt	72 h	trace	-	-
2	2	rt	72 h	60	3.1:1	-34
3	3	rt	24 h	68	1.5:1	36
4	3-1	rt	24 h	37	2.1:1	26
5	3-2	rt	72 h	trace	-	-
6	4	rt	72 h	28	4.1:1	-66
7	7	rt	72 h	trace	-	-
8	8	rt	72 h	trace	-	-
9	9	rt	72 h	58	1.5:1	69
10	5	rt	2 h	71	1.9:1	62
11	6	rt	1 h	73	3.1:1	72
12	6	- 10	10 h	78	5.2:1	78
13	6	- 20	24 h	82	7.1:1	80

[a] Isolated yield. [b] Determined by ^{1}H NMR. [c] Determined by chiral HPLC.

The Screening of Additives for the Model Reaction

The additive **A2-4** and **A4** was prepared according to the reported literature procedures[7,8]. Others were commercially available.

General procedure: To a stirred solution of the substrate **7** (36.8 mg, 0.1 mmol) in dry dichloromethane (1.0 mL) at −20 ℃ was added sequentially **additive** (20 mol %) and **Cat. 6** (3.8 mg, 10 mol %). When the starting material disappeared (monitored by TLC), $Ph_3P{=}CHCO_2Et$ was added. After stirred at −20 ℃ for 30 min, the reaction was warmed to room temperature and stirred for another 30 min. Then the mixture was directly purified by flash column chromatography on silica gel to give the product **9**.

Supplementary Table 2. The screening of additives

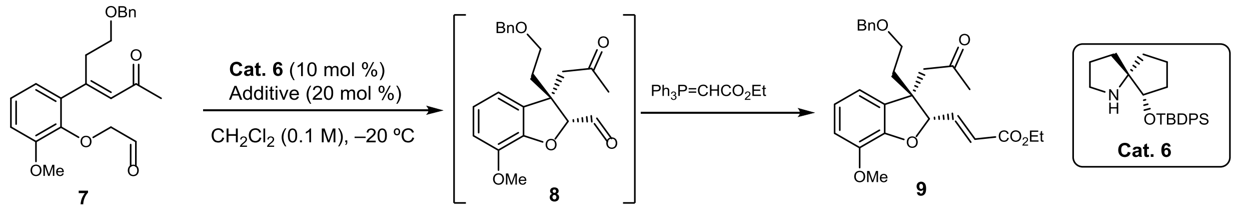

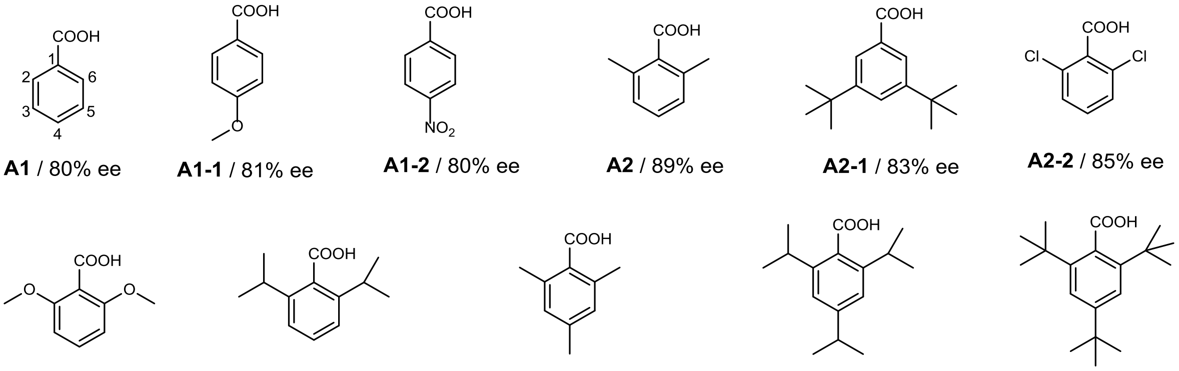

A1 / 80% ee, **A1-1** / 81% ee, **A1-2** / 80% ee, **A2** / 89% ee, **A2-1** / 83% ee, **A2-2** / 85% ee

A2-3 / 86% ee, **A2-4** / 94% ee, **A2-5** / 90% ee, **A3** / 95% ee, **A4** / 88% ee

enty	catalyst	additive	time	yield [%][a]	dr [%][b]	ee [%][c]
1	**6**	**A1**	24 h	82	7.1:1	80
2	**6**	**HOAc**	24 h	71	8.2:1	78
3	**6**	**TFA**	48 h	55	5.2:1	38
4	**6**	**NaOAc**	72 h	< 20	-	71
5	**6**	**Et_3N**	72 h	< 20	-	48
6	**6**	**DBU**	12 h	69	6.6:1	4
7	**6**	**A1-1**	72 h	58	7.8:1	81
8	**6**	**A1-2**	35 h	71	7:1	80
9	**6**	**A2**	20 h	83	6.9:1	89
10	**6**	**A2-1**	40 h	77	6.8:1	83
11	**6**	**A2-2**	20 h	73	9:1	85
12	**6**	**A2-3**	20 h	78	8.3:1	86
13	**6**	**A2-4**	20h	78	7:1	94
14	**6**	**A2-5**	20 h	83	7.2:1	90
**15**	**6**	**A3**	**20 h**	**86**	**7.4:1**	**95**
16	**6**	**A4**	40 h	76	8.7:1	88

[a] Isolated yield. [b] Determined by ^1H NMR. [c] Determined by chiral HPLC.

Synthesis of Additive A2-4

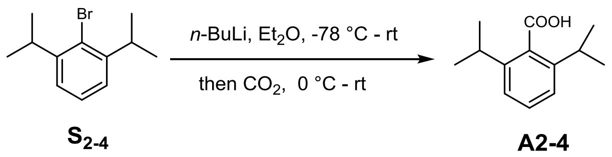

The additive **A2-4** was prepared according to the reported literature procedure[7]. To a stirred solution of **S_{2-5}** (500 mg, 2.08 mmol, 1 eq.) in anhydrous Et_2O (6 mL) at −78 ℃ was added *n*-BuLi (1.3 mL, 3.12 mmol, 2.5 M in hexane, 1.5 eq.). The solution was warmed to room temperature and stirred for 1 h. Then the reaction was cooled to 0 ℃, dry CO_2 (g) was slowly bubbled through the mixture and a large amount of white solid was observed. After stirred at room temperature for 30 min, the reaction mixture was directly purified by flash column chromatography (petroleum ether: ethyl acetate = 5:1) on silica gel to give the product **A2-4** (308 mg, 72%) as a white solid. The analytic data were as follows:

^{1}H NMR (400 MHz, $CDCl_3$) δ 11.40 (s, 1H), 7.38 (t, *J* = 8 Hz, 1H), 7.21 (d, *J* = 7.8 Hz, 2H), 3.14 – 2.98 (m, 2H), 1.30 (d, *J* = 6.8 Hz, 12H); **^{13}C NMR** (100 MHz, $CDCl_3$) δ 176.54, 144.77, 131.66, 129.96, 122.88, 31.60, 24.16.

Synthesis of Additive A4

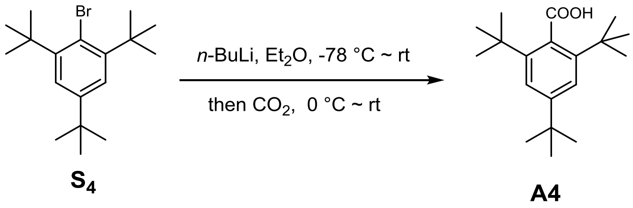

The additive **A4** was prepared according to the reported literature procedure[8].To a stirred solution of **S_4** (800 mg, 2.46 mmol, 1 eq.) in anhydrous Et_2O (10 mL) at −78 ℃ was added *n*-BuLi (1.5 mL, 3.69 mmol, 2.5 M in hexane, 1.5 eq.). The solution was warmed to room temperature and stirred for 1 h. Then the reaction was cooled to 0 ℃, dry CO_2 (g) was slowly bubbled through the mixture and a large amount of white solid was observed. After stirred at room temperature for 30 min, the reaction mixture was directly purified by flash column chromatography (petroleum ether: ethyl acetate = 10:1) on silica gel to give the product **A4** (528 mg, 74%) as a white solid. The analytic data were as follows:

^{1}H NMR (600 MHz, $CDCl_3$) δ 7.44 (s, 2H), 1.48 (s, 18H), 1.32 (s, 9H); **^{13}C NMR** (150 MHz, $CDCl_3$) δ 150.73, 146.43, 127.50, 122.19, 37.05, 35.08, 32.27, 31.29; **EI MS** m/z (%): 57 (22), 257 (24), 275 (100), 290 (16) $[M]^+$.

The Screening of Solvents for the Model Reaction

To a stirred solution of the substrate **7** (36.8 mg, 0.1 mmol) in dry solvent (1.0 mL) was added sequentially 2,4,6-triisopropylbenzoic acid **A3** (5.0 mg, 20 mol %) and **Cat. 6** (3.8 mg, 10 mol %). When the starting material disappeared (monitored by TLC), $Ph_3P=CHCO_2Et$ was added. After stirred for 1 h, the reaction mixture was concentrated *in vacuum* and purified by flash column chromatography on silica gel to give the product **9**.

Supplementary Table 3. The screening of solvents

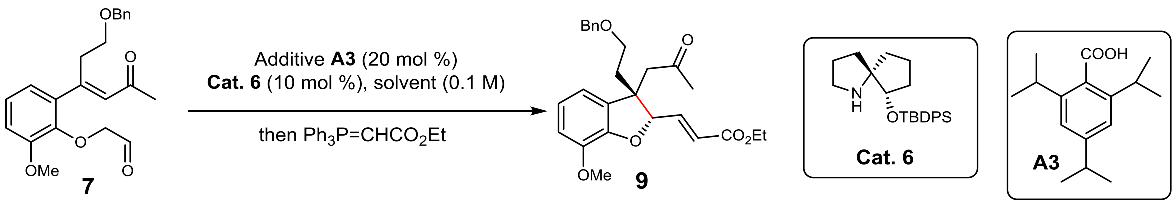

enty	catalyst	solvent	*T*	time	conversion [%][b] (yield [%][a])	dr [%][b]	ee [%][c]
1	**6**	**THF**	-20 ℃	72 h	48(33)	11:1	92
2	**6**	**CH_3CN**	-20 ℃	72 h	76(45)	9.3:1	89
3	**6**	**CH_3OH**	-20 ℃	72 h	trace	-	-
4	**6**	**DMF**	-20 ℃	72 h	47(27)	9.2:1	93
5	**6**	**Toluene**	-20 ℃	30 h	100(77)	8.7:1	94.2
6	**6**	**$CHCl_3$**	-20 ℃	20 h	100(87)	6.8:1	94.2
7	**6**	**DCE**	-20 ℃	20 h	100(86)	8:1	94
8	**6**	**DCM**	-20 ℃	20 h	100(86)	7.1:1	95
**9**	**6**	**DCM**	**-30 ℃**	**48 h**	**100(87)**	**10.2:1**	**96.2**
10	**6**	**DCM**	-40 ℃	96 h	100(85)	11.8:1	96.4

[a] Isolated yield. [b] Determined by ^1H NMR. [c] Determined by chiral HPLC.

The Attempts of the Intramolecular Aldol Condensation

To a stirred solution of the substrate **7** (36.8 mg, 0.1 mmol) in dry dichloromethane (1.0 mL) at −30 ℃ was added sequentially 2,4,6-triisopropylbenzoic acid **A3** (5.0 mg, 20 mol %) and **Cat. 6** (3.8 mg, 10 mol %). After stirred at −30 ℃ for 48 h, the reaction mixture was filtered through a short silica gel column for the removal of **Cat. 6**. The filtrate was concentrated *in vacuo* and directly used to the next step.

To a stirred solution of the substrate **8** (0.05 mmol) in solvent (1.0 mL) was added catalyst (50 mol %) and additive (50 mol %) at room temperature. When the starting material disappeared (monitored by TLC), the reaction mixture was concentrated *in vacuum* and purified by flash column chromatography on silica gel**.**

Supplementary Table 4. Attempts of the intramolecular Aldol condensation

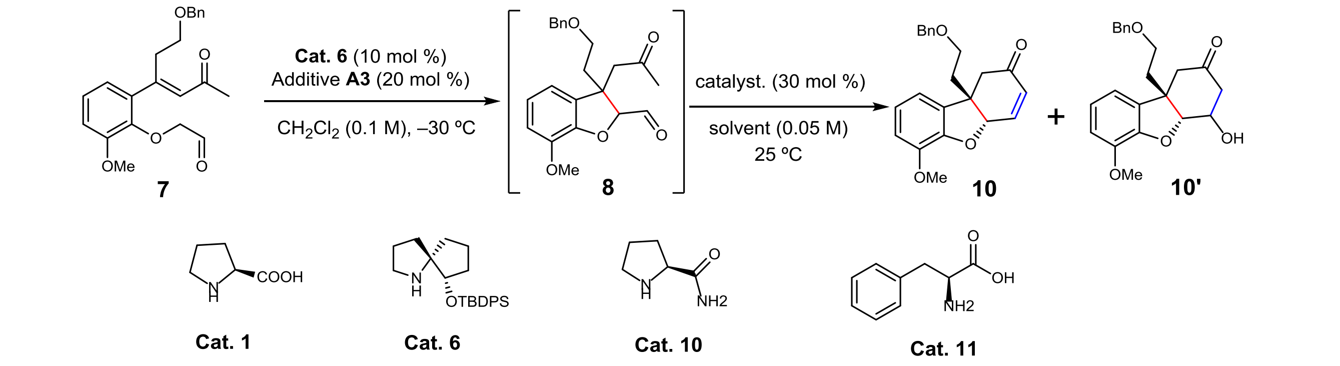

enty	cat.	additive	solvent	time	conversion [%][a]	yield(10) [%][b]	yield(10') [%][b]
1	**1**	-	DMF	72 h	0	NR	NR
2	**1**	-	CH_3CN	72 h	0	NR	NR
3	**1**	$HClO_4$	CH_3CN	72 h	100	decomposition	decomposition
4	**10**	PPTS	CH_3CN	72 h	30	NR	< 10
5	**11**	CSA	CH_3CN	72 h	30	NR	NR

[a] Determined by ^{1}H NMR. [b] Isolated yield. NR: no reaction. PPTS: pyridinium 4-toluenesulfonate. CSA: camphorsulfonic acid.

Compound 10′ : ^{1}H NMR (400 MHz, $CDCl_3$) ^{1}H NMR (400 MHz, $CDCl_3$) δ 7.37 – 7.23 (m, 5H), 6.89 – 6.79 (m, 1H), 6.74 (dd, *J* = 8.1, 0.8 Hz, 1H), 6.61 (dd, *J* = 7.5, 1.0 Hz, 1H), 4.99 (dd, *J* = 3.9, 1.1 Hz, 1H), 4.46 – 4.33 (m, 3H), 3.85 (s, 3H), 3.57 – 3.45 (m, 2H), 3.14 (d, *J* = 2.9 Hz, 1H), 3.03 (d, *J* = 16.2 Hz, 1H), 2.64 (d, *J* = 16.1 Hz, 1H), 2.42 (ddd, *J* = 18.5, 4.5, 1.1 Hz, 1H), 2.28 (dd, *J* = 18.6, 3.1 Hz, 1H), 2.11 (dt, *J* = 13.7, 6.8 Hz, 1H), 1.99 (dt, *J* = 14.4, 5.8 Hz, 1H); ^{13}C NMR (100 MHz, $CDCl_3$) δ 208.77, 146.20, 144.11, 137.51, 132.73, 128.33, 127.69, 127.64, 122.02, 115.10, 111.57, 87.63, 73.15, 68.18, 66.62, 55.73, 47.28, 46.98, 41.38, 41.01; **HRMS ESI** Calcd for $C_{22}H_{22}O_4Na$ $[M+Na]^+$: 391.1521, Found: 391.1515; **IR (neat)**: 3425, 2938, 2909, 1717, 1493, 1460, 1272, 1203, 1059, 738 cm^{-1}; **EI MS** m/z (%): 91 (100), 161 (79), 187 (23), 215 (55) , 259 (23), 350 (8) 368 (17).

General Procedure for the Enantioselective Robinson Annulation

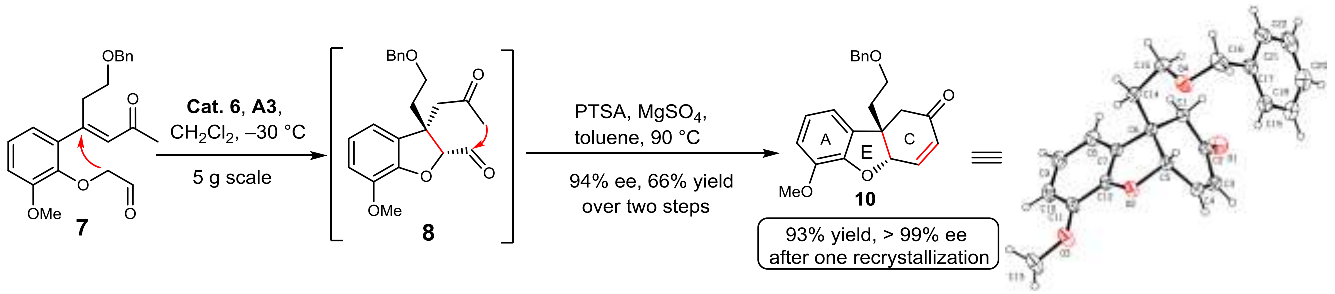

To a stirred solution of the substrate **7** (5.07 g, 13.78 mmol, 1 eq.) in dry DCM (150 mL) at −30 ℃ was added sequentially 2,4,6-triisopropylbenzoic acid **A3** (684 mg, 2.76 mmol, 0.2 eq) and **Cat. 6** (523 mg, 1.38mmol, 0.1 eq.). After stirred at −30 ℃ for 48 h, the reaction mixture was concentrated *in vacuo* (< **30** ℃). Then dry toluene (150 mL), $MgSO_4$ (4.5 g, 30 mg/mL, dried in the muffle furnace at 500 ℃ for 4 h) and PTSA (711 mg, 4.13 mmol, 0.3 eq) were sequentially added at room temperature. The above flask was next placed in an oil-bath (**preheated** at 90 ℃), and the heterogeneous mixture was stirred at 90 ℃ for 2 h. After cooled to room temperature, the dark grey mixture was filtered through a short silica gel column for the removal of $MgSO_4$. The filtrate was concentrated *in vacuo*. The crude product was purified by column chromatography on silica gel (petroleum ether: ethyl acetate = 10:1) to give product **10** (3.18 g, 66% yield, 94% ee) as a colorless oil.

Recrystallization: The product **10** (7.1 g, 20.29 mmol, 94% ee) obtained above was dissolved in ethyl acetate (6 mL). The solution was placed in refrigerator at −20 ℃ for 3 days. Following the filtration and washing with *n*-hexane, the enantioenriched crystal **10** was isolated (6.6 g, 18.86 mmol, 93% yield; > 99% ee).

The single crystal of compound **10** (CCDC **1882059**) was used for the determination of its absolute configuration via X-ray crystallography.

Compound **10**: ^{1}**H NMR** (400 MHz, $CDCl_3$) δ 7.37 – 7.27 (m, 5H), 6.86 (t, *J* = 7.8 Hz, 1H), 6.78 (d, *J* = 7.9 Hz, 1H), 6.74 – 6.65 (m, 2H), 6.06 (d, *J* = 10.3 Hz, 1H), 5.36 (d, *J* = 2.5 Hz, 1H), 4.42 (q, *J* = 11.8 Hz, 2H), 3.86 (s, 3H), 3.57 – 3.39 (m, 2H), 2.98 (d, *J* = 16.4 Hz, 1H), 2.76 (d, *J* = 16.5 Hz, 1H), 2.12 – 2.01 (m, 1H), 2.00 – 1.89 (m, 1H); ^{13}**C NMR** (100 MHz, $CDCl_3$) δ 196.78, 145.03, 145.00, 142.34, 137.88, 132.96, 130.97, 128.34, 127.61, 127.58, 122.17, 114.98, 112.11, 83.16, 73.13, 66.27, 55.85, 48.74, 43.66, 39.90; **HRMS ESI** Calcd for $C_{22}H_{22}O_4Na$ $[M+Na]^+$: 373.1410, Found: 373.1411; **IR (neat)**: 2936, 2861, 1688, 1491, 1455, 1281, 1068, 937, 738 cm^{-1}; **EI MS** m/z (%): 91 (100), 115 (22), 187 (23), 215 (54), 241 (24), 259 (23), 350 (16); $[\alpha]_D^{25}$ = +23 (c = 0.20, $CHCl_3$); **Mp**: 79−80 ℃;

Enantiomeric excess is determined by chiral HPLC (OZ-H, *n*-Hexane/*i*-PrOH = 80/20, flow rate = 1.0 mL/min, 220 nm), t_R (major) = 9.1 min, t_R (minor) = 10.0 min.

The Investigation on the Enantioselectivity of Tricyclic Product 10.

To a stirred solution of the substrate **7** (110 mg, 0.3 mmol) in dry dichloromethane (3.0 mL) was added sequentially additive (20 mol %) and **Cat. 6** (10 mol %). When the starting material disappeared (monitored by TLC), the reaction mixture was divided into two parts. One of them was treated with $Ph_3P{=}CHCO_2Et$, giving the product **9**. Another part was concentrated *in vacuum* and heated to 90 ℃ in dry toluene in the presence of $MgSO_4$ and PTSA, giving the tricyclic product **10**.

Supplementary Table 5. Influence of the diastereoselectivity of initial Michael reaction

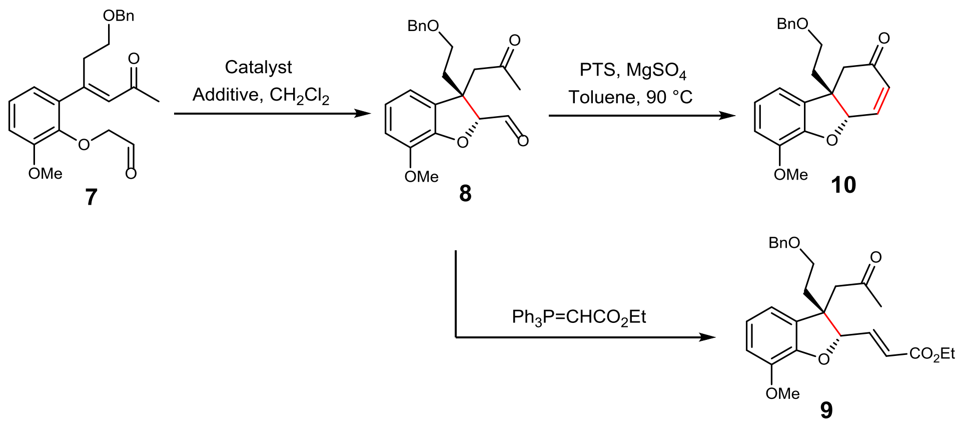

entry	dr [%][a]	ee (9) [%][b]	ee (10) [%][b]	Δ ee (9−10) [%]
1	3.1:1	77.5	71.8	**5.7**
2	3.9:1	95.5	90.2	**5.3**
3	5.2:1	77.7	73.6	**4.1**
4	6.9:1	86.5	84.3	**2.2**
5	10.2:1	94.8	92.8	**2**
6	>20:1	95.5	95.0	**0.5**

[a] Determined by ^{1}H NMR prior to the addition of $Ph_3P{=}CHCO_2Et$. [b] Determined by chiral HPLC.

Compound **8**: **^{1}H NMR** (400 MHz, $CDCl_3$) δ 9.77 (d, *J* = 0.5 Hz, 1H), 7.34 – 7.27 (m, 5H), 6.86 – 6.82 (m, 1H), 6.78 (dd, *J* = 8.1, 1.2 Hz, 1H), 6.58 (dd, *J* = 7.3, 1.3 Hz, 1H), 5.05 (s, 1H), 4.43 (d, *J* = 4.9 Hz, 2H), 3.91 (s, 3H), 3.88 – 3.83 (m, 2H), 3.54 – 3.47 (m, 2H), 3.26 (d, *J* = 18.2 Hz, 1H), 2.87 (d, *J* = 18.2 Hz, 1H), 2.14 (td, *J* = 6.0, 1.9 Hz, 2H), 1.94 (s, 3H); **^{13}C NMR** (100 MHz, $CDCl_3$) δ 207.03, 200.82, 146.68, 144.48, 137.86, 132.18, 128.34, 127.70, 127.64, 121.84, 114.69, 111.74, 91.55, 73.16, 66.43, 55.87, 49.80, 47.20, 39.99, 30.92; **HRMS ESI** Calcd for $C_{22}H_{24}O_5Na$ $[M+Na]^+$: 391.1521, Found: 391.1528; **IR (neat)**: 2937, 2861, 1723, 1622, 1493, 1457, 1365, 1274, 1204, 1100, 1066, 911, 734 cm^{-1}; **EI MS** m/z (%): 91 (100), 161 (9), 189 (7), 204 (8), 231 (2), 265 (2), 311 (2), 368 (1).

The Proposed Reaction Mechanism

In order to obtain the chiral product **9** with high enantioselectivity, we made great efforts toward extensive screening of the additives.

As is shown in **Figure 1** (also please see the **Table 2**), firstly, the acidity of the derivatives of benzoic acid had a negligible influence on stereochemical outcome (**A1** vs **A1-1** vs **A1-2**). Subsequently, the effect on the substituents at various positions on aryl ring of benzoic acid was investigated and the results demonstrated that the substituents at C2 and C6 positions had a more significant impact on improving enantioselectivity than that of other positions (**A2** vs **A2-1** vs **A2-4**). Therefore, a variety of substituent groups with diverse steric and electronic properties at the C2 and C6 positions were further investigated (**A2**, **A2-2**, **A2-3**, **A2-4**). In comparison with the electronic effect of substituents (**A2** vs **A2-2** vs **A2-3**), the steric hindrance of benzoic acid had an obvious effect in obtaining high enantioselectivities (**A2** vs **A2-4**). Among of them, the more sterically bulky additive **A2-4** afforded the better enantioselectivity. To further optimize the structure of additives, different substituents at C2, C4 and C6 positions of benzoic acid were also evaluated (**A2-5**, **A3**, **A4**), and finally, we found the additive **A3** gave the best result (95% ee). Unfortunately, the more hindered 2,4,6-tritertbutylbenzoic acid (**A4**) had a negative effect on enantioselectivity, maybe owing to its mismatching with the rigid catalyst **Cat. 6** in this Michael addition for the construction of quaternary carbon center.

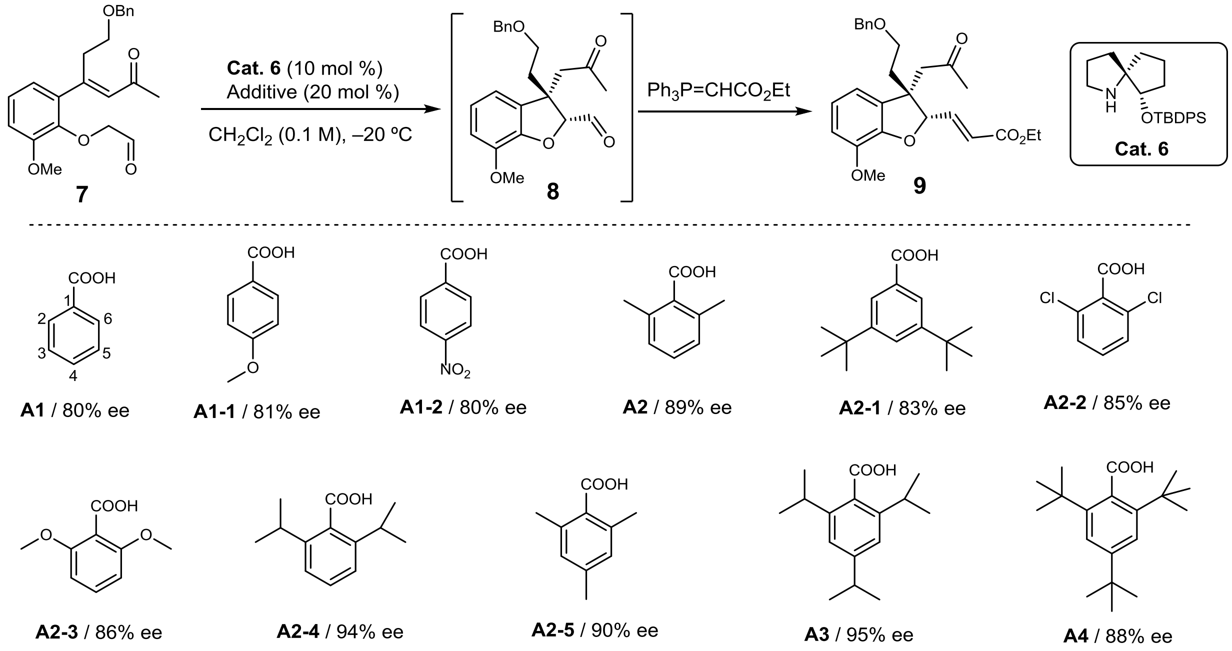

Supplementary Figure 1. The screening of the additives

Based on the above experimental results and the well-established reactions model by aminocatalysis[9,10], a possible stereocontrol model of this Michael addition is proposed (**Figure 2**). Firstly, dehydration between **Cat. 6** and the aldehyde of substrate **S7** in the presence of the additive **A3** generates the iminium ion intermediate **B** (with the effect of an achiral counteranion[11]), which can easily convert into the more stable enamine intermediate **C**. As is shown in the transition-state model **C**, both the enamine formation and a hydrogen bonding interaction

between the additive **A3** and the enone moiety of **S7** probably work together to impact the stereoselectivity of the Michael addition. In this process, due to the steric hindrance of the TBDPS group in **Cat. 6**, the intramolecular nucleophilic attack from the Si face of the enone moiety is more favorable, thus leading to the formation of the quaternary center with a S configuration (if R^2 is C_2H_4OBn). However, presumably due to the steric hindrance of the R^L group and R^2 group, the process of the other nucleophilic attack (reaction models **E** and **F**) was unfavorable. Moreover, the additive **A3** is characterized with two bulky isopropyl groups, which may effectively match with the rigid catalyst **Cat. 6** to implement a chiral reaction environment. Finally, hydrolysis of iminium ion intermediate **D** affords the product **P8** and the catalyst **Cat. 6** is released for the next catalytic cycle.

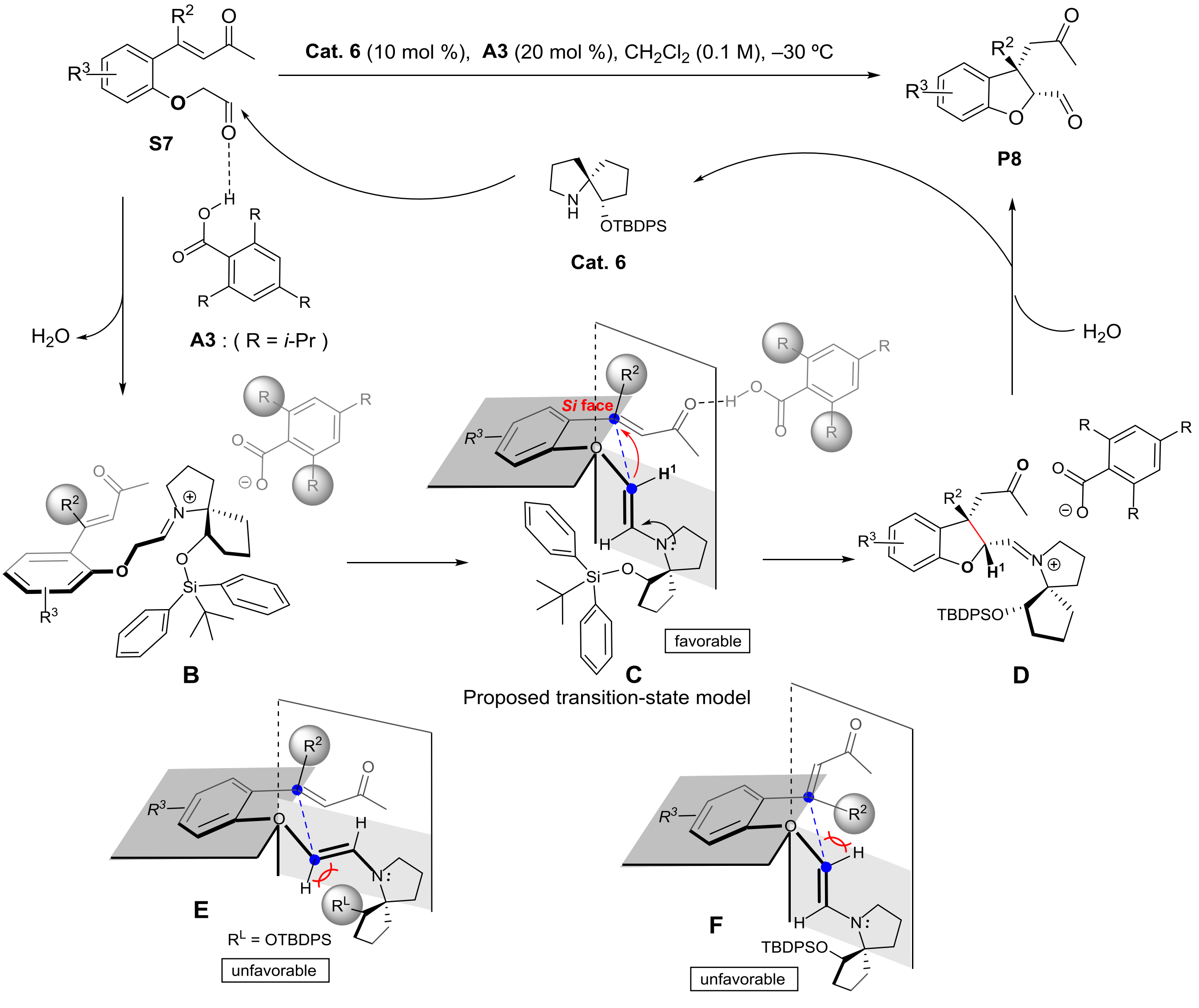

Supplementary Figure 2. The proposed reaction mechanism

Supplementary Note 4

Generality of Asymmetric Michael Addition/Wittig Reaction

Supplementary Table 6. The substrate scope of one-pot Michael addition/Wittig reaction

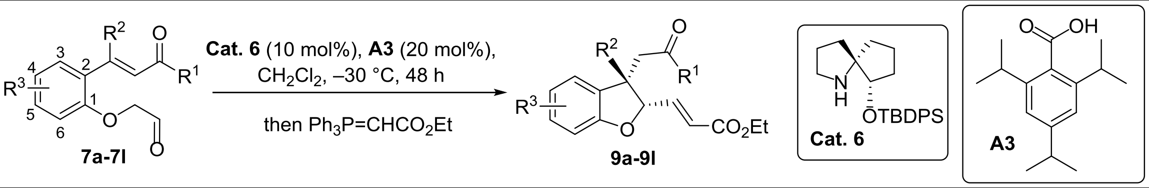

Entry	Substrate	R[1]	R[2]	R[3]	Product	Yield [%][a]	d.r.[b]	ee [%][c]
1	**7a**	Me	Me	H	**9a**	74	8.4:1	89
2	**7b**	Me	C_5H_{11}	H	**9b**	78	9.4:1	93
3	**7c**	Me	C_8H_{17}	H	**9c**	80	11.2:1	95
4	**7d**	Ph	C_8H_{17}	H	**9d**	71	9.3:1	93
5	**7e**	Me	C_8H_{17}	3-F	**9e**	78	>20:1	95
6	**7f**	Me	C_8H_{17}	4-F	**9f**	78	12.2:1	95
7	**7g**	Me	C_8H_{17}	4-Cl	**9g**	74	11.3:1	96
8	**7h**	Me	C_8H_{17}	4-Me	**9h**	87	17.8:1	95
9	**7i**	Me	C_8H_{17}	5-CF_3	**9i**	78	13.9:1	96
10	**7j**	Me	C_8H_{17}	4-MeO	**9j**	82	13.6:1	89
11	**7k**	Me	C_8H_{17}	5-MeO	**9k**	65	19:1	88
12	**7l**	Me	C_8H_{17}	6-MeO	**9l**	82	13.3:1	96

[a] Isolated yield. [b] The dr value was determined by ^1H NMR prior to the addition of $Ph_3P{=}CHCO_2Et$. [c] The ee value was determined by chiral HPLC.

Disscusion of the substrate scope.

Having established the optimal reaction conditions, we next explored the substrate scope of the intramolecular Michael addition (Table 6). The substituents on the double bond of the enone moiety were first investigated (Table 6, entries 1-3). The results showed that the chain length of the R^2 group had an evident influence on stereochemical outcome, and a more sterically hindered group led to a higher yield and better enantio- and diastereoselectivities. Subsequently, when the methyl group of the enone moiety was replaced by a phenyl group, the reaction proceeded well without any decrease in the yield and enantioselectivity (Table 6, entry 4). Lastly, the substitution pattern of arene was investigated (Table 6, entries 5-12), and a variety of aldehydes **7e-7l** reacted smoothly, generating the desired hydrobenzofuran products **9e-9l**, respectively. Both electron-deficient and electron-rich substrates successfully participated in the Michael addition to give good yields as well as excellent enantio- and diastereoselectivities. These reaction results demonstrated that the SPD catalyst had exhibited unique property in this unprecedented Michael addition for the construction of the challenging benzylic quaternary carbon stereocenter. More importantly, the current asymmetric transformation provides a potential

platform for the preparation of a series of structurally related natural products or drugs bearing such hydrobenzofuran skeleton.

General procedure: To a stirred solution of the substrates **7a-7l** (0.1 mmol) in dry DCM (1.0 mL) at −30 ℃ was added sequentially 2,4,6-triisopropylbenzoic acid **A3** (5.0 mg, 20 mol %) and **Cat. 6** (3.8 mg, 10 mol %). When the starting materials disappeared (monitored by TLC, about 48 h), $Ph_3P=CHCO_2Et$ was added. After stirring at −30 ℃ for 30 min, the reaction was warmed to room temperature and stirred for another 30 min. Then the reaction mixture was directly purified by flash column chromatography on silica gel to give the products **9a-9l**. The detailed analytic data were as follows:

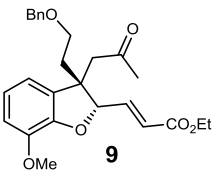

9

Compound **9** (87% yield, 96% ee, 10.2:1 dr, colorless oil): $[\alpha]_D^{25}$= −43 (c = 0.10, $CHCl_3$); **^{1}H NMR** (400 MHz, $CDCl_3$) δ 7.32 (dt, *J* = 18.8, 7.7 Hz, 5H), 6.90 – 6.75 (m, 3H), 6.64 (dd, *J* = 7.1, 1.4 Hz, 1H), 6.09 (dd, *J* = 15.7, 1.5 Hz, 1H), 5.39 (dd, *J* = 5.7, 1.5 Hz, 1H), 4.40 (q, *J* = 11.7 Hz, 2H), 4.15 (q, *J* = 7.1 Hz, 2H), 3.88 (s, 3H), 3.48 – 3.40 (m, 2H), 3.26 (d, *J* = 17.8 Hz, 1H), 2.49 (d, *J* = 17.9 Hz, 1H), 2.44 – 2.35 (m, 1H), 2.04 (dt, *J* = 14.6, 4.6 Hz, 1H), 1.93 (s, 3H), 1.25 (t, *J* = 7.1 Hz, 3H); **^{13}C NMR** (100 MHz, $CDCl_3$) δ 206.68, 165.96, 146.01, 144.93, 142.15, 138.04, 133.02, 128.33, 127.84, 127.64, 123.53, 121.44, 116.44, 112.05, 90.70, 73.11, 66.71, 60.41, 55.97, 50.68, 46.59, 35.89, 30.90, 14.16; **HRMS ESI** Calcd for $C_{26}H_{30}O_6Na$ $[M+Na]^+$: 461.1935, Found: 461.1931; **IR (neat)**: 2957, 2937, 2865, 1718, 1621, 1491, 1457, 1367, 1280, 1269, 1177, 1099, 1066, 737 cm^{-1}; **EI MS** m/z (%): 91 (100), 257 (25), 274 (23), 380 (47), 438 (3). Enantiomeric excess is determined by chiral HPLC (AY-H, *n*-Hexane/*i*-PrOH = 90/10, flow rate = 1.0 mL/min, 220 nm) , t_R (minor) = 16.7 min, t_R (major) = 31.3 min.

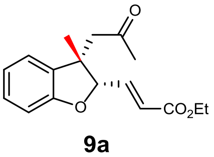

9a

Compound **9a** (74% yield, 89% ee, 8.4:1 dr, colorless oil): $[\alpha]_D^{25}$ = −4 (c = 0.25, $CHCl_3$); **^{1}H NMR** (600 MHz, $CDCl_3$) δ 7.16 (td, *J* = 7.8, 1.3 Hz, 1H), 7.13 (d, *J* = 7.6 Hz, 1H), 6.94 – 6.83 (m, 3H), 6.16 (dd, *J* = 15.7, 1.6 Hz, 1H), 5.02 (dd, *J* = 5.4, 1.5 Hz, 1H), 4.19 (q, *J* = 7.1 Hz, 2H), 2.86 (d, *J* = 17.2 Hz, 1H), 2.51 (d, *J* = 17.2 Hz, 1H), 2.01 (s, 3H), 1.53 (s, 3H), 1.28 (t, *J* = 7.1 Hz, 3H); **^{13}C NMR** (150 MHz, $CDCl_3$) δ 206.31, 165.85, 157.68, 141.87, 134.10, 128.74, 123.65, 123.53, 121.20, 110.22, 90.87, 60.56, 48.58, 47.50, 31.41, 24.48, 14.19; **HRMS ESI** Calcd for $C_{17}H_{20}O_4Na$ $[M+Na]^+$: 311.1254, Found: 311.1259; **IR (neat)**: 2920, 1720, 1476, 1367, 1302, 1272, 1177, 1035, 754 cm^{-1}; **EI MS** m/z (%): 145 (100), 160 (33), 185

(38), 230 (21), 288 (17); Enantiomeric excess is determined by chiral HPLC (IF-3, *n*-Hexane/*i*-PrOH = 95/5, flow rate = 1.0 mL/min, 220 nm) , t_R (minor) = 8.0 min, t_R (major) = 9.1 min.

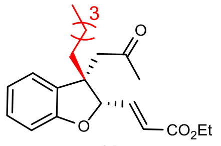

9b

Compound **9b** (78% yield, 93% ee, 9.4:1 dr, colorless oil): $[\alpha]_D^{28}$ = −52.5 (c = 0.40, $CHCl_3$); **^{1}H NMR** (600 MHz, $CDCl_3$) δ 7.16 (td, *J* = 7.9, 1.3 Hz, 1H), 7.05 (dd, *J* = 7.4, 0.9 Hz, 1H), 6.91 – 6.80 (m, 3H), 6.09 (dd, *J* = 15.7, 1.6 Hz, 1H), 5.20 (dd, *J* = 5.4, 1.6 Hz, 1H), 4.17 (q, *J* = 7.1 Hz, 2H), 2.99 (d, *J* = 17.7 Hz, 1H), 2.55 (d, *J* = 17.8 Hz, 1H), 2.05 (s, 3H), 1.95 – 1.83 (m, 2H), 1.29 – 1.17 (m, 8H), 1.05 (ddd, *J* = 17.3, 12.1, 5.4 Hz, 1H), 0.82 (t, *J* = 7.1 Hz, 3H); **^{13}C NMR** (150 MHz, $CDCl_3$) δ 206.42, 165.96, 158.06, 142.83, 132.29, 128.75, 124.06, 123.34, 120.84, 110.09, 89.19, 60.50, 51.09, 46.94, 37.44, 32.07, 31.26, 23.74, 22.42, 14.19, 13.91; **HRMS ESI** Calcd for $C_{21}H_{28}O_4Na$ $[M+Na]^+$: 367.1880, Found: 367.1881; **IR (neat)**: 2956, 2930, 2860, 1718, 1596, 1477, 1460, 1367, 1301, 1269, 1177, 1036, 752 cm^{-1}; **EI MS** m/z (%): 201 (25), 227 (100), 286 (18), 344 (16); Enantiomeric excess is determined by chiral HPLC (IF-3, *n*-Hexane/*i*-PrOH = 95/5, flow rate = 1.0 mL/min, 220 nm) , t_R (minor) = 6.4 min, t_R (major) = 7.1 min.

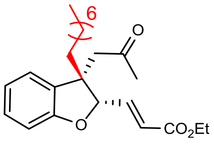

9c

Compound **9c** (80% yield, 95% ee, 11.2:1 dr, colorless oil): $[\alpha]_D^{27}$ = −60 (c = 0.20, $CHCl_3$); **^{1}H NMR** (600 MHz, $CDCl_3$) δ 7.17 (t, *J* = 7.6 Hz, 1H), 7.05 (d, *J* = 7.3 Hz, 1H), 6.92 – 6.77 (m, 3H), 6.09 (d, *J* = 15.6 Hz, 1H), 5.20 (d, *J* = 5.1 Hz, 1H), 4.17 (q, *J* = 7.0 Hz, 2H), 2.99 (d, *J* = 17.8 Hz, 1H), 2.55 (d, *J* = 17.8 Hz, 1H), 2.05 (s, 3H), 1.93 – 1.84 (m, 2H), 1.29 – 1.18 (m, 14H), 1.08 – 0.97 (m, 1H), 0.85 (t, *J* = 7.1 Hz, 3H); **^{13}C NMR** (150 MHz, $CDCl_3$) δ 206.37, 165.96, 158.06, 142.83, 132.30, 128.76, 124.07, 123.36, 120.84, 110.09, 89.20, 60.51, 46.96, 37.51, 31.79, 29.93, 29.37, 29.20, 24.10, 22.60, 14.20, 14.03; **HRMS ESI** Calcd for $C_{24}H_{34}O_4Na$ $[M+Na]^+$: 409.2349, Found: 409.2341; **IR (neat)**: 2956, 2927, 2856, 1720, 1596, 1477, 1460, 1368, 1300, 1262, 1177, 1044, 751 cm^{-1}; **EI MS** m/z (%): 227 (100), 241 (13), 243 (26), 328 (17), 386 (14); Enantiomeric excess is determined by chiral HPLC (IF-3, *n*-Hexane/*i*-PrOH = 95/5, flow rate = 1.0 mL/min, 220 nm) , t_R (minor) = 8.9 min, t_R (major) = 10.3 min.

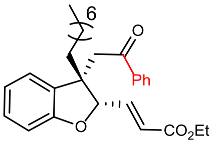

9d

Compound **9d** (71% yield, 93% ee, 9.3:1 dr, colorless oil): $[\alpha]_D^{28}$ = −60 (c = 0.50, $CHCl_3$); **^{1}H NMR** (400 MHz, $CDCl_3$) δ 7.87 – 7.80 (m, 2H), 7.53 (t, *J* = 7.4 Hz, 1H), 7.41 (t, *J* = 7.7 Hz, 2H), 7.19 – 7.13 (m, 1H), 7.11 – 7.04 (m, 1H), 6.95 – 6.82 (m, 3H), 6.12 (dd, *J* = 15.7, 1.5 Hz, 1H), 5.36 (dd, *J* = 5.4, 1.5 Hz, 1H), 4.08 (q, *J* = 7.1 Hz, 2H), 3.66 (d, *J* = 17.6 Hz, 1H), 2.99 (d, *J* = 17.6 Hz, 1H), 2.03 – 1.91 (m, 2H), 1.28 – 1.13 (m, 14H), 1.10 – 0.99 (m, 1H), 0.83 (t, *J* = 7.0 Hz, 3H); **^{13}C NMR** (100 MHz, $CDCl_3$) δ 198.06, 165.86, 158.00, 142.97, 137.31, 133.12, 132.47, 128.74, 128.53, 127.88, 124.11, 123.37, 120.76, 110.10, 89.49, 60.39, 51.38, 41.82, 37.72, 31.74, 29.87, 29.31, 29.15, 24.01, 22.57, 14.08, 14.03; **HRMS ESI** Calcd for $C_{29}H_{36}O_4Na$ $[M+Na]^+$: 471.2506, Found: 471.2494; **IR (neat)**: 2927, 2855, 1719, 1688, 1596, 1477, 1460, 1367, 1301, 1266, 1180, 992, 741cm^{-1}; **EI MS** m/z (%): 105 (83), 207 (28), 289 (32), 335 (100), 448 (2); Enantiomeric excess is determined by chiral HPLC (IE-3, *n*-Hexane/*i*-PrOH = 95/5, flow rate = 1.0 mL/min, 220 nm), t_R (major) = 9.1 min, t_R (minor) = 10.4 min.

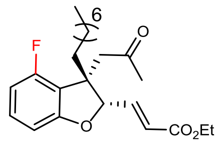

9e

Compound **9e** (78% yield, 95% ee, >20:1 dr, colorless oil): $[\alpha]_D^{25}$ = −92 (c = 0.50, $CHCl_3$); **^{1}H NMR** (600 MHz, $CDCl_3$) δ 7.12 (td, *J* = 8.2, 5.8 Hz, 1H), 6.79 (dd, *J* = 15.7, 5.8 Hz, 1H), 6.65 (d, *J* = 8.0 Hz, 1H), 6.58 (t, *J* = 8.9 Hz, 1H), 6.01 (dd, *J* = 15.7, 1.4 Hz, 1H), 5.29 (dd, *J* = 5.8, 1.3 Hz, 1H), 4.16 (q, *J* = 7.1 Hz, 2H), 3.40 (d, *J* = 18.4 Hz, 1H), 2.76 (d, *J* = 18.5 Hz, 1H), 2.11 (s, 3H), 1.94 – 1.86 (m, 2H), 1.27 – 1.18 (m, 14H), 1.08 – 1.02 (m, 1H), 0.85 (t, *J* = 7.1 Hz, 3H); **^{13}C NMR** (150 MHz, $CDCl_3$) δ 206.41, 165.83, 160.13 (d, *J* = 9.6 Hz), 159.90 (d, *J* = 246.5 Hz), 142.42, 130.15 (d, *J* = 9.1 Hz), 123.70, 118.16 (d, *J* = 18.1 Hz), 108.39 (d, *J* = 21.7 Hz) 106.30, 89.22, 60.53, 52.18, 46.62, 38.00, 31.78, 30.77, 29.84, 29.35, 29.16, 24.31, 22.59, 14.19, 14.00; **^{19}F NMR** (564 MHz, $CDCl_3$) δ -120.00; **HRMS ESI** Calcd for $C_{24}H_{33}FO_4Na$ $[M+Na]^+$: 427.2255, Found: 427.2249; **IR (neat)**: 2928, 2855, 1718, 1625, 1601, 1460, 1368, 1180, 1018, 739 cm^{-1}; **EI MS** m/z (%): 245 (100), 301 (21), 346 (53), 404 (8); Enantiomeric excess is determined by chiral HPLC (ID-3, *n*-Hexane/*i*-PrOH = 95/5, flow rate = 1.0 mL/min, 220 nm), t_R (major) = 5.5 min, t_R (minor) = 6.0 min.

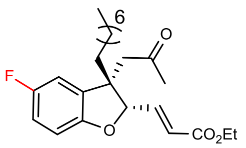

9f

Compound **9f** (78% yield, 95% ee, 12.2:1 dr, colorless oil): $[\alpha]_D^{25}$ = −45 (c = 0.40, $CHCl_3$); **^{1}H NMR** (400 MHz, $CDCl_3$) δ 6.87 – 6.74 (m, 4H), 6.08 (dd, *J* = 15.7, 1.5 Hz, 1H), 5.21 (dd, *J* = 5.4, 1.4 Hz, 1H), 4.18 (q, *J* = 7.1 Hz, 2H), 2.94 (d, *J* = 18.0 Hz, 1H), 2.56 (d, *J* = 18.0 Hz, 1H), 2.08 (s, 3H), 1.94 – 1.80 (m, 2H), 1.29 – 1.19 (m, 14H), 1.10 – 0.98 (m, 1H), 0.86 (t,

J = 6.9 Hz, 3H); **^{13}C NMR** (100 MHz, $CDCl_3$) δ 206.01, 165.89, 157.62 (d, J = 238.2 Hz), 153.87, 142.43, 133.79 (d, J = 7.9 Hz), 123.46, 114.89 (d, J = 24.2 Hz) 111.48 (d, J = 25.0 Hz), 110.26 (d, J = 8.4 Hz), 89.72, 60.59, 51.28, 46.65, 37.28, 31.76, 31.16, 29.85, 29.34, 29.18, 24.04, 22.59, 14.18, 14.04; **^{19}F NMR** (376 MHz, $CDCl_3$) δ -123.17; **HRMS ESI** Calcd for $C_{24}H_{33}FO_4Na$ $[M+Na]^+$: 427.2255, Found: 427.2245; **IR (neat)**: 2927, 2856, 2860, 1720, 1658, 1480, 1368, 1265, 1174, 1035, 740 cm^{-1}; **EI MS** m/z (%): 245 (100), 261 (89), 276 (24), 347 (14), 404 (20); Enantiomeric excess is determined by chiral HPLC (IF-3, *n*-Hexane/*i*-PrOH = 95/5, flow rate = 1.0 mL/min, 220 nm) , t_R (minor) = 5.9 min, t_R (major) = 6.9 min.

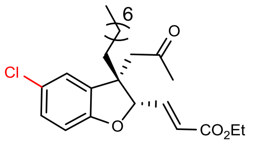

9g

Compound **9g** (74% yield, 96% ee, 11.3:1 dr, colorless oil): $[\alpha]_D^{27}$ = −70 (c = 0.50, $CHCl_3$); **^{1}H NMR** (600 MHz, $CDCl_3$) δ 7.12 (dd, J = 8.4, 1.6 Hz, 1H), 7.03 (s, 1H), 6.84 – 6.74 (m, 2H), 6.06 (d, J = 15.6 Hz, 1H), 5.23 (d, J = 5.0 Hz, 1H), 4.17 (q, J = 7.1 Hz, 2H), 2.96 (d, J = 18.1 Hz, 1H), 2.58 (d, J = 18.1 Hz, 1H), 2.09 (s, 3H), 1.95 – 1.78 (m, 2H), 1.28 – 1.19 (m, 14H), 1.06 – 0.98 (m, 1H), 0.86 (t, J = 7.1 Hz, 3H); **^{13}C NMR** (150 MHz, $CDCl_3$) δ 205.81, 165.78, 156.74, 142.22, 134.33, 128.66, 125.67, 124.34, 123.58, 111.03, 89.80, 60.57, 51.28, 46.78, 37.58, 31.76, 31.06, 29.83, 29.31, 29.17, 23.98, 22.59, 14.17, 14.01; **HRMS ESI** Calcd for $C_{24}H_{33}ClO_4Na$ $[M+Na]^+$: 443.1960, Found: 443.1949; **IR (neat)**: 2927, 2856, 1719, 1469, 1367, 1305, 1264, 1179, 1035, 740 cm^{-1}; **EI MS** m/z (%): 261 (100), 277 (85), 292 (27), 363 (15), 420 (22); Enantiomeric excess is determined by chiral HPLC (IF-3, *n*-Hexane/*i*-PrOH = 98/2, flow rate = 1.0 mL/min, 220 nm) , t_R (minor) = 8.6 min, t_R (major) = 9.7 min.

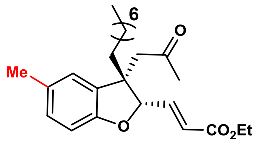

9h

Compound **9h** (87% yield, 95% ee, 17.8:1 dr, colorless oil): $[\alpha]_D^{27}$ = −60 (c = 0.40, $CHCl_3$); **^{1}H NMR** (600 MHz, $CDCl_3$) δ 6.95 (d, J = 8.1 Hz, 1H), 6.87 – 6.80 (m, 2H), 6.73 (d, J = 8.1 Hz, 1H), 6.08 (dd, J = 15.6, 1.5 Hz, 1H), 5.17 (dd, J = 5.4, 1.5 Hz, 1H), 4.17 (q, J = 7.1 Hz, 2H), 2.97 (d, J = 17.7 Hz, 1H), 2.54 (d, J = 17.7 Hz, 1H), 2.29 (s, 3H), 2.05 (s, 3H), 1.93 – 1.80 (m, 2H), 1.29 – 1.16 (m, 14H), 1.18 – 0.99 (m, 1H), 0.86 (t, J = 7.1 Hz, 3H); **^{13}C NMR** (150 MHz, $CDCl_3$) δ 206.44, 165.97, 155.99, 143.02, 132.29, 130.10, 129.11, 124.53, 123.23, 109.59, 89.26, 60.45, 51.12, 46.98, 37.51, 31.78, 31.25, 29.93, 29.34, 29.19, 24.06, 22.60, 20.89, 14.19, 14.01; **HRMS ESI** Calcd for $C_{25}H_{36}O_4Na$ $[M+Na]^+$: 423.2506, Found: 423.2497; **IR (neat)**: 2926, 2855, 1720, 1657, 1489, 1367, 1265, 1177, 1036, 739 cm^{-1}; **EI MS** m/z (%): 241 (100), 257 (34), 343 (17),

400 (23); Enantiomeric excess is determined by chiral HPLC (IE-3, *n*-Hexane/*i*-PrOH = 95/5, flow rate = 1.0 mL/min, 220 nm), t_R (major) = 7.8 min, t_R (minor) = 8.9 min.

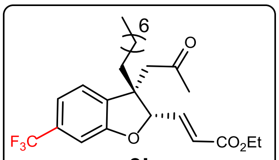

9i

Compound **9i** (78% yield, 96% ee, 13.9:1 dr, colorless oil): $[\alpha]_D^{25}$ = −40 (c = 0.40, $CHCl_3$); **^{1}H NMR** (400 MHz, $CDCl_3$) δ 7.17 (d, *J* = 0.8 Hz, 2H), 7.08 (s, 1H), 6.82 (dd, *J* = 15.7, 5.4 Hz, 1H), 6.09 (dd, *J* = 15.7, 1.5 Hz, 1H), 5.27 (dd, *J* = 5.4, 1.5 Hz, 1H), 4.18 (q, *J* = 7.1 Hz, 2H), 3.00 (d, *J* = 18.1 Hz, 1H), 2.59 (d, *J* = 18.1 Hz, 1H), 2.09 (s, 3H), 1.96 – 1.83 (m, 2H), 1.30 – 1.16 (m, 14H), 1.18 – 0.97 (m, 1H), 0.86 (t, *J* = 6.9 Hz, 3H); **^{13}C NMR** (150 MHz, $CDCl_3$) δ 205.67, 165.75, 158.37, 141.86, 136.54, 131.42 (q, *J* = 32.4 Hz), 124.55, 123.99 (q, *J* = 272.2 Hz), 123.80, 118.10, 107.21, 89.94, 60.64, 51.05, 46.68, 37.40, 31.76, 31.10, 29.83, 29.31, 29.15, 24.04, 22.58, 14.18, 13.98; **^{19}F NMR** (376 MHz, $CDCl_3$) δ -62.30; **HRMS ESI** Calcd for $C_{25}H_{33}F_3O_4Na$ $[M+Na]^+$: 477.2223, Found: 477.2215; **IR (neat)**: 2928, 2857, 1721, 1435, 1324, 1268, 1168, 1126 cm^{-1}; **EI MS** m/z (%): 295 (100), 311 (53), 351 (18), 397 (30), 454 (8); Enantiomeric excess is determined by chiral HPLC (IF-3, *n*-Hexane/*i*-PrOH = 95/5, flow rate = 1.0 mL/min, 220 nm), t_R (minor) = 4.8 min, t_R (major) = 5.2 min.

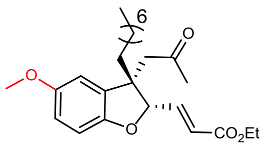

9j

Compound **9j** (82% yield, 89% ee, 13.6:1 dr, colorless oil): $[\alpha]_D^{27}$ = −60 (c = 0.30, $CHCl_3$); **^{1}H NMR** (600 MHz, $CDCl_3$) δ 6.84 (dd, *J* = 15.6, 5.5 Hz, 1H), 6.75 (d, *J* = 8.6 Hz, 1H), 6.70 (dd, *J* = 8.6, 2.6 Hz, 1H), 6.64 (d, *J* = 2.5 Hz, 1H), 6.08 (d, *J* = 15.6 Hz, 1H), 5.18 (d, *J* = 4.3 Hz, 1H), 4.17 (q, *J* = 7.1 Hz, 2H), 3.76 (s, 3H), 2.96 (d, *J* = 17.8 Hz, 1H), 2.54 (d, *J* = 17.8 Hz, 1H), 2.06 (s, 3H), 1.94 – 1.83 (m, 2H), 1.28 – 1.18 (m, 14H), 1.09 – 0.99 (m, 1H), 0.86 (t, *J* = 7.1 Hz, 3H); **^{13}C NMR** (150 MHz, $CDCl_3$) δ 206.33, 165.98, 154.43, 152.12, 142.93, 133.42, 123.32, 113.40, 110.78, 109.97, 89.40, 60.48, 56.05, 51.44, 46.86, 37.32, 31.78, 31.26, 29.94, 29.36, 29.20, 24.12, 22.60, 14.19, 14.02; **HRMS ESI** Calcd for $C_{25}H_{36}O_5Na$ $[M+Na]^+$: 439.2455, Found: 439.2449; **IR (neat)**: 2927, 2855, 1720, 1485, 1367, 1268, 1177, 1034 cm^{-1}; **EI MS** m/z (%): 257 (100), 273 (53), 359 (21), 416 (67); Enantiomeric excess is determined by chiral HPLC (IF-3, *n*-Hexane/*i*-PrOH = 95/5, flow rate = 1.0 mL/min, 220 nm), t_R (minor) = 7.4 min, t_R (major) = 8.5 min.

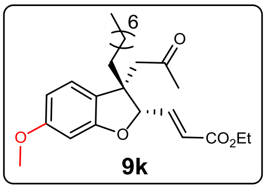

Compound **9k** (65% yield, 88% ee, 19:1 dr, colorless oil): $[\alpha]_D^{28}$ = −13 (c = 0.15, $CHCl_3$); **^{1}H NMR** (600 MHz, $CDCl_3$) δ 6.92 (d, *J* = 8.3 Hz, 1H), 6.85 (dd, *J* = 15.6, 5.4 Hz, 1H), 6.47 – 6.39 (m, 2H), 6.09 (dd, *J* = 15.7, 1.5 Hz, 1H), 5.19 (dd, *J* = 5.4, 1.5 Hz, 1H), 4.17 (q, *J* = 7.1 Hz, 2H), 3.77 (s, 3H), 2.94 (d, *J* = 17.7 Hz, 1H), 2.51 (d, *J* = 17.7 Hz, 1H), 2.04 (s, 3H), 1.89 – 1.82 (m, 2H), 1.28 – 1.19 (m, 14H), 1.08 – 1.01 (m, 1H), 0.86 (t, *J* = 7.1 Hz, 3H); **^{13}C NMR** (150 MHz, $CDCl_3$) δ 206.52, 165.96, 160.86, 159.32, 142.87, 124.42, 124.23, 123.25, 106.48, 96.68, 89.85, 60.50, 55.48, 50.61, 47.14, 37.60, 31.79, 31.29, 29.94, 29.39, 29.20, 24.15, 22.60, 14.21, 14.01; **HRMS ESI** Calcd for $C_{25}H_{36}O_5Na$ $[M+Na]^+$: 439.2455, Found: 439.2456; **IR (neat)**: 2927, 2855, 1719, 1622, 1596, 1497, 1367, 1268, 1149, 739 cm^{-1}; **EI MS** m/z (%): 257 (100), 303 (6), 359 (26), 416 (12); Enantiomeric excess is determined by chiral HPLC (IF-3, *n*-Hexane/*i*-PrOH = 95/5, flow rate = 1.0 mL/min, 220 nm), t_R (minor) = 7.2 min, t_R (major) = 8.7 min.

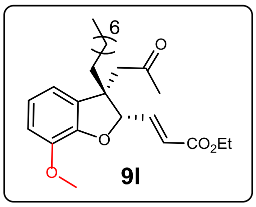

Compound **9l** (82% yield, 96% ee, 13.3:1 dr, colorless oil): $[\alpha]_D^{25}$ = −23 (c = 0.40, $CHCl_3$); **^{1}H NMR** (400 MHz, $CDCl_3$) δ 6.88 – 6.77 (m, 3H), 6.67 (d, *J* = 7.4 Hz, 1H), 6.12 (d, *J* = 15.7 Hz, 1H), 5.25 (d, *J* = 5.7 Hz, 1H), 4.17 (q, *J* = 7.0 Hz, 2H), 3.89 (s, 3H), 2.98 (d, *J* = 17.6 Hz, 1H), 2.55 (d, *J* = 17.6 Hz, 1H), 2.04 (s, 3H), 1.94 – 1.81 (m, 2H), 1.28 – 1.17 (m, 14H), 1.09 – 0.97 (m, 1H), 0.85 (t, *J* = 6.9 Hz, 3H); **^{13}C NMR** (100 MHz, $CDCl_3$) δ 206.47, 165.99, 146.16, 144.80, 142.41, 133.49, 123.48, 121.49, 116.16, 111.86, 89.85, 60.50, 55.97, 51.54, 46.89, 37.25, 31.77, 31.34, 29.90, 29.35, 29.19, 24.08, 22.59, 14.18, 14.04; **HRMS ESI** Calcd for $C_{25}H_{36}O_5Na$ $[M+Na]^+$: 439.2455, Found: 439.2454; **IR (neat)**: 2927, 2855, 1720, 1491, 1459, 1270, 1177 cm^{-1}; **EI MS** m/z (%): 257 (100), 273 (56), 303 (45), 358 (28), 416 (29); Enantiomeric excess is determined by chiral HPLC (IE-3, *n*-Hexane/*i*-PrOH = 95/5, flow rate = 1.0 mL/min, 220 nm), t_R (major) = 14.9 min, t_R (minor) = 17.8 min.

Supplementary Note 5

Asymmetric Total Synthesis of (–)-Codeine and (–)-Morphine:

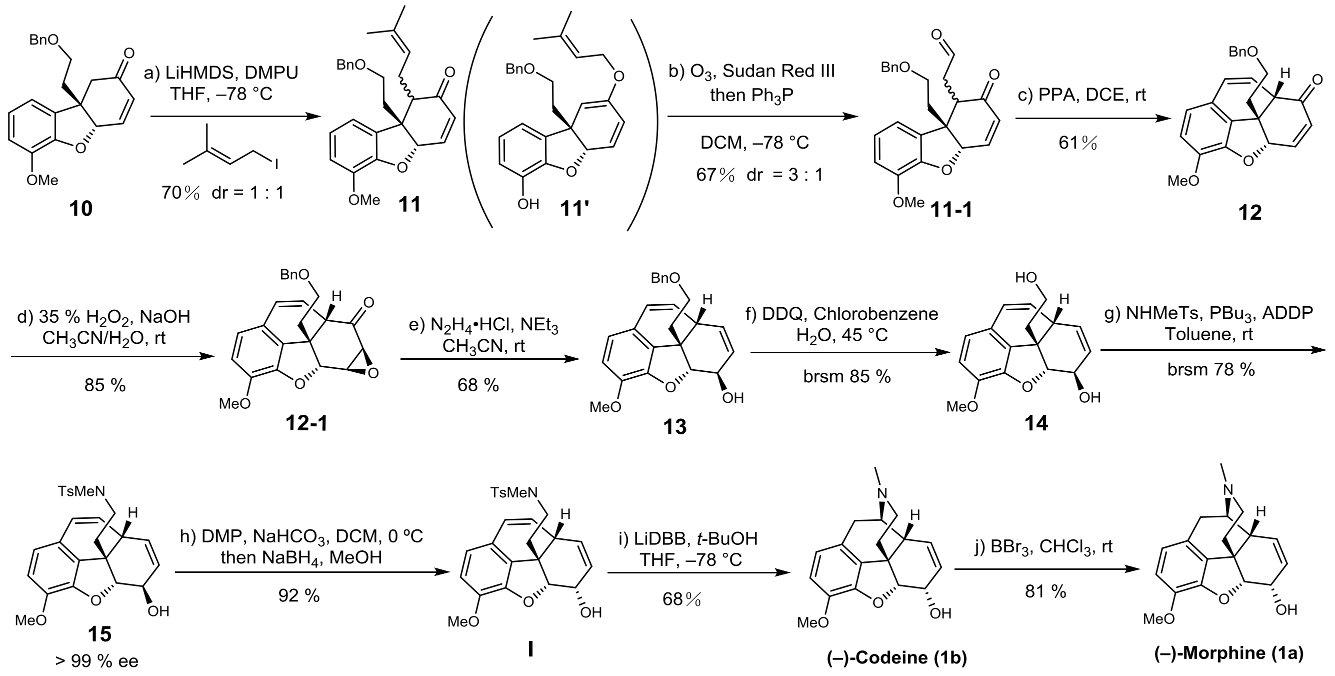

Supplementary Figure 3. **The synthetic route**

The Synthesis of Compound 11 and 11'

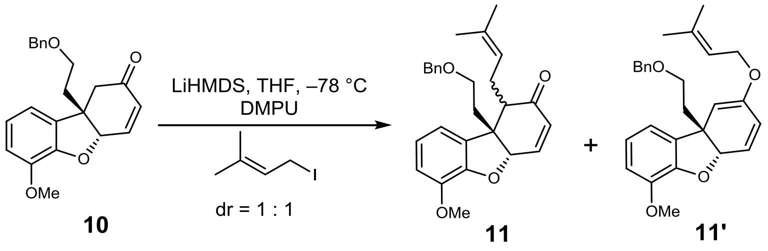

Under argon atmosphere, to a stirred solution of **10** (2.0 g, 5.71 mmol, 1 eq.) in dry THF (90 mL) at −78 ℃ was slowly added (over ca. 2 mins) LiHMDS (6.6 mL, 6.57 mmol, 1 M in THF/ethylbenzene, 1.15 eq.). After stirred for 50 min at −78 ℃, DMPU (897 uL, 7.42 mmol, 1.3 eq.) was added. 5 min later, the freshly prepared 3,3-dimethylallyl iodide (1.13 mL, 8.57 mmol, 1.5 eq.) was added. Then the reaction was warmed to room temperature (over ca. 20 min) under argon and stirred for 6 h in dark. Finally, the mixture was quenched with saturated $Na_2S_2O_3$ solution and extracted with EtOAc. The combined organic layer was washed with saturated $Na_2S_2O_3$ solution and brine, dried with Na_2SO_4 and concentrated *in vacuum*. The crude product was purified by column chromatography on silica gel (petroleum ether: ethyl acetate = 10:1) to give product **11** (1.67 g, 70%, dr = 1:1, colorless oil) as a inseparable mixture of isomers and compound **11'** (692 mg, 29% yield, colorless oil).

Note: The 3,3-dimethylallyl iodide must be **freshly** prepared according to the reported literature procedure[12], otherwise, it would give inferior yield.

Storage conditions: The 3,3-dimethylallyl iodide was relatively stable for about 24 h, when it was stored in refrigerator at −20 ℃ in the presence of copper powder and protected from light.

Compound **11**: $[\alpha]_D^{25}$ = −50 (c = 0.20, $CHCl_3$); **^{1}H NMR** (400 MHz, $CDCl_3$) δ 7.37 – 7.17 (m, 5H), 6.96 – 6.74 (m, 2.5H), 6.73 – 6.61 (m, 1.5H), 6.07 (d, *J* = 10.2 Hz, 0.5H), 6.00 (d, *J* = 10.2 Hz, 0.5H), 5.33 (d, *J* = 3.3 Hz, 0.5H), 5.16 (d, *J* = 3.6 Hz, 0.5H), 5.03 (t, *J* = 7.2 Hz, 0.5H), 4.81 (t, *J* = 7.3 Hz, 0.5H), 4.44 – 4.30 (m, 2H), 3.89 (s, 1.5H), 3.86 (s, 1.5H), 3.59 – 3.46 (m, 1H), 3.31 – 3.47 (m, 1H), 2.94 (dd, *J* = 10.5, 3.5 Hz, 0.5H), 2.58 – 2.49 (m, 0.5H), 2.40 (dd, *J* = 11.2, 3.7 Hz, 0.5H), 2.35 – 2.24 (m, 1H), 2.24 – 2.12 (m, 1H), 2.11 – 1.96 (m, 1H), 1.63 (s, 1.5H), 1.58 (s, 1.5H), 1.47 (s, 1.5H), 1.39 (s, 1.5H); **^{13}C NMR** (100 MHz, $CDCl_3$) δ 200.95, 200.27, 146.72, 145.11, 144.76, 139.47, 139.32, 137.87, 137.81, 133.43, 133.18, 132.79, 131.51, 130.53, 129.20, 128.27, 128.24, 127.54, 127.48, 127.39, 122.09, 121.80, 121.00, 120.94, 116.10, 115.48, 112.02, 111.72, 83.59, 81.63, 73.08, 72.86, 66.44, 66.12, 55.78, 55.73, 52.49, 52.15, 51.33, 37.73, 33.82, 28.11, 26.13, 25.66, 25.59, 17.64, 17.60; **HRMS ESI** Calcd for $C_{27}H_{30}O_4Na$ $[M+Na]^+$: 441.2042, Found: 441.2031; **IR (neat)**: 2921, 2856, 1687, 1619, 1490, 1455, 1281, 1197, 1102, 1067, 733 cm^{-1}; **EI MS** m/z (%): 91 (100), 109 (15), 215 (12), 283 (28), 327 (14), 418 (16).

Compound **11'**: $[\alpha]_D^{25}$ = −40 (c = 0.20, $CHCl_3$); **^{1}H NMR** (400 MHz, $CDCl_3$) δ 7.35 – 7.23 (m, 6H), 6.85 (t, *J* = 7.8 Hz, 1H), 6.76 (dd, *J* = 7.5, 1.0 Hz, 1H), 6.70 (dd, *J* = 8.1, 0.9 Hz, 1H), 6.08 – 5.97 (m, 2H), 5.39 – 5.33 (m, 1H), 5.31 (d, *J* = 4.0 Hz, 1H), 4.56 (s, 1H), 4.41 (s, 2H), 4.19 – 4.08 (m, 2H), 3.85 (s, 3H), 3.58 (ddd, *J* = 9.5, 6.9, 5.4 Hz, 1H), 3.48 (dt, *J* = 9.5, 7.1 Hz, 1H), 2.26 – 2.07 (m, 2H), 1.75 (s, 3H), 1.65 (s, 3H); **^{13}C NMR** (100 MHz, $CDCl_3$) δ 149.91, 146.25, 144.48, 138.10, 138.00, 135.38, 128.19, 127.37, 127.33, 127.30, 123.68, 121.41, 119.11, 114.97, 110.32, 98.95, 84.14, 72.91, 66.50, 63.52, 55.62, 47.96, 40.95, 25.71, 18.05; **HRMS ESI** Calcd for $C_{27}H_{30}O_4Na$ $[M+Na]^+$: 441.2042, Found: 441.2037; **IR (neat)**: 2928, 2856, 1663, 1617, 1491, 1455, 1276, 1198, 1101, 732 cm^{-1}; **EI MS** m/z (%): 91 (100), 215 (28), 241 (24), 350 (25), 418 (7).

Experiments in Alkylation Reaction at α-position of ketone

The Failed Approaches

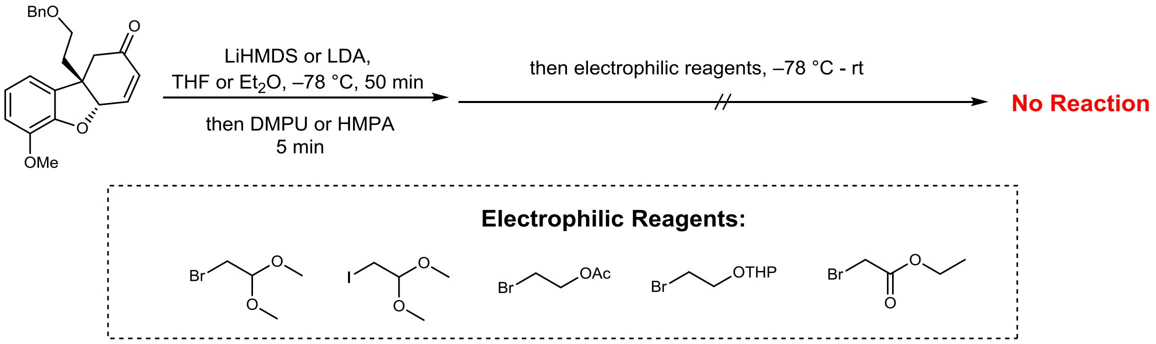

Supplementary Table 7. Conditions Optimization[a]

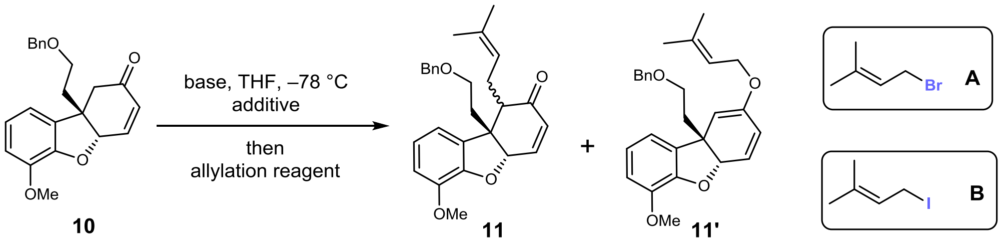

entry	reagent	base	additive	yield (11)	yield (11')[b]	d.r.[c]
1	A	LiHMDS	–	15%	5%	1:1
2	A	KHMDS	–	trace	40%	1:1
3	A	LiHMDS	HMPA	15%	47%	1:1
4	A	LiHMDS	DMPU	33%	24%	1:1
**5**	**B**	**LiHMDS**	**DMPU**	**70%**	**29%**	**1:1**

[a] Reaction was performed by following the above mentioned method for the synthesis of compound **11**; [b] Isolated yield; [c] The d.r. value was determined by ^{1}H NMR.

The Synthesis of Compound 10

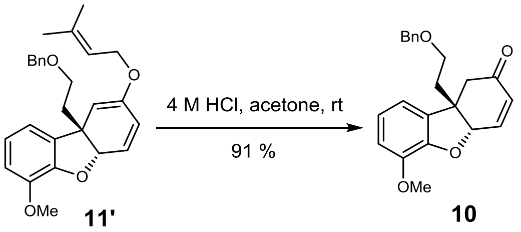

To a stirred solution of **11'** (200 mg, 0.478 mmol) in acetone (8 mL) at 0 ℃ was slowly added HCl aqueous solution (2.0 mL, 4 M). Then the reaction was warmed to room temperature and stirred for 30 min. The mixture was extracted with EtOAc. The combined organic layer was washed with saturated $NaHCO_3$ solution and brine, dried with $MgSO_4$ and concentrated *in vacuo*. The crude product was purified by column chromatography on silica gel (petroleum ether: ethyl acetate = 10:1) to give product **10** (152 mg, 91%) as a colorless oil.

The Synthesis of Compound 11-1

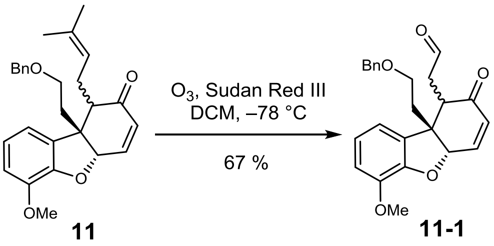

To a stirred solution of **11** (90 mg, 0.22 mmol, 1 eq.) in DCM (5 mL) at −78 ºC was added Sudan Red III (20 uL, 1 mg/mL in DCM, CAS: 85-86-9). Then ozone was **slowly** bubbled through the reaction mixture until a colour change from red/pink to pale yellow was observed. After purged with argon at −78 ºC for 10 min, the reaction was quenched with PPh_3 (173 mg, 0.66 mmol, 3 eq.) and stirred for 30 min at room temperature. Finally, the reaction mixture was directly purified by flash column chromatography on silica gel (petroleum ether: ethyl acetate = 8:1) to give product **11-1** (58 mg, 67%, dr = 1.7:1, a inseparable mixture of isomers) as a colorless oil.

Note: The product **11-1** would be decomposed if ozone was bubbled for a long time.

Compound **11-1**: $[\alpha]_D^{25}$ = −85 (c = 0.10, $CHCl_3$); **^{1}H NMR** (400 MHz, $CDCl_3$) δ 9.73 (d, *J* = 4.7 Hz, 0.4H), 9.71 (d, *J* = 1.6 Hz, 0.6H), 7.36 – 7.24 (m, 3.7H), 7.23 – 7.16 (m, 1.2H), 6.98 (dd, *J* = 10.2, 4.8 Hz, 0.6H), 6.92 – 6.76 (m, 2H), 6.76 – 6.64 (m, 1.4H), 6.26 (d, *J* = 10.2 Hz, 0.6H), 6.11 (d, *J* = 10.2 Hz, 0.4H), 5.59 (dd, *J* = 3.3, 1.1 Hz, 0.4H), 5.18 (d, *J* = 4.8 Hz, 0.6H), 4.48 – 4.39 (m, 1.2H), 4.32 (q, *J* = 11.8 Hz, 1.3H), 3.90 (s, 1.8H), 3.86 (s, 1.1H), 3.61 (ddd, *J* = 15.7, 10.5, 4.2 Hz, 0.7H), 3.54 – 3.36 (m, 2.2H), 3.29 (dd, *J* = 16.3, 9.6 Hz, 0.4H), 2.99 (ddd, *J* = 17.0, 10.7, 1.8 Hz, 0.6H), 2.69 (dd, *J* = 17.4, 2.6 Hz, 0.4H), 2.38 (dd, *J* = 17.0, 2.5 Hz, 0.6H), 2.21 – 2.03 (m, 1.1H), 2.03 – 1.86 (m, 1H); **^{13}C NMR** (100 MHz, $CDCl_3$) δ 199.61, 199.25, 197.55, 197.18, 146.53, 145.47, 145.11, 145.07, 141.47, 139.35, 137.63, 137.49, 133.08, 131.62, 131.03, 130.10, 128.33, 128.29, 127.65, 127.60, 127.57, 127.42, 122.33, 121.86, 115.66, 115.13, 112.16, 112.04, 82.92, 82.12, 73.17, 72.81, 66.18, 65.99, 55.84, 55.75, 52.15, 51.66, 47.24, 45.67, 39.38, 37.50, 37.15, 31.29; **HRMS ESI** Calcd for $C_{24}H_{24}O_5Na$ $[M+Na]^+$: 415.1521, Found: 415.1511; **IR (neat)**: 2933, 2859, 1724, 1687, 1490, 1455, 1282, 1205, 1095, 737 cm^{-1}; **EI MS** m/z (%): 91 (100), 239 (14), 257 (27), 283 (13), 301 (7), 392 (12).

The Synthesis of Compound 12

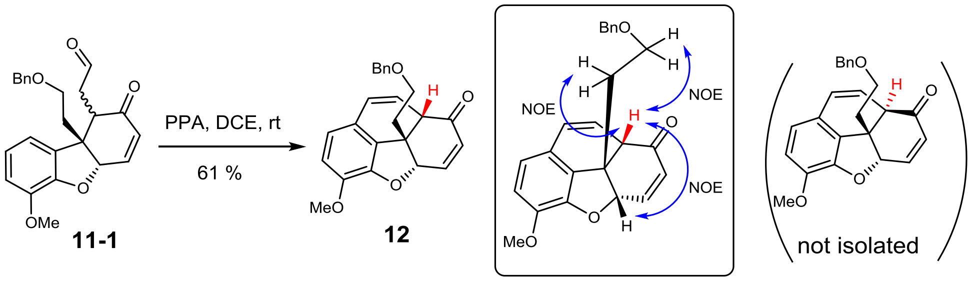

To a stirred solution of **11-1** (735 mg, 1.88 mmol, 1 eq.) in dry DCE (150 mL, 5 mg/mL) at 0 ℃ was added polyphosphoric acids (PPA, 318 mg, 0.94 mmol, 0.5 eq.). Then the reaction was warmed to room temperature and stirred for 24 h under argon. After completion, the solution was filtered through the silica gel column and the filtrate was concentrated *in vacuo*. The crude product was purified by column chromatography on silica gel (petroleum ether: ethyl acetate = 10:1) to give product **12** (429 mg, 61%, colorless oil) as a single diastereomer.

Proposed Reaction Process

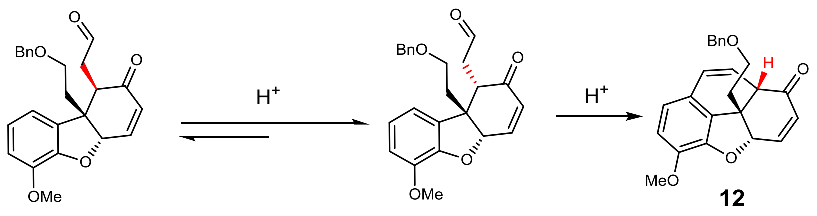

Compound **12**: $[\alpha]_D^{25}$ = +28 (c = 0.10, $CHCl_3$); **^{1}H NMR** (400 MHz, $CDCl_3$) δ 7.39 – 7.22 (m, 5H), 6.67 (dt, *J* = 15.6, 5.3 Hz, 3H), 6.55 (dd, *J* = 9.5, 0.8 Hz, 1H), 6.20 – 6.04 (m, 2H), 5.68 (dd, *J* = 3.2, 1.2 Hz, 1H), 4.46 (q, *J* = 11.8 Hz, 2H), 3.87 (s, 3H), 3.61 (dt, *J* = 11.3, 5.7 Hz, 1H), 3.53 (ddd, *J* = 10.0, 7.7, 5.2 Hz, 1H), 3.38 (d, *J* = 6.5 Hz, 1H), 2.11 (ddd, *J* = 13.3, 7.6, 5.6 Hz, 1H), 1.94 (dt, *J* = 14.5, 5.5 Hz, 1H); **^{13}C NMR** (150 MHz, $CDCl_3$) δ 197.75, 144.79, 142.84, 142.03, 138.01, 132.47, 128.74, 128.40, 127.69, 127.57, 125.21, 125.01, 123.48, 118.55, 112.88, 84.66, 73.24, 66.85, 56.21, 46.10, 44.90, 35.24; **HRMS ESI** Calcd for $C_{24}H_{22}O_4Na$ $[M+Na]^+$: 397.1416, Found: 397.1405; **IR (neat)**: 2932, 2861, 1735, 1674, 1507, 1453, 1284, 1207, 1089, 898 cm^{-1}; **EI MS** m/z (%): 91 (100), 139 (29), 195 (36), 239 (55), 266 (72), 283 (8), 374 (16).

The Synthesis of Compound 12-1

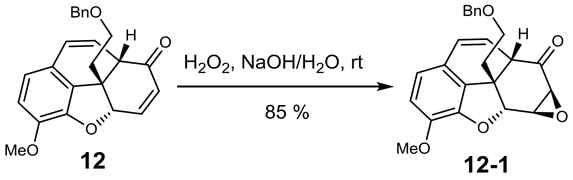

To a stirred solution of **12** (152 mg, 0.406 mmol, 1 eq.) in CH_3CN (30 mL, 5 mg/mL) at 0 ℃ was added sequentially a 35% H_2O_2 aqueous solution (175 uL, 2.03 mmol, 5 eq.) and 1% NaOH aqueous solution (812 uL, 0.203 mmol, 0.5 eq.). The reaction mixture was allowed to warm to room temperature and stirred until the starting material was not observed by TLC (about 1 h). After quenched with saturated $Na_2S_2O_3$ solution, the mixture was extracted with EtOAc and washed with brine. The combined extract was dried with Na_2SO_4 and concentrated *in vacuo*. The crude product was purified by column chromatography on silica gel (petroleum ether: ethyl acetate = 15:1) to give product **12-1** (135 mg, 85%) as a colorless oil.

Compound **12-1**: $[\alpha]_D^{18}$ = −58 (c = 0.20, $CHCl_3$); **^{1}H NMR** (400 MHz, $CDCl_3$) δ 7.40 – 7.24 (m, 5H), 6.76 – 6.60 (m, 3H), 5.77 (dd, *J* = 9.4, 6.1 Hz, 1H), 5.61 (s, 1H), 4.48 (s, 2H), 3.87 (s, 3H), 3.74 – 3.67 (m, 2H), 3.58 (dt, *J* = 10.0, 6.0 Hz, 1H), 3.54 – 3.46 (m, 1H), 3.31 (d, *J* = 3.7 Hz, 1H), 2.09 (t, *J* = 5.9 Hz, 2H); **^{13}C NMR** (100 MHz, $CDCl_3$) δ 205.22, 144.36, 144.31, 138.02, 128.35, 127.59, 127.58, 127.15, 126.62, 123.77, 123.14, 118.87, 112.90, 83.88, 73.28, 67.00, 58.19, 56.10, 55.58, 50.63, 45.46, 35.7; **HRMS ESI** Calcd for $C_{24}H_{22}O_5Na$ $[M+Na]^+$: 413.1365, Found: 413.1361; **IR (neat)**: 2920, 2850, 1716, 1508, 1455, 1285, 1093 cm^{-1}; **EI MS** m/z (%): 91 (100), 155 (18), 183 (39), 211 (22), 256 (24), 299 (11), 390 (17).

The Synthesis of Compound 13

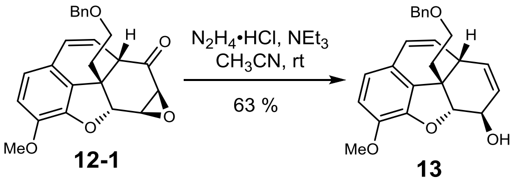

The Wharton rearrangement reaction was based on the procedure reported by Luche[13].

To a solution of **12-1** (307 mg, 0.787 mmol, 1 eq.) in dry CH_3CN (15 mL) at 25 ℃ was added N_2H_4•HCl (162 mg, 2.361 mmol, 3 eq.) and NEt_3 (493 uL, 3.54 mmol, 4.5 eq.). Then the reaction was stirred vigorously under argon at room temperature for 12 h. After completion, the mixture was concentrated *in vacuo* and purified by column chromatography on silica gel (petroleum ether: ethyl acetate = 3:1) to give product **13** (186 mg, 63%) as a colorless oil.

Compound **13**: $[\alpha]_D^{24}$ = +98 (c = 0.20, $CHCl_3$); **^{1}H NMR** (400 MHz, $CDCl_3$) δ 7.36 – 7.23 (m, 5H), 6.67 (d, *J* = 8.1 Hz, 1H), 6.62 (d, *J* = 8.1 Hz, 1H), 6.41 (dd, *J* = 9.5, 1.5 Hz, 1H), 5.79 (dd, *J* = 9.5, 5.7 Hz, 1H), 5.71 (dt, *J* = 10.3, 2.9 Hz, 1H), 5.63 – 5.54 (m, 1H), 4.83 (d, *J* = 4.5 Hz, 1H), 4.41 (s, 2H), 4.12 (brs, 1H), 3.87 (s, 3H), 3.58 – 3.44 (m, 2H), 3.33 – 3.22 (m, 1H), 2.37 (brs, 1H), 2.11 – 1.92 (m, 2H); **^{13}C NMR** (100 MHz, $CDCl_3$) δ 145.23, 144.12, 138.18, 129.52, 128.52, 128.30, 127.64, 127.57, 127.49, 123.47, 123.32, 117.82, 112.31, 95.28, 73.06, 68.73, 66.85, 56.18, 43.13, 38.86, 38.05; **HRMS ESI** Calcd for $C_{24}H_{24}O_4Na$ $[M+Na]^+$: 399.1572, Found: 399.1565; **IR (neat)**: 3392, 2923, 2850, 1507, 1452, 1264, 1093, 1059, 737 cm^{-1}; **EI MS** m/z (%): 91 (100), 115 (13), 152(15), 180 (14), 241 (18), 267 (17), 376 (6).

The Synthesis of Compound 14

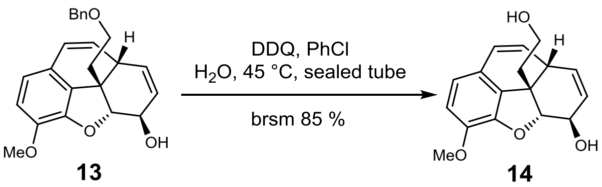

To a stirred solution of **13** (70 mg, 0.186 mmol, 1 eq.) in chlorobenzene (8 mL) was added DDQ (169 mg, 0.744 mmol, 4 eq.) and H_2O (50 uL, 2.79 mmol, 15 eq.) at room temperature. Then the reaction was sealed in argon and stirred at 45 ℃ for 4 h. After cooled to room temperature, the reaction mixture was diluted with DCM, quenched with saturated $NaHCO_3$ solution and extracted with DCM. The combined organic layer was washed with saturated $NaHCO_3$ solution, dried with Na_2SO_4 and concentrated *in vacuo*. The residue was purified by column chromatography on silica gel (petroleum ether: ethyl acetate = 1:2) to give product **14** (28 mg, 52%) as a colorless oil, together with recovered starting material **13** (23 mg, 33%) as a colorless oil.

Note: Chlorobenzene was the best solvent and gave the highest yield. Other solvents (such as DCM or DCE) would give inferior yields.

Debenzylation under the conventional conditions such as catalytic hydrogenolysis, Birch-like reductive cleavage (Li, Na or K in liquid ammonia), or Lewis/Brønsted acids ($AlCl_3$, $TiCl_4$, BCl_3, TMSI or HBr etc.) catalyzed cleavage was first investigated, and poor regioselectivity or complex mixtures were observed.

Compound **14**: $[\alpha]_D^{24}$ = +95 (c = 0.15, $CHCl_3$); **^{1}H NMR** (600 MHz, $CDCl_3$) δ 6.65 (dd, *J* = 23.4, 8.1 Hz, 2H), 6.43 (d, *J* = 9.5 Hz, 1H), 5.83 (dd, *J* = 9.5, 5.7 Hz, 1H), 5.73 (dt, *J* = 10.3, 2.8 Hz, 1H), 5.62 – 5.54 (m, 1H), 4.87 (d, *J* = 4.4 Hz, 1H), 4.18 – 4.06 (m, 1H), 3.86 (s, 3H), 3.66 – 3.56 (m, 2H), 3.28 – 3.18 (m, 1H), 2.54 (s, 2H), 2.05 – 1.99 (m, 1H), 1.97 – 1.86 (m, 1H); **^{13}C NMR** (150 MHz, $CDCl_3$) δ 145.36, 144.36, 129.36, 128.40, 127.92, 127.41, 123.86, 123.35, 118.15, 112.99, 95.53, 68.64, 59.41, 56.39, 43.37, 41.42, 39.51; **HRMS ESI** Calcd for $C_{17}H_{18}O_4Na$ $[M+Na]^+$: 309.1097, Found: 309.1103; **IR (neat)**: 3363, 2962, 2926, 1633, 1506, 1438, 1261, 1104, 1054, 794 cm^{-1}; **EI MS** m/z (%): 115 (71), 153 (98), 241 (60), 286 (78).

The Synthesis of Compound 15

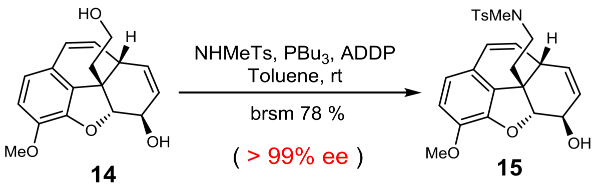

Under argon atmosphere, to a stirred solution of **14** (18.2 mg, 0.064 mmol, 1 eq.) in dry toluene (2.5 mL, freshly distilled from Na) at 0 ℃ was added *N*-methylbenzenesulfonamide (NHMeTs, 13 mg, 0.07 mmol, 1.1eq.), tributylphosphane (PBu_3, 19 uL, 0.076 mmol, 1.2 eq.) and 1,1′-(azodicarbonyl)dipiperidine (ADDP, 19 mg, 0.076 mmol, 1.2 eq.). Then the reaction mixture was warmed to room temperature and stirred for 12 h under argon. After completion, the mixture was directly purified by column chromatography on silica gel (petroleum ether: ethyl acetate = 3:1) to give product **15** (14.5 mg, 50%) as a colorless oil, together with recovered starting material **14** (5.1 mg, 28%) as a colorless oil.

Compound **15** (> 99% ee): Enantiomeric excessis was determined by chiral HPLC (IE-3, *n*-Hexane/*i*-PrOH = 65/35, flow rate = 1.0 mL/min, 220 nm), t_R (major) = 35.9 min, t_R (minor) = 42.9 min.

$[\alpha]_D^{25}$ = +55 (c = 0.20, $CHCl_3$); **^{1}H NMR** (400 MHz, $CDCl_3$) δ 7.58 (d, *J* = 8.3 Hz, 2H), 7.28 (d, *J* = 8.0 Hz, 2H), 6.67 (dd, *J* = 12.0, 7.4 Hz, 1H), 6.63 (d, *J* = 8.1 Hz, 1H), 6.42 (dd, *J* = 9.5, 1.6 Hz, 1H), 5.83 (dd, *J* = 9.5, 5.7 Hz, 1H), 5.73 (dt, *J* = 10.3, 2.9 Hz, 1H), 5.59 (ddd, *J* = 10.3, 3.2, 2.0 Hz, 1H), 4.70 (d, *J* = 4.5 Hz, 1H), 4.16 – 4.07 (m, 1H), 3.87 (s, 3H), 3.26 – 3.16 (m,

1H), 3.10 – 2.97 (m, 2H), 2.63 (s, 3H), 2.42 (s, 3H), 2.01 – 1.94 (m, 1H), 1.88 – 1.81 (m, 1H); **^{13}C NMR** (100 MHz, $CDCl_3$) δ 145.32, 144.10, 143.27, 134.54, 129.63, 128.67, 128.26, 127.92, 127.30, 127.25, 123.85, 123.24, 118.12, 112.68, 94.93, 68.54, 56.22, 46.07, 43.17, 38.75, 36.44, 34.80, 21.46; **HRMS ESI** Calcd for $C_{25}H_{27}NO_5SNa$ $[M+Na]^+$: 476.1502, Found: 476.1503; **IR (neat)**: 3427, 2924, 1506, 1336, 1279, 1159, 1047 cm^{-1}; **EI MS** m/z (%): 91 (100), 155 (57), 241 (48), 298 (15), 453 (3).

The Synthesis of Compound I

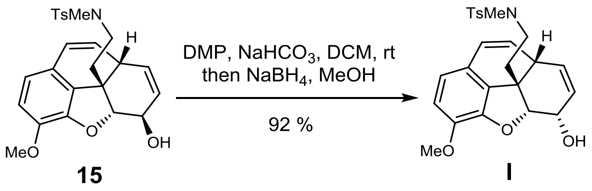

To a stirred solution of alcohol **15** (58 mg, 0.128 mmol, 1 eq.) in dry DCM (20 mL) at 0 ℃ was added $NaHCO_3$ (54 mg, 0.64 mmol, 5 eq.) and Dess-Martin reagent (81 mg, 0.192 mmol, 1.5 eq.). The reaction mixture was slowly warmed to room temperature and stirred for 1 h. Then MeOH (10 mL) was added and the reaction was cooled to 0 ℃. Subsequently, $NaBH_4$ (14.5 mg, 0.384 mmol, 3 eq.) was added. The reaction was stirred at 0 ℃ for 15 min, then quenched with saturated NaS_2O_3 solution and exracted with EtOAc. The combined organic layer was washed brine, dried with Na_2SO_4 and concentrated *in vacuum*. The crude product was purified by column chromatography on silica gel (petroleum ether: ethyl acetate = 3:1) to give product **I** (53.5 mg, 92% yield) as a colorless oil.

Compound **I**: $[\alpha]_D^{25}$ = −110 (c = 0.10, $CHCl_3$); **^{1}H NMR** (400 MHz, $CDCl_3$) δ 7.61 (d, *J* = 8.2 Hz, 2H), 7.29 (d, *J* = 8.0 Hz, 2H), 6.61 (dd, *J* = 20.2, 8.0 Hz, 2H), 6.51 (d, *J* = 9.4 Hz, 1H), 6.01 (dd, *J* = 9.3, 6.5 Hz, 1H), 5.81 (d, *J* = 10.1 Hz, 1H), 5.34 – 5.24 (m, 1H), 5.14 (d, *J* = 6.2 Hz, 1H), 4.28 (brs, 1H), 3.85 (s, 3H), 3.20 (ddd, *J* = 15.1, 9.9, 5.5 Hz, 1H), 2.94 (d, *J* = 3.1 Hz, 1H), 2.87 – 2.72 (m, 2H), 2.66 (s, 3H), 2.42 (s, 3H), 2.21 – 2.11 (m, 1H), 2.00 – 1.90 (m, 1H)； **^{13}C NMR** (100 MHz, $CDCl_3$) δ 146.00, 143.86, 143.37, 134.36, 131.94, 129.68, 128.99, 128.49, 127.37, 125.15, 123.56, 118.04, 112.30, 89.95, 65.77, 56.05, 46.67, 45.00, 37.17, 35.04, 33.65, 21.47; **HRMS ESI** Calcd for $C_{25}H_{27}NO_5SNa$ $[M+Na]^+$: 476.1502, Found: 476.1492; **IR (neat)**: 3391, 2922, 1506, 1337, 1268, 1160, 1089 cm^{-1}; **EI MS** m/z (%): 91 (79), 155 (34), 198 (24), 241 (100), 268 (20), 298 (36), 453 (12).

The Synthesis of (–)-Codeine

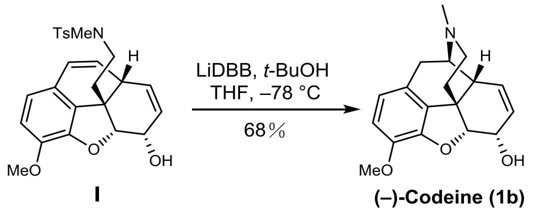

The LiDBB was freshly prepared following the Rychnovsky's protocol[14,15]: Under argon atmosphere, a oven-dried 10 mL Schlenk flask was charged with 4,4′-di-*tert*-butylbiphenyl (DBB, 266 mg, 1 mmol, 1 eq.) and dry THF (2 mL, freshly distilled from Na) at 0 ℃. Then metal lithium (70 mg, 10 mmol, 10 eq.) was **quickly** clipped into the flask under a stream of argon. The solution turned **dark green** and stirred vigorously under argon for 5 h at 0 ℃.

Under argon atmosphere, to a stirred solution of **I** (53 mg, 0.117 mmol, 1 eq.) and *t*-BuOH (87 mg, 1.17 mmol, 10 eq.) in dry THF (55 mL, 1 mg/mL) at −78 ℃ was added **dropwise** LiDBB (ca. 0.4 M in THF) via syringe until the color of the solution was markedly changed from colorless to dark green and **maintained for about 10 s**. Then the reaction mixture was stirred at −78 ℃ for 10 min and quenched with a pre-mixed saturated NH_4Cl solution and methanol (v:v = 1:1, 2 mL). The resulting mixture (PH > 10) was extracted with DCM. The combined organic extract was washed with brine, dried with Na_2SO_4, and concentrated *in vacuum*. The crude product was purified by column chromatography on silica gel (DCM : MeOH: NH_4OH = 10: 1: 0.01) to give product **(–)-codeine (1a)** (23.9 mg, 68.3% yield) as a white amorphous solid.

Compound **(–)-codeine**: $[\alpha]_D^{24}$ = −150 (c = 0.10, EtOH); Lit[16,17]: $[\alpha]_D$ = −133 (c = 0.23, EtOH); **^{1}H NMR** (600 MHz, $CDCl_3$) δ 6.66 (d, *J* = 8.2 Hz, 1H), 6.57 (d, *J* = 8.2 Hz, 1H), 5.71 (d, *J* = 9.9 Hz, 1H), 5.30 (d, *J* = 9.9 Hz, 1H), 4.90 (dd, *J* = 6.5, 0.9 Hz, 1H), 4.22 – 4.13 (m, 1H), 3.85 (s, 3H), 3.35 (dd, *J* = 5.9, 3.1 Hz, 1H), 3.05 (d, *J* = 18.6 Hz, 1H), 2.70 – 2.65 (m, 1H), 2.59 (dd, *J* = 12.2, 4.3 Hz, 1H), 2.44 (s, 3H), 2.40 (td, *J* = 12.3, 3.5 Hz, 1H), 2.30 (dd, *J* = 18.6, 6.2 Hz, 1H), 2.07 (dt, *J* = 12.4, 4.9 Hz, 1H), 1.88 (dd, *J* = 12.6, 1.3 Hz, 1H); **^{13}C NMR** (150 MHz, $CDCl_3$) δ 146.32, 142.20, 133.41, 131.08, 128.26, 127.24, 119.53, 112.92, 91.35, 66.41, 58.89, 56.35, 46.45, 43.11, 42.96, 40.80, 35.83, 20.41; **HRMS ESI** Calcd for $C_{18}H_{22}NO_3$ $[M+H]^+$: 300.1594, Found: 300.1585; **IR (neat)**: 3397, 2921, 2102, 1644, 1504, 1455, 1274, 1047, 737 cm^{-1}; **EI MS** m/z (%): 151 (61), 152 (21), 162 (33), 214 (24), 229 (25), 242 (9), 280 (13), 299 (100).

Note: In order to avoid the deviation of chemical shift caused by a little acid which might be introduced during purification by silica gel column chromatography, **^{1}H NMR** and **^{13}C NMR** spectra of our synthetic (–)-codeine

were obtained by carrying out the **Titration NMR Experiment** by using about 0.5 equiv of 28% NH_4OH (Figure 4).

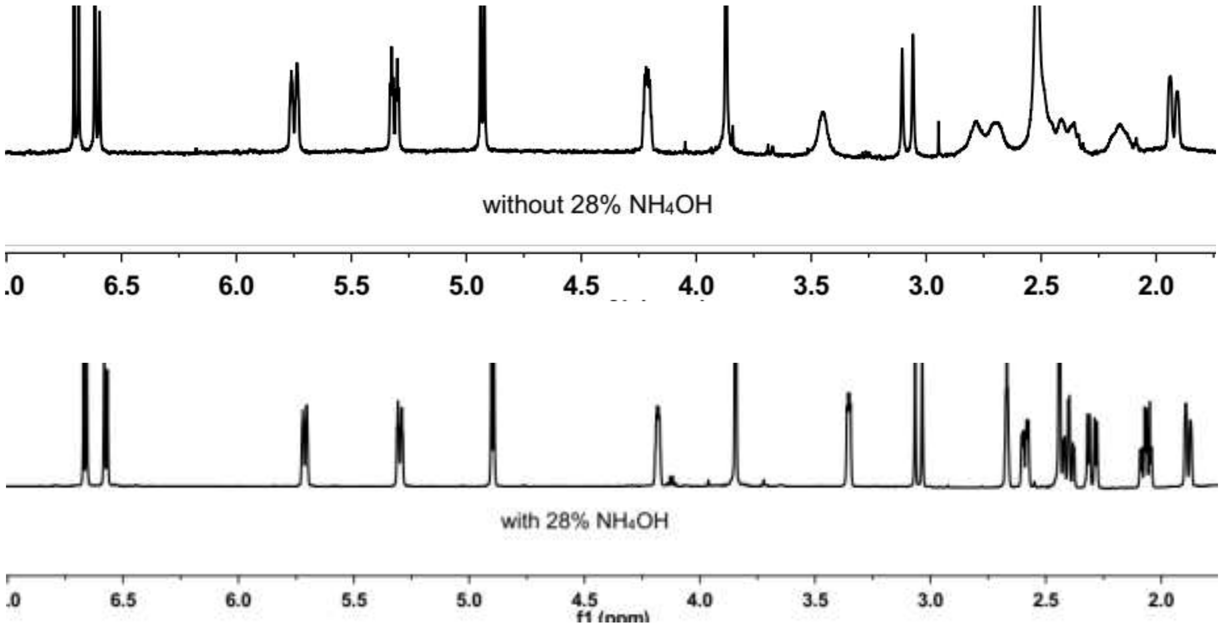

Supplementary Figure 4. **Comparison of ^{1}H NMR Spectra of (–)-Codeine (600 MHz, $CDCl_3$)**

Comparison of Hydroamination Reaction with the Previously Reported Approaches

Supplementary Table 8. **Comparison of Reaction Result**

**Author(Year)**[ref]	**Approach**	**Scale**	**Yield**
**This work**	**LiDBB in THF**	**53 mg**	**68%**
Zhang (2015)[18]	Li/NH_3(l) in THF	10 mg	60%
Guillou (2008)[19]	Li/NH_3(l) in THF	6 mg	51%
Hudlicky(2007, 2009)[20,21]	$Hg(OAc)_2$ in THF then $LiAlH_4$	50 mg	17.6%
Trost(2002, 2005)[16,17]	LDA, tungsten bulb	7 mg	57%

Result: This methodology (LiDBB) showed a remarkable superiority in terms of the reproducibility (for the more details, please see the ref.13), synthetic scale (53 mg scale *vs* <10 mg scale), and the chemical yield (the more accurate and higher yield).

The Synthesis of (–)-Morphine

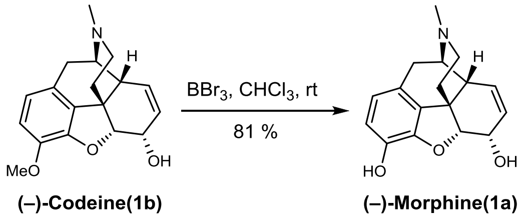

To a stirred solution of **(–)-codeine** (10 mg, 0.0334 mmol, 1eq.) in dry $CHCl_3$ (1.0 mL) at room temperature was added boron tribromide (200 uL, 0.2 mmol, 1 M in DCM, 6 eq.). After stirred for 20 min, the reaction was quenched at 0 ℃ with 10% aq. ammonium hydroxide (1 mL). The resulting mixture (PH > 7) was extracted with 10% ethanol in DCM. The combined organic layer was washed with brine, dried with Na_2SO_4, and concentrated *in vacuum*. The crude product was purified by preparative thin layer chromatography (DCM: MeOH = 5 : 1) to afford **(–)-morphine** (7.7 mg, 81%) as white powder.

Compound **(–)-morphine**: $[\alpha]_D^{27}$ = −114 (c = 0.07, EtOH); Lit[22]: $[\alpha]_D^{24}$ = −132 (c = 0.10, EtOH); **^{1}H NMR** (600 MHz, $CDCl_3$) δ 6.63 (d, *J* = 8.1 Hz, 1H), 6.50 (d, *J* = 8.2 Hz, 1H), 5.66 (d, *J* = 9.6 Hz, 1H), 5.27 (d, *J* = 9.9 Hz, 1H), 4.89 (d, *J* = 6.4 Hz, 1H), 4.20 (d, *J* = 3.3 Hz, 1H), 3.42 (s, 1H), 3.03 (d, *J* = 18.6 Hz, 1H), 2.74 (s, 1H), 2.67 (d, *J* = 9.2 Hz, 1H), 2.47 (s, 4H), 2.34 (dd, *J* = 18.6, 6.0 Hz, 1H), 2.12 (td, *J* = 12.2, 4.1 Hz, 1H), 1.88 (d, *J* = 11.7 Hz, 1H); **^{13}C NMR** (100 MHz, $CDCl_3$) δ 145.46, 138.18, 133.02, 130.70, 128.22, 126.08, 119.95, 117.03, 91.50, 66.53, 58.99, 46.49, 43.02, 42.89, 40.44, 35.32, 20.60; **HRMS ESI** Calcd for $C_{17}H_{20}NO_3$ $[M+H]^+$: 286.1438, Found: 286.1438; **IR (neat)**: 3373, 2925, 2374, 1610, 1459, 1250, 1195, 1032, 908, 734 cm^{-1}; **EI MS** m/z (%): 115 (25), 124 (19), 162 (27), 174 (10), 200 (7), 215 (17), 256 (4), 268 (10), 285 (100); **Mp**: 210-213 ℃ (decomp.).

For ¹H NMR Comparison of Our Synthetic (–)-Codeine with the Previously Reported (±)-Codeine, see the Following Table 9.

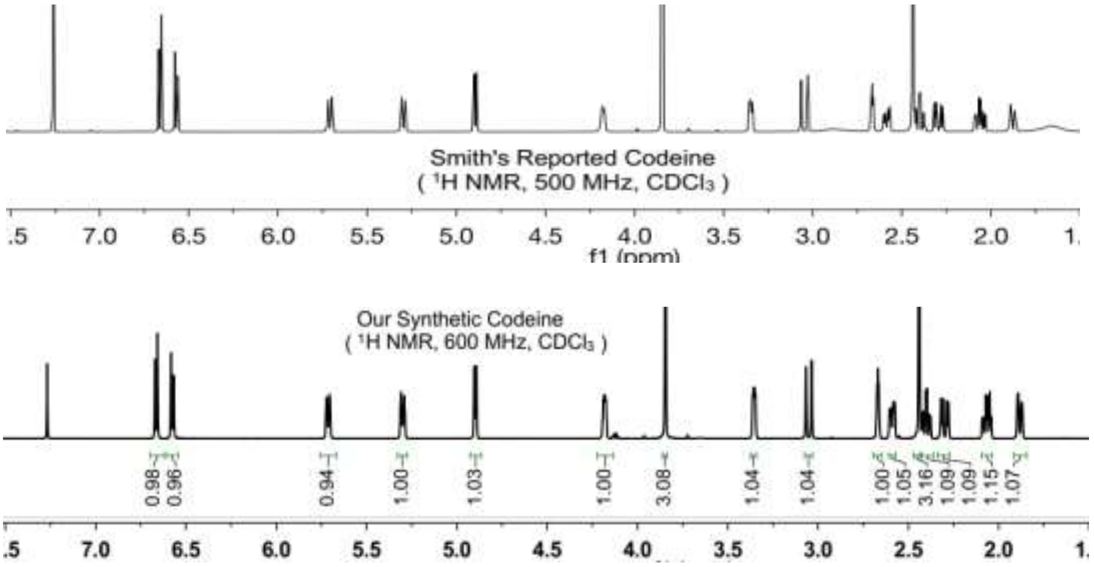

Supplementary Table 9. **Comparison of ¹H NMR Spectral Data of Codeine**

A: Martin D. Smith's Report[23]	B: Our Synthetic One	Error (B–A)
δ (ppm, 500 MHz, $CDCl_3$)	δ (ppm, 600 MHz, $CDCl_3$)	Δδ/ppm
6.66 (d, *J* = 8.2 Hz, 1H)	6.66 (d, *J* = 8.2 Hz, 1H)	0
6.57 (d, *J* = 8.2 Hz, 1H)	6.57 (d, *J* = 8.2 Hz, 1H)	0
5.71 (dddd, *J* = 9.9, 3.2, 1.9, 1.3 Hz, 1H)	5.71 (d, *J* = 9.9 Hz, 1H)	0
5.30 (ddd, *J* = 9.9, 3.2, 2.6 Hz, 1H)	5.30 (d, *J* = 9.9 Hz, 1H)	0
4.89 (dd, *J* = 6.5, 1.3 Hz, 1H)	4.90 (dd, *J* = 6.5, 0.9 Hz, 1H)	0.01
4.18 (ddd, *J* = 6.5, 3.2, 2.6 Hz, 1H)	4.22 – 4.13 (m, 1H)	-
3.84 (s, 3H)	3.85 (s, 3H)	0.01
3.35 (dd, *J* = 6.2, 3.2 Hz, 1H)	3.35 (dd, *J* = 5.9, 3.1 Hz, 1H)	0
3.05 (d, *J* = 18.6 Hz, 1H)	3.05 (d, *J* = 18.6 Hz, 1H)	0.01
2.67 (ddd, *J* = 3.2, 3.2, 1.9 Hz, 1H)	2.70 – 2.65 (m, 1H)	-
2.59 (dd, *J* = 12.5, 5.3 Hz, 1H)	2.59 (dd, *J* = 12.2, 4.3 Hz, 1H)	0
2.44 (s, 3H)	2.44 (s, 3H)	0
2.40 (td, *J* = 12.5, 3.6 Hz, 1H)	2.40 (td, *J* = 12.3, 3.5 Hz, 1H)	0
2.29 (dd, *J* = 18.6, 6.2 Hz, 1H)	2.30 (dd, *J* = 18.6, 6.2 Hz, 1H)	0.01
2.06 (td, *J* = 12.5, 5.3 Hz, 1H)	2.07 (dt, *J* = 12.4, 4.9 Hz, 1H)	0.01
1.88 (ddd, *J* = 12.5, 3.6, 1.7 Hz, 1H)	1.88 (dd, *J* = 12.6, 1.3 Hz, 1H)	0

For ^{13}C NMR Comparison of Our Synthetic (–)-Codeine with the Previously Reported (±)-Codeine, see the Following Table 10.

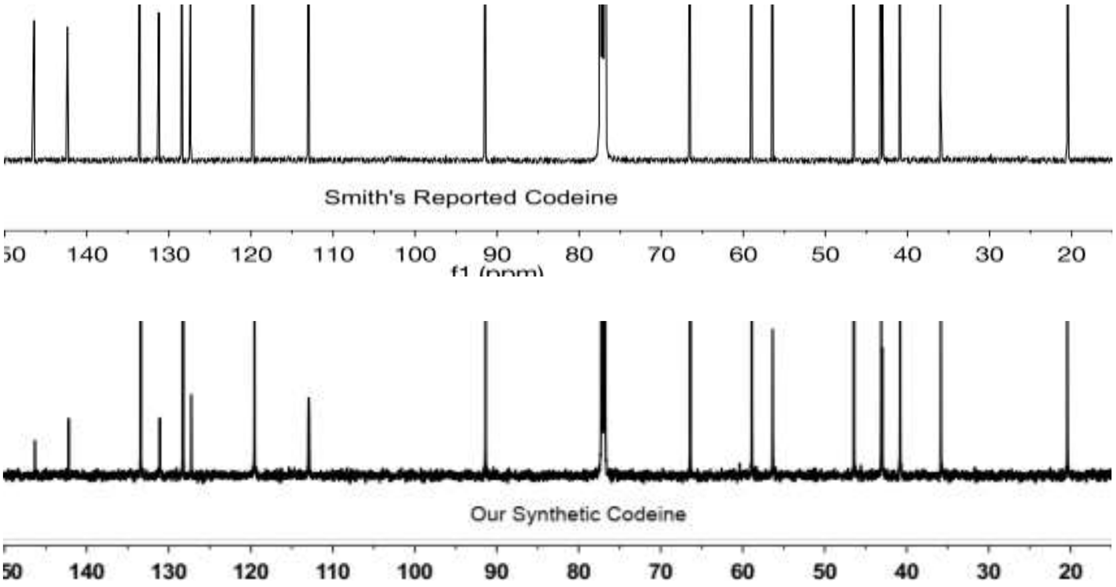

Supplementary Table 10. **Comparison of ^{13}C NMR Spectral Data of Codeine**

A: Martin D. Smith's Report[23]	B: Our Synthetic One	Error (A–B)
δ (ppm, 125 MHz, $CDCl_3$)	δ (ppm, 150 MHz, $CDCl_3$)	Δδ/ppm
146.4	146.3	0.1
142.4	142.2	0.2
133.6	133.4	0.2
131.2	131.1	0.1
128.4	128.3	0.1
127.4	127.2	0.2
119.7	119.5	0.2
113.0	112.9	0.1
91.5	91.4	0.1
66.5	66.4	0.1
59.0	58.9	0.1
56.5	56.4	0.1
46.6	46.5	0.1
43.3	43.1	0.2
43.1	43.0	0.1
41.0	40.8	0.2
36	35.8	0.2
20.5	20.4	0.1

For ^{1}H NMR Comparison of Our Synthetic (–)-Morphine with the Previously Reported (±)-Morphine, see the Following Table 11.

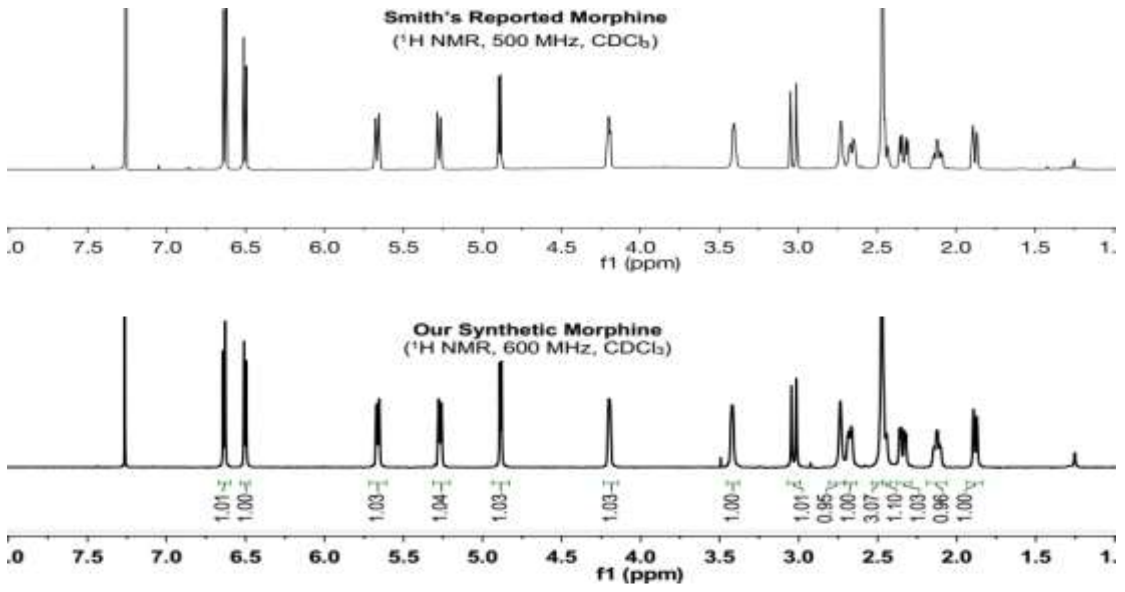

Supplementary Table 11. Comparison of ^{1}H NMR Spectral Data of Morphine

A: Martin D. Smith's Report[23]	B: Our Synthetic One	Error (B–A)
δ (ppm, 500 MHz,$CDCl_3$)	δ (ppm, 600 MHz,$CDCl_3$)	Δδ/ppm
6.63 (d, J = 8.1 Hz, 1H)	6.63 (d, J = 8.1 Hz, 1H)	0
6.50 (d, J = 8.1 Hz, 1H)	6.50 (d, J = 8.2 Hz, 1H)	0
5.67 (dddd, J = 9.9, 3.1,1.6, 1.1 Hz, 1H)	5.66 (d, J = 9.6 Hz, 1H)	-0.01
5.28 (ddd, J = 9.9, 3.3, 1.9 Hz, 1H)	5.27 (d, J = 9.9 Hz, 1H)	-0.01
4.89 (dd, J = 6.5, 1.1 Hz, 1H)	4.89 (d, J = 6.4 Hz, 1H)	0
4.20 (ddd, J = 6.5, 3.1, 1.9 Hz, 1H)	4.20 (d, J = 3.3 Hz, 1H)	0
3.41 (dd, J = 6.4, 3.2 Hz, 1H)	3.42 (s, 1H)	0.01
3.03 (d, J = 18.7 Hz, 1H)	3.03 (d, J = 18.6 Hz, 1H)	0
2.73 (br, 1H)	2.74 (s, 1H)	0.01
2.66 (dd,J = 12.5, 4.7 Hz, 1H)	2.68 (d, J = 9.9 Hz, 1H)	0.02
2.47 (s, 3H)	2.47 (s, 3H)	0
2.45 (td, J = 12.5, 3.4 Hz, 1H)	2.44 (s, 1H)	-0.01
2.33 (dd, J = 18.7, 6.4 Hz, 1H)	2.34 (dd, J = 18.6, 6.0 Hz, 1H)	0.01
2.12 (td, J = 12.5, 4.7 Hz, 1H)	2.12 (td, J = 12.2, 4.1 Hz, 1H)	0
1.88 (ddd, J = 12.5, 3.4, 1.7 Hz, 1H)	1.88 (d, J = 11.7 Hz, 1H)	0

For ^{13}C NMR Comparison of Our Synthetic (–)-Morphine with the Previously Reported (±)-Morphine, see the Following Table 12.

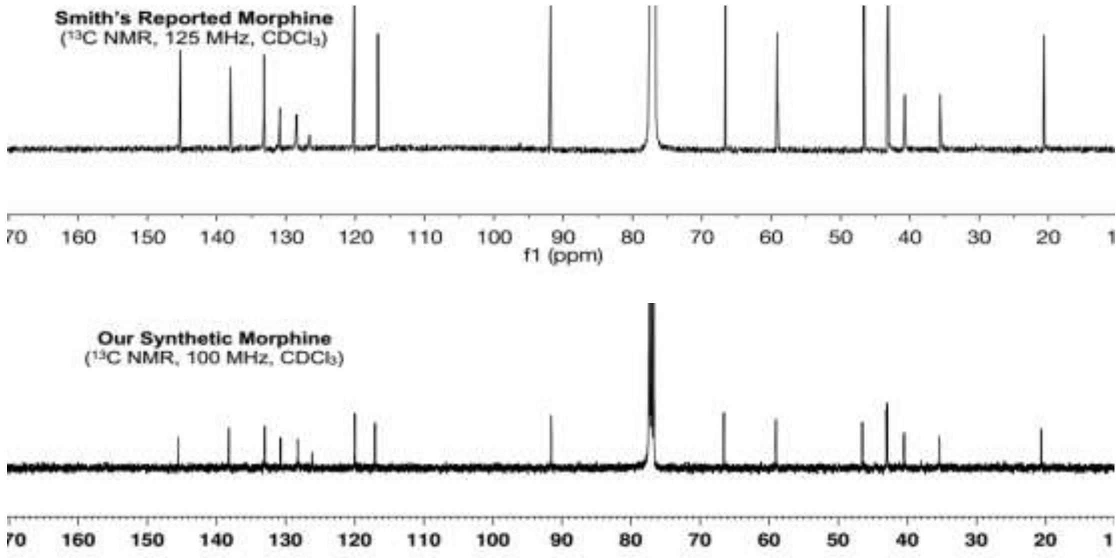

Supplementary Table 12. **Comparison of ^{13}C NMR Spectral Data of Morphine**

A: Martin D. Smith's Report[23]	B: Our Synthetic One	Error (B–A)
δ (ppm, 125 MHz,$CDCl_3$)	δ (ppm, 100 MHz,$CDCl_3$)	Δδ/ppm
145.2	145.5	0.3
138.0	138.2	0.2
133.2	133.0	-0.2
130.9	130.7	-0.2
128.5	128.2	-0.3
126.6	126.1	-0.5
120.2	120.0	-0.2
116.8	117	0.2
91.9	91.5	-0.4
66.6	66.5	-0.1
59.1	59.0	-0.1
46.6	46.5	-0.1
43.3	43.0	-0.3
43.1	42.9	-0.2
40.7	40.4	-0.3
35.6	35.3	-0.3
20.7	20.6	-0.1

X-Ray Crystallographic Data

The crystal structure and X-ray crystallographic data of compound **10** (CCDC **1882059**) was described as follows:

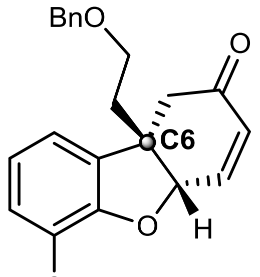

10

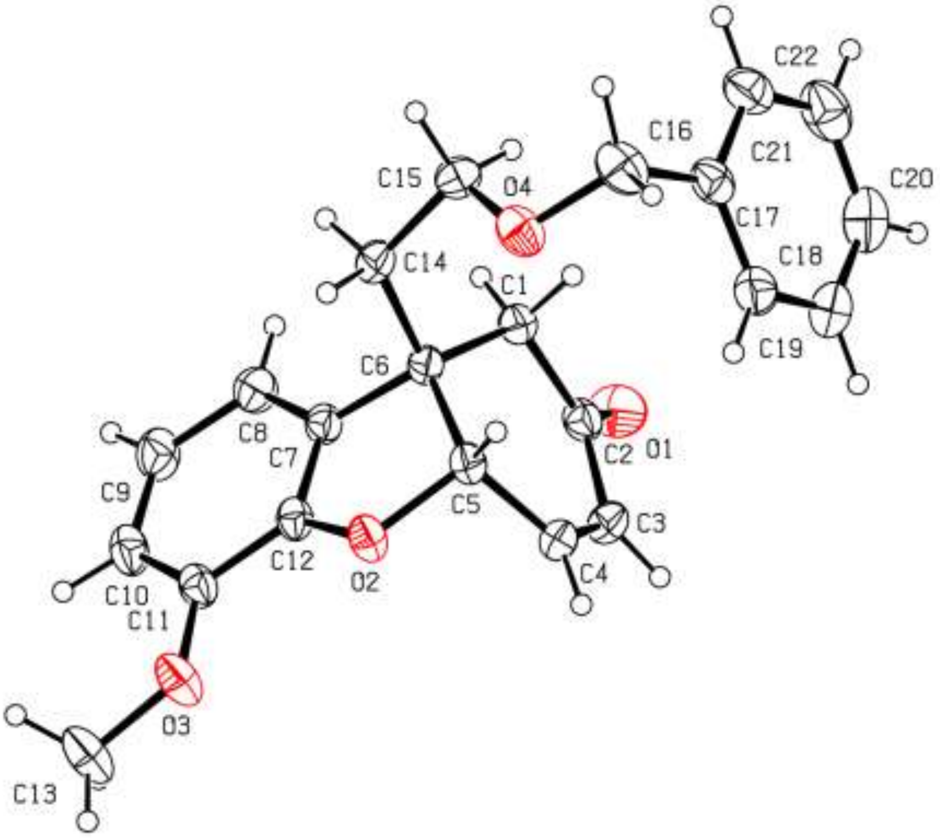

Supplementary Figure 5. X-ray crystal structure of **10**

Supplementary Table 13 Crystal data and structure refinement for compound 10.	
Identification code	zhangq_0724

Empirical formula	$C_{22}H_{22}O_4$
Formula weight	350.39
Temperature/K	262(13)
Crystal system	orthorhombic
Space group	$P2_12_12_1$
a/Å	8.7666(3)
b/Å	8.7971(2)
c/Å	23.6955(6)
α/°	90
β/°	90
γ/°	90
Volume/$Å^3$	1827.42(9)
Z	4
$\rho_{calc}g/cm^3$	1.274
μ/mm^{-1}	0.702
F(000)	744.0
Crystal size/mm^3	0.18 × 0.15 × 0.12
Radiation	CuKα (λ = 1.54184)
2Θ range for data collection/°	7.462 to 133.184
Index ranges	$-9 \leq h \leq 10$, $-10 \leq k \leq 10$, $-27 \leq l \leq 28$
Reflections collected	8612
Independent reflections	3152 [R_{int} = 0.0254, R_{sigma} = 0.0266]
Data/restraints/parameters	3152/12/236
Goodness-of-fit on F^2	1.060
Final R indexes [I>=2σ (I)]	R_1 = 0.0333, wR_2 = 0.0839
Final R indexes [all data]	R_1 = 0.0352, wR_2 = 0.0855
Largest diff. peak/hole / e $Å^{-3}$	0.12/-0.15
Flack parameter	-0.05(10)

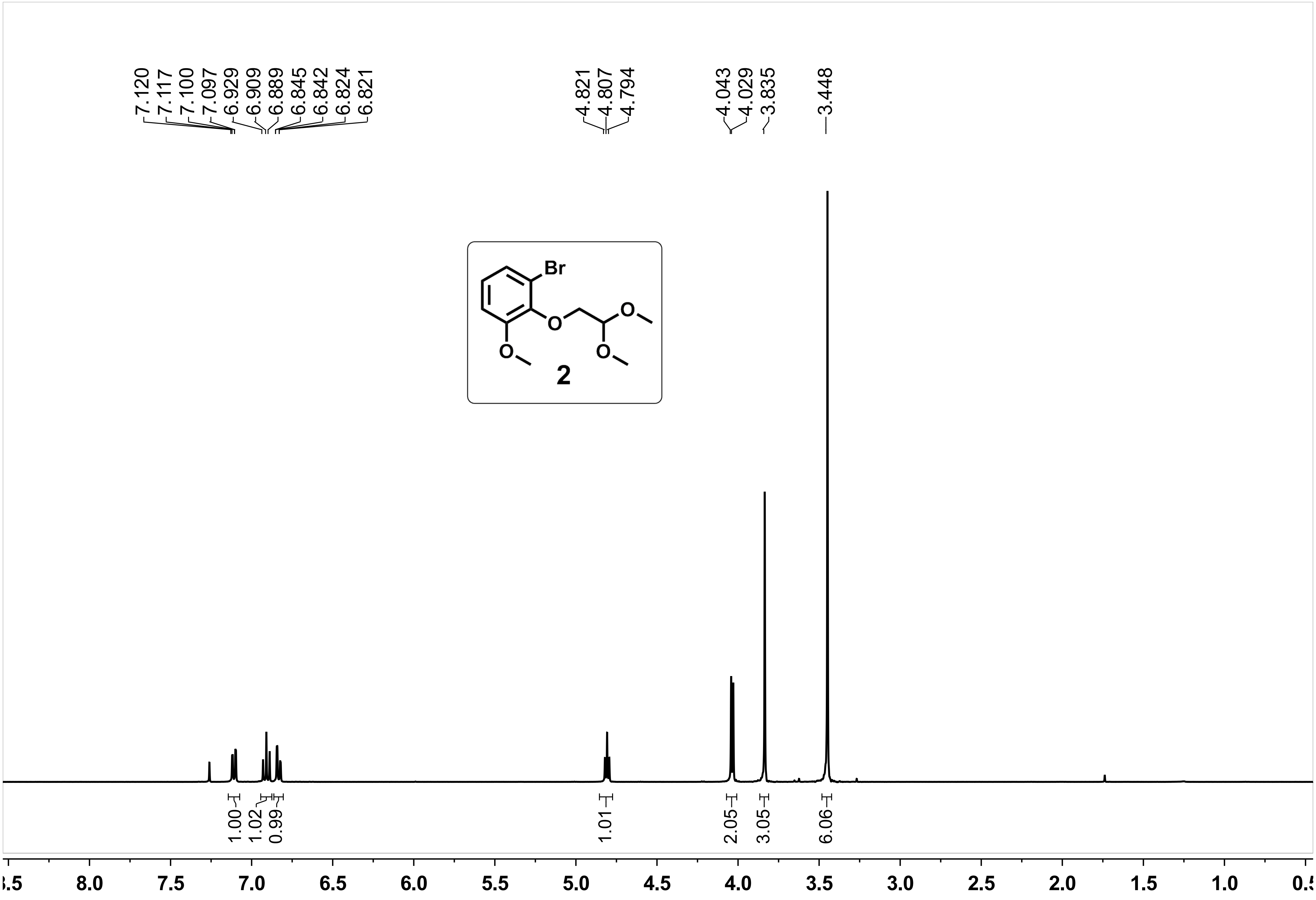

Supplementary Figure 6. ^{1}H NMR of **2**

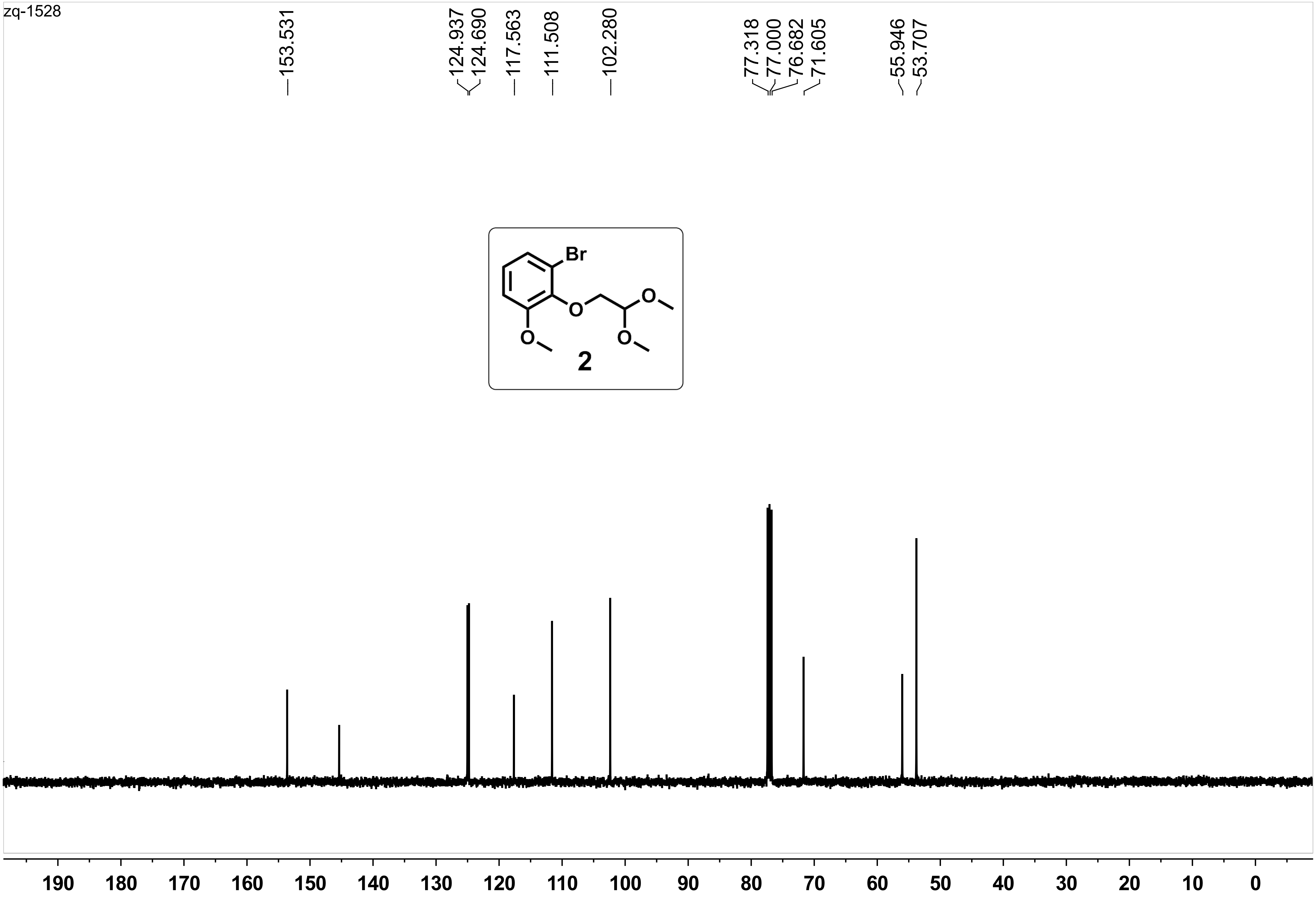

Supplementary Figure 7. ^{13}C NMR of **2**

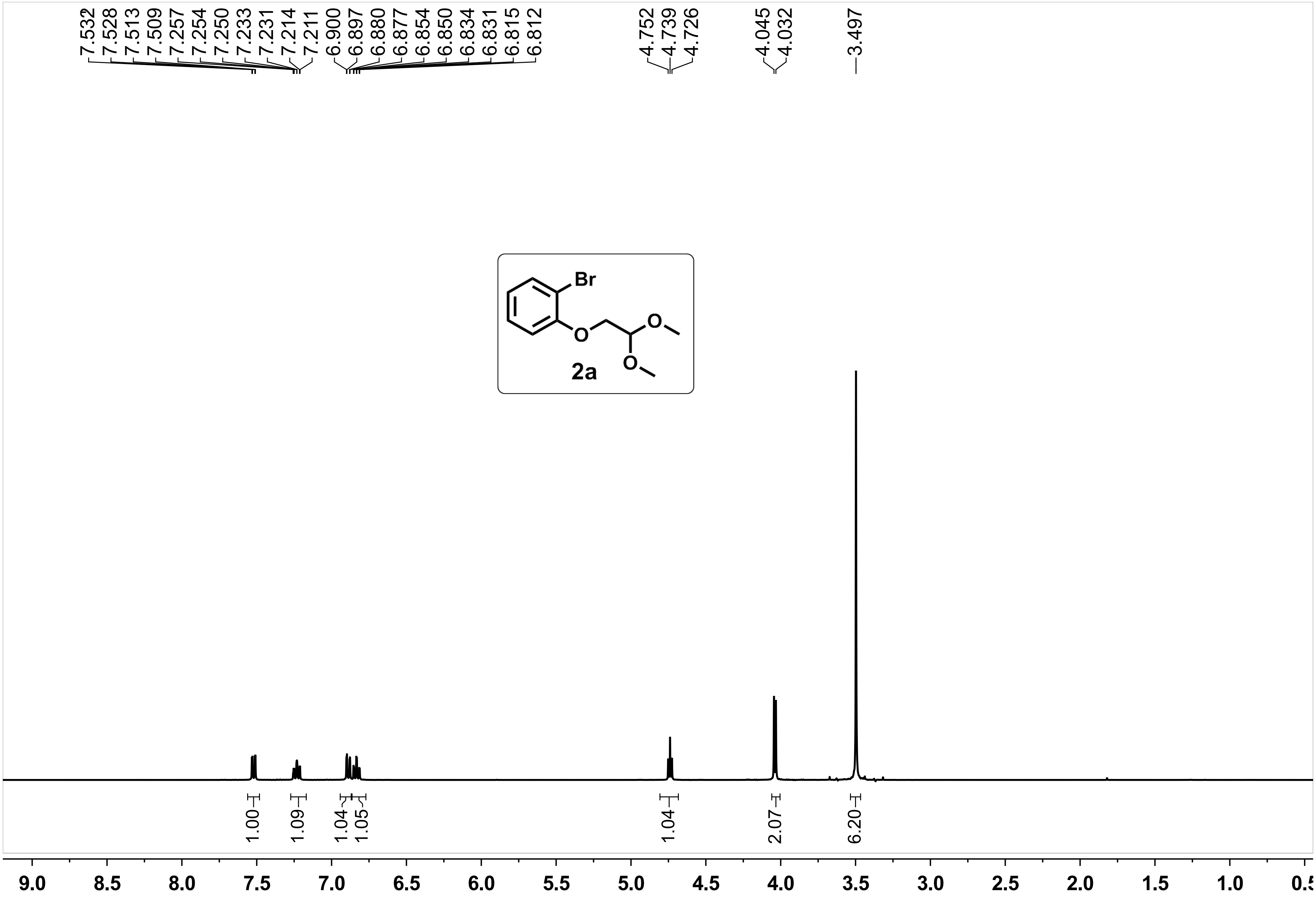

Supplementary Figure 8. ^{1}H NMR of **2a**

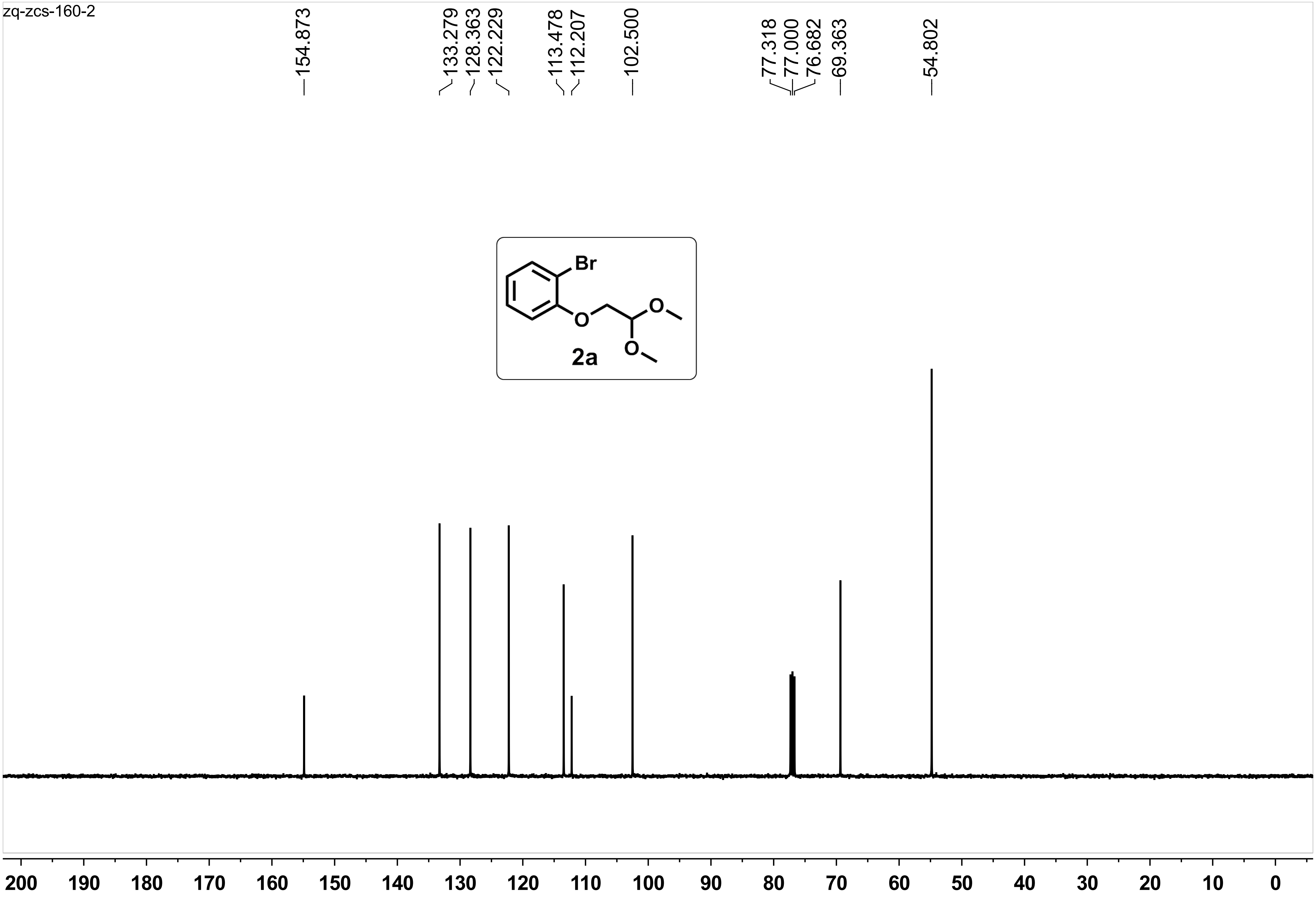

Supplementary Figure 9. ^{13}C NMR of **2a**

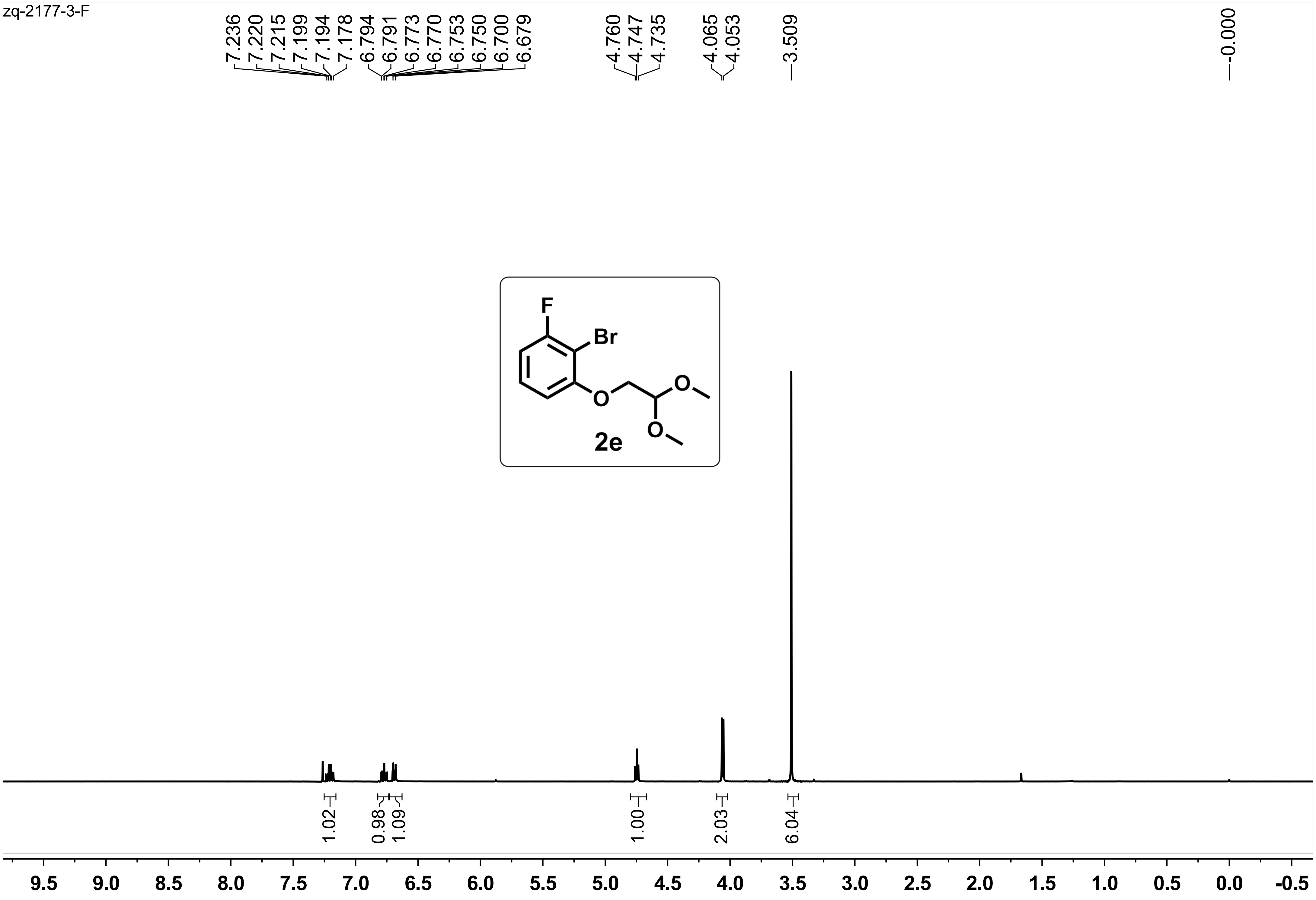

Supplementary Figure 10. ^{1}H NMR of **2e**

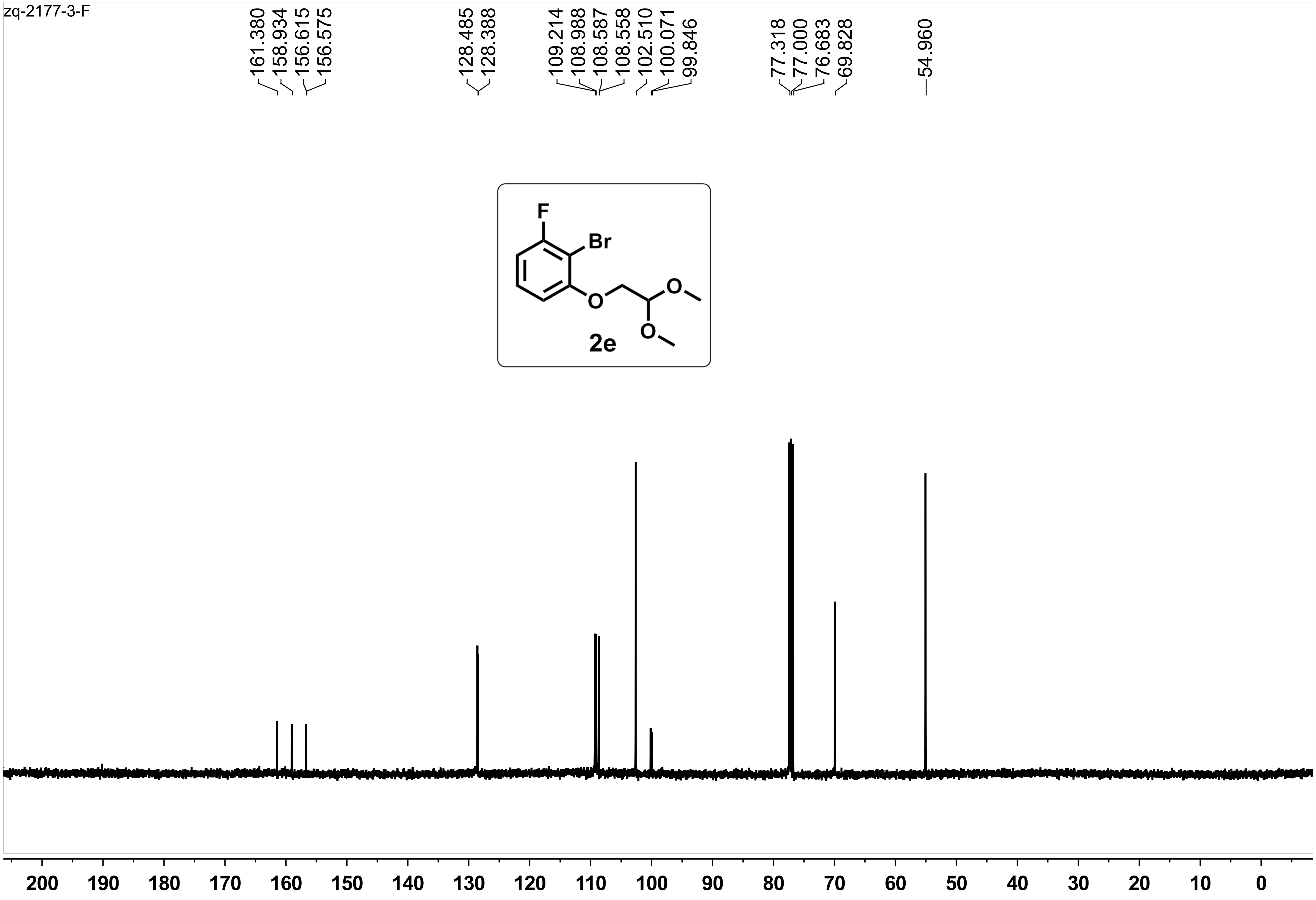

Supplementary Figure 11. ^{13}C NMR of **2e**

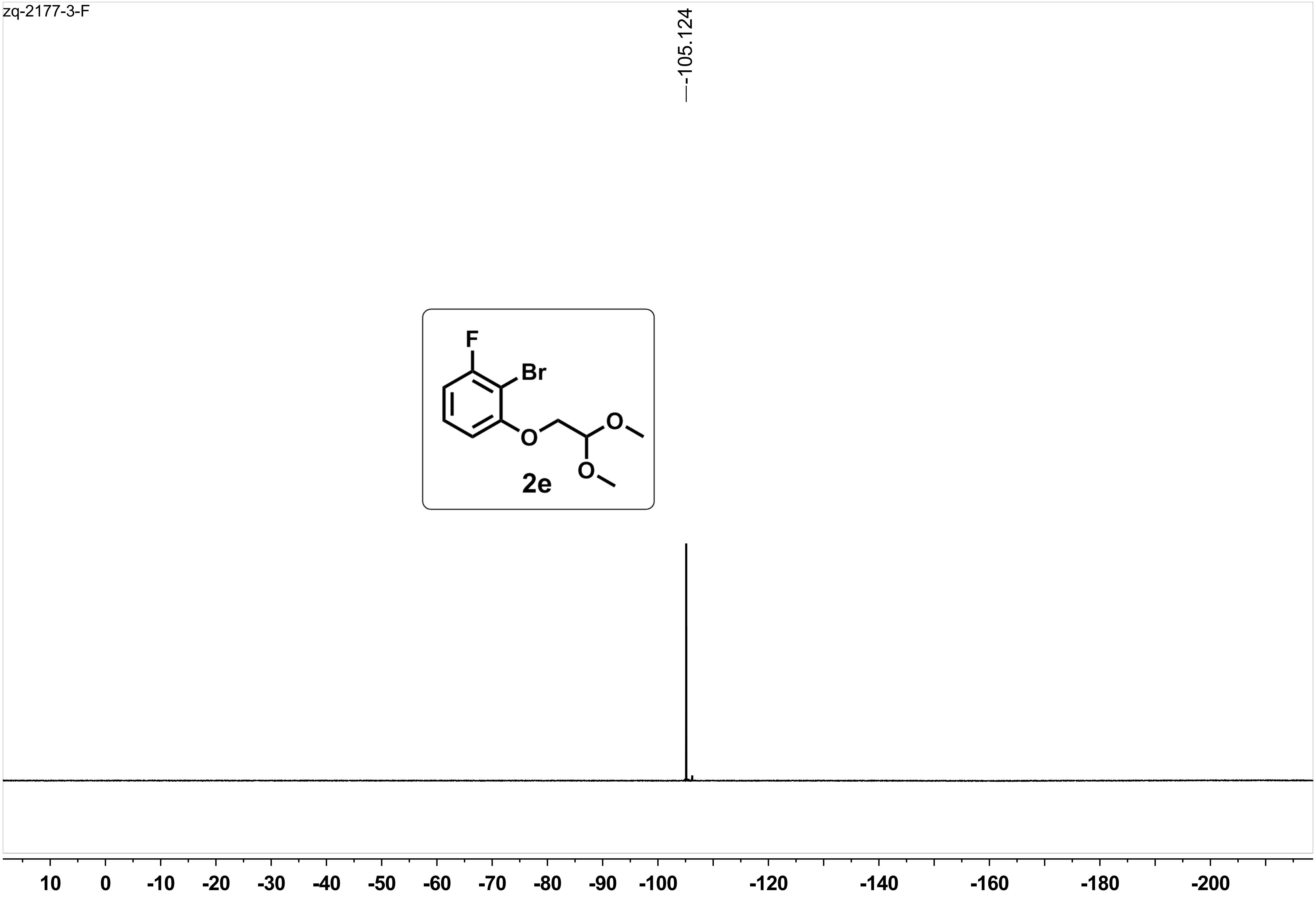

Supplementary Figure 12. ^{19}F NMR of **2e**

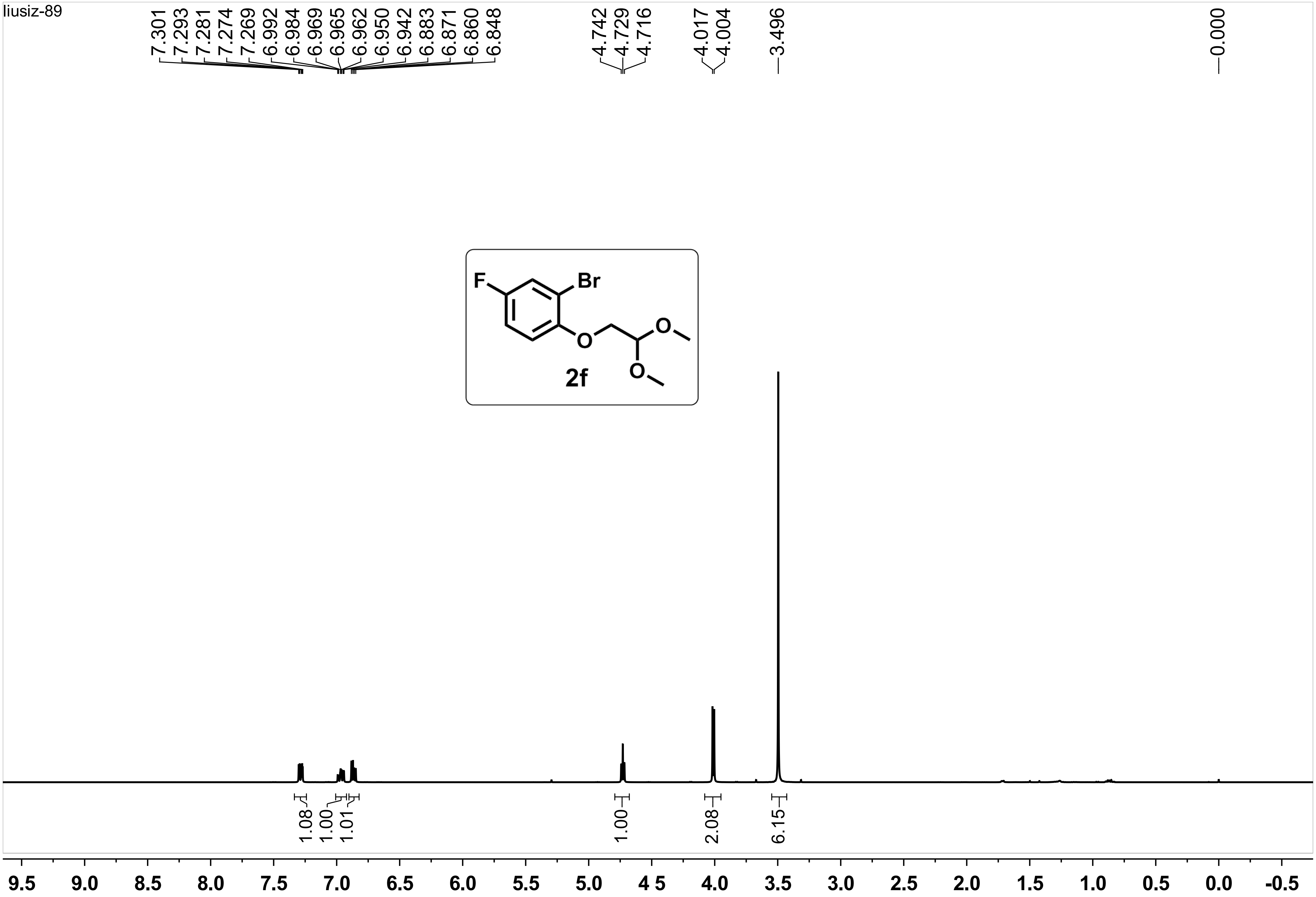

Supplementary Figure 13. ^{1}H NMR of **2f**

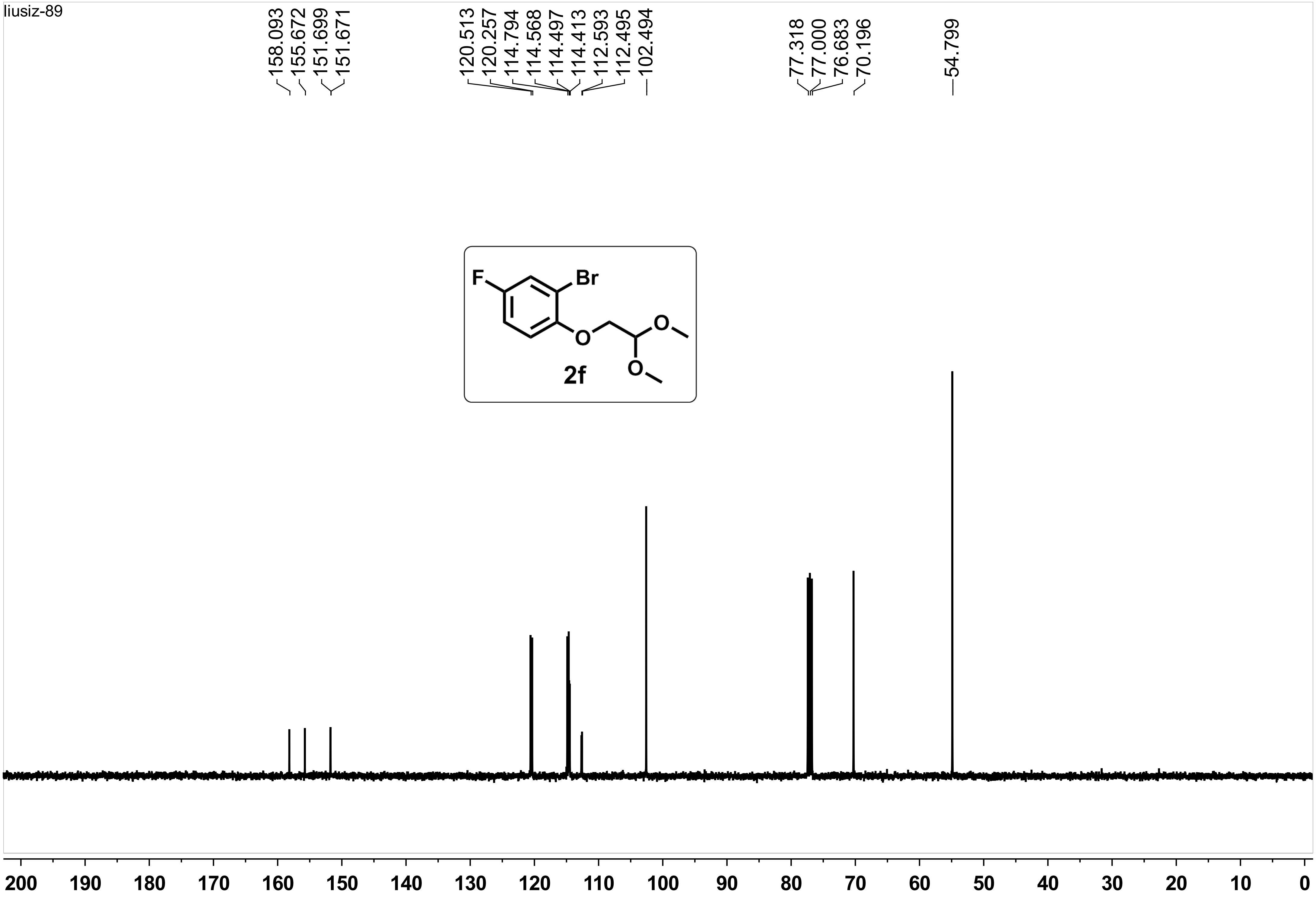

Supplementary Figure 14. ^{13}C NMR of **2f**

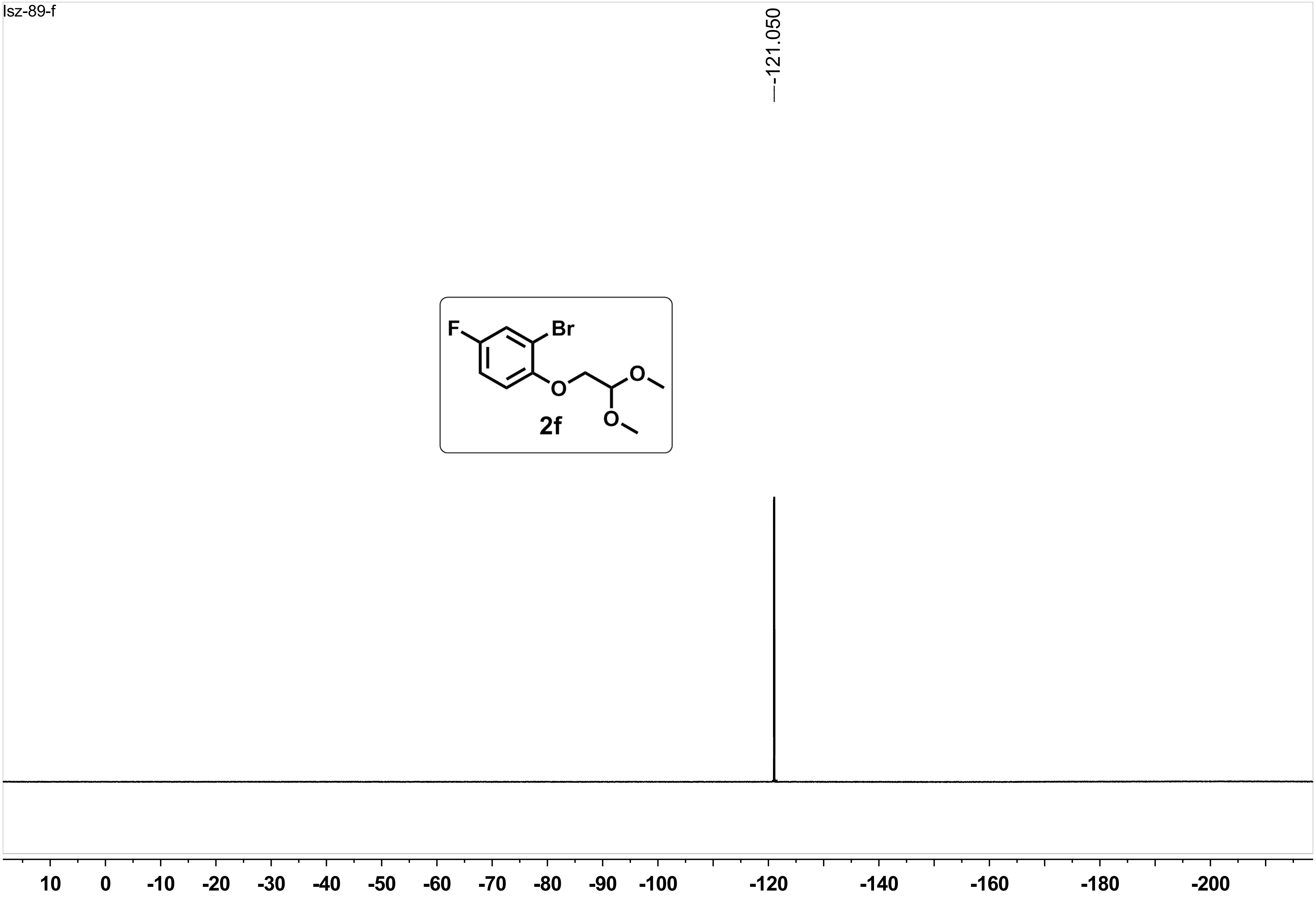

Supplementary Figure 15. ^{19}F NMR of **2f**

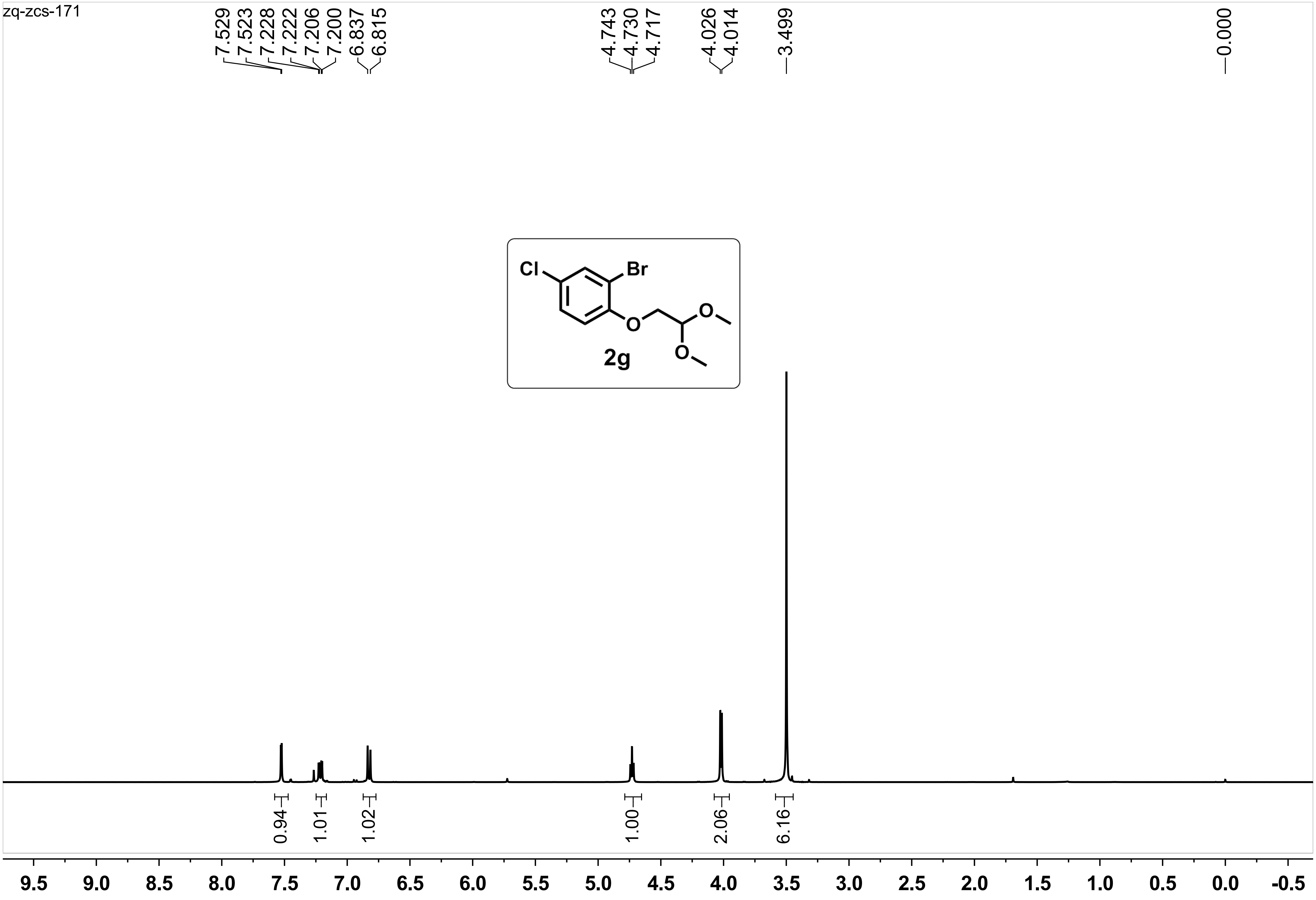

Supplementary Figure 16. ^{1}H NMR of **2g**

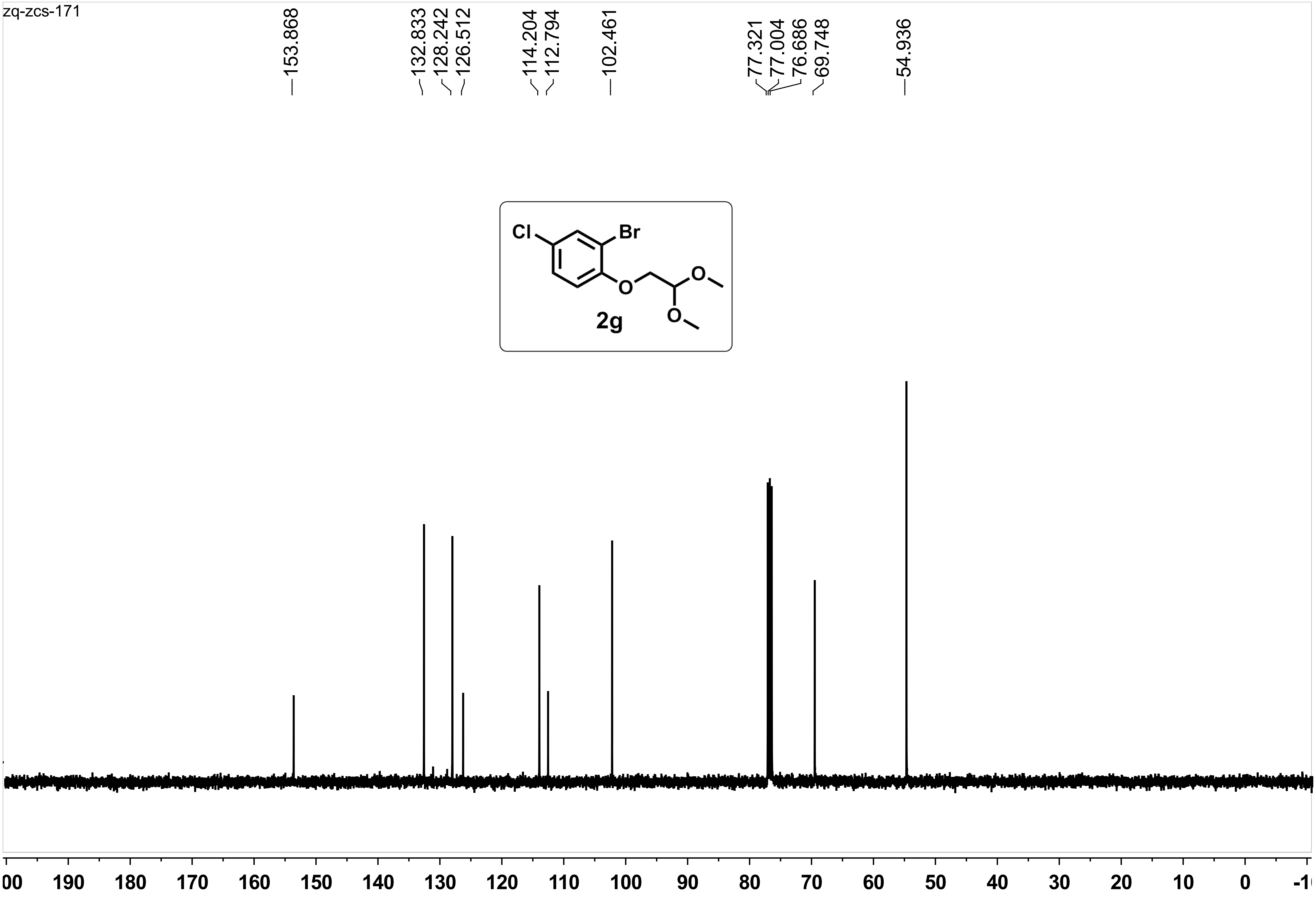

Supplementary Figure 17. ^{13}C NMR of **2g**

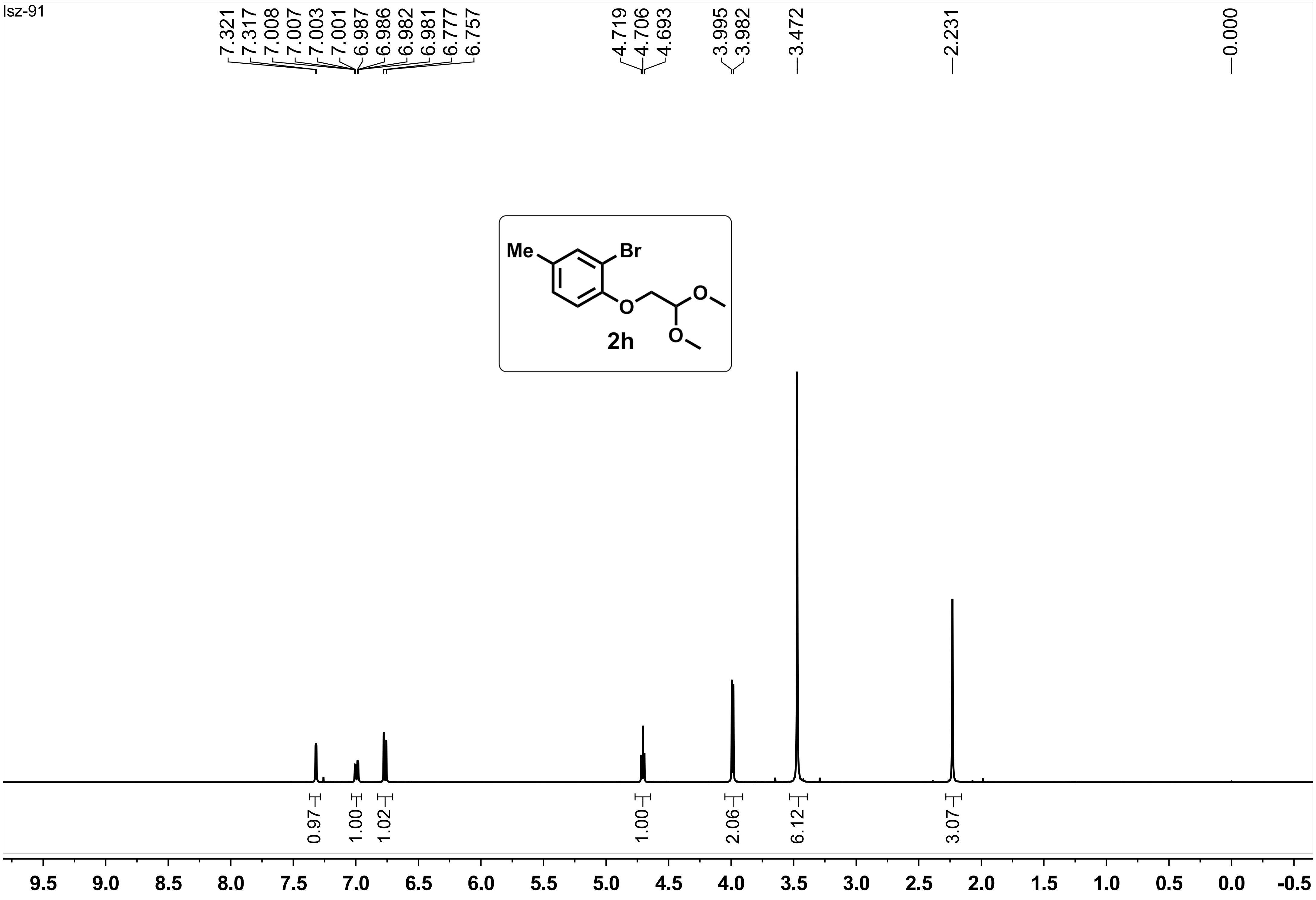

Supplementary Figure 18. ^{1}H NMR of **2h**

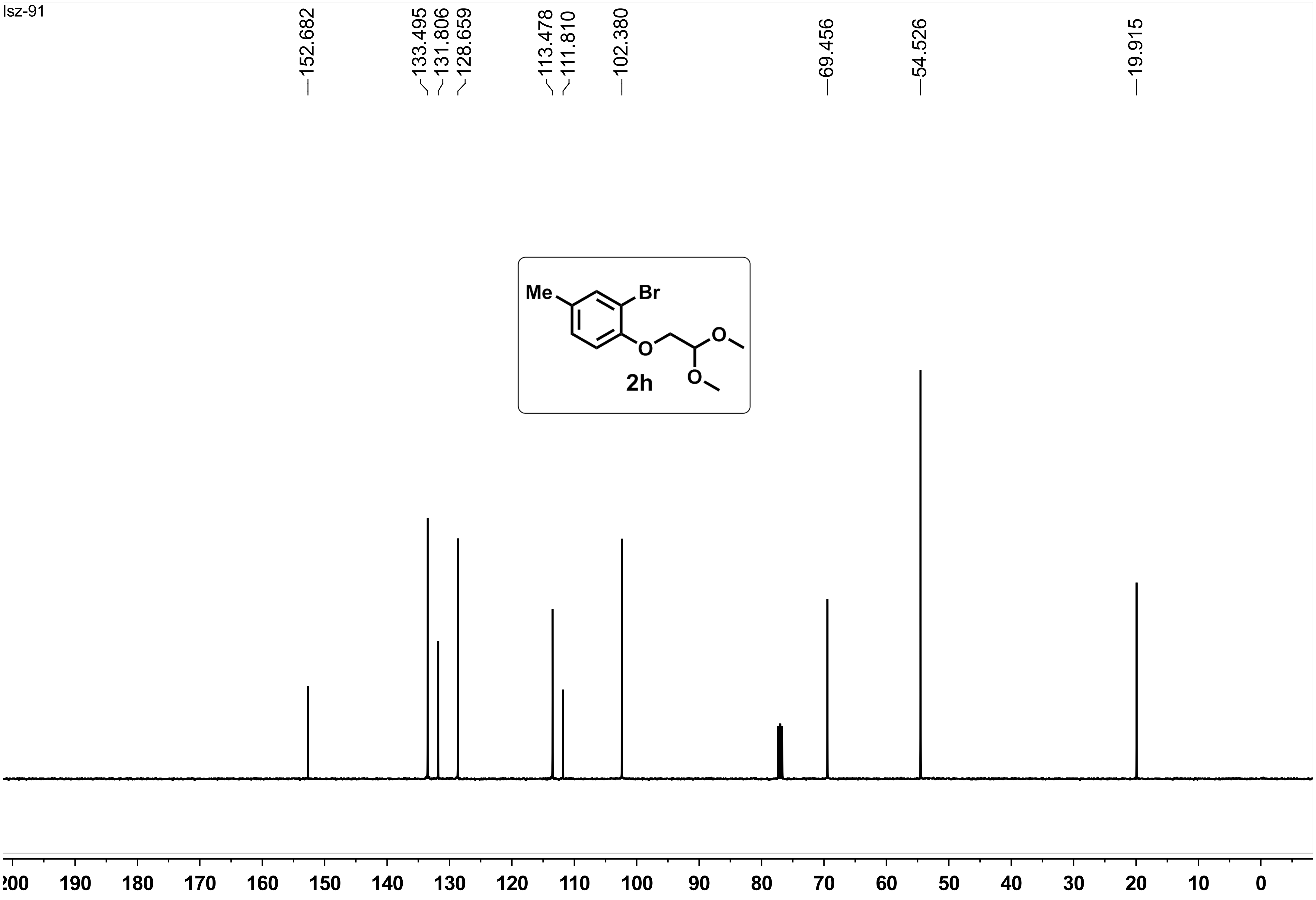

Supplementary Figure 19. ^{13}C NMR of **2h**

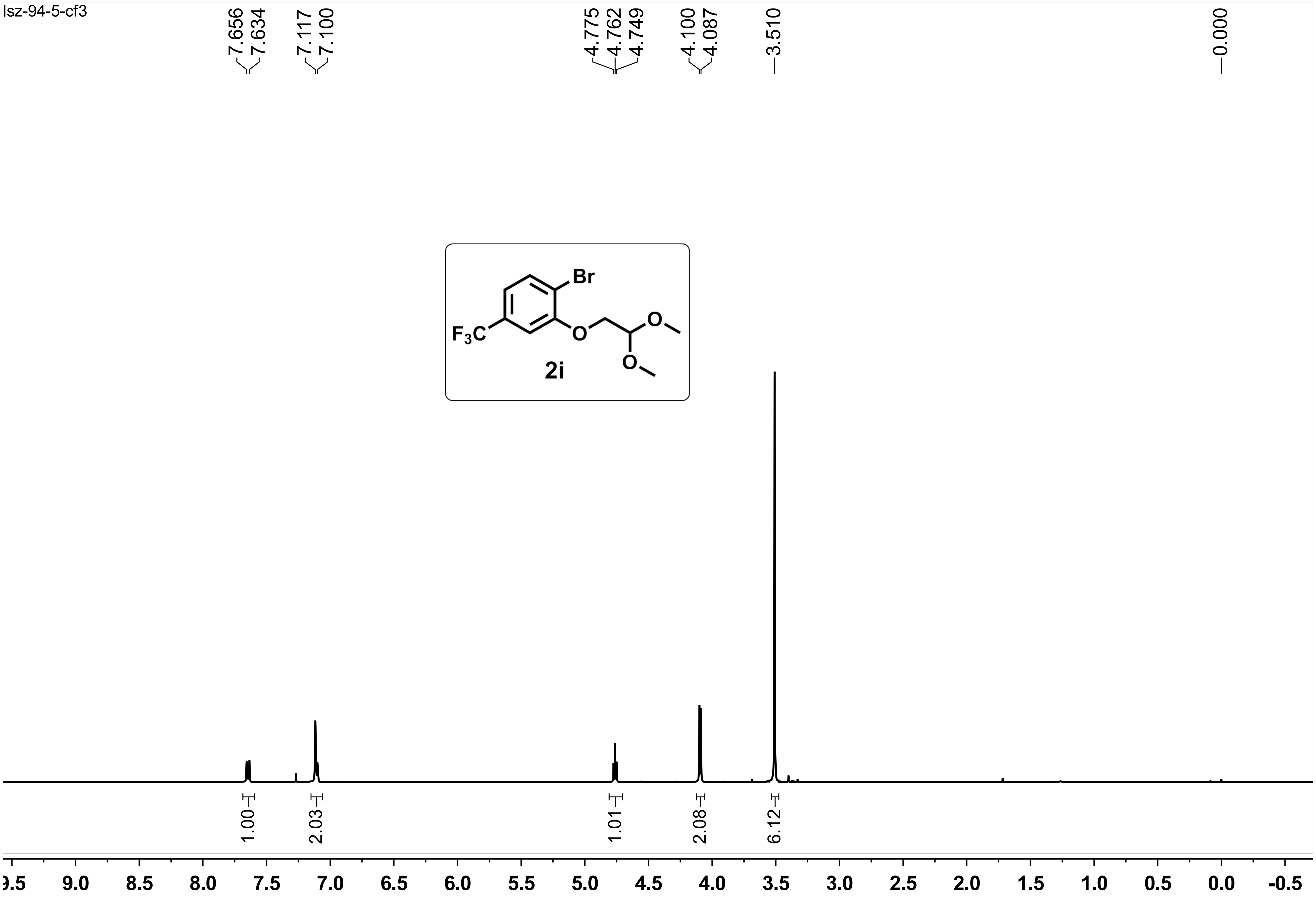

Supplementary Figure 20. ^{1}H NMR of **2i**

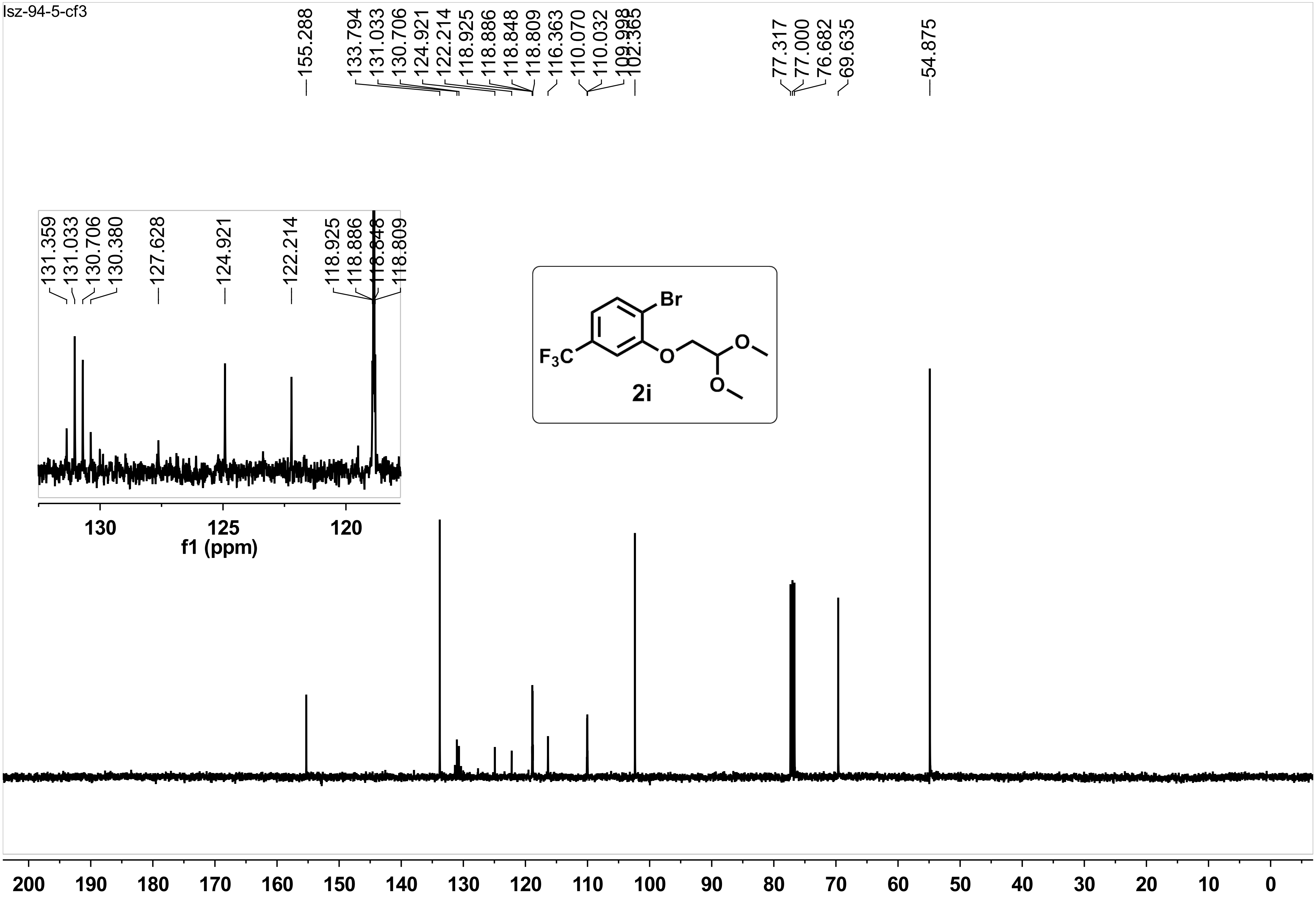

Supplementary Figure 21. ^{13}C NMR of **2i**

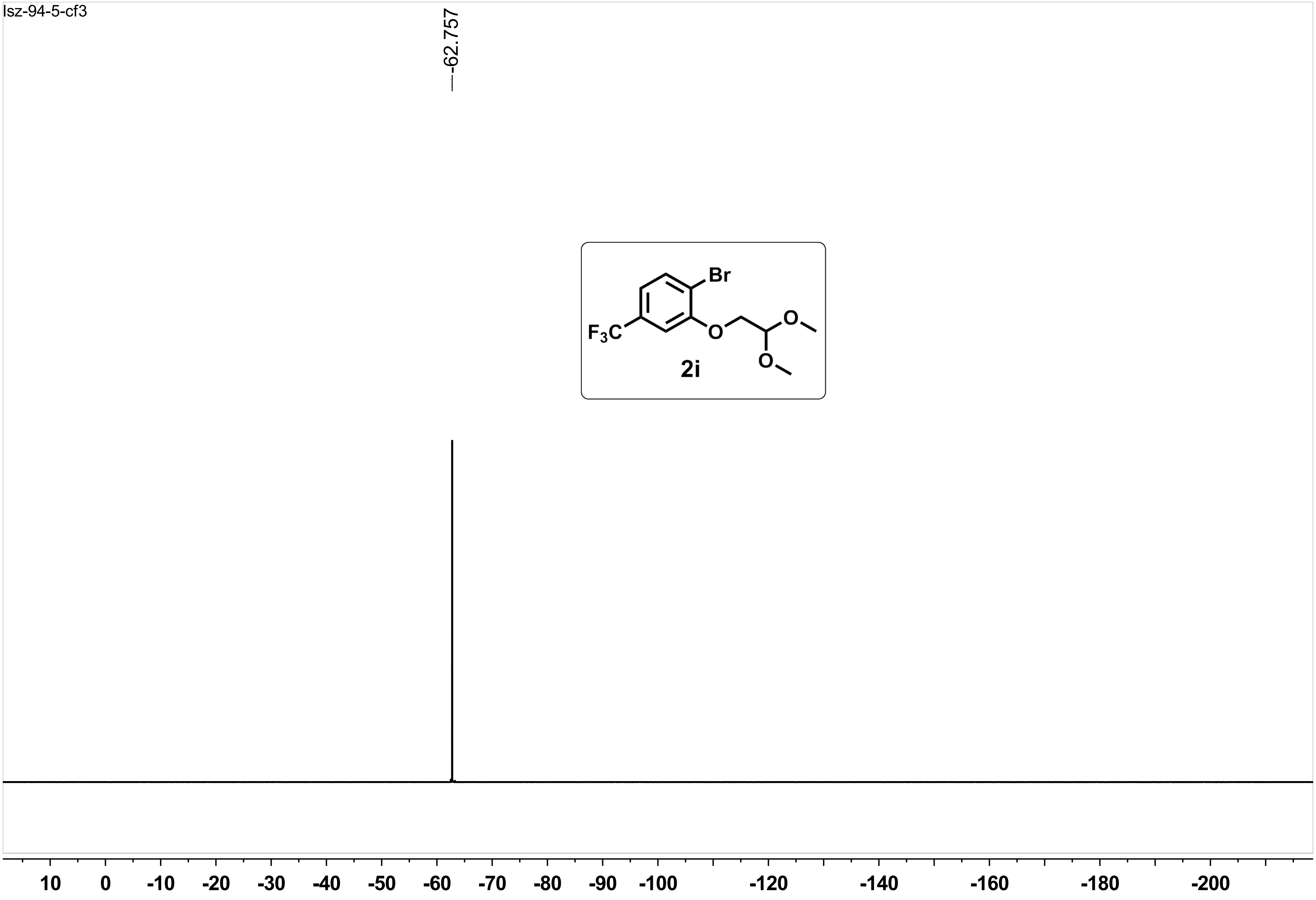

Supplementary Figure 22. ^{19}F NMR of **2i**

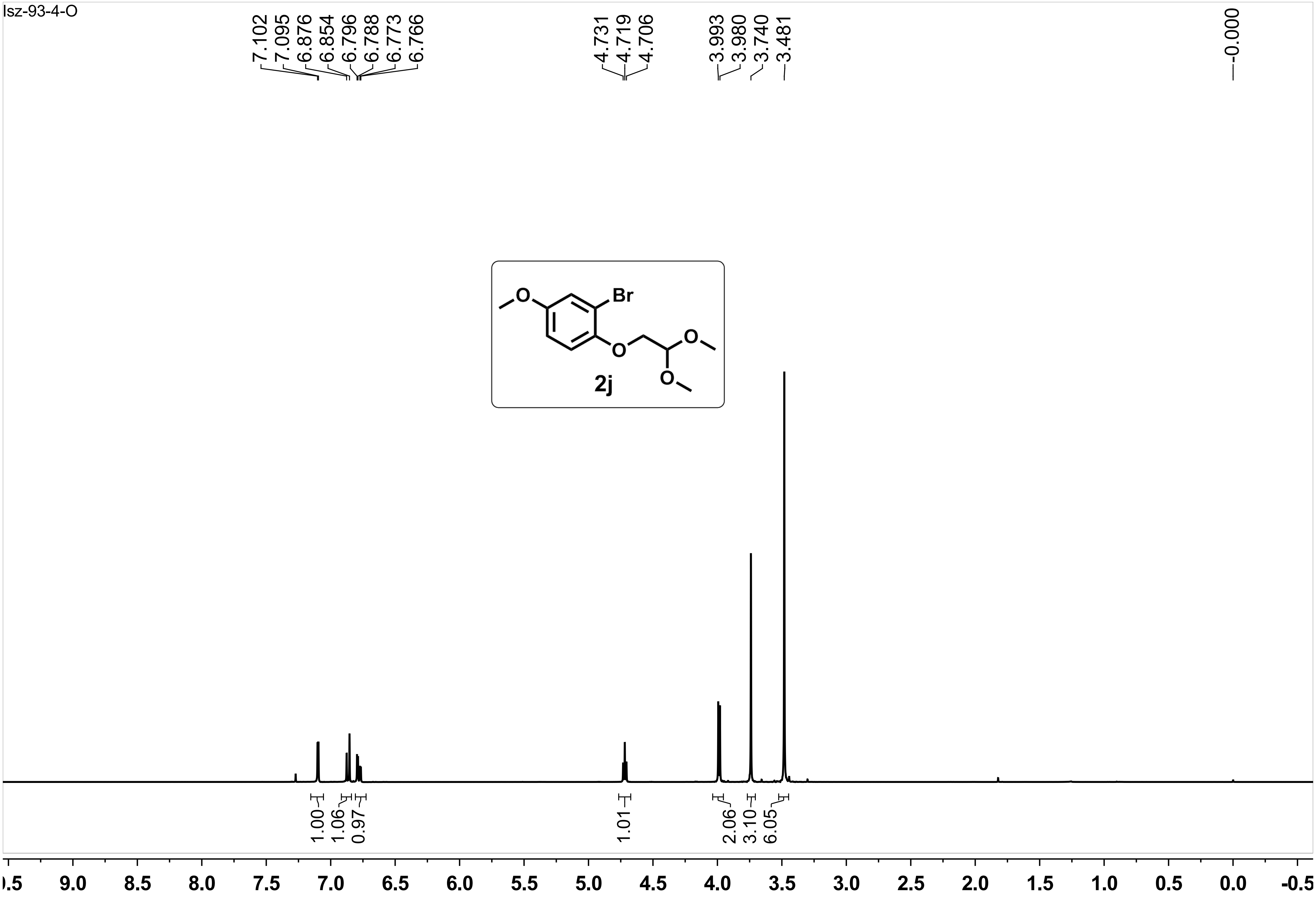

Supplementary Figure 23. ^{1}H NMR of **2j**

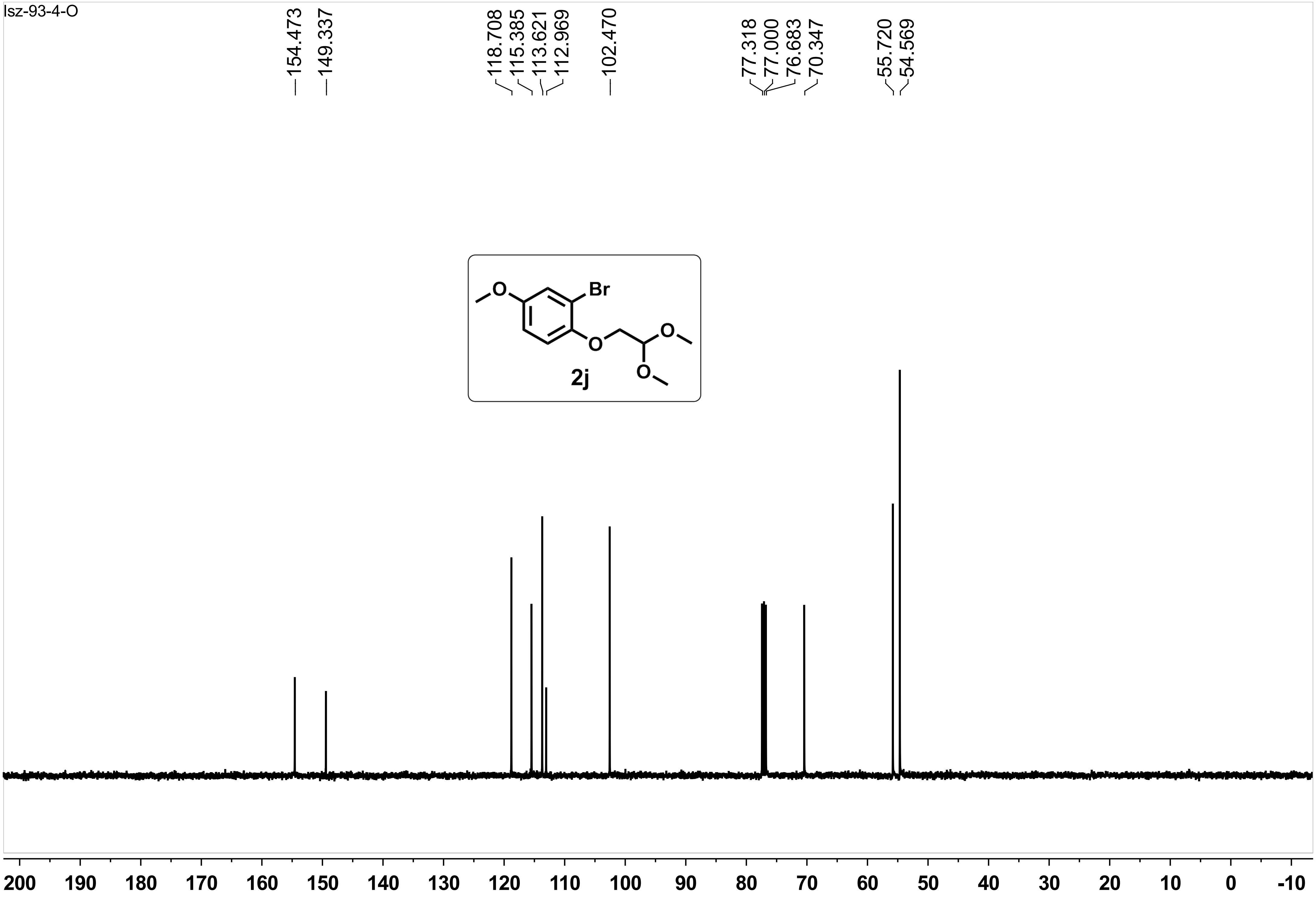

Supplementary Figure 24. ^{13}C NMR of **2j**

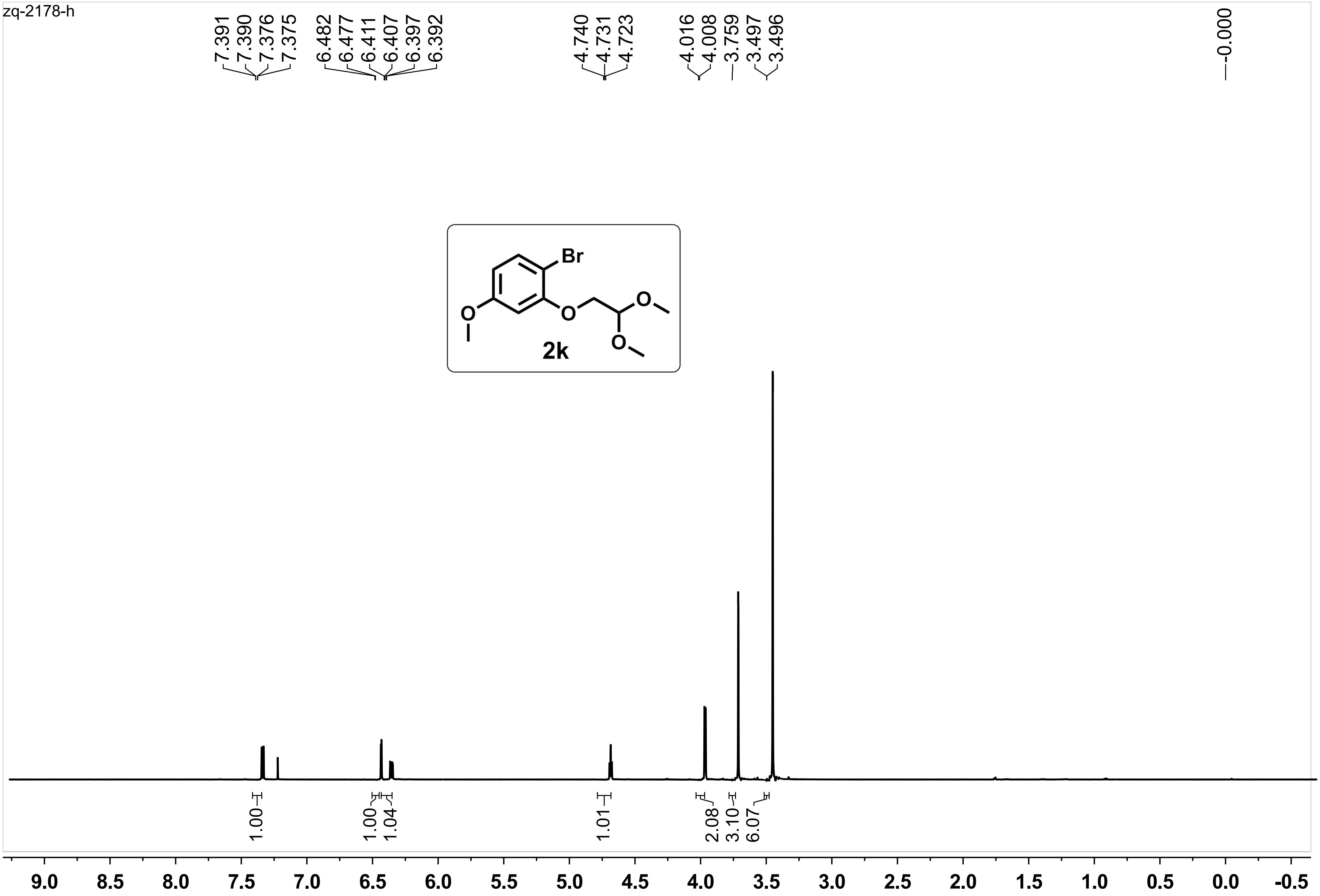

Supplementary Figure 25. ^{1}H NMR of **2k**

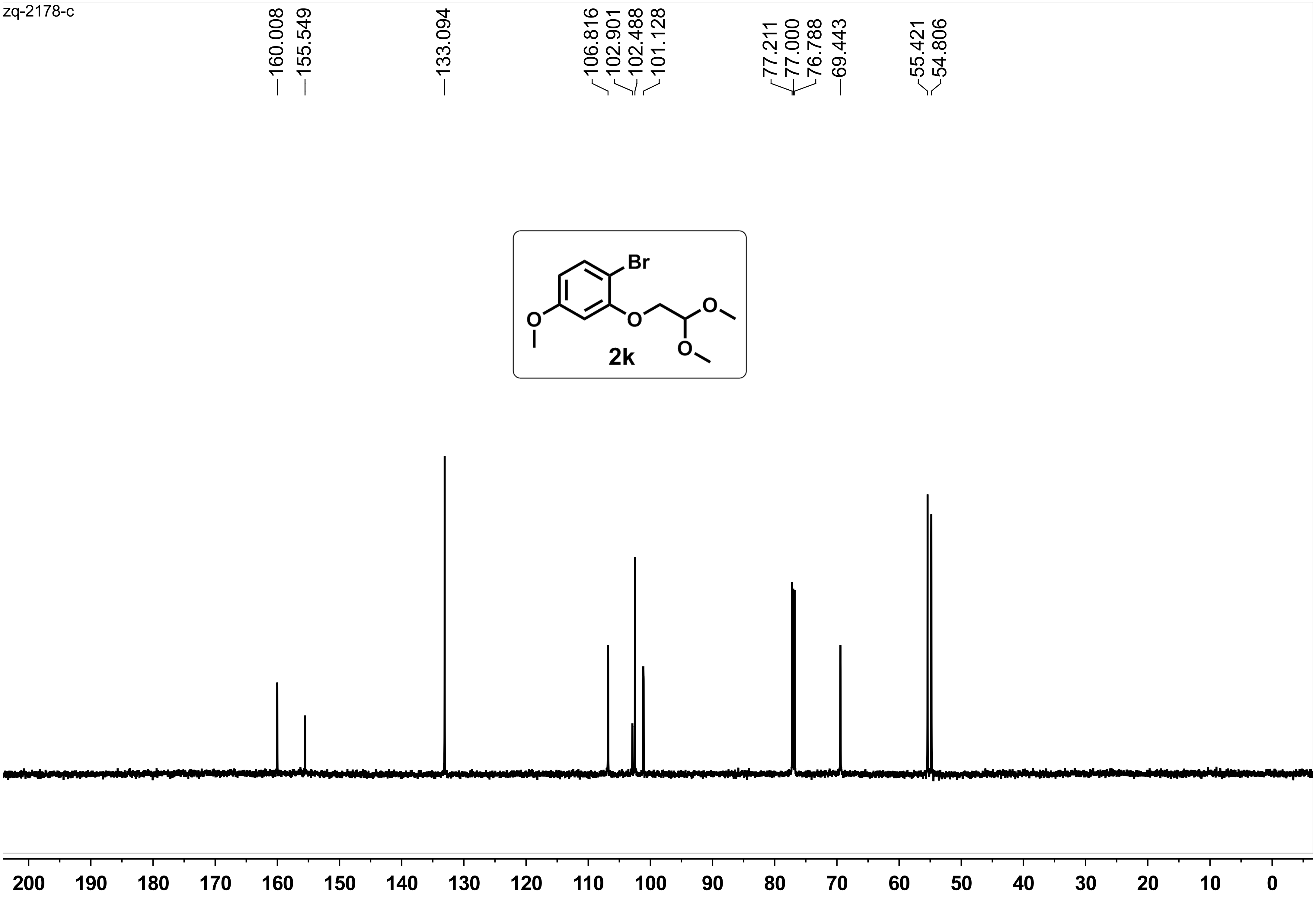

Supplementary Figure 26. ^{13}C NMR of **2k**

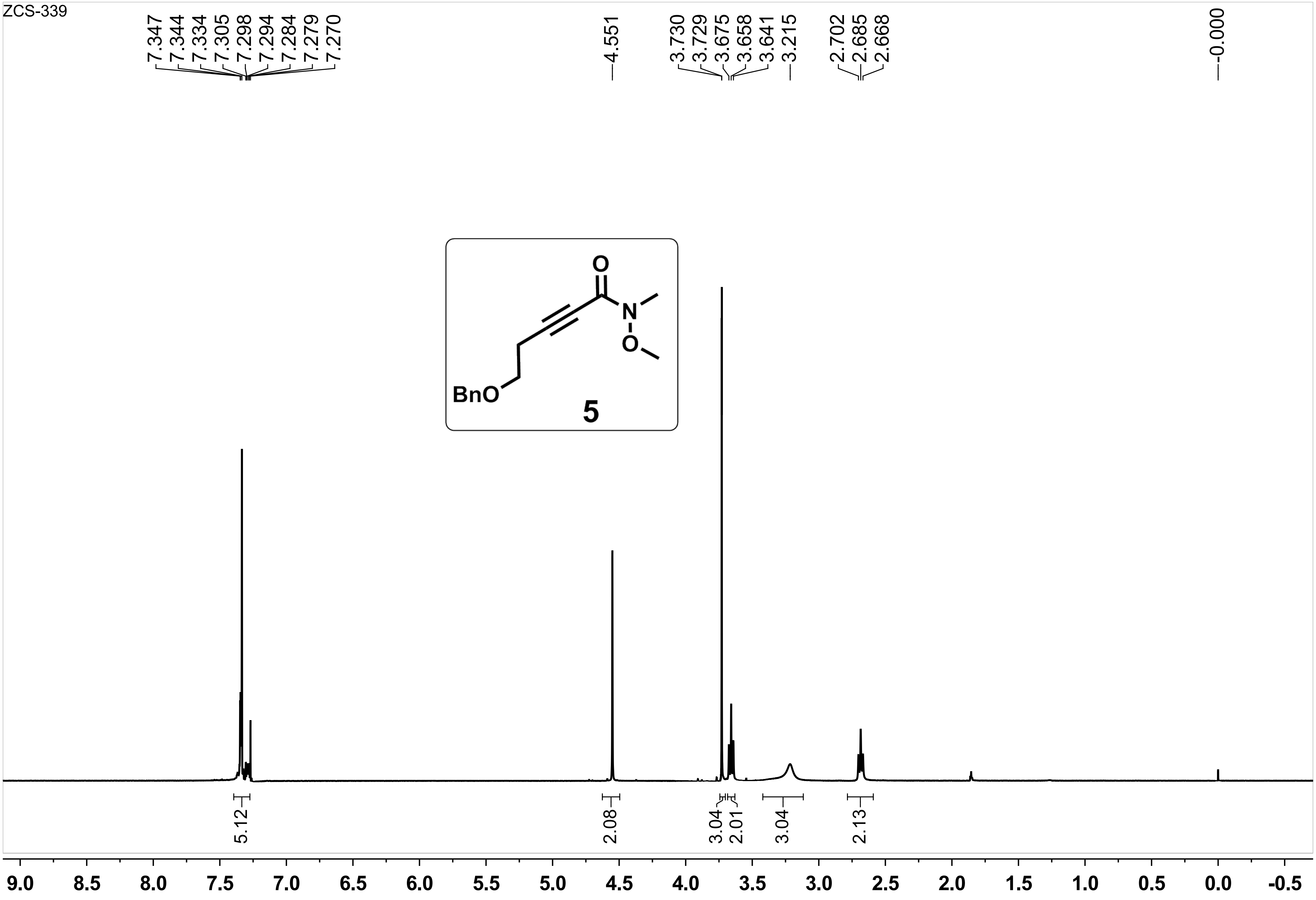

Supplementary Figure 27. ^{1}H NMR of **5**

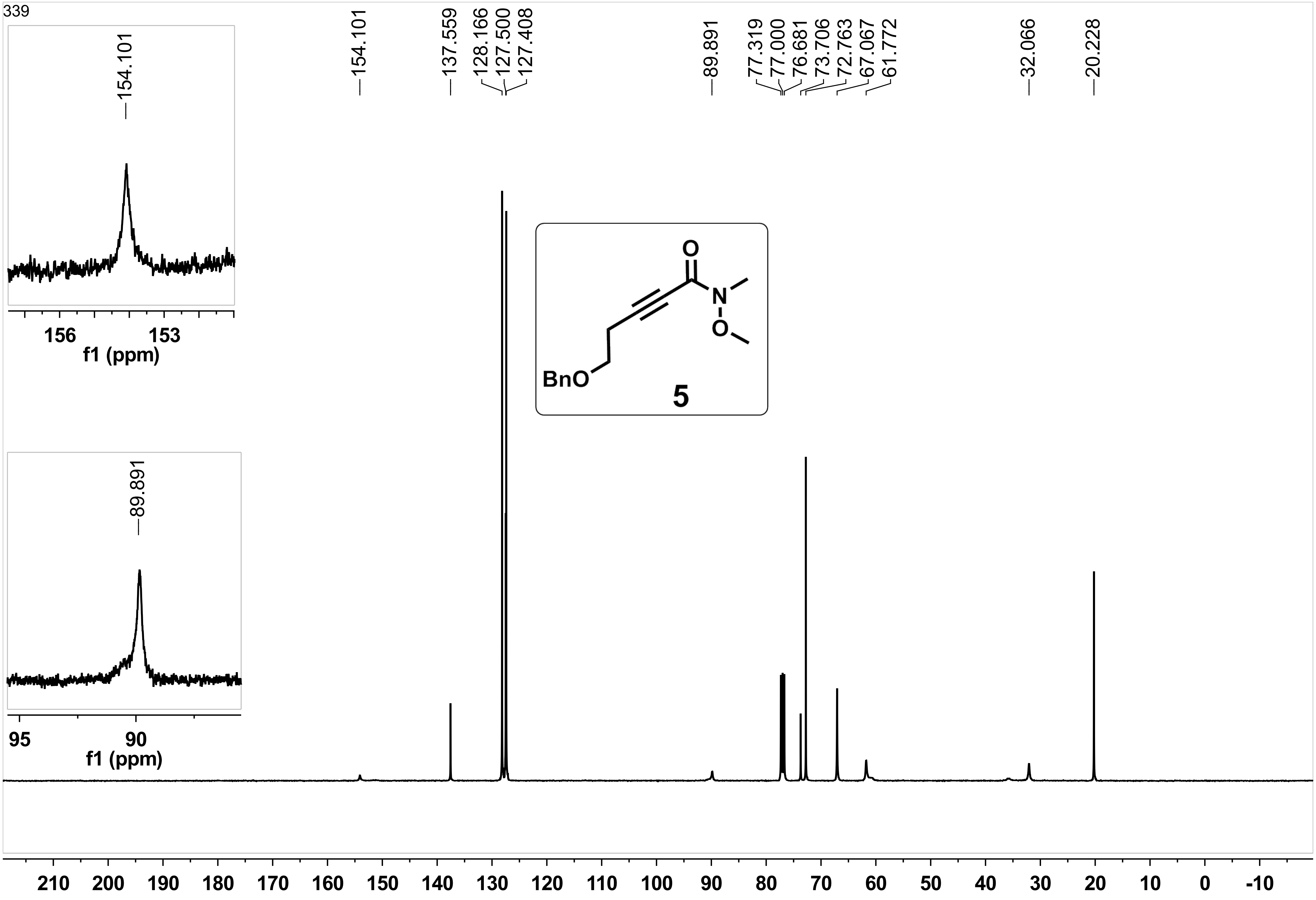

Supplementary Figure 28. ^{13}C NMR of **5**

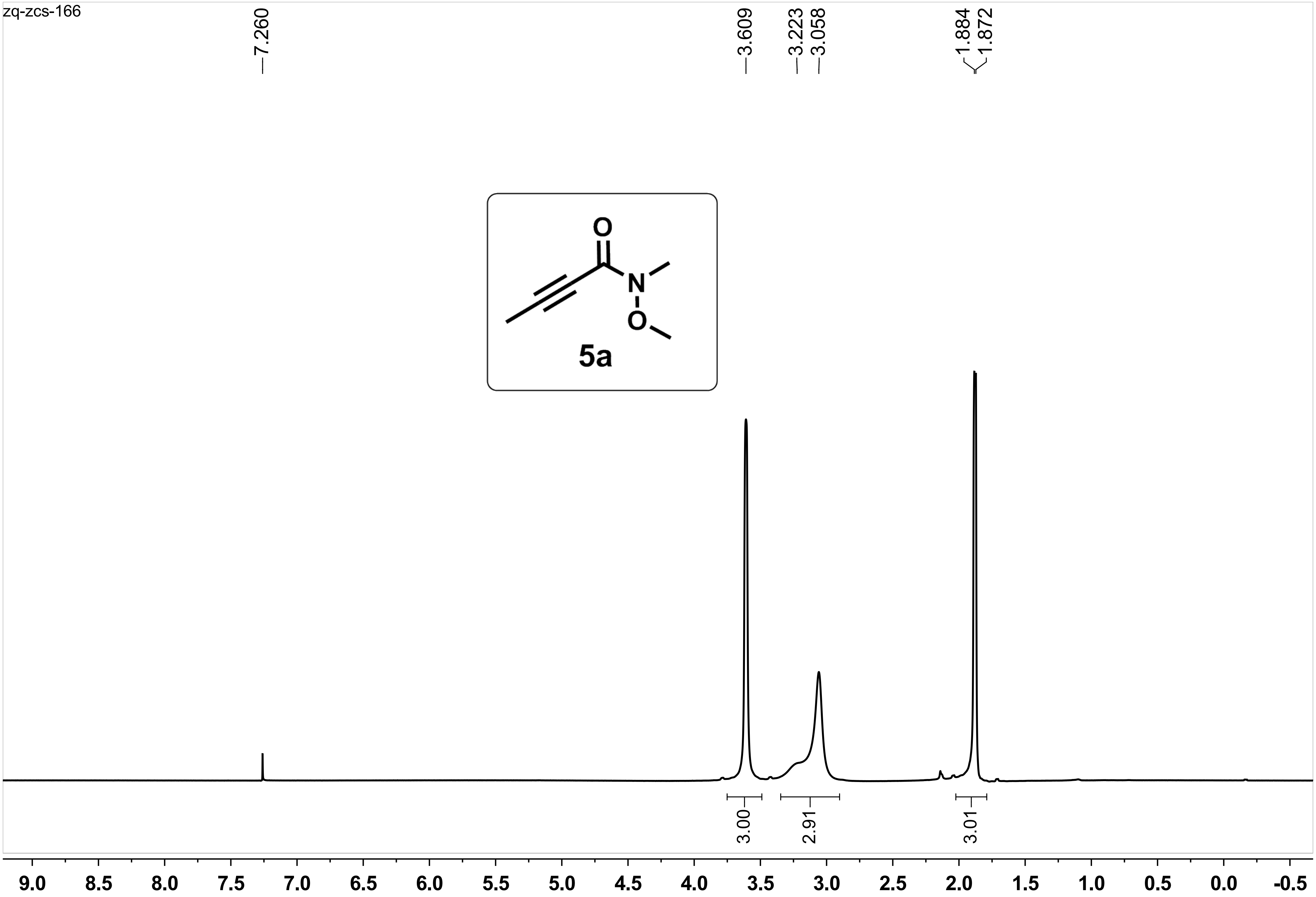

Supplementary Figure 29. ^{1}H NMR of **5a**

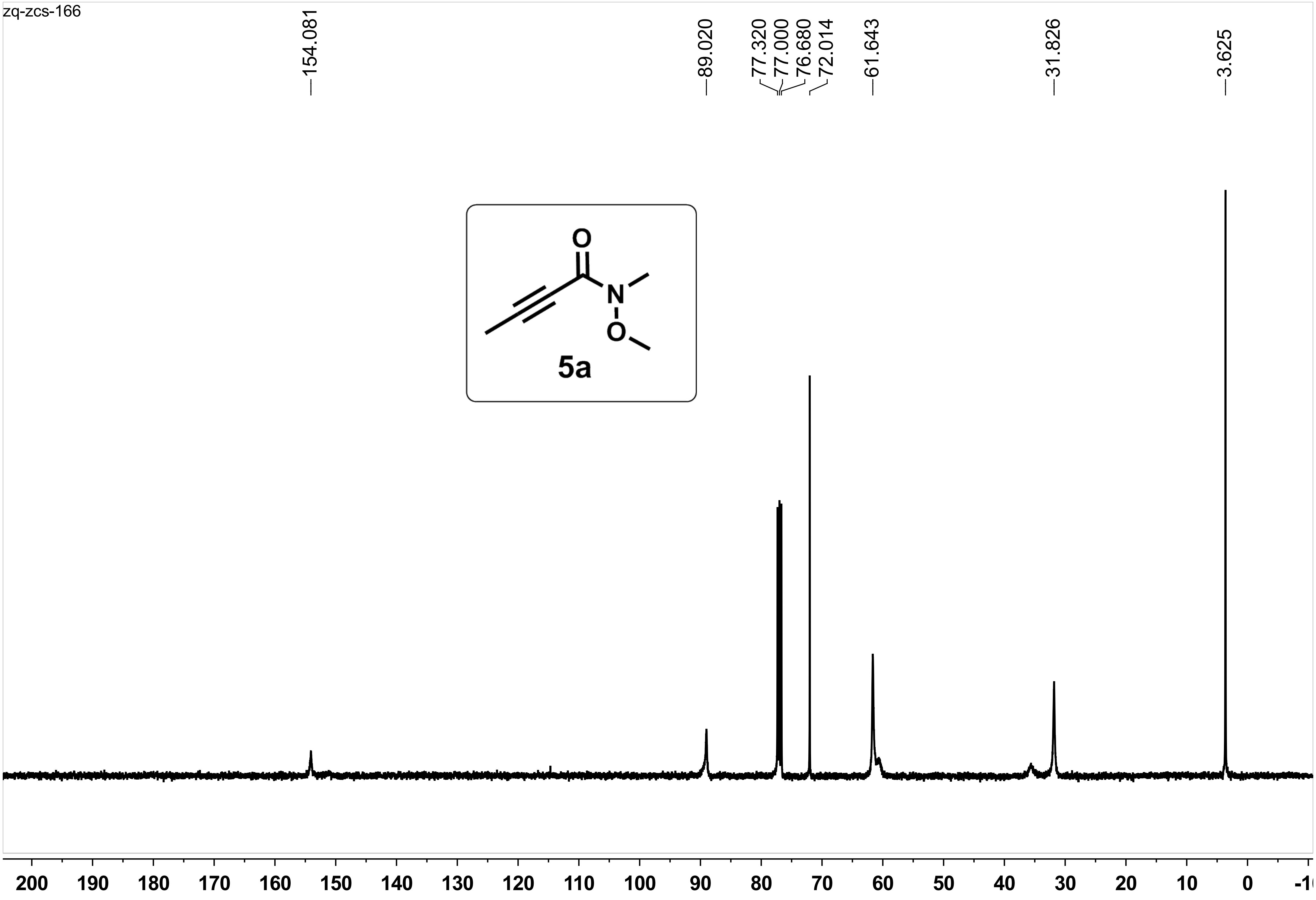

Supplementary Figure 30. ^{13}C NMR of **5a**

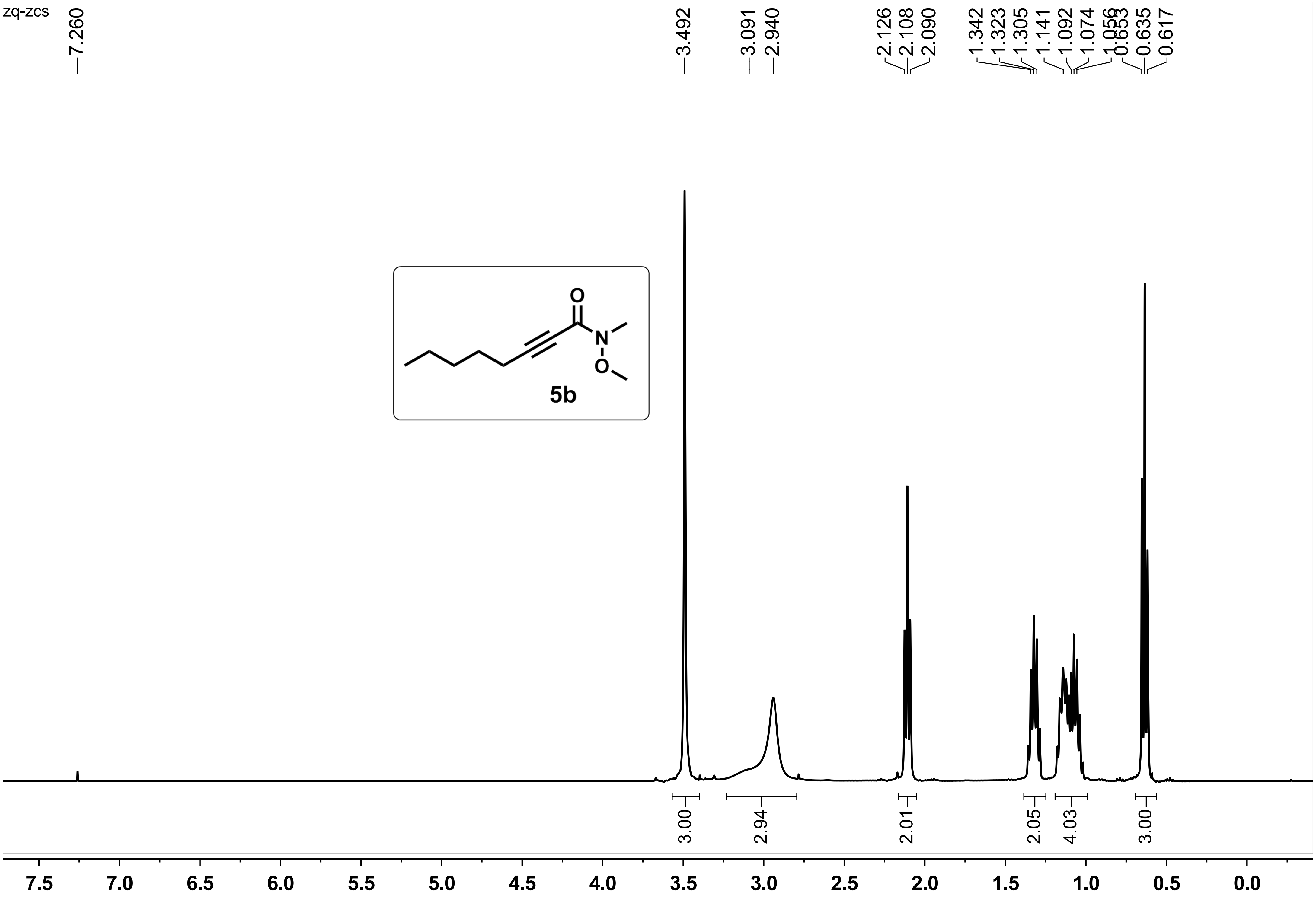

Supplementary Figure 31. ^{1}H NMR of **5b**

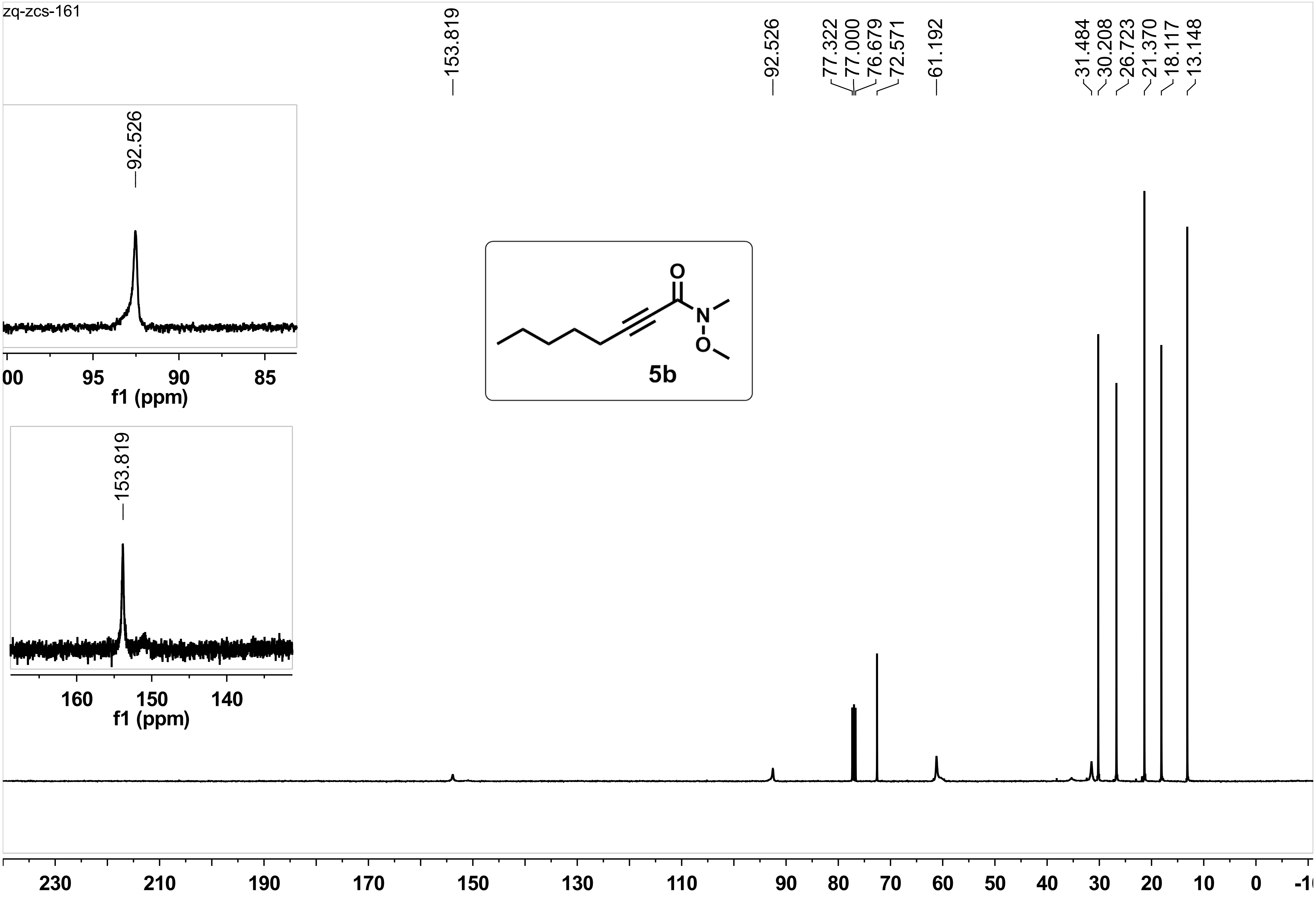

Supplementary Figure 32. ^{13}C NMR of **5b**

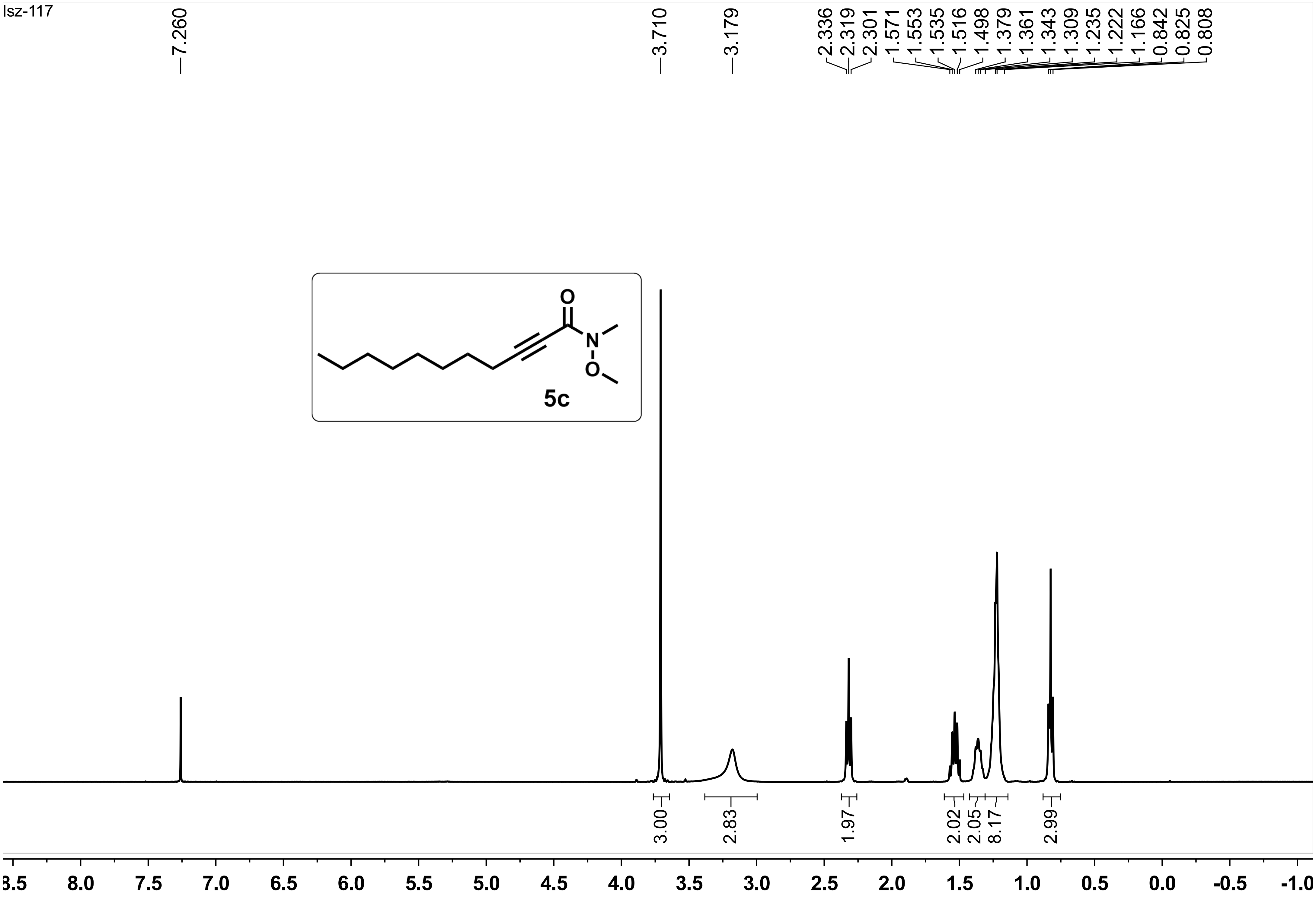

Supplementary Figure 33. ^{1}H NMR of **5c**

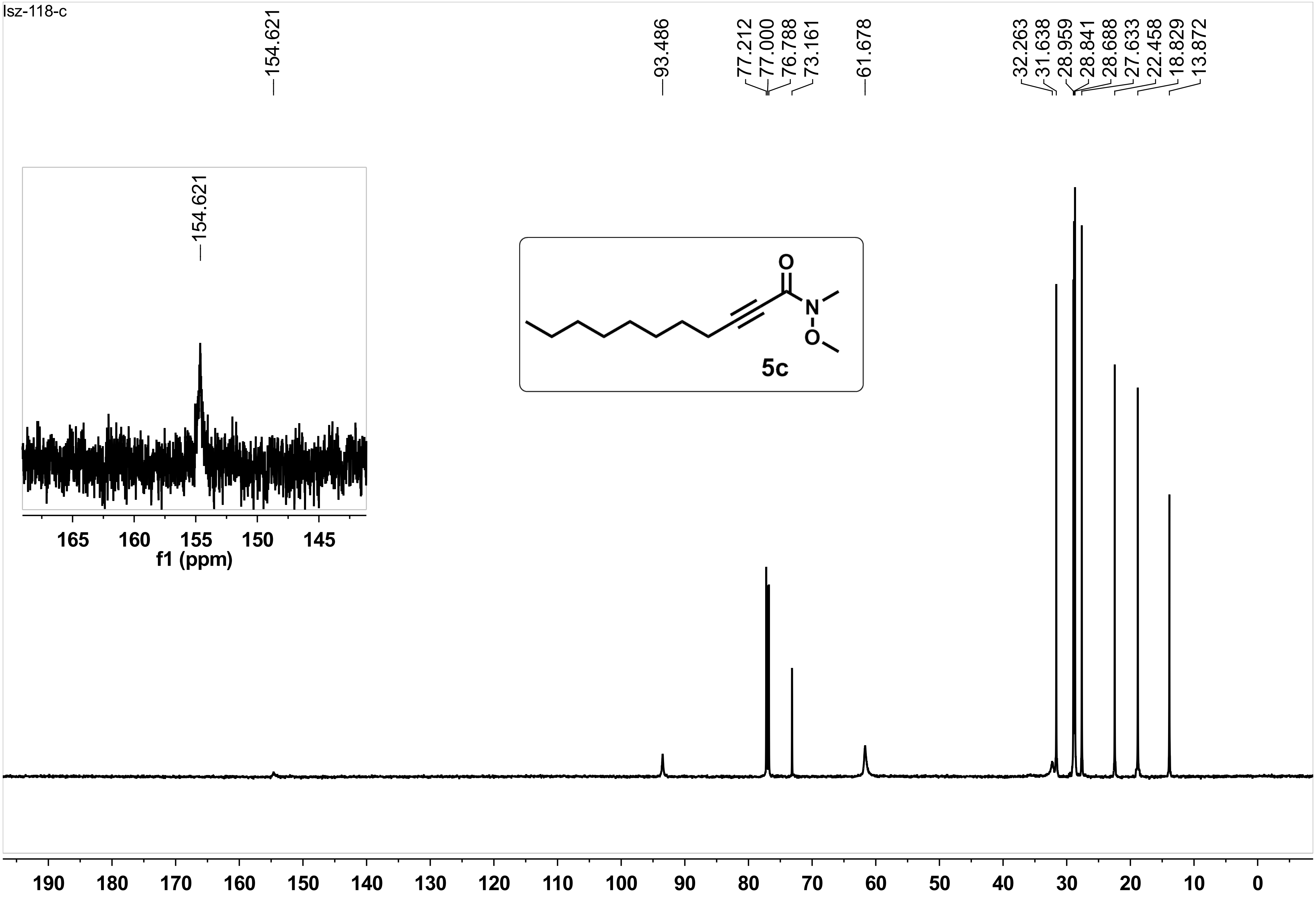

Supplementary Figure 34. ^{13}C NMR of **5c**

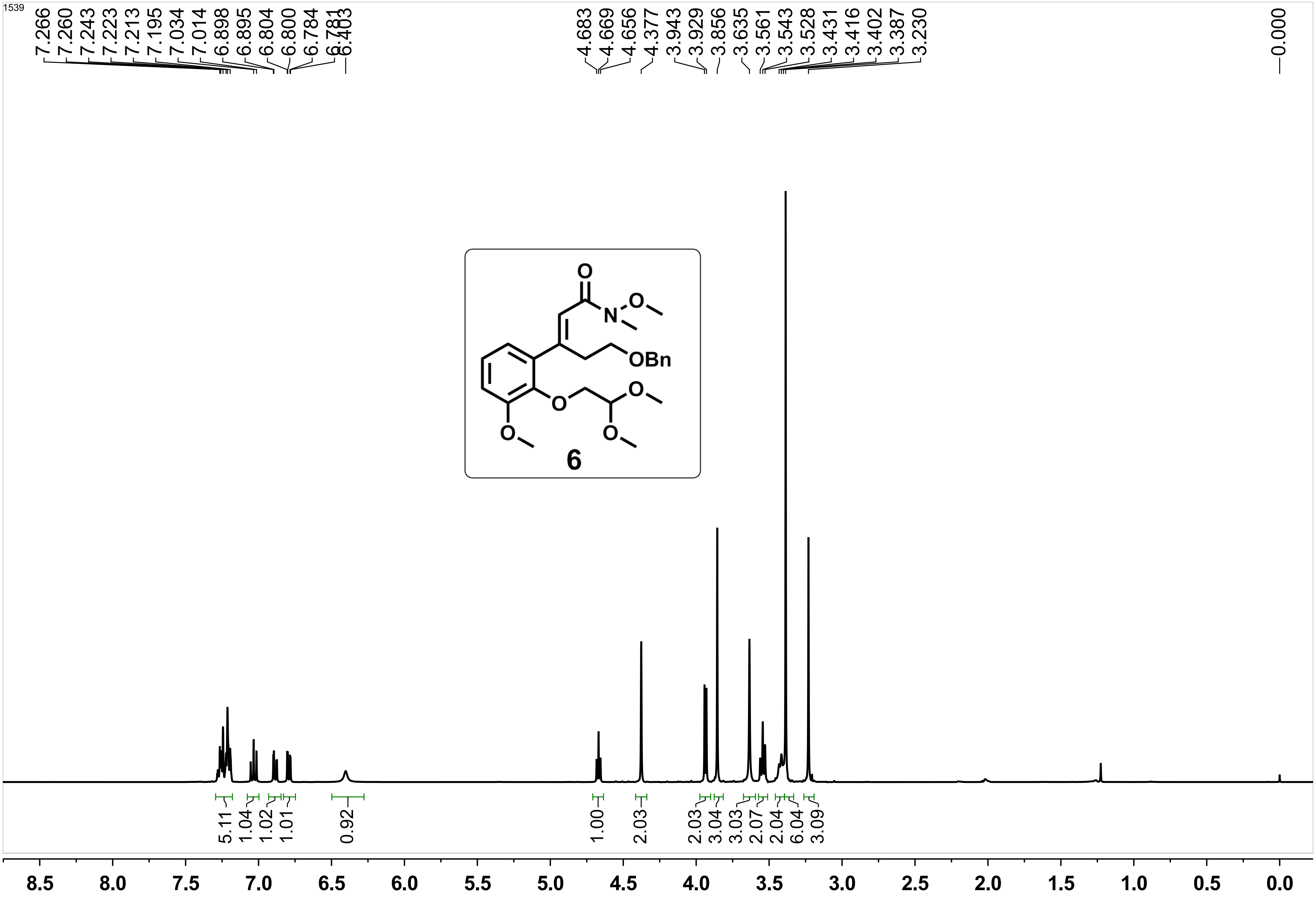

Supplementary Figure 35. ^{1}H NMR of **6**

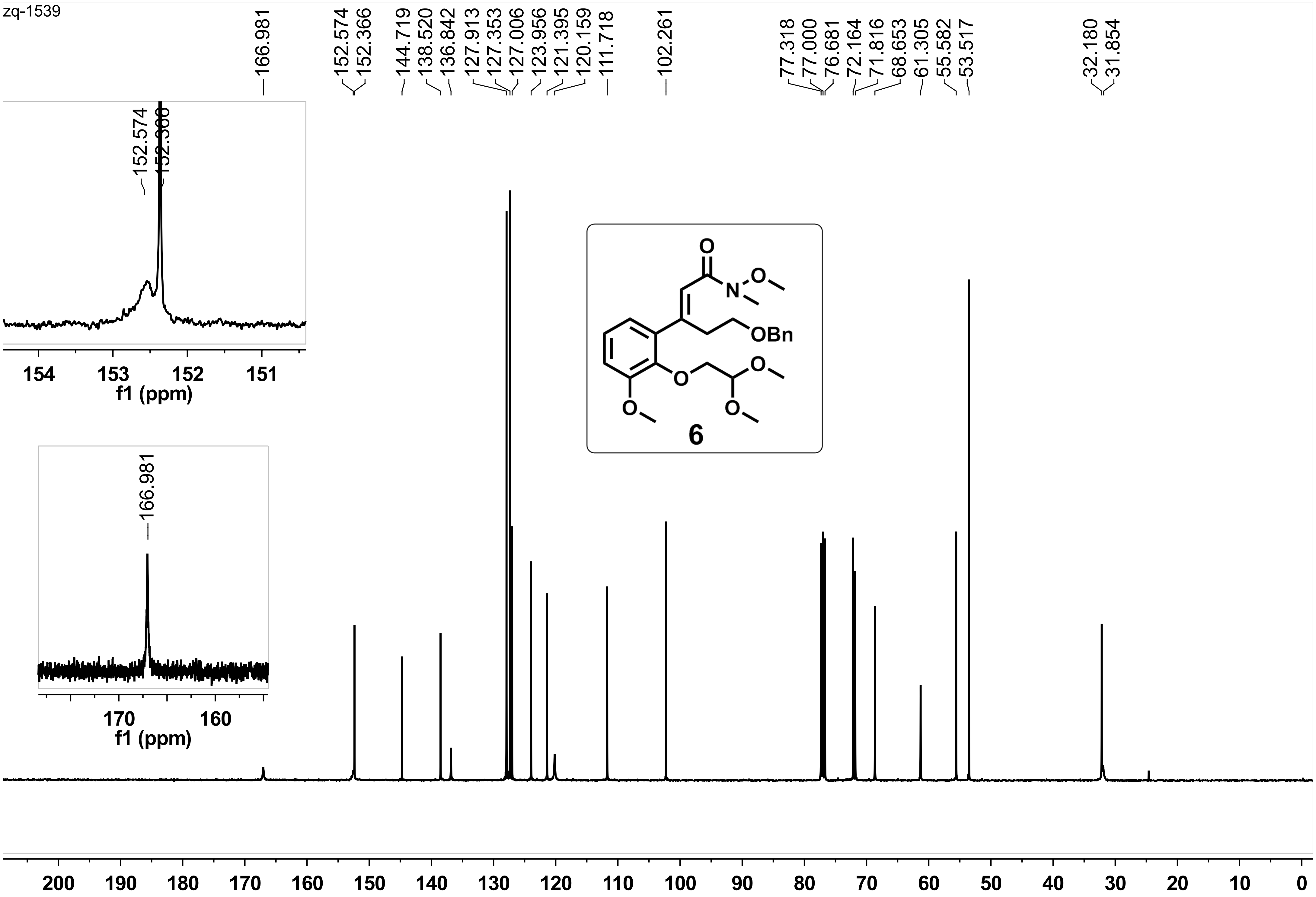

Supplementary Figure 36. ^{13}C NMR of **6**

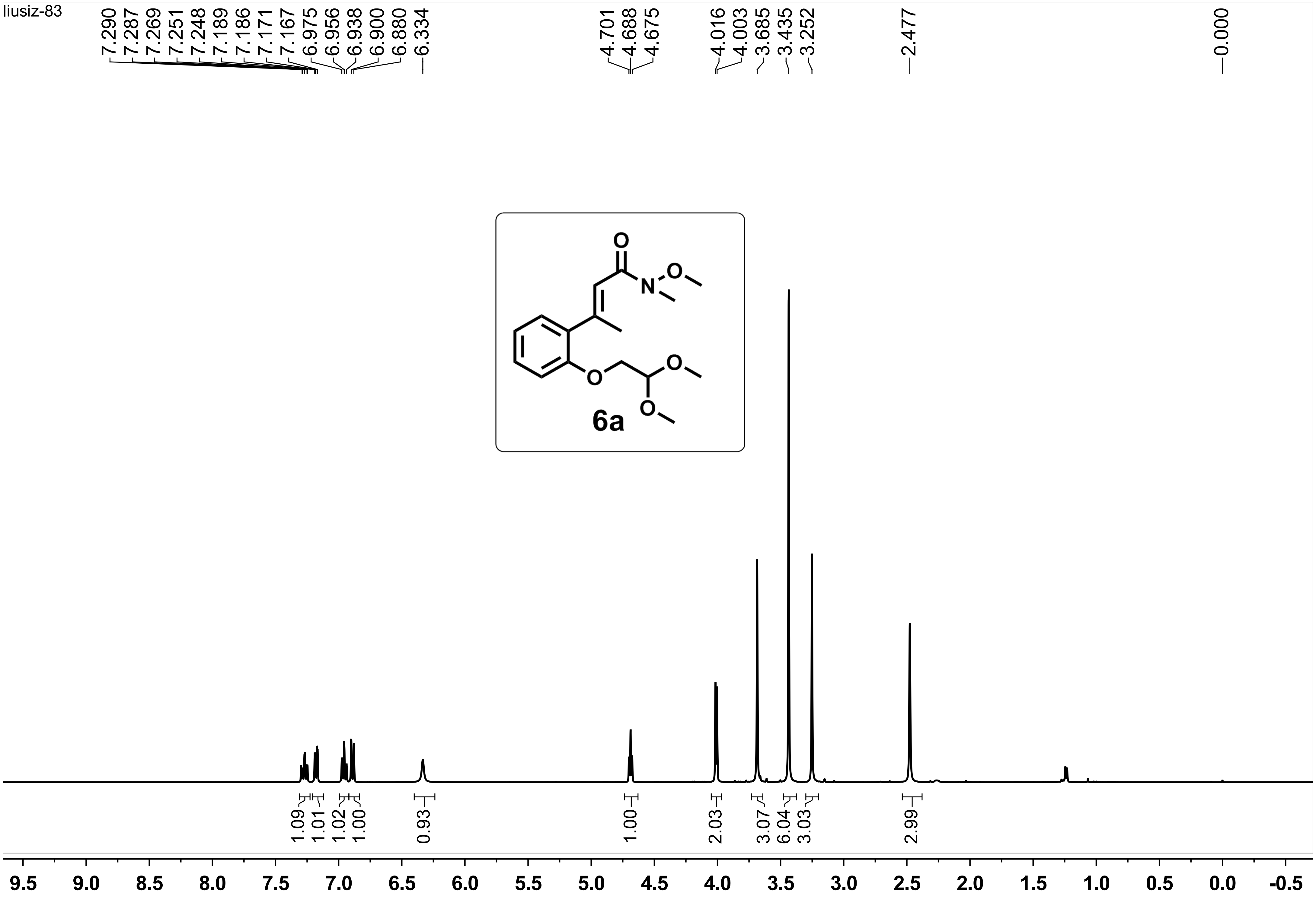

Supplementary Figure 37. ^{1}H NMR of **6a**

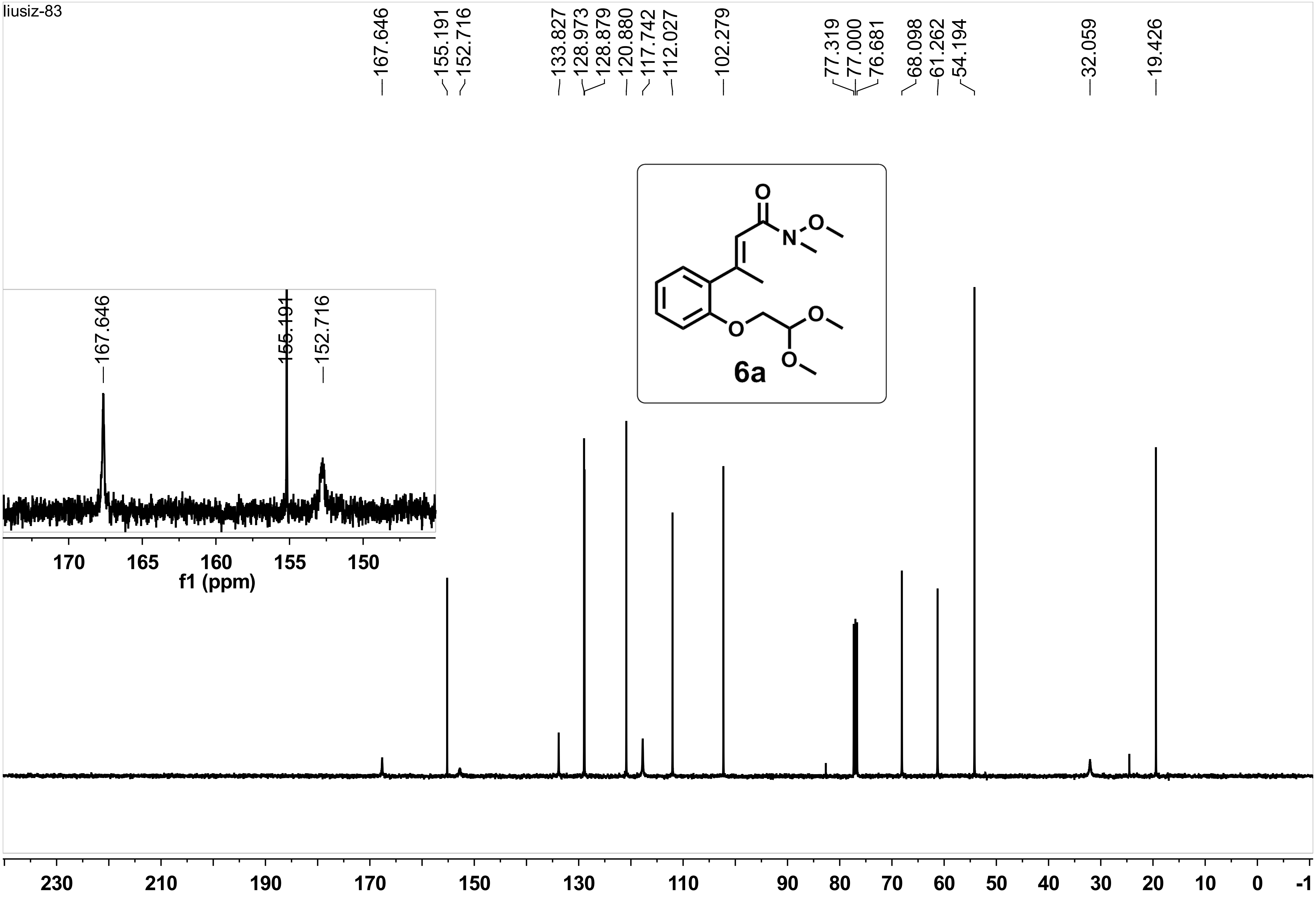

Supplementary Figure 38. ^{13}C NMR of **6a**

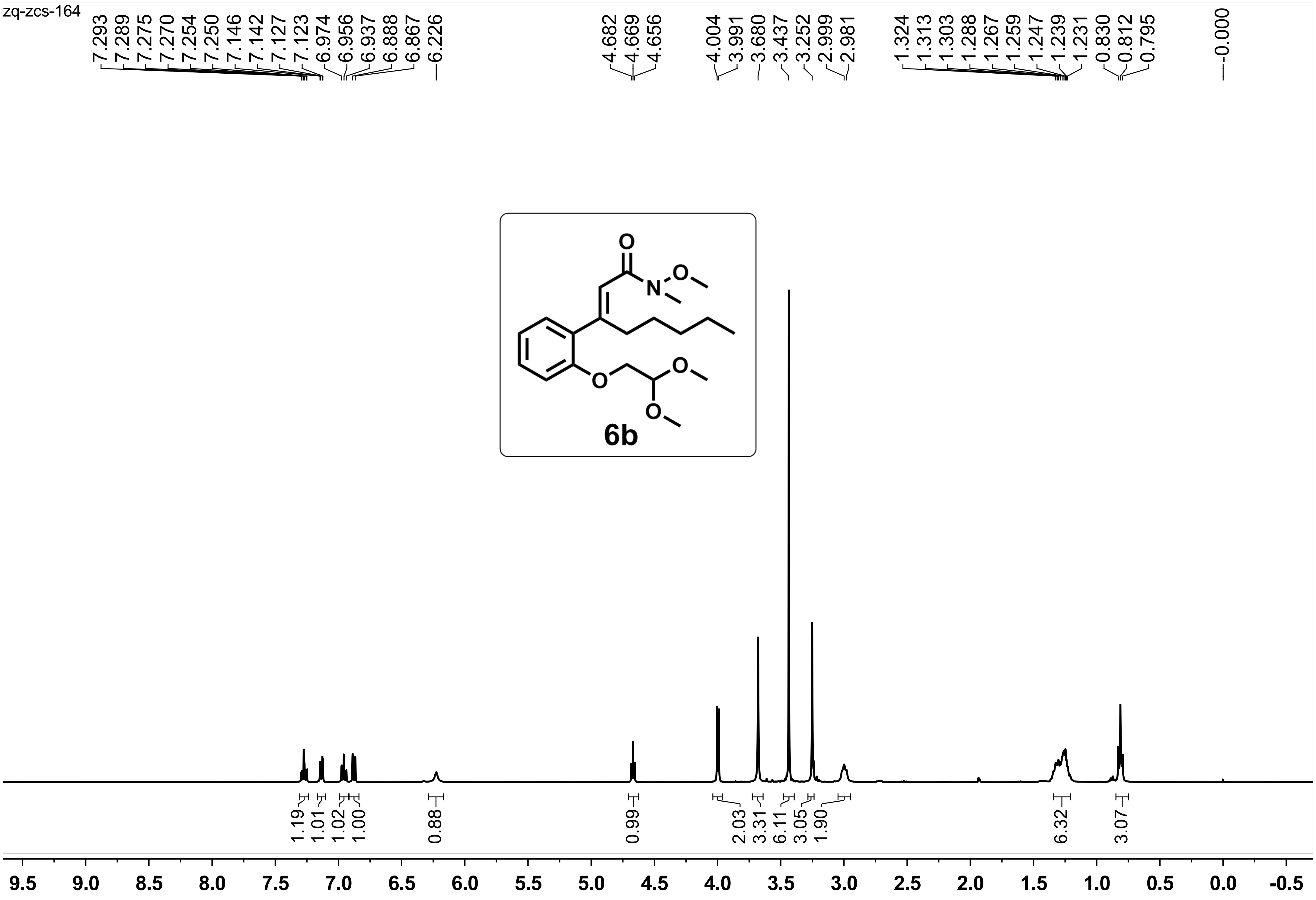

Supplementary Figure 39. ^{1}H NMR of **6b**

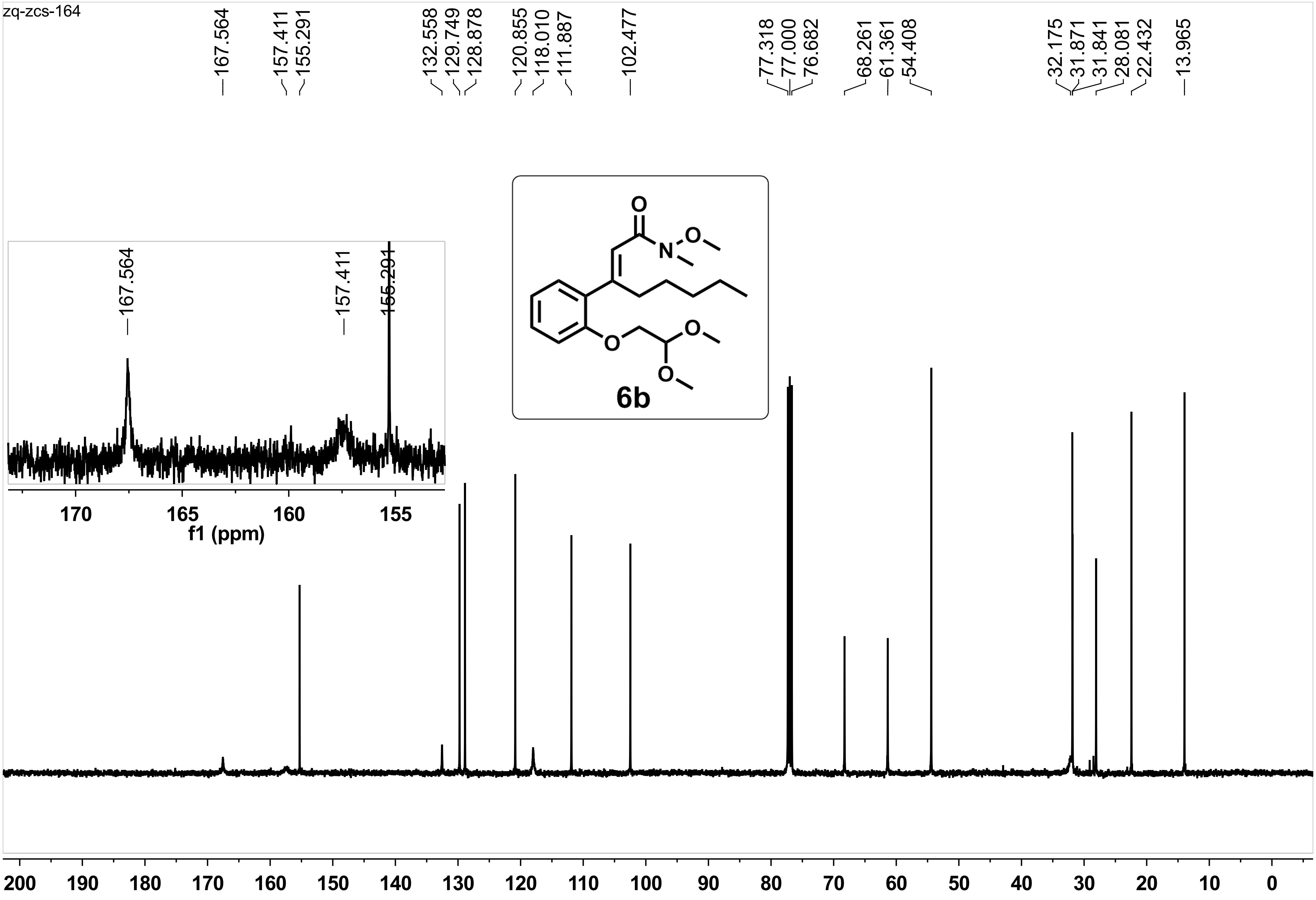

Supplementary Figure 40. ^{13}C NMR of **6b**

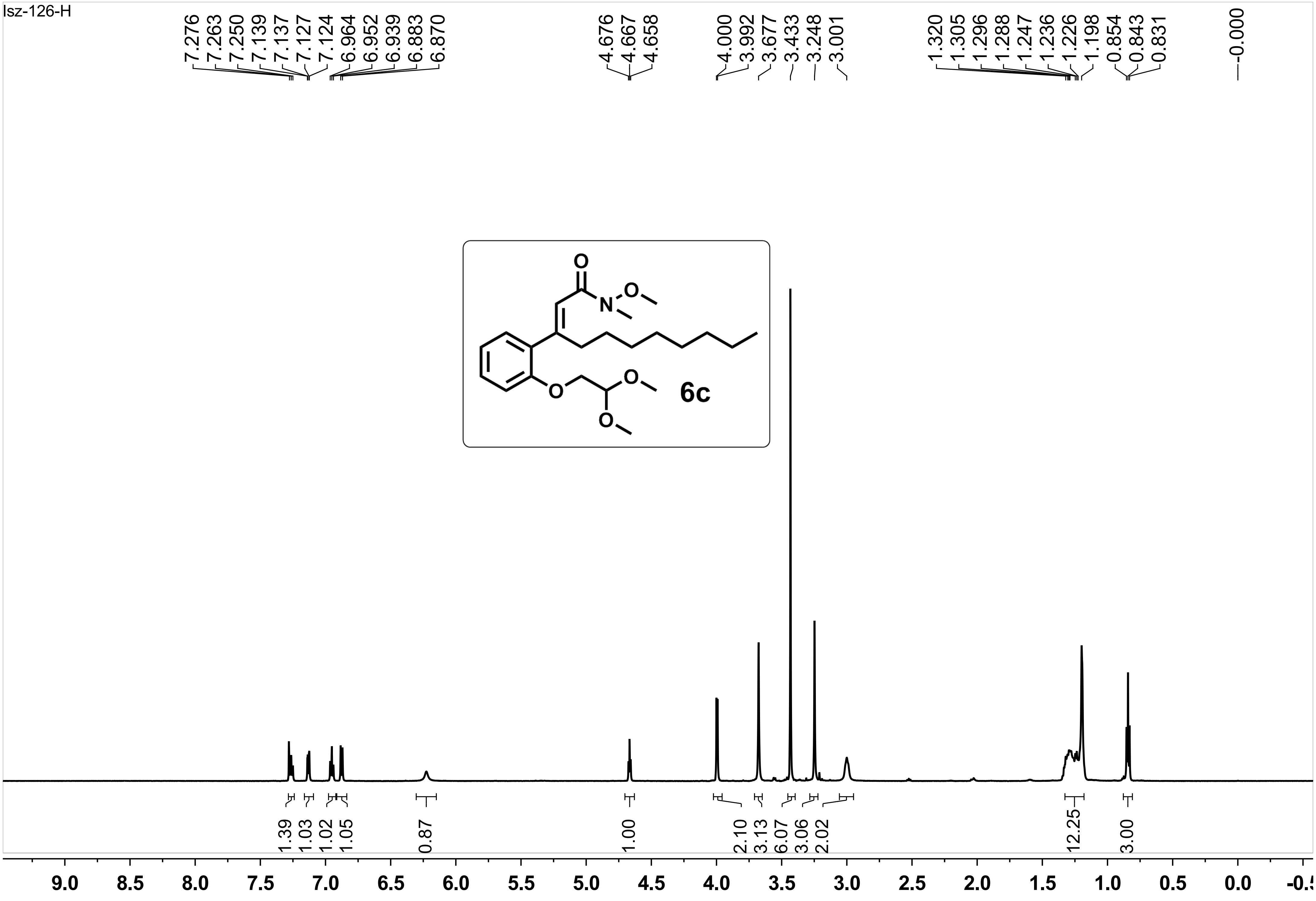

Supplementary Figure 41. ^{1}H NMR of **6c**

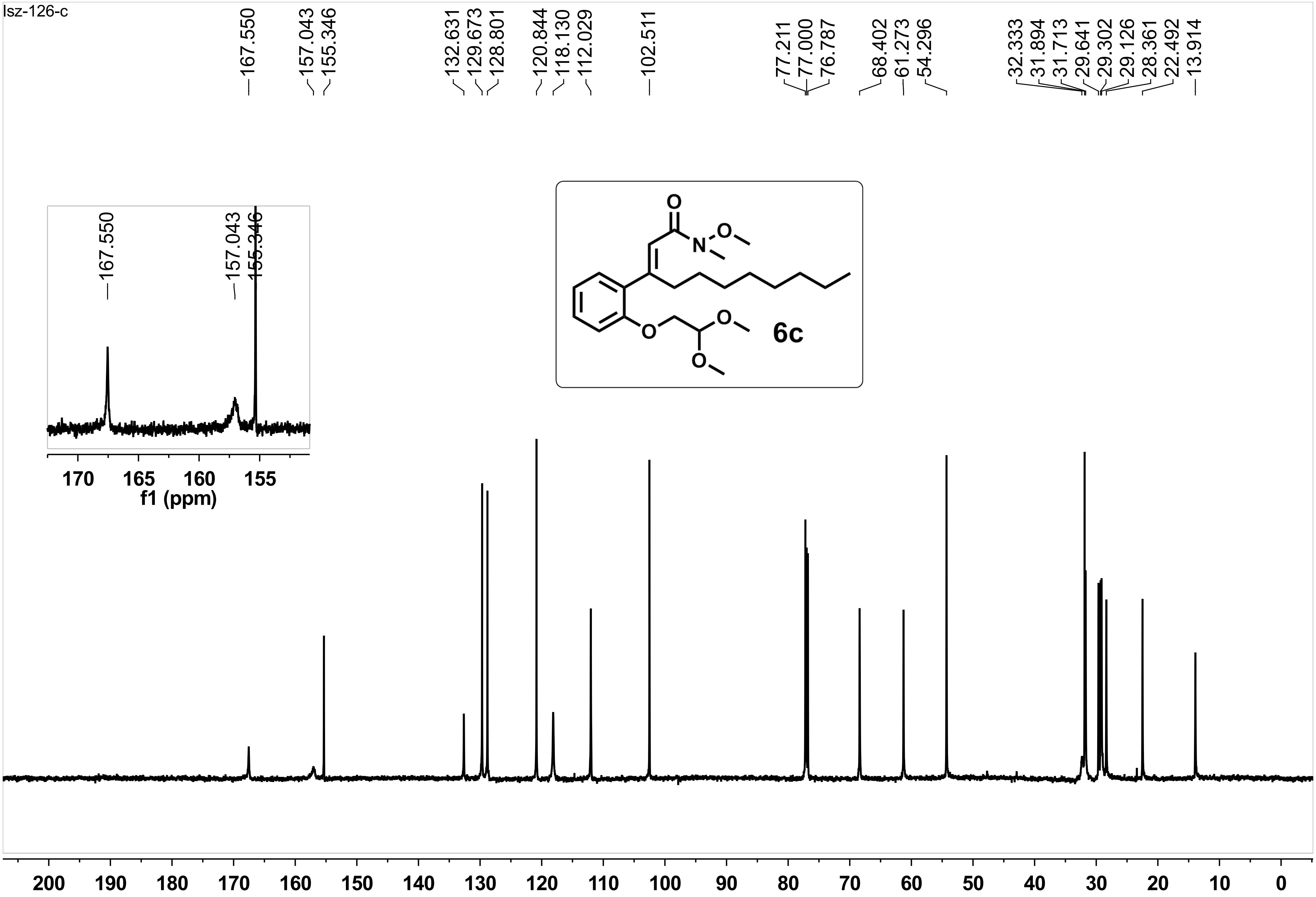

Supplementary Figure 42. ^{13}C NMR of **6c**

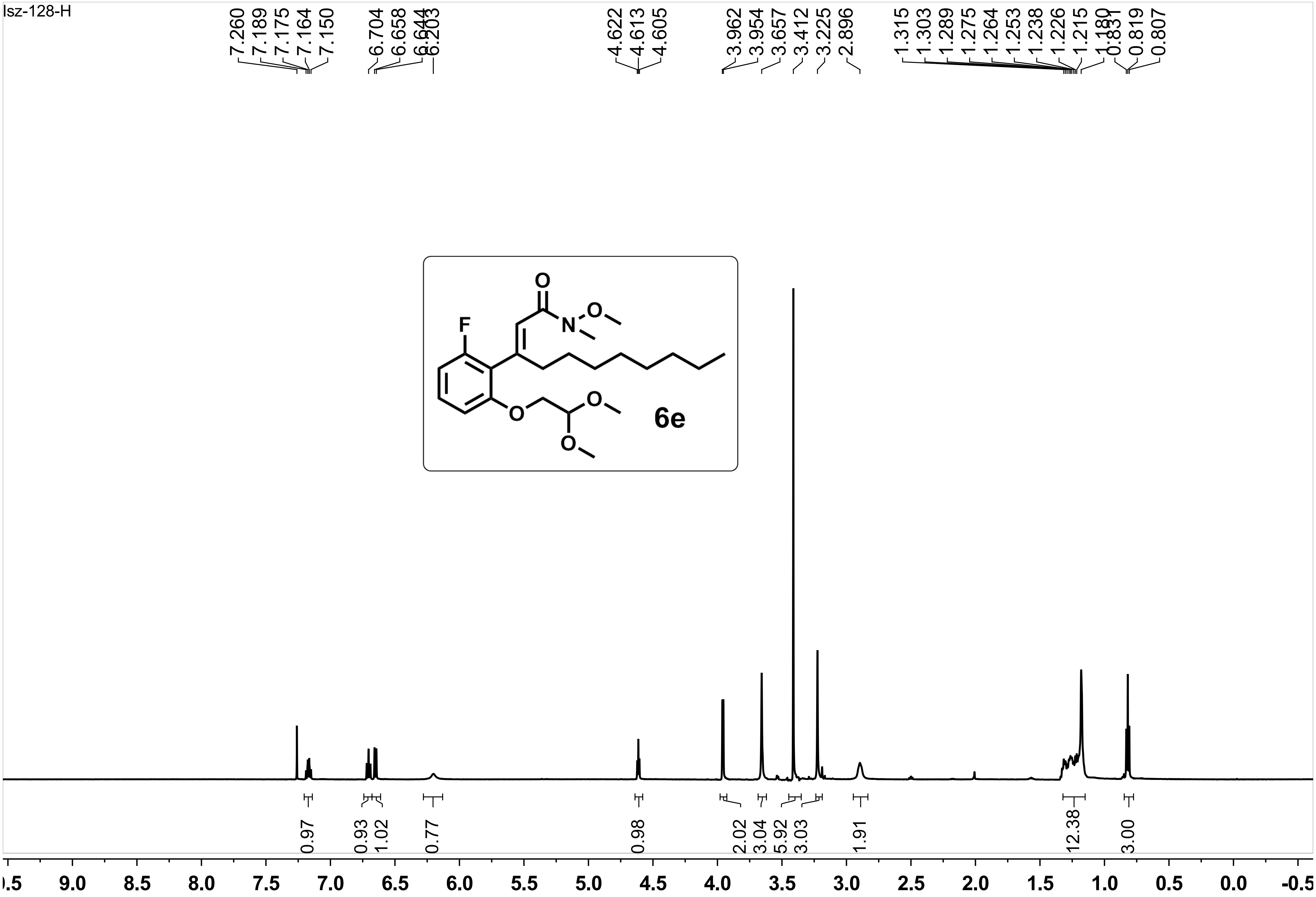

Supplementary Figure 43. ^{1}H NMR of **6e**

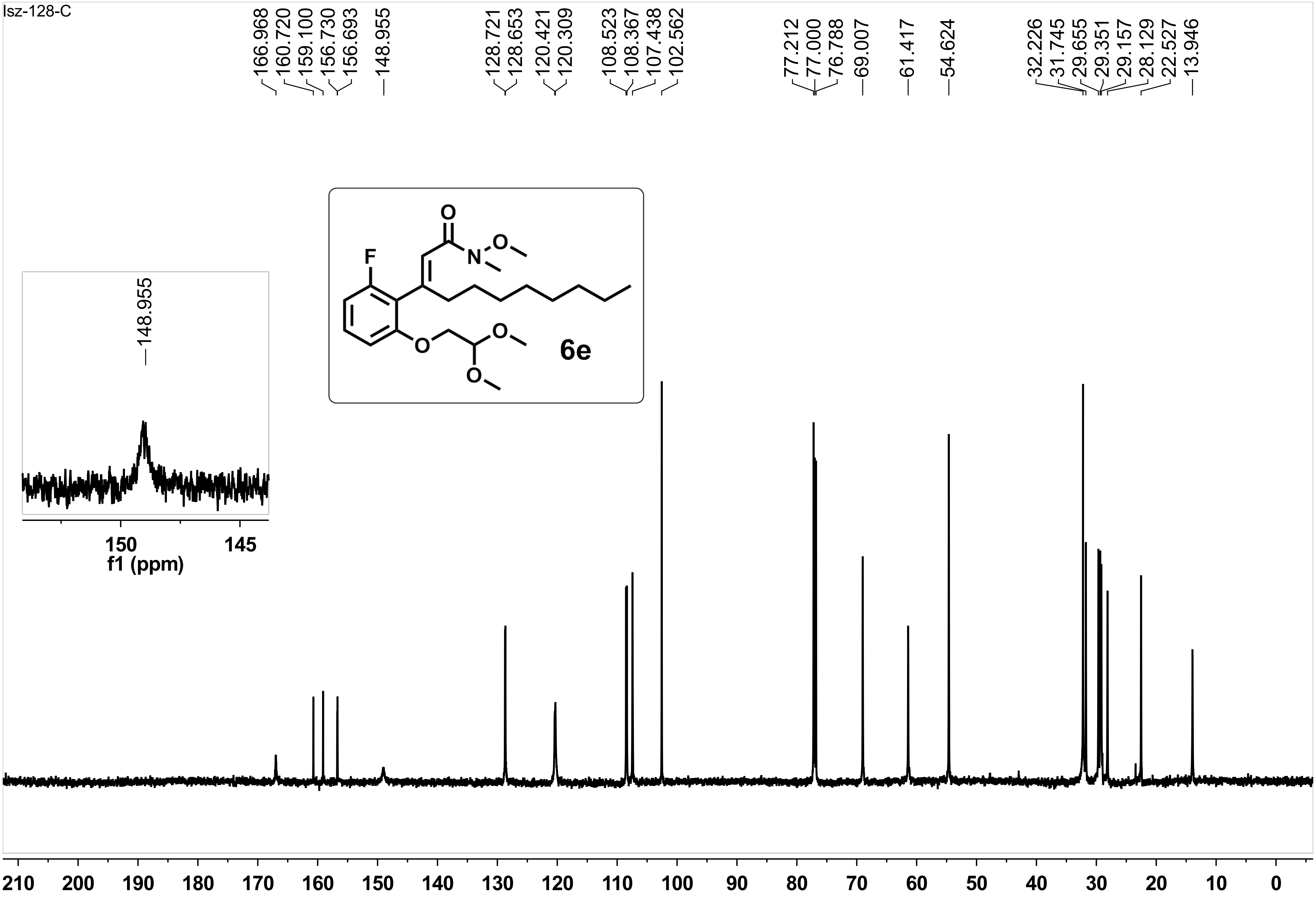

Supplementary Figure 44. ^{13}C NMR of **6e**

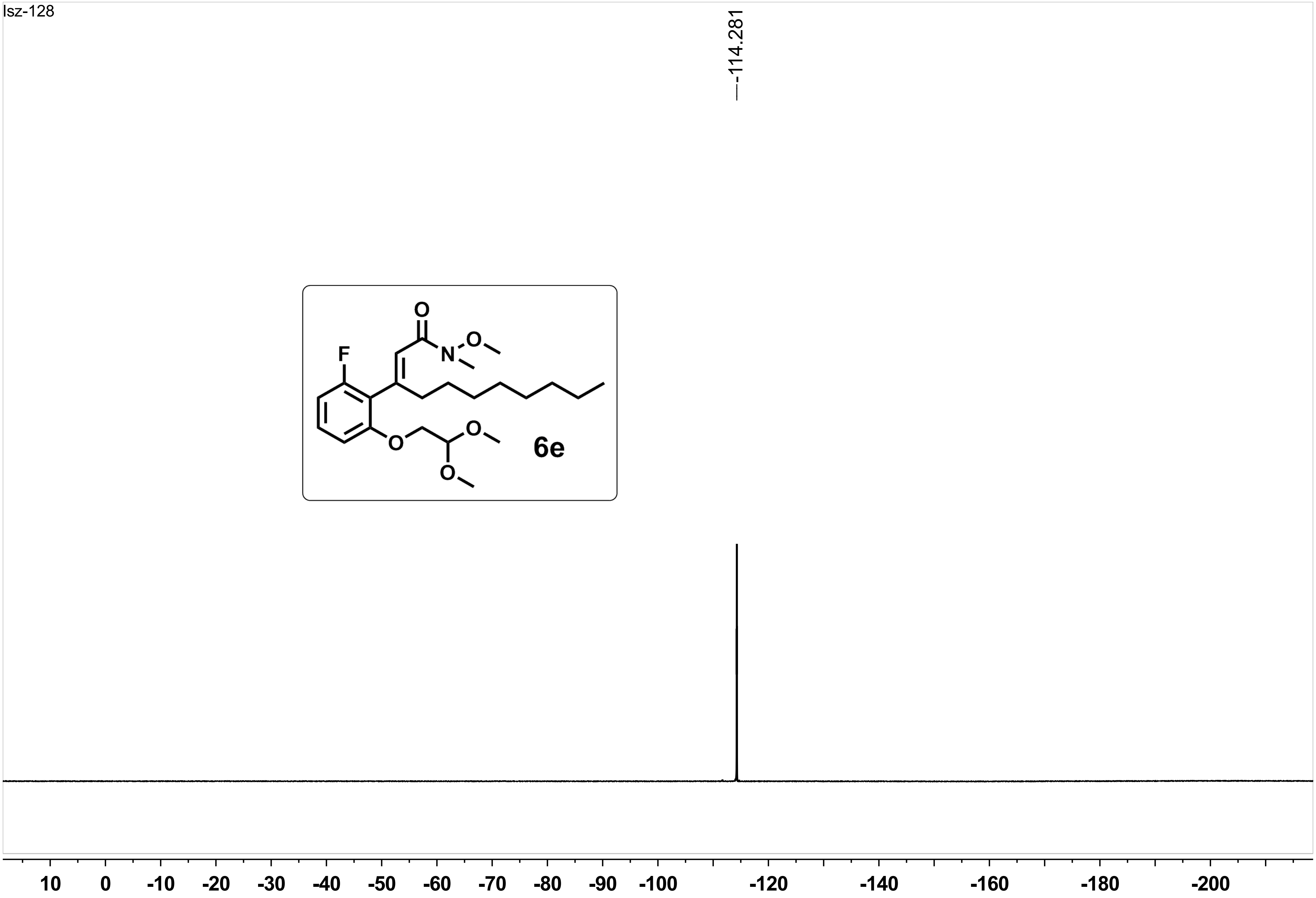

Supplementary Figure 45. ^{19}F NMR of **6e**

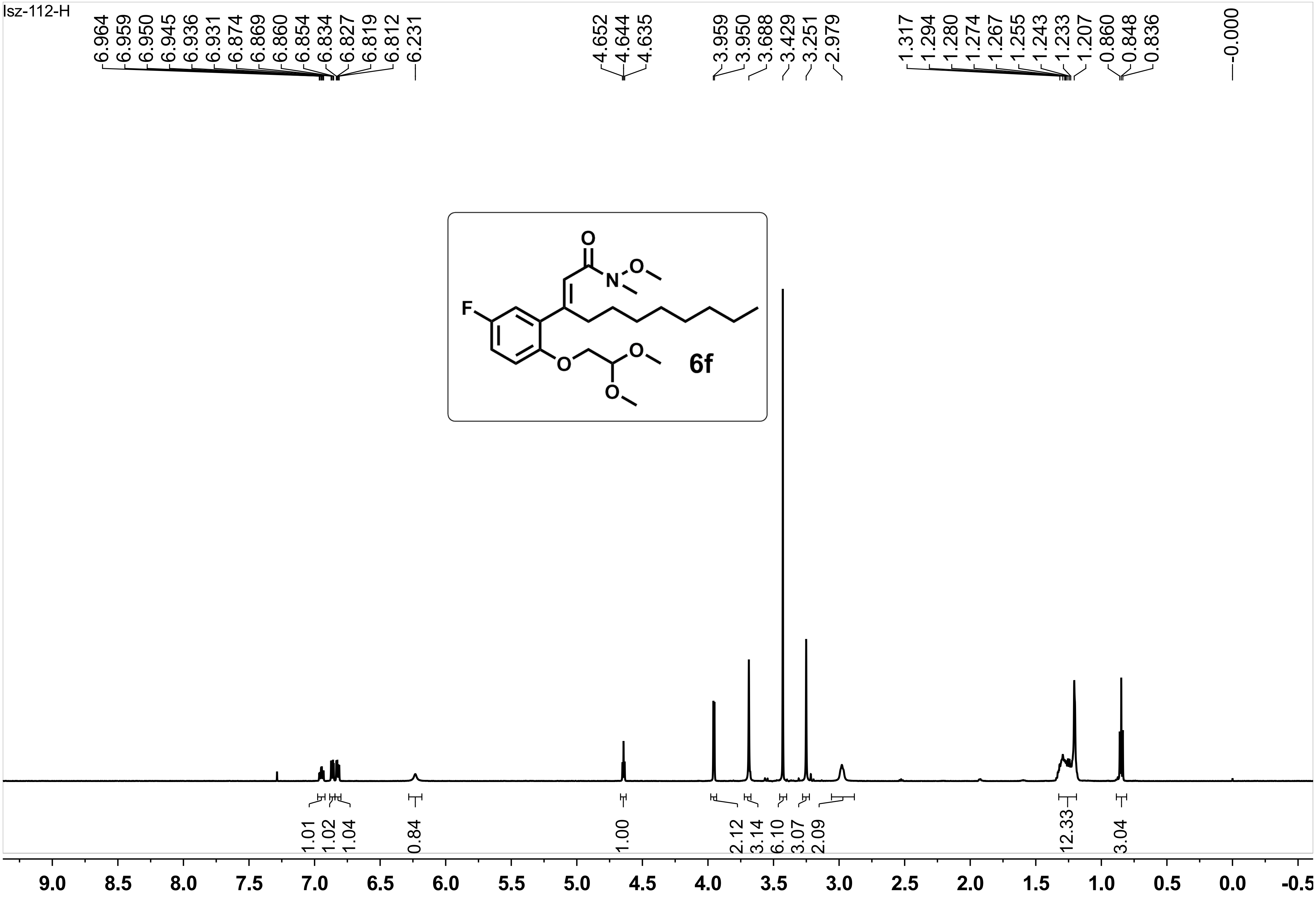

Supplementary Figure 46. ^{1}H NMR of **6f**

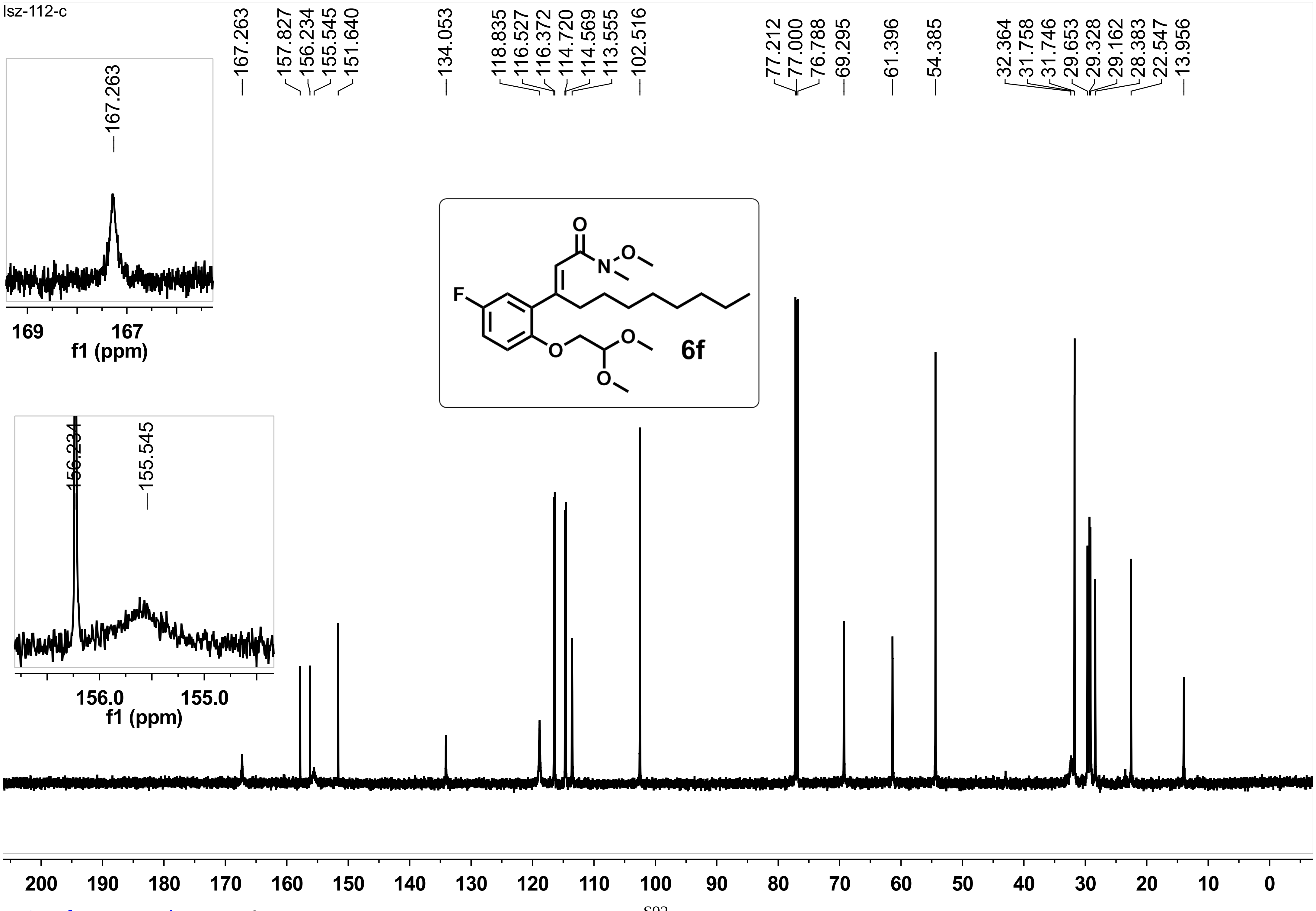

Supplementary Figure 47. ^{13}C NMR of **6f**

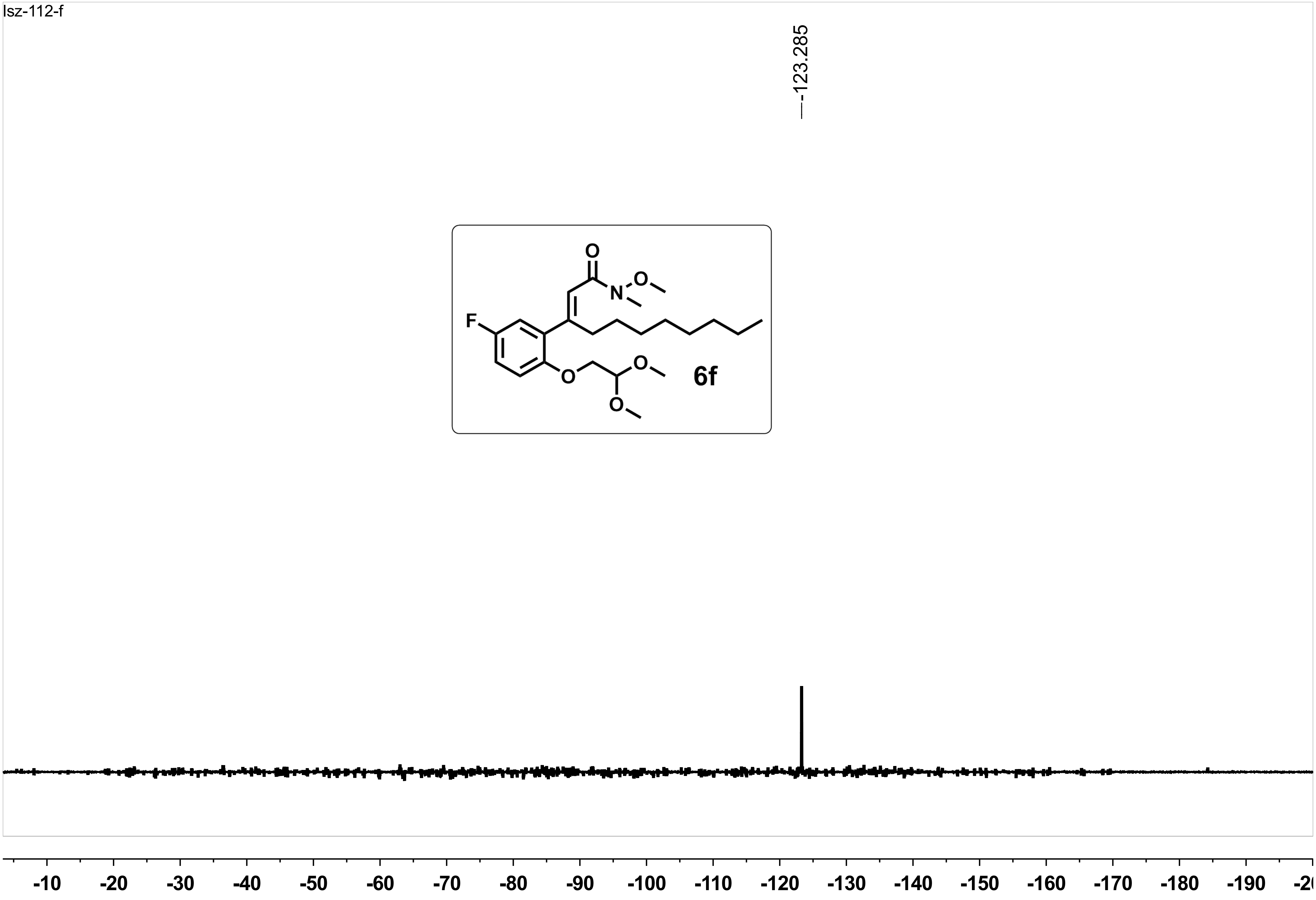

Supplementary Figure 48. ^{19}F NMR of **6f**

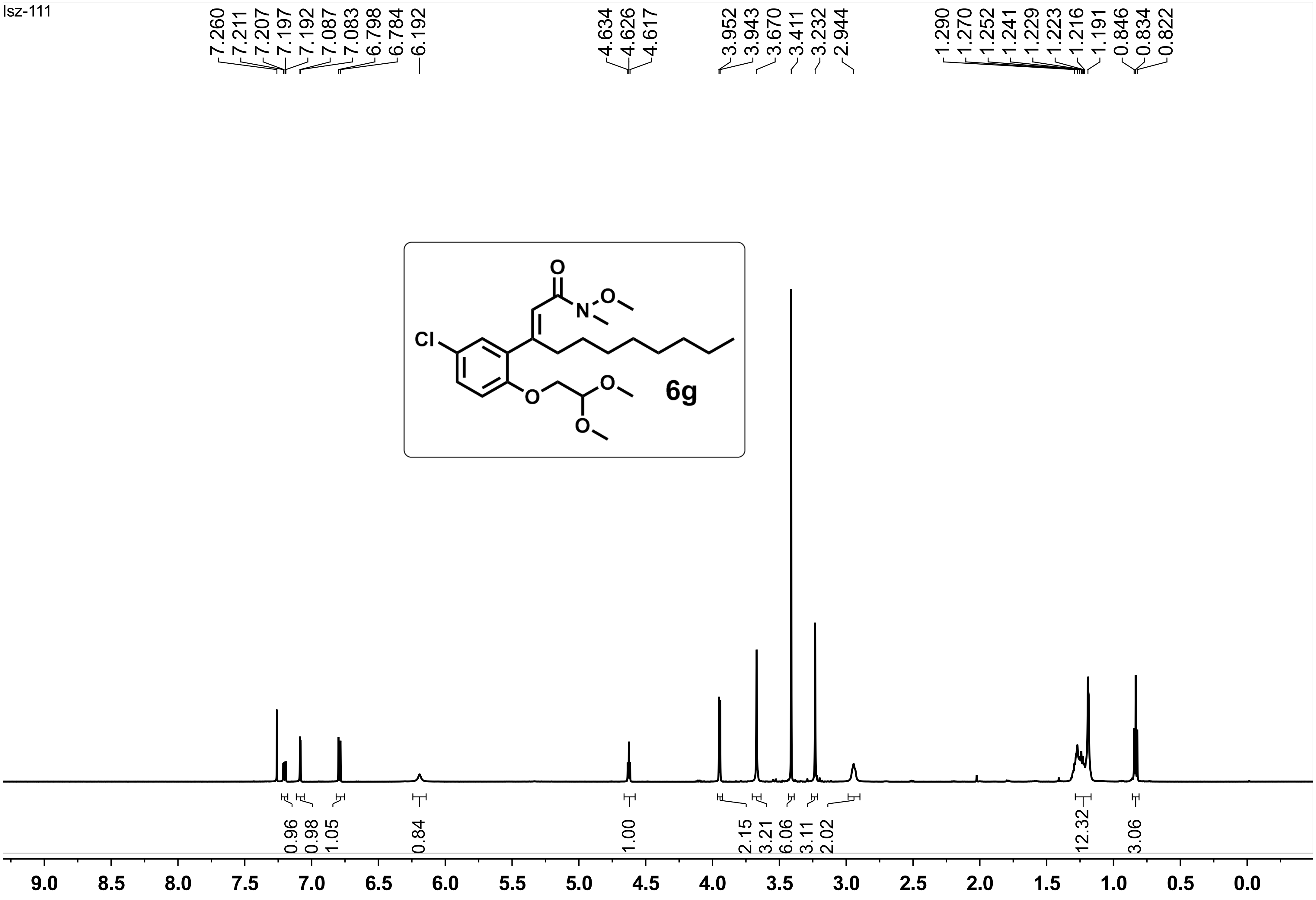

Supplementary Figure 49. ^{1}H NMR of **6g**

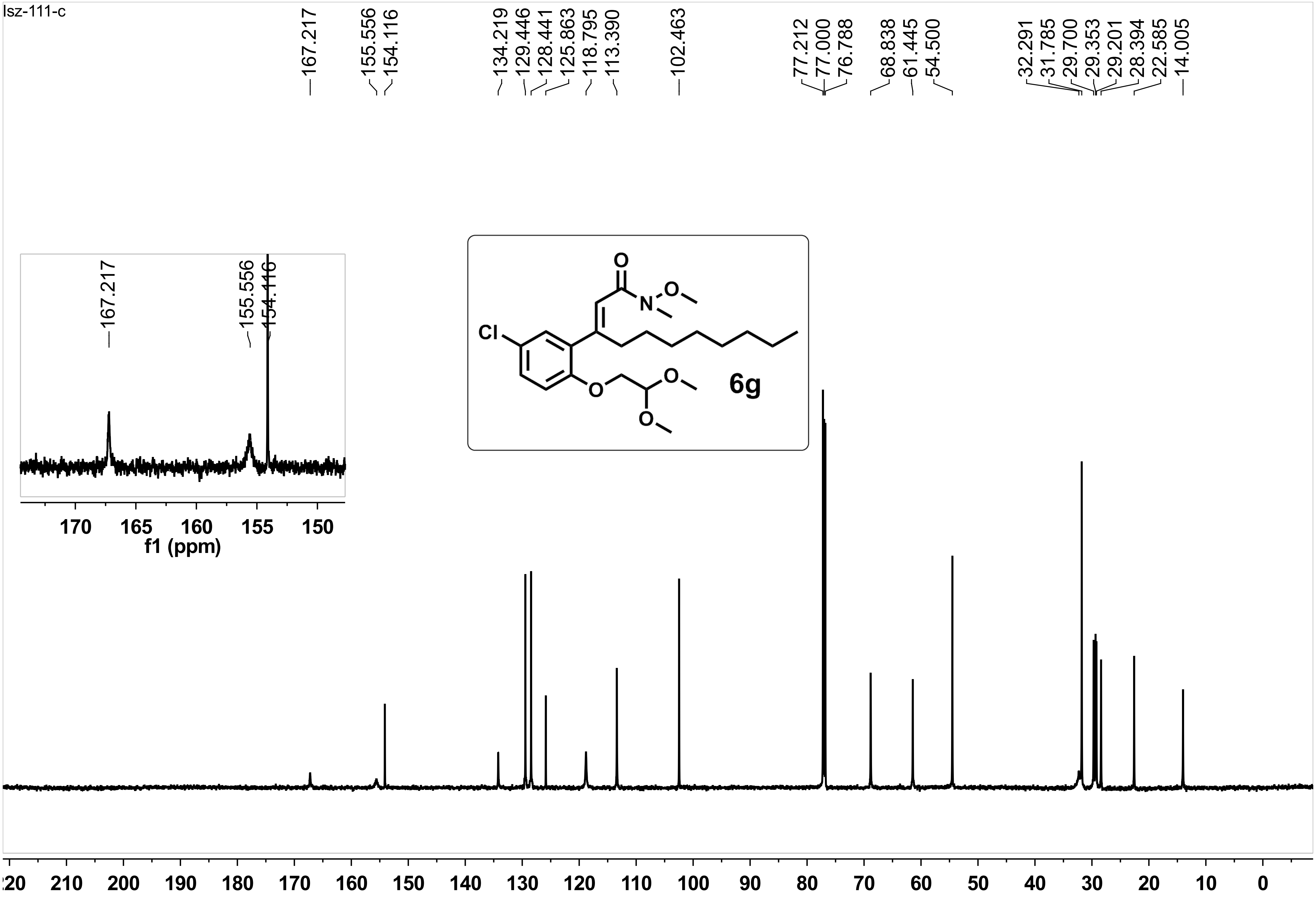

Supplementary Figure 50. ^{13}C NMR of **6g**

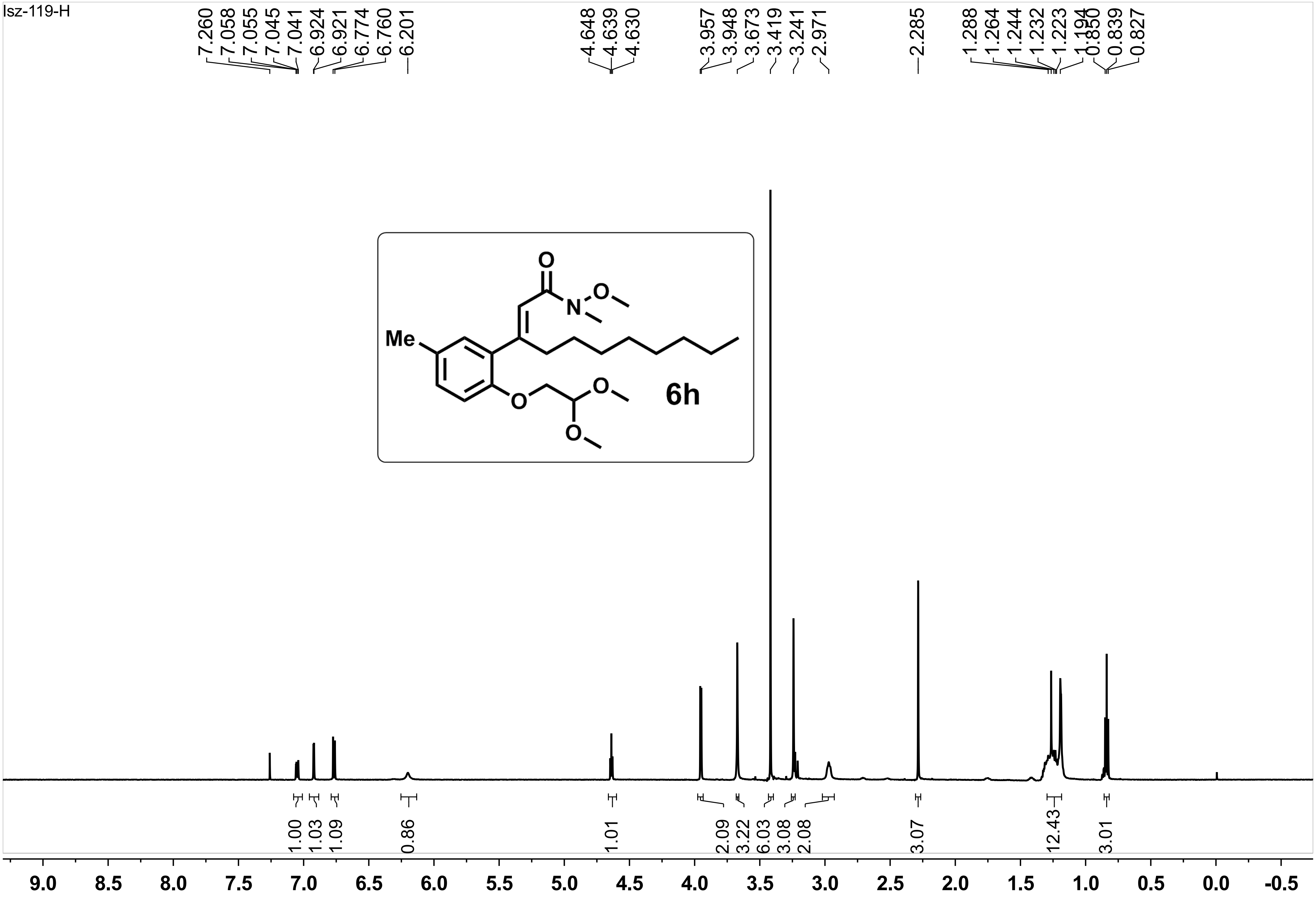

Supplementary Figure 51. ^{1}H NMR of **6h**

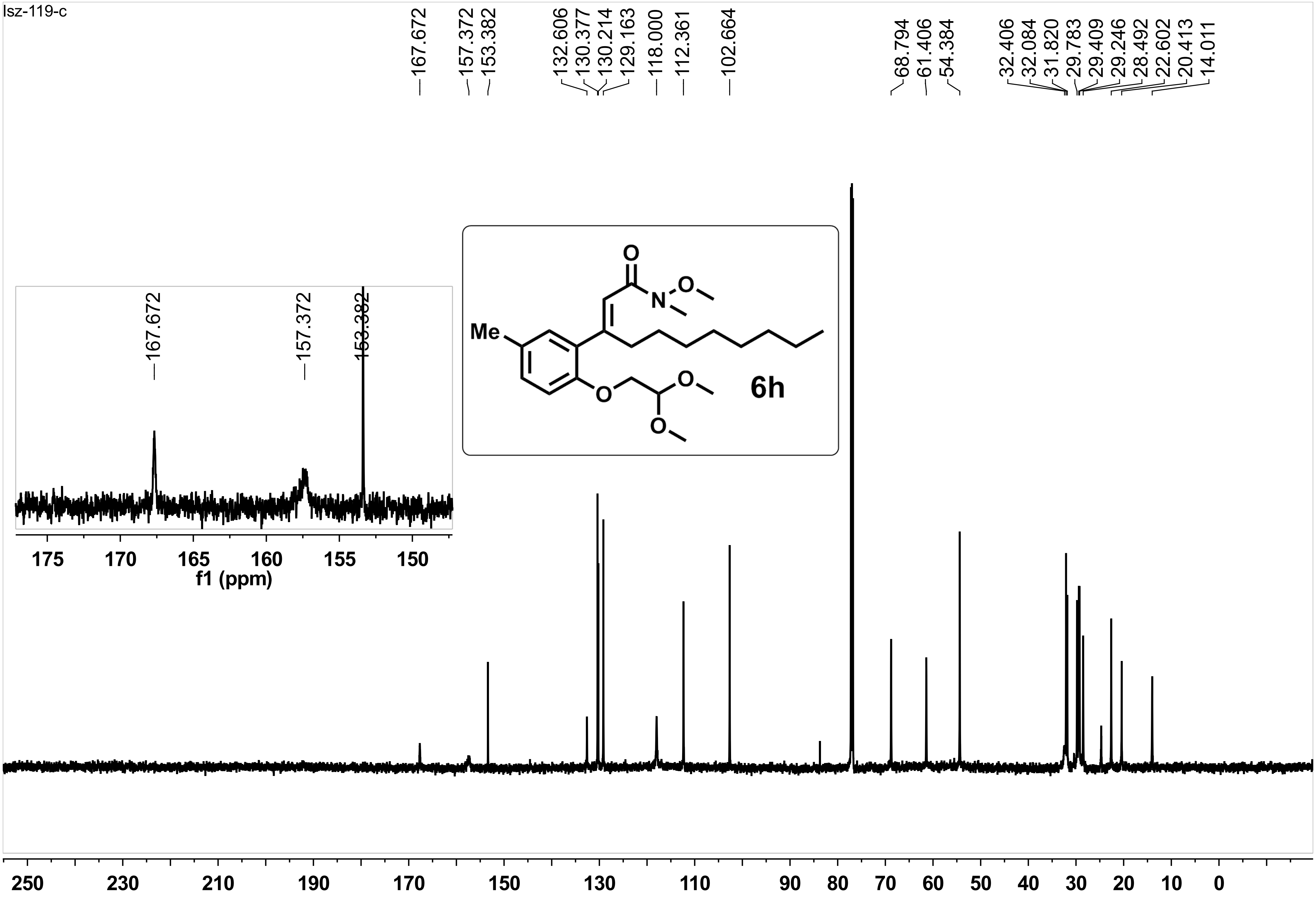

Supplementary Figure 52. ^{13}C NMR of **6h**

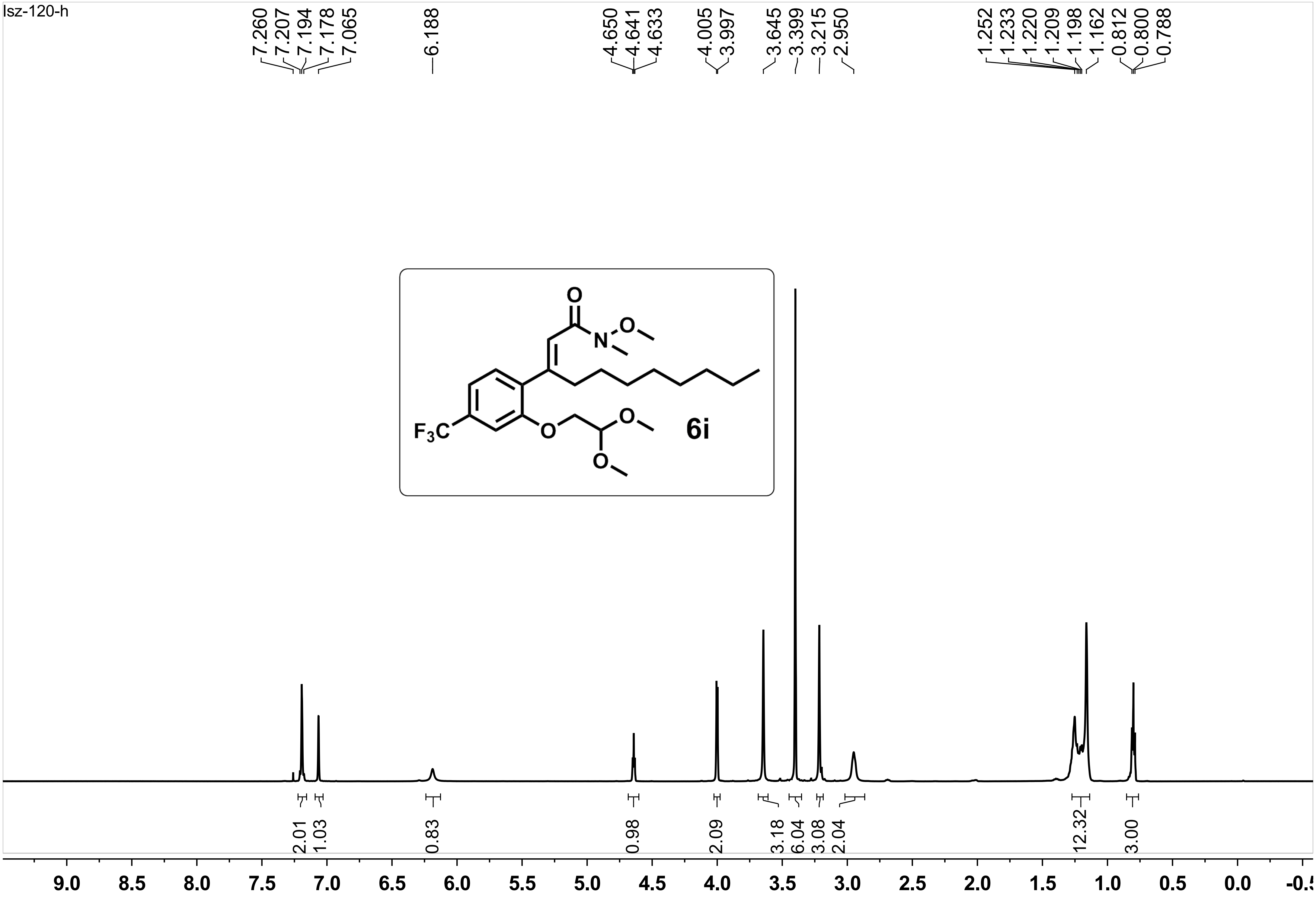

Supplementary Figure 53. ^{1}H NMR of **6i**

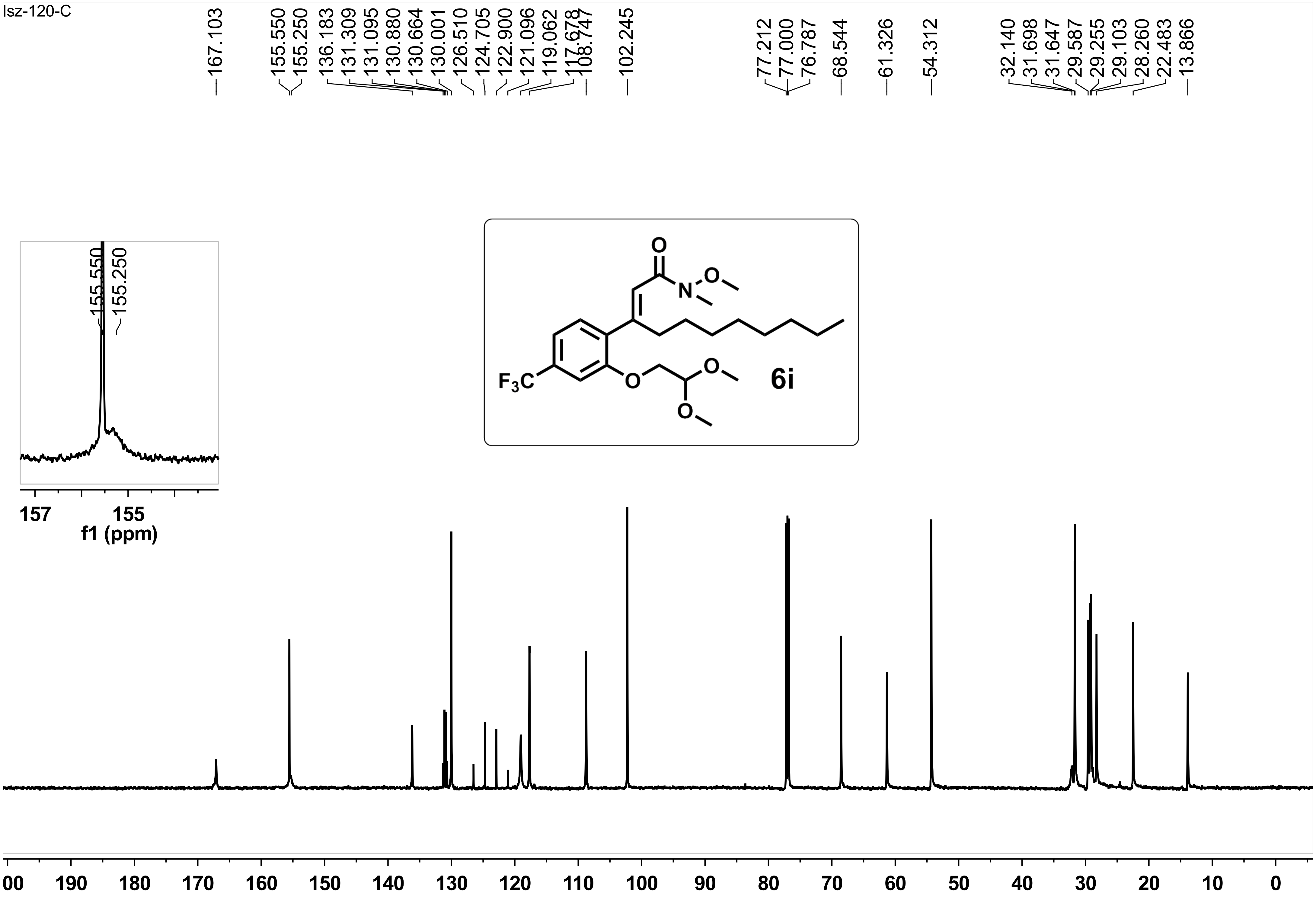

Supplementary Figure 54. ^{13}C NMR of **6i**

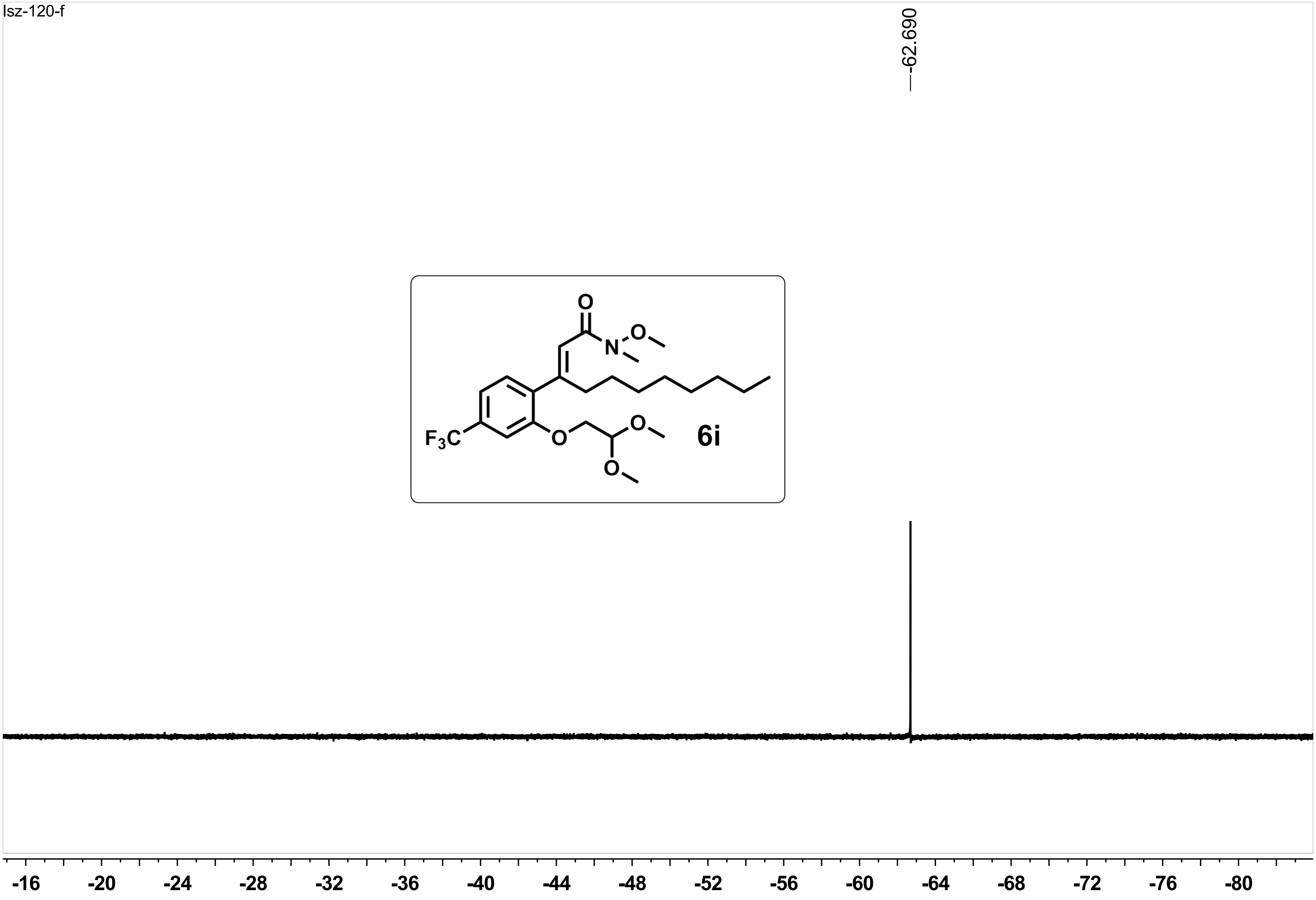

Supplementary Figure 55. ^{19}F NMR of **6i**

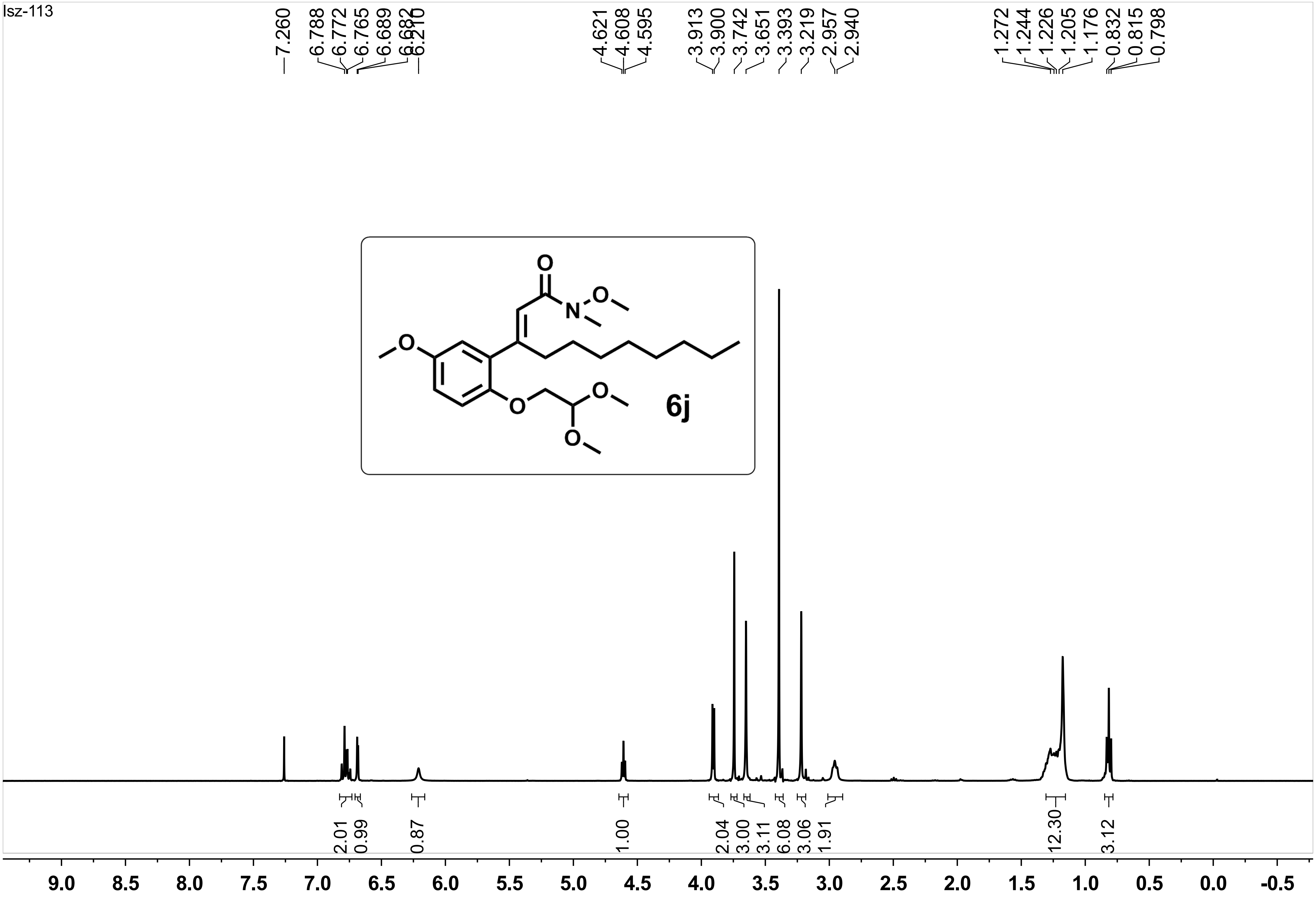

Supplementary Figure 56. ^{1}H NMR of **6j**

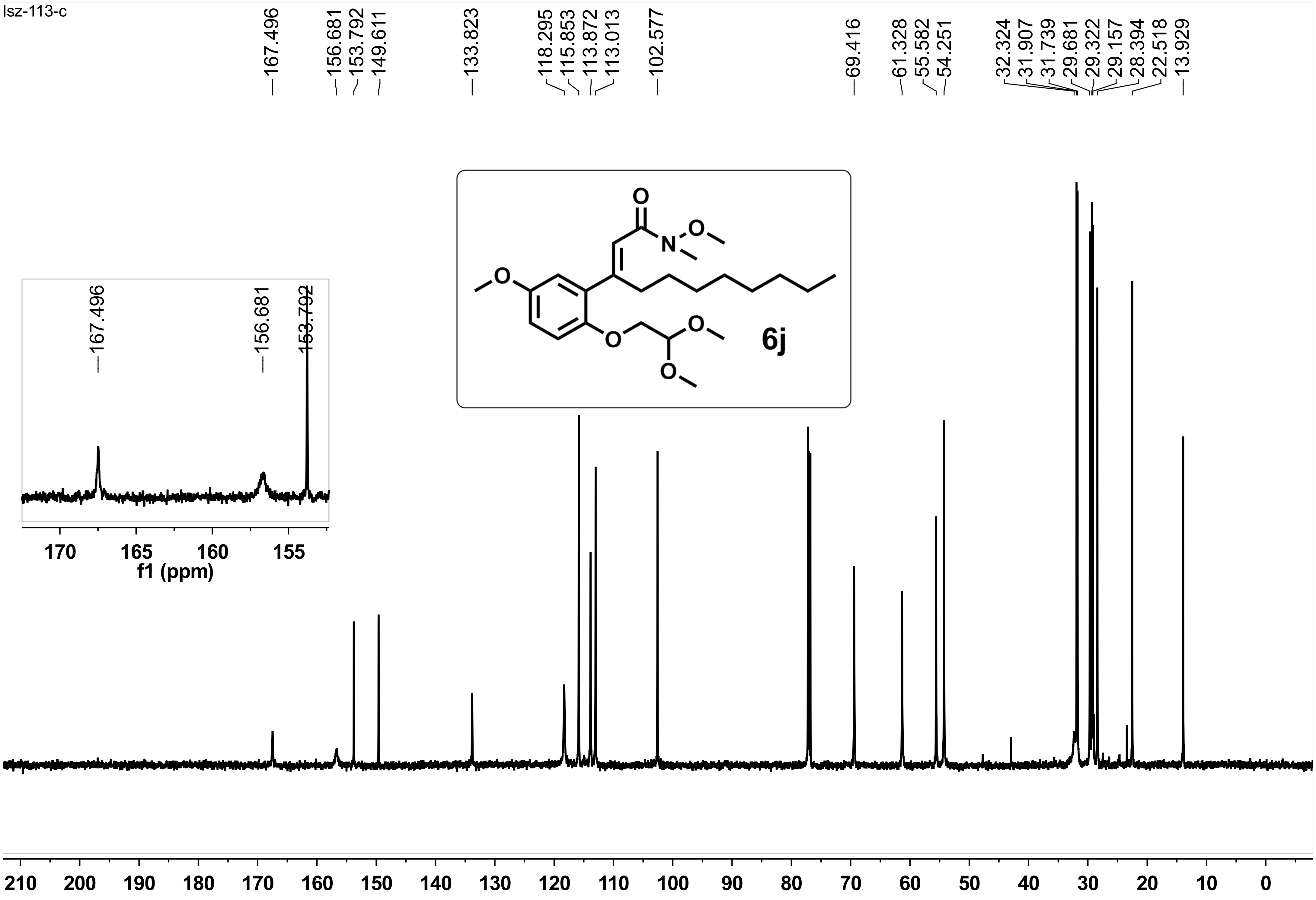

Supplementary Figure 57. ^{13}C NMR of **6j**

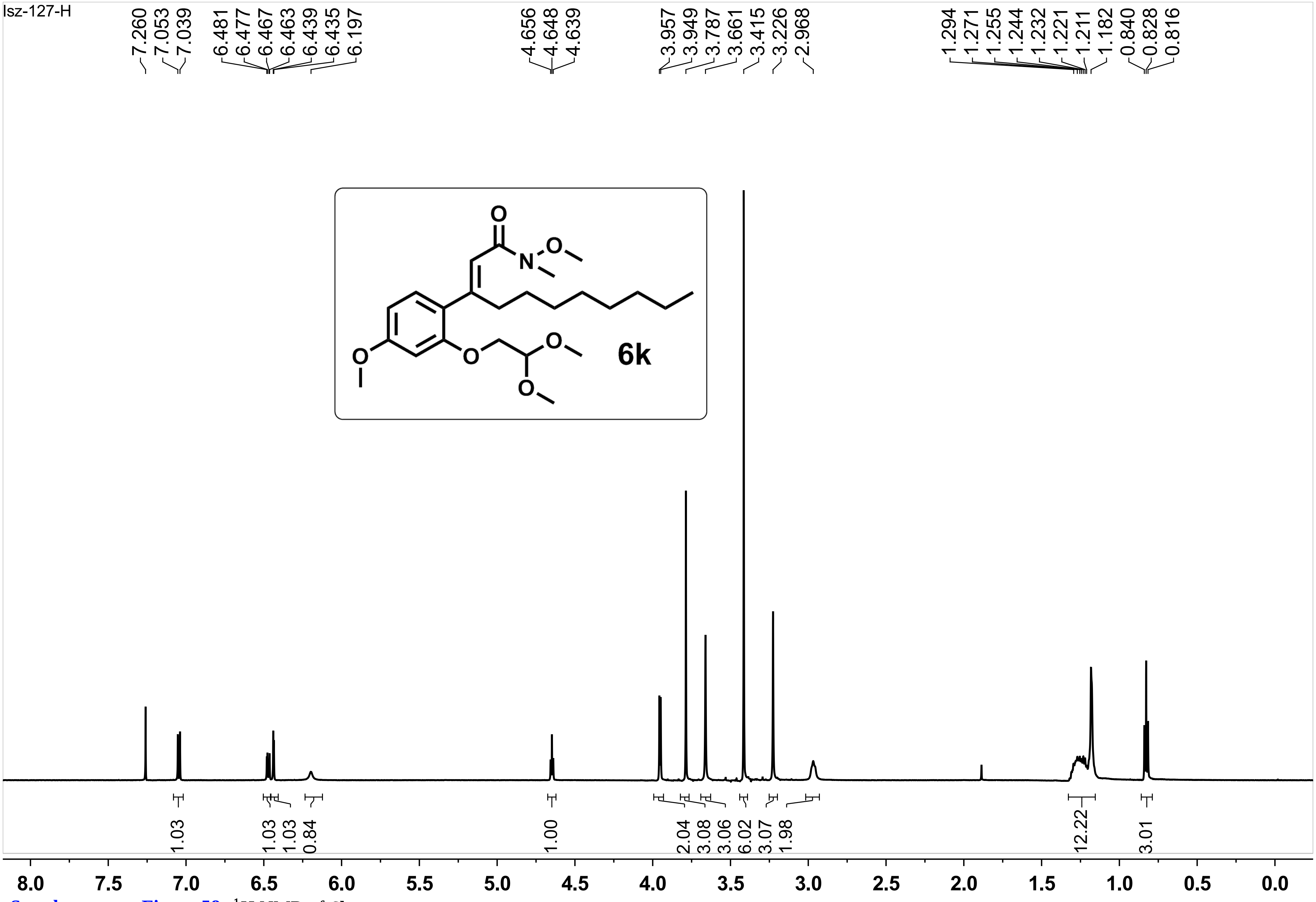

Supplementary Figure 58. ^{1}H NMR of **6k**

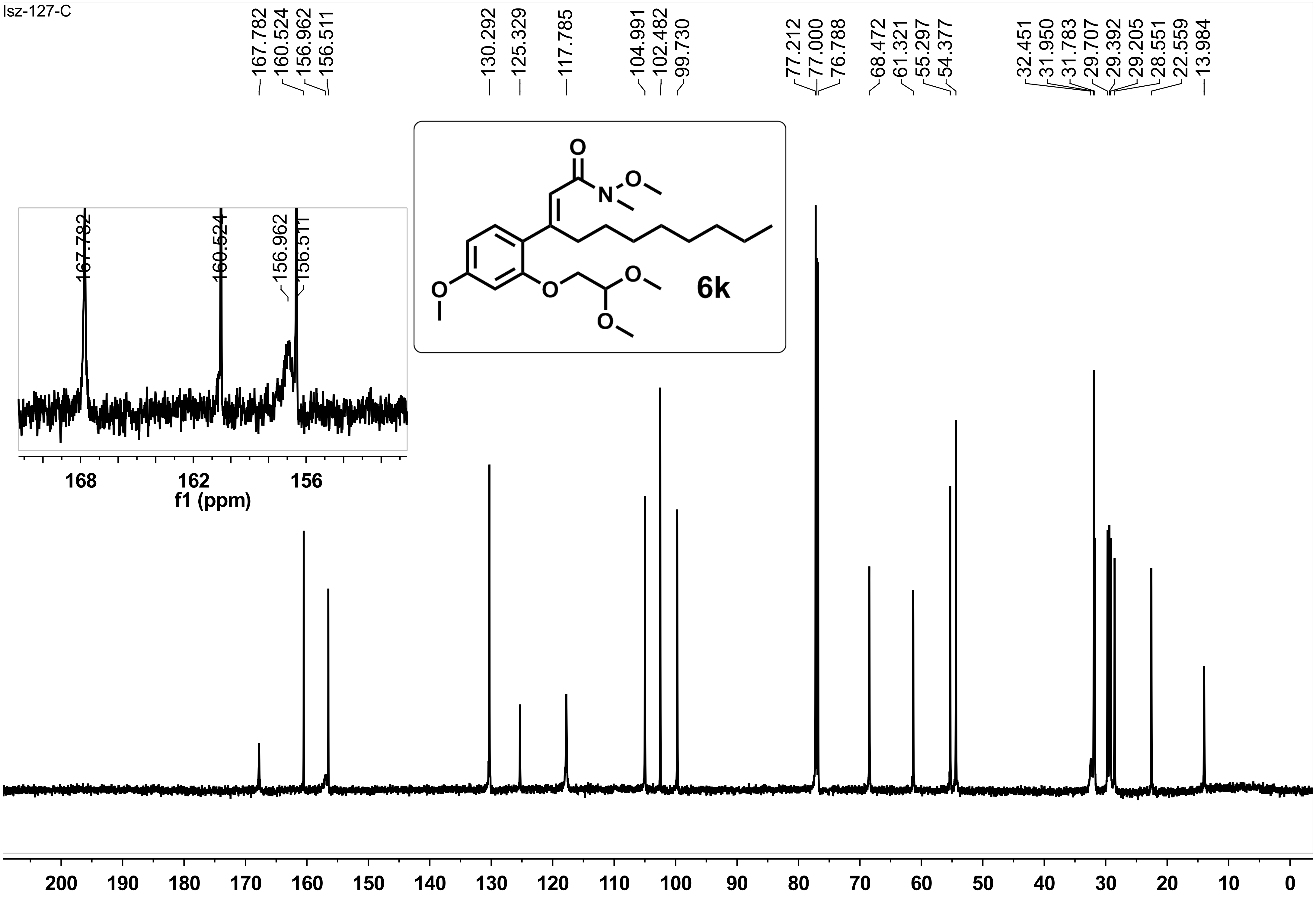

Supplementary Figure 59. ^{13}C NMR of **6k**

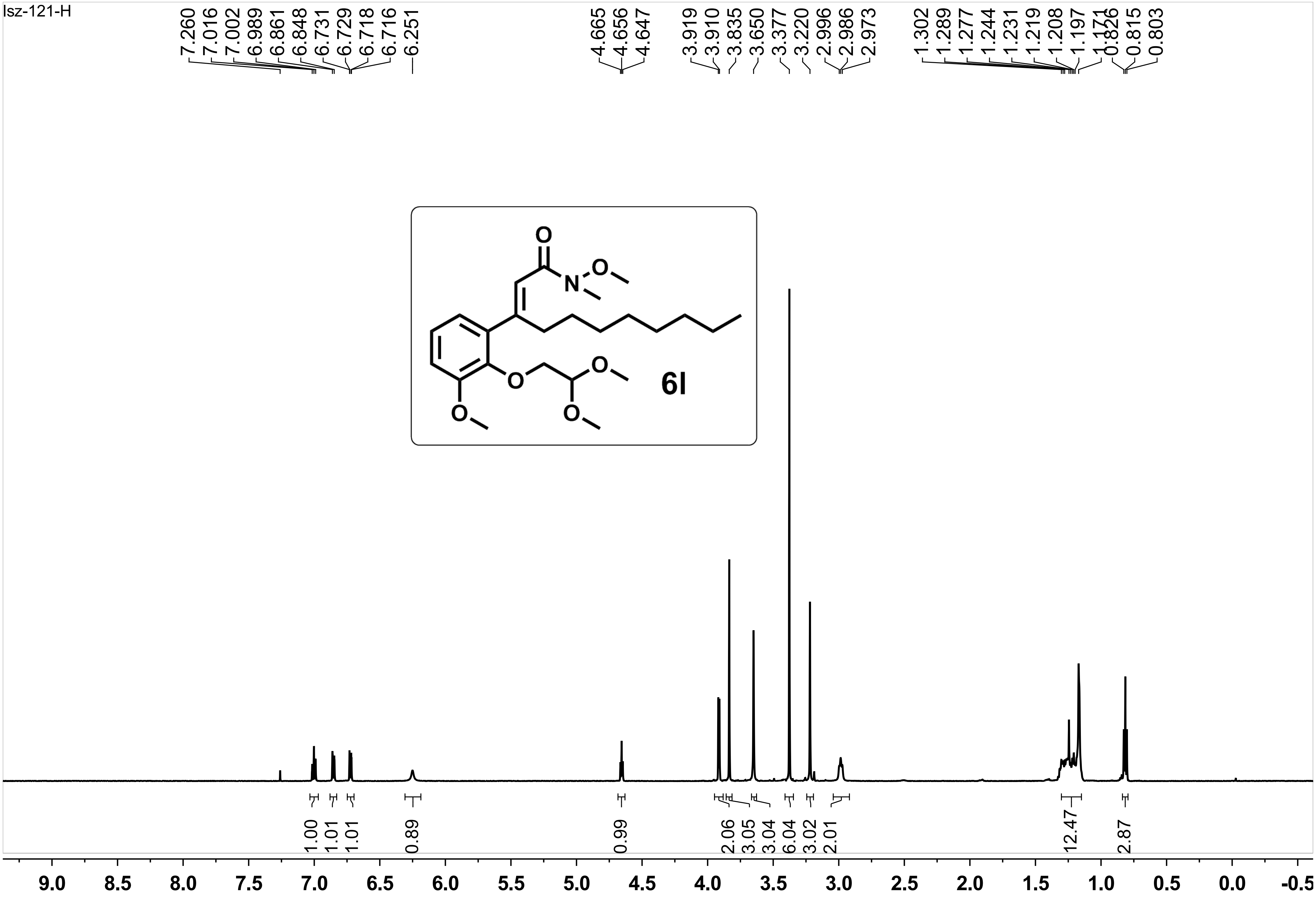

Supplementary Figure 60. ^{1}H NMR of **6l**

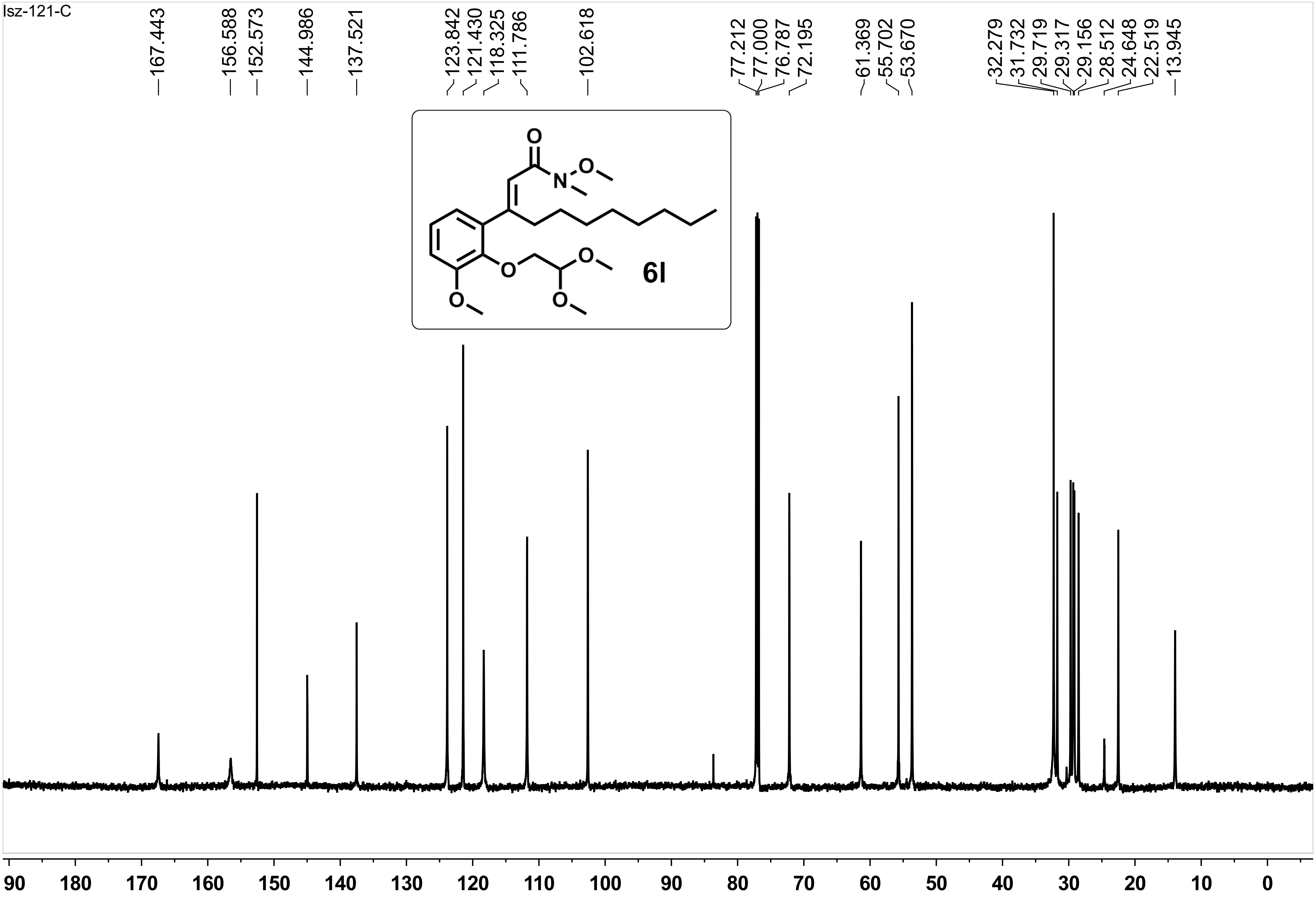

Supplementary Figure 61. ^{13}C NMR of **6l**

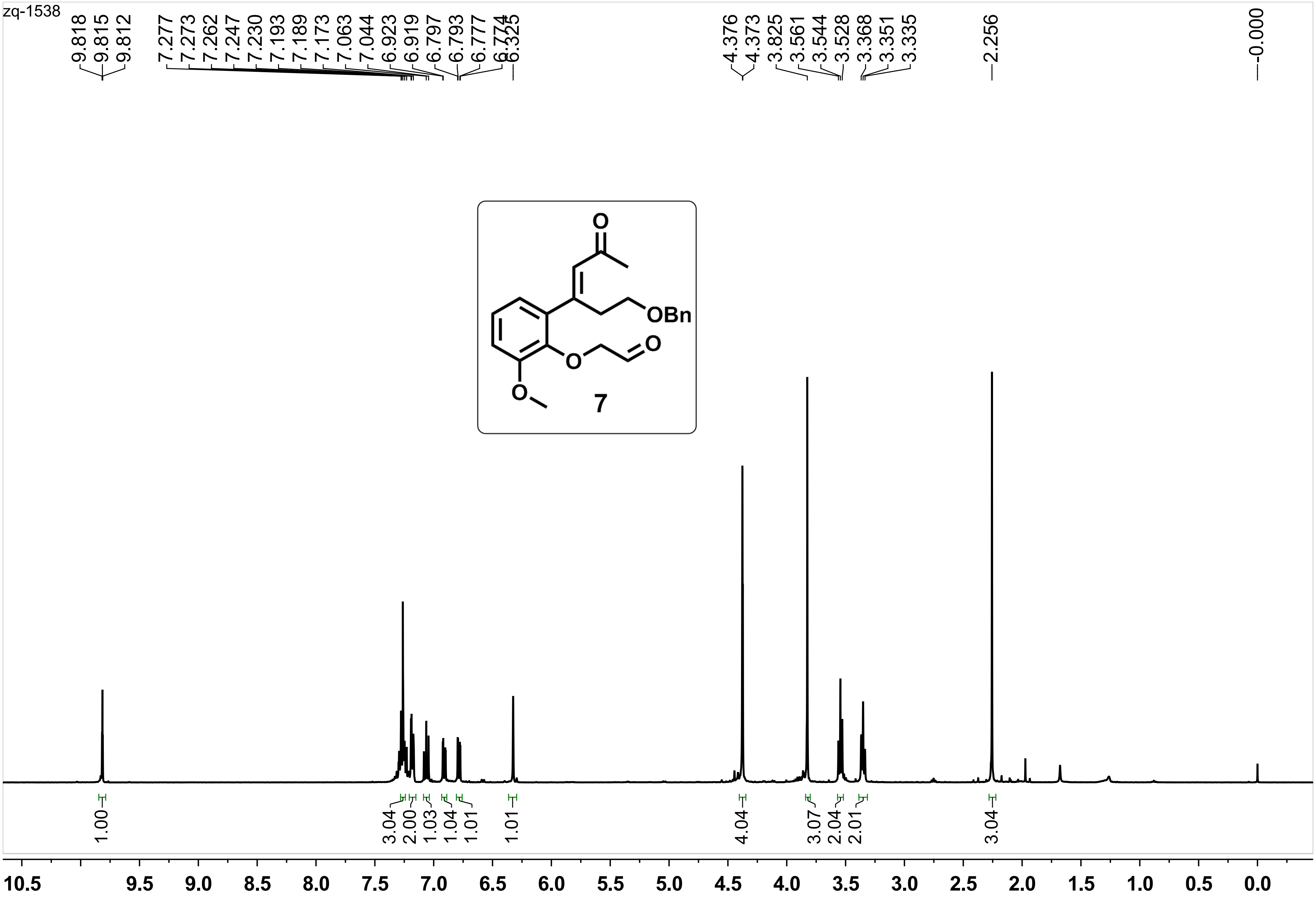

Supplementary Figure 62. ^{1}H NMR of **7**

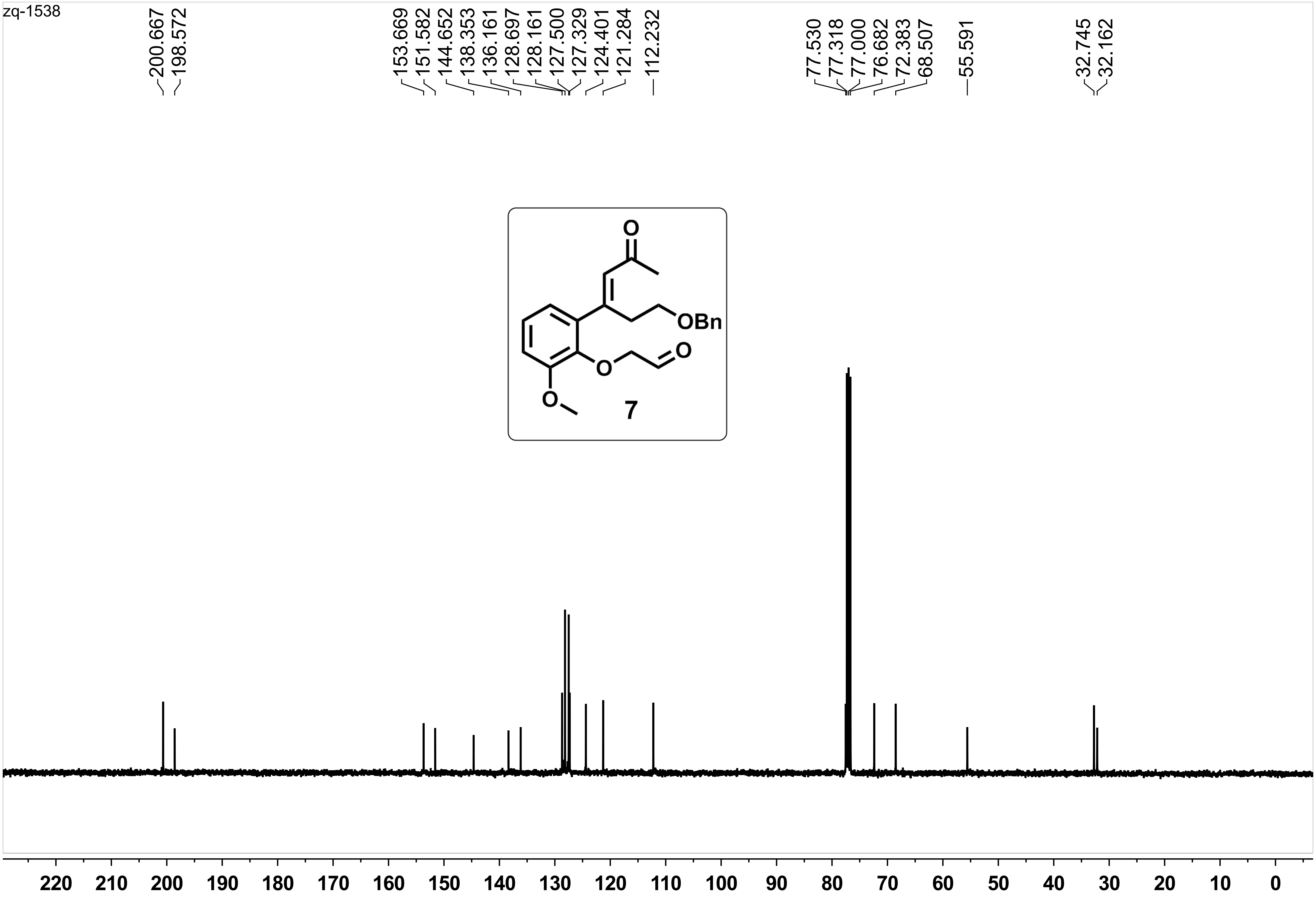

Supplementary Figure 63. ^{13}C NMR of **7**

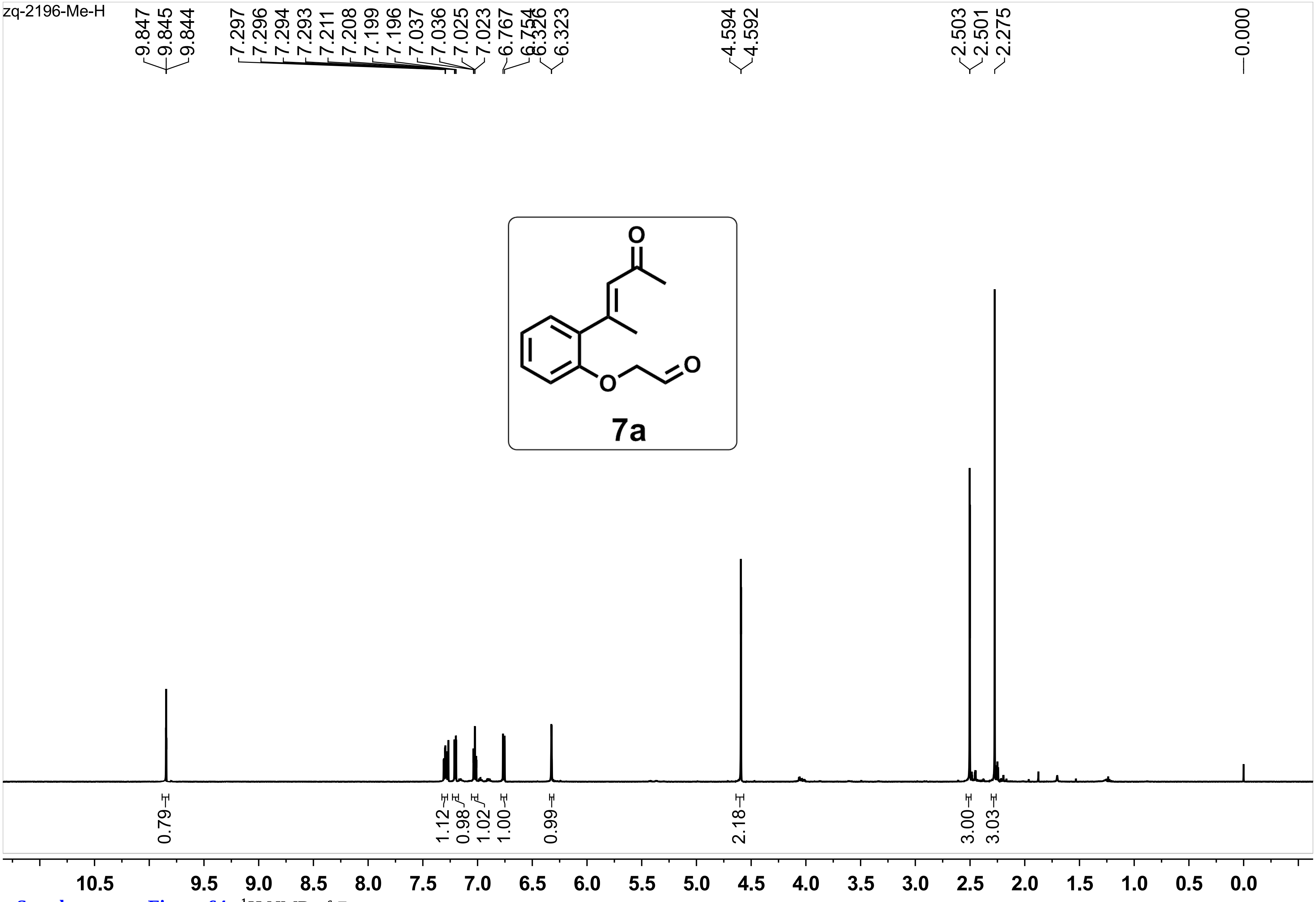

Supplementary Figure 64. ^{1}H NMR of **7a**

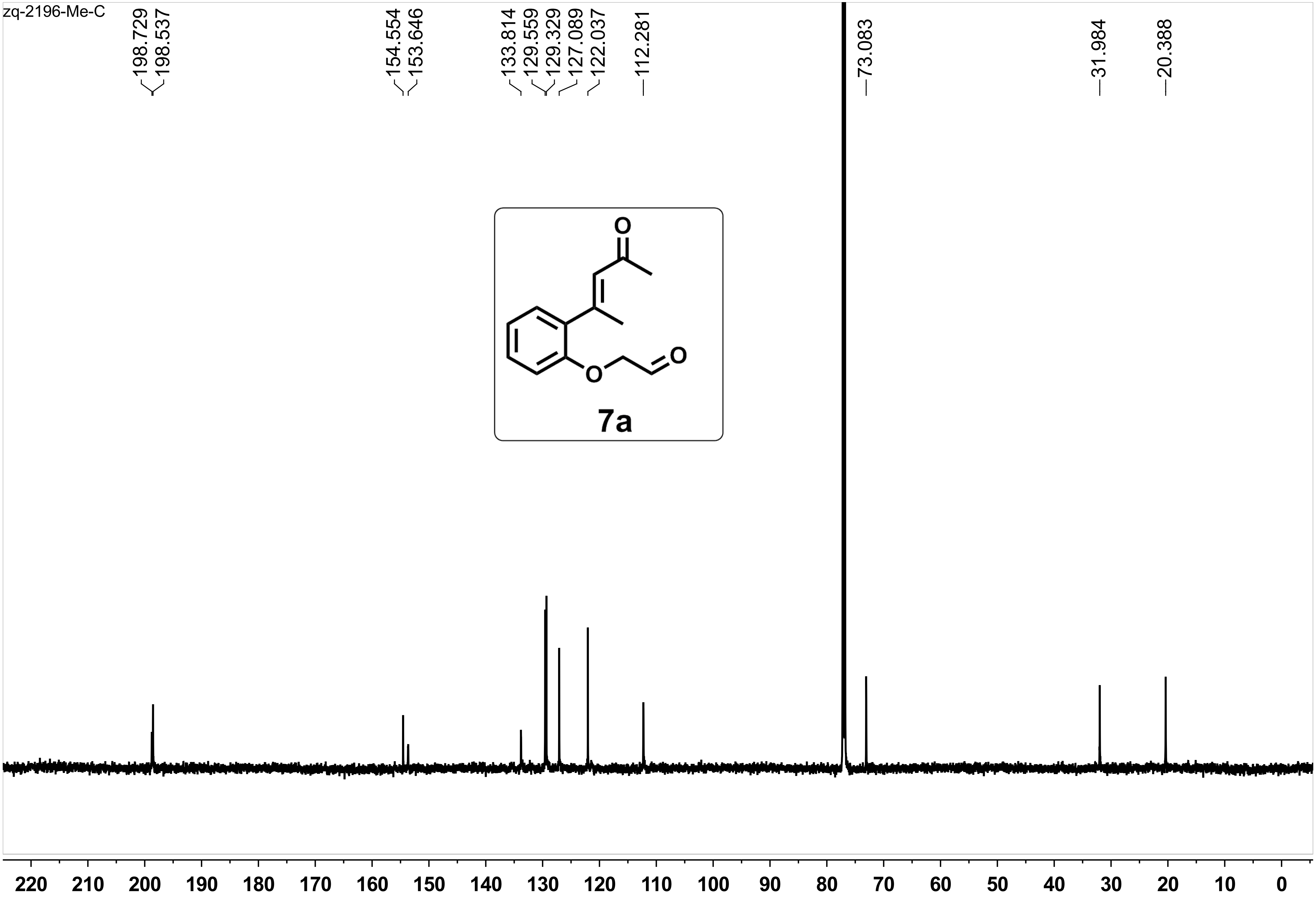

Supplementary Figure 65. ^{13}C NMR of **7a**

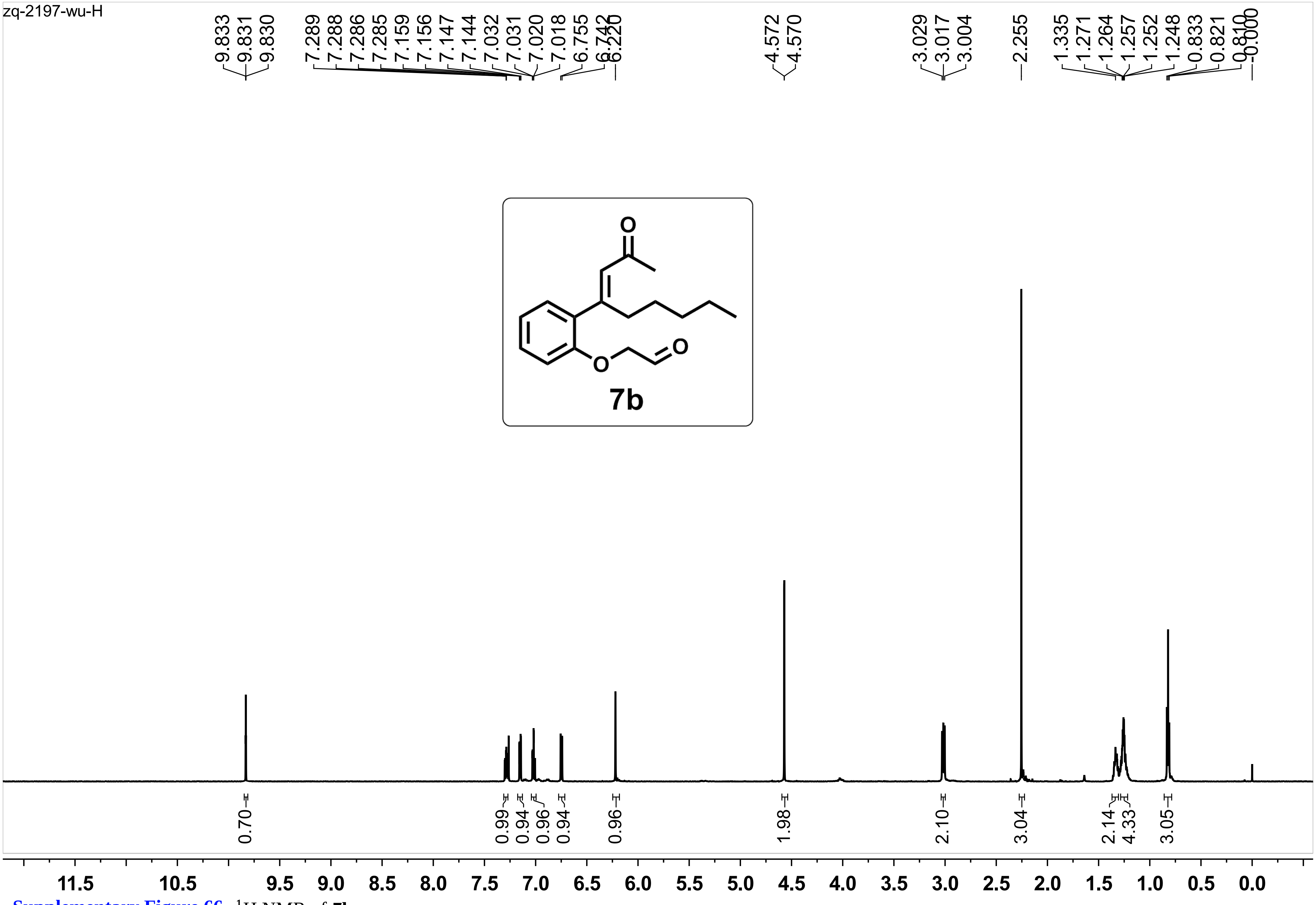

Supplementary Figure 66. ^{1}H NMR of **7b**

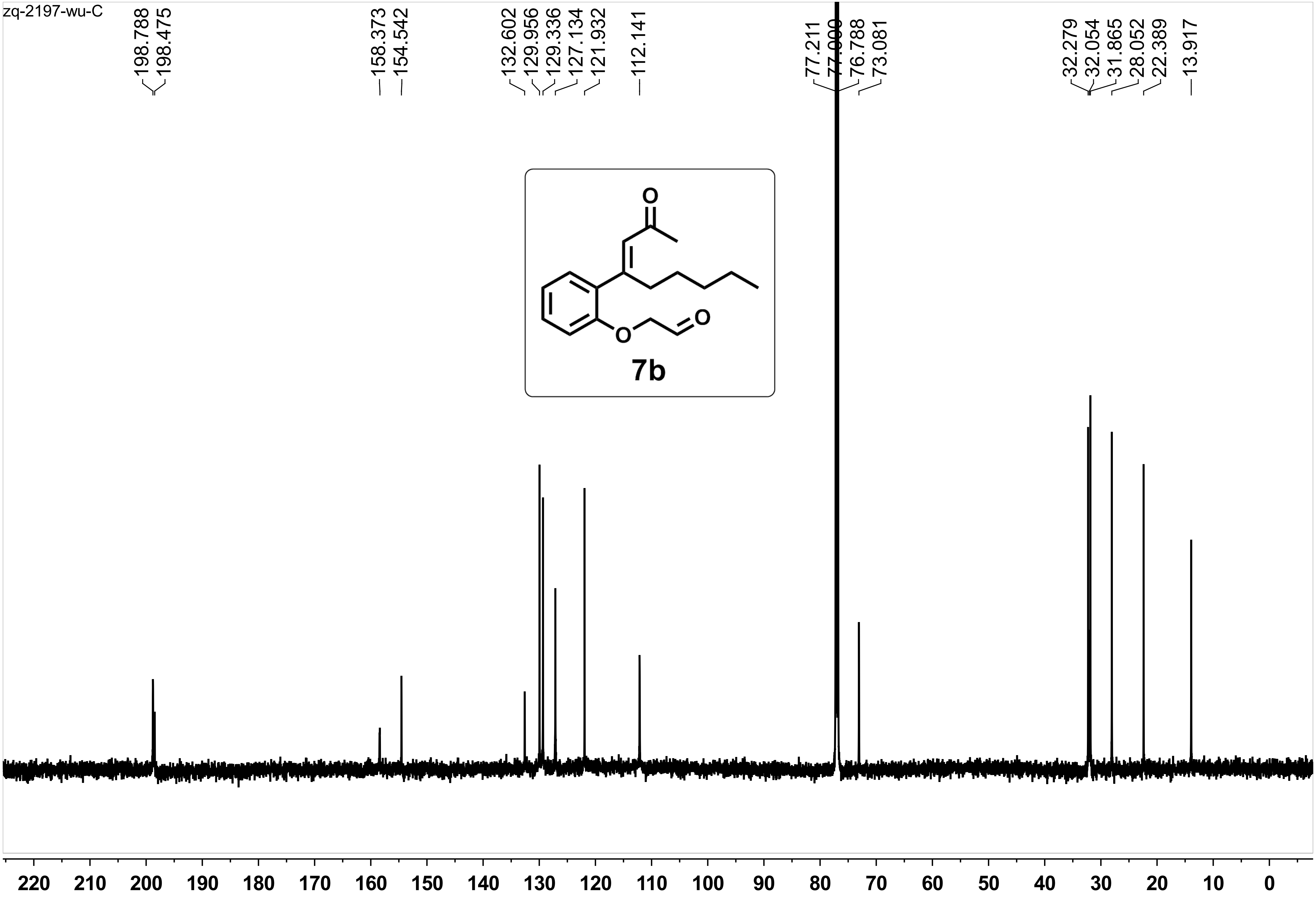

Supplementary Figure 67. ^{13}C NMR of **7b**

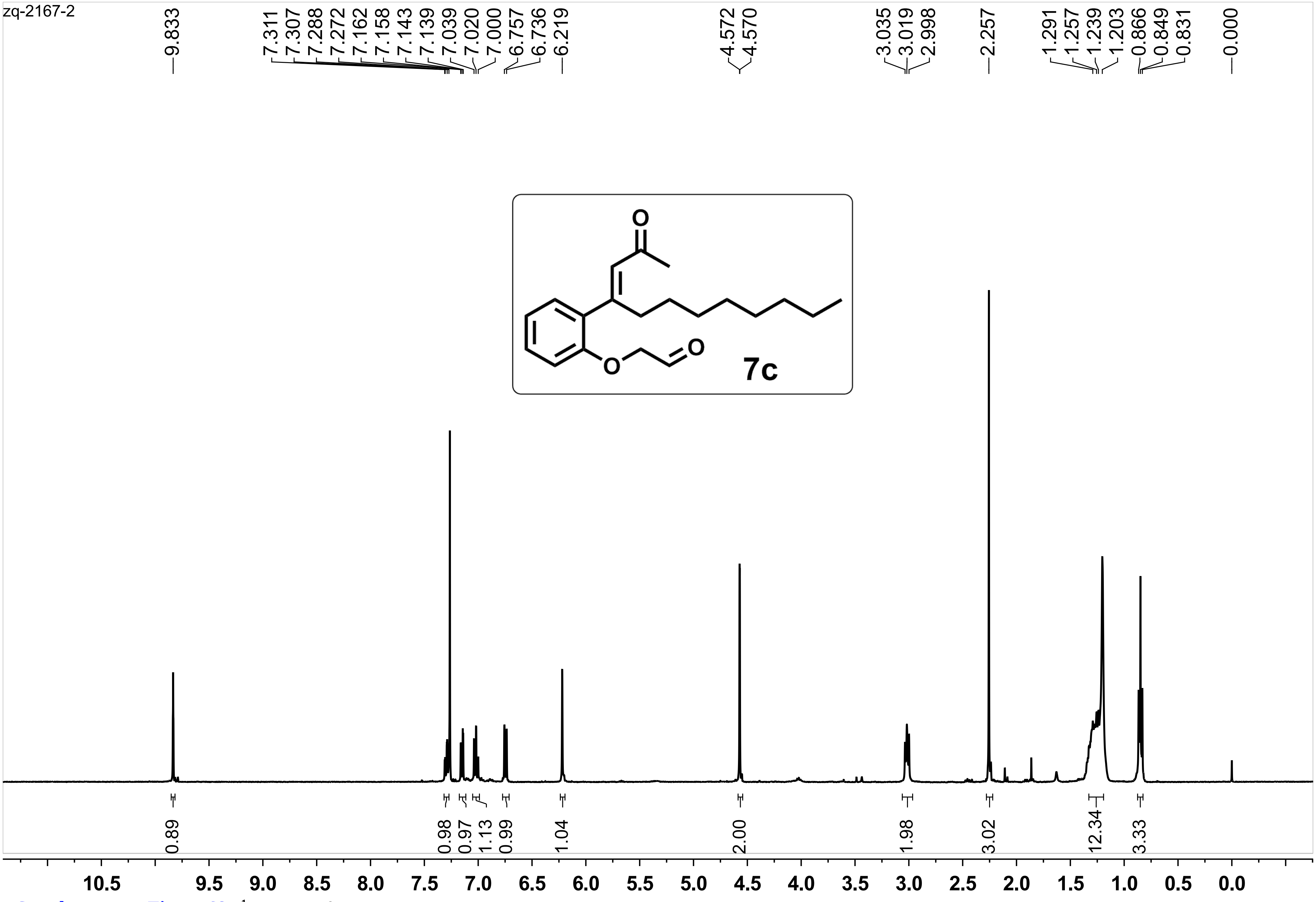

Supplementary Figure 68. ^{1}H NMR of **7c**

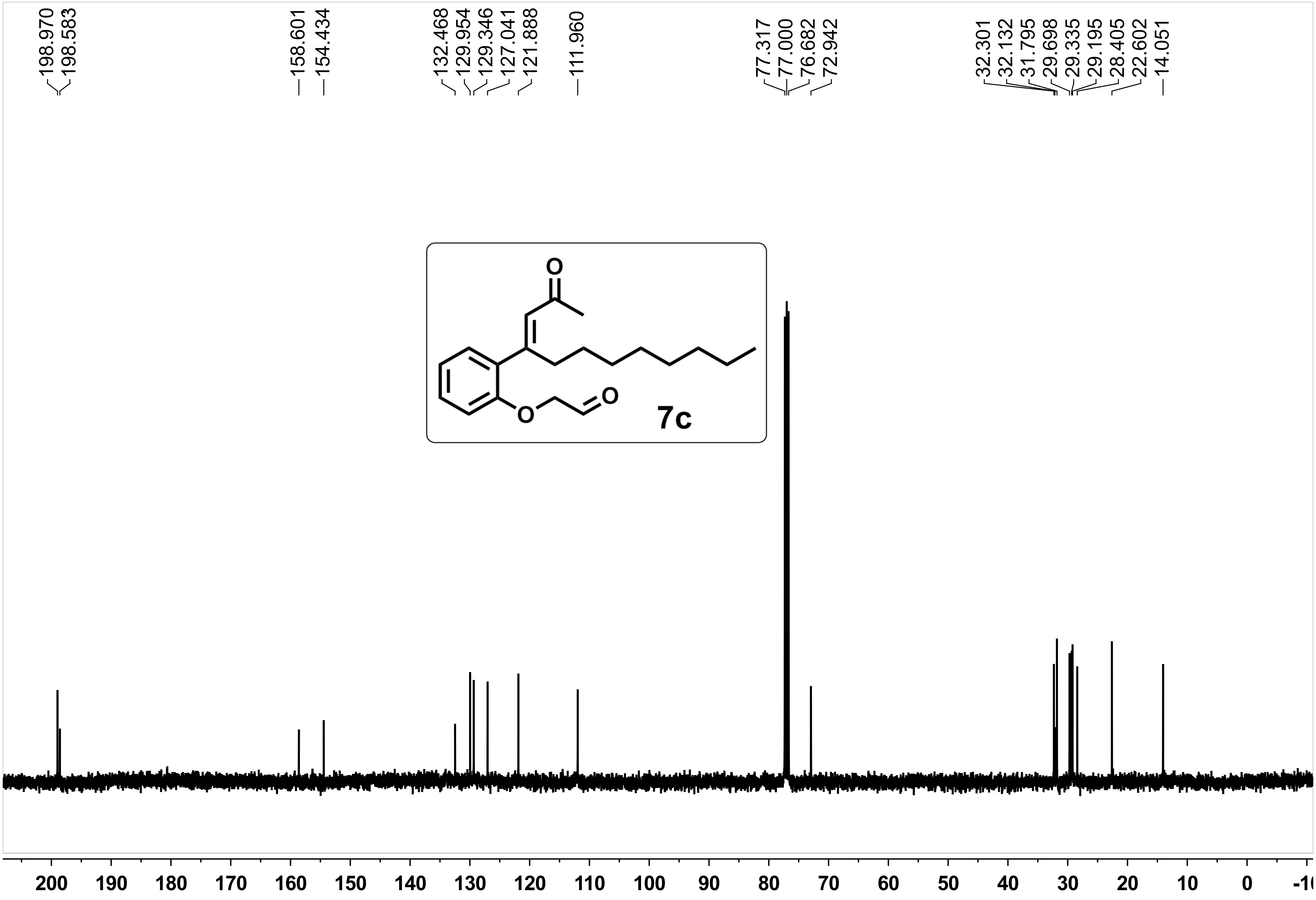

Supplementary Figure 69. ^{13}C NMR of **7c**

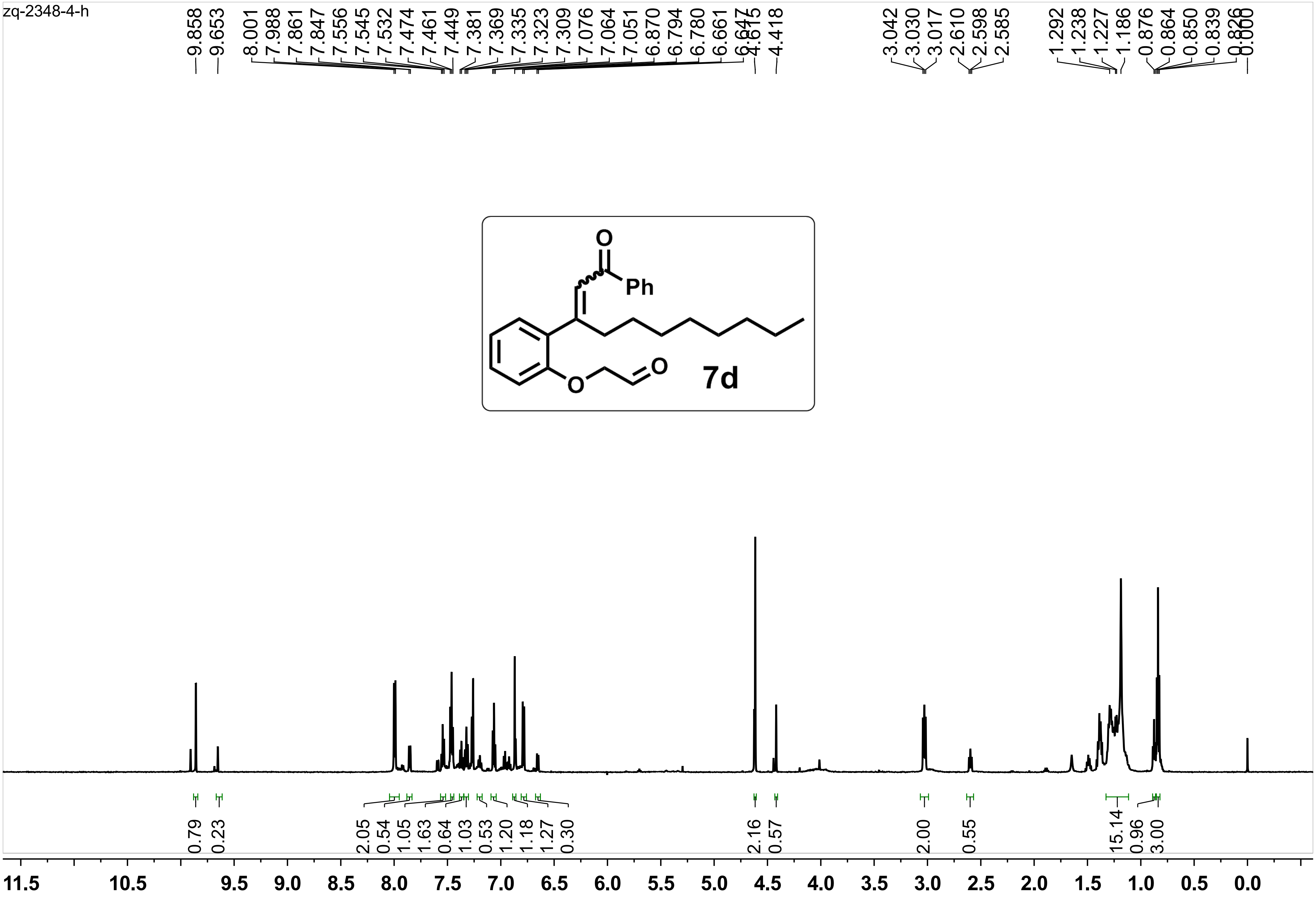

Supplementary Figure 70. ^{1}H NMR of **7d**

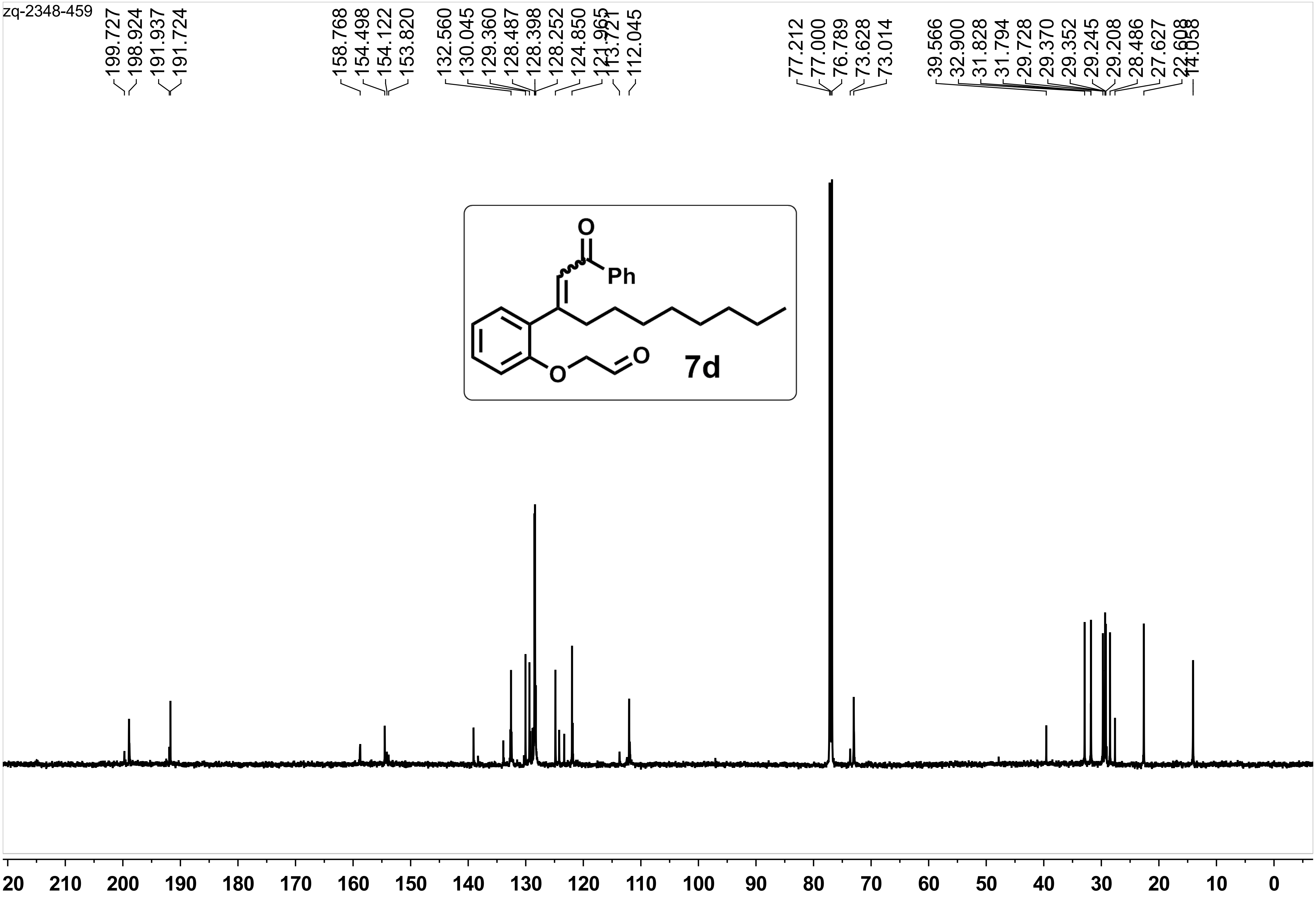

Supplementary Figure 71. ^{13}C NMR of **7d**

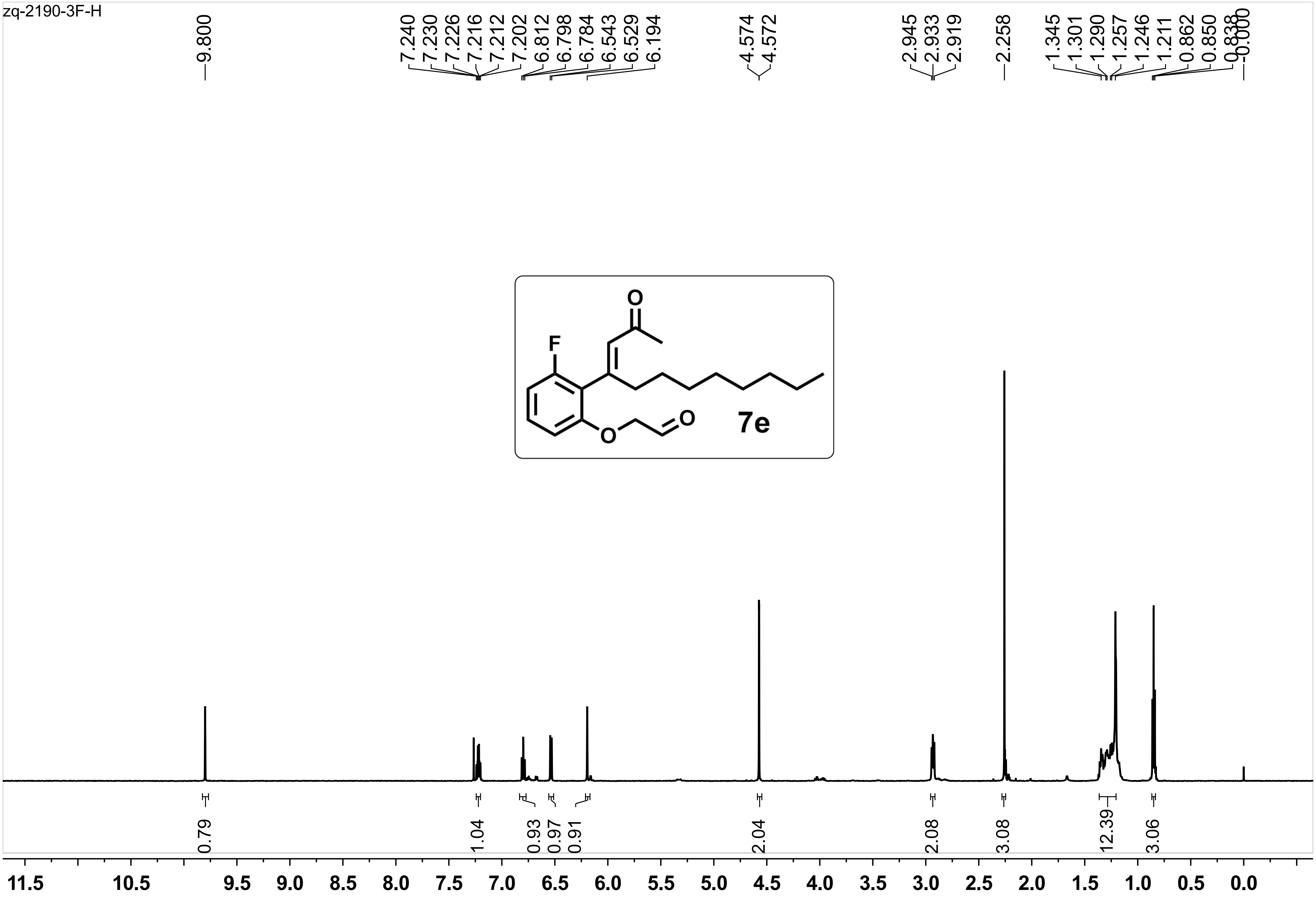

Supplementary Figure 72. ^{1}H NMR of **7e**

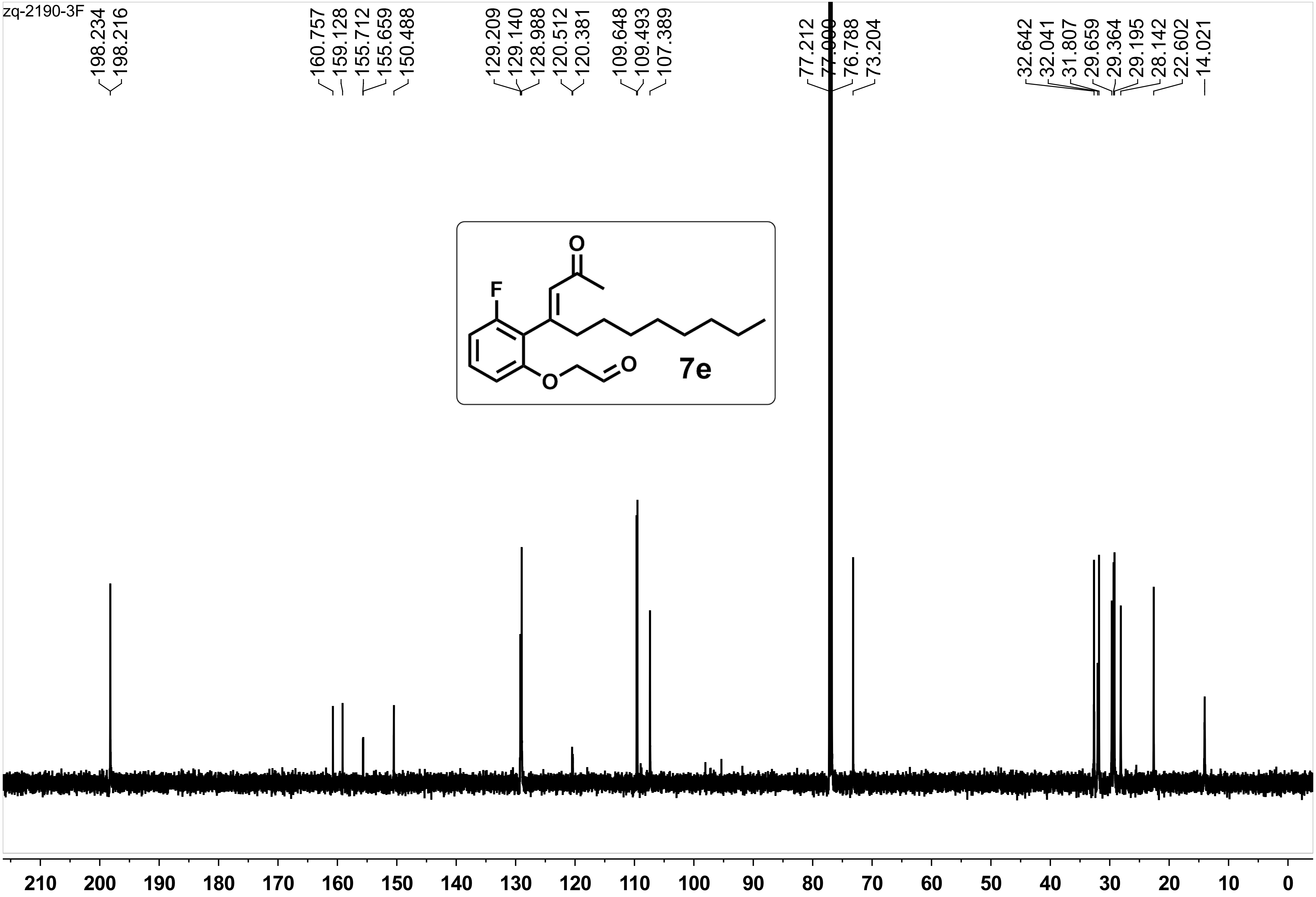

Supplementary Figure 73. ^{13}C NMR of **7e**

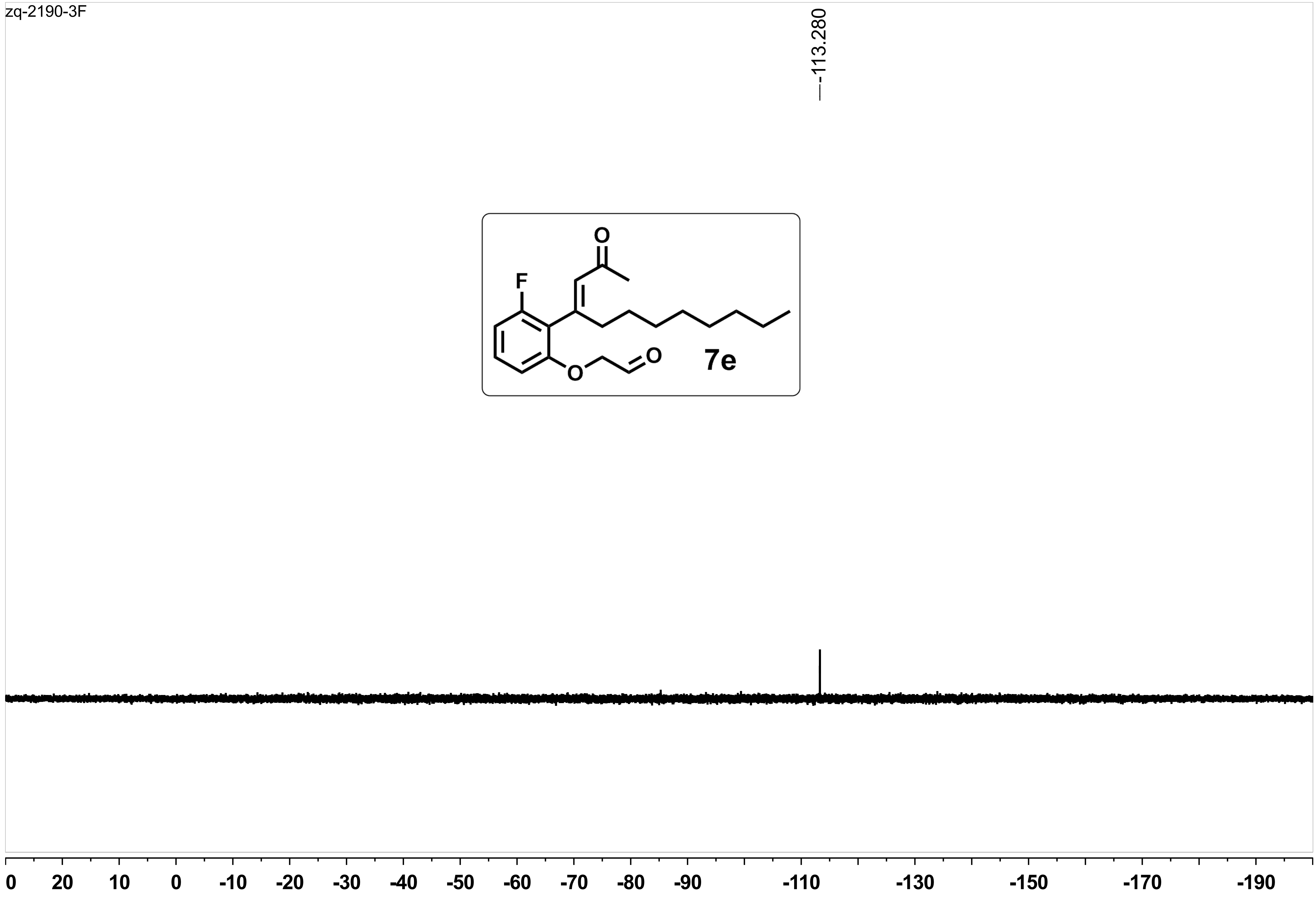

Supplementary Figure 74. ^{19}F NMR of **7e**

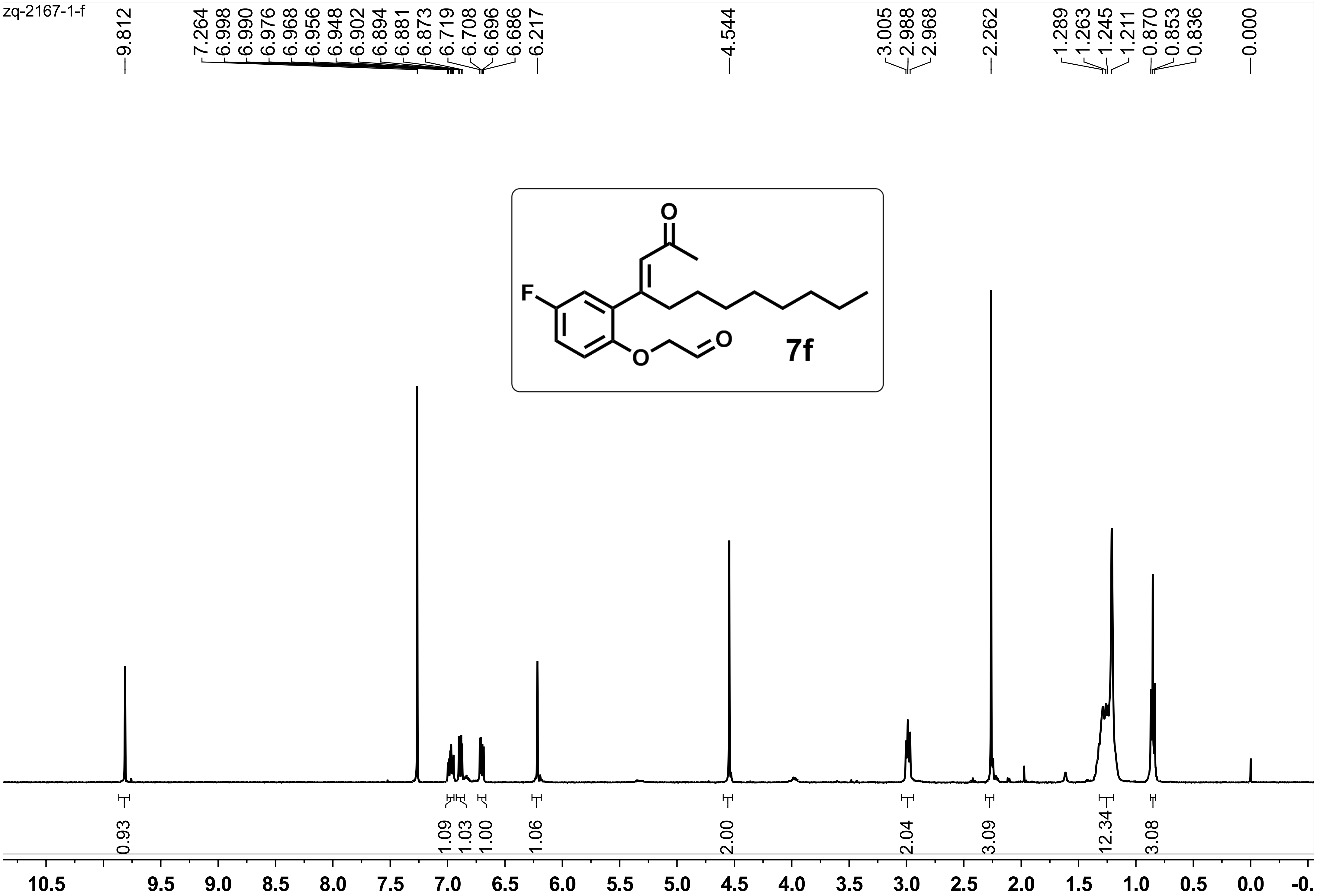

Supplementary Figure 75. ^{1}H NMR of **7f**

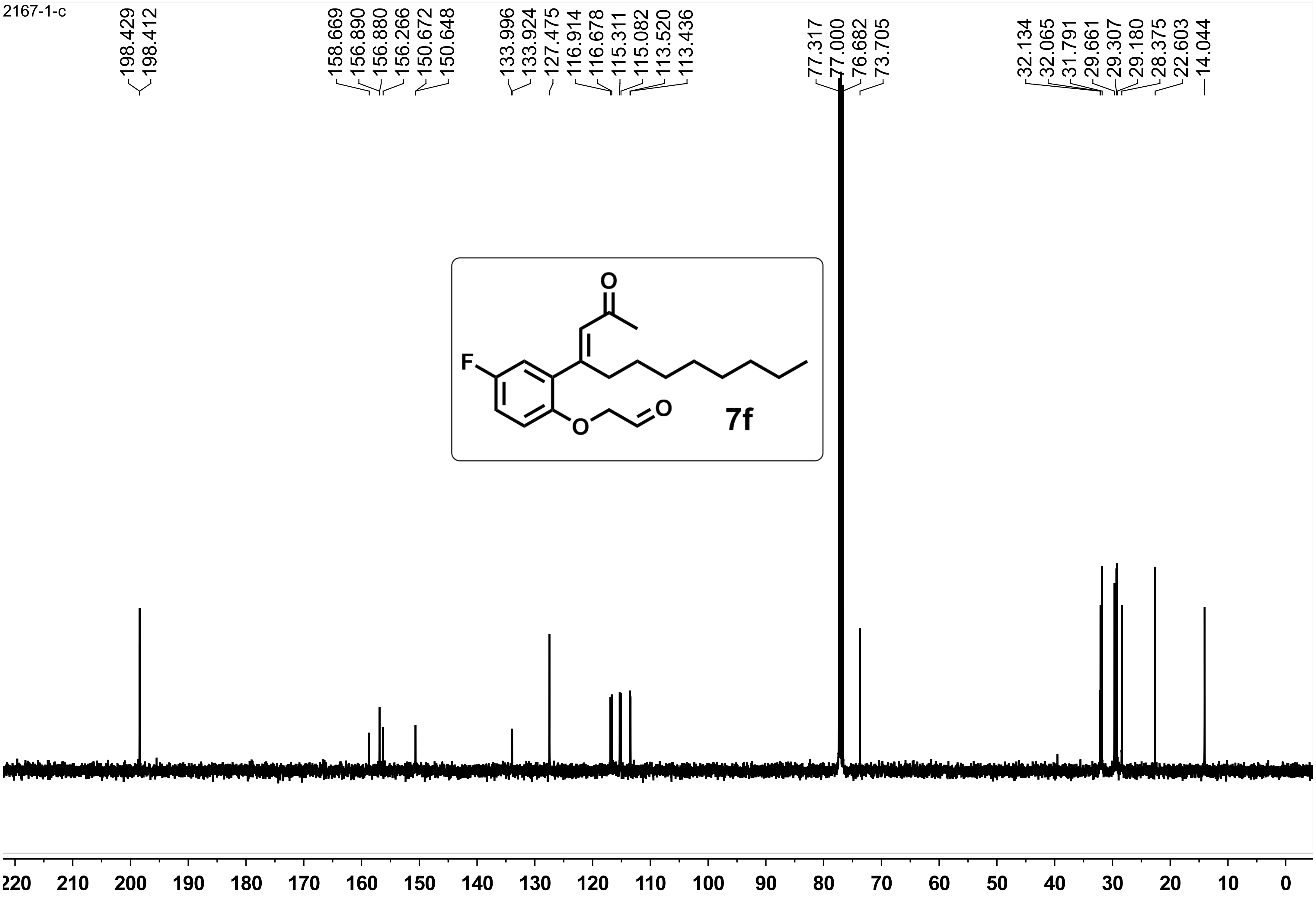

Supplementary Figure 76. ^{13}C NMR of **7f**

zq-2167-1-f

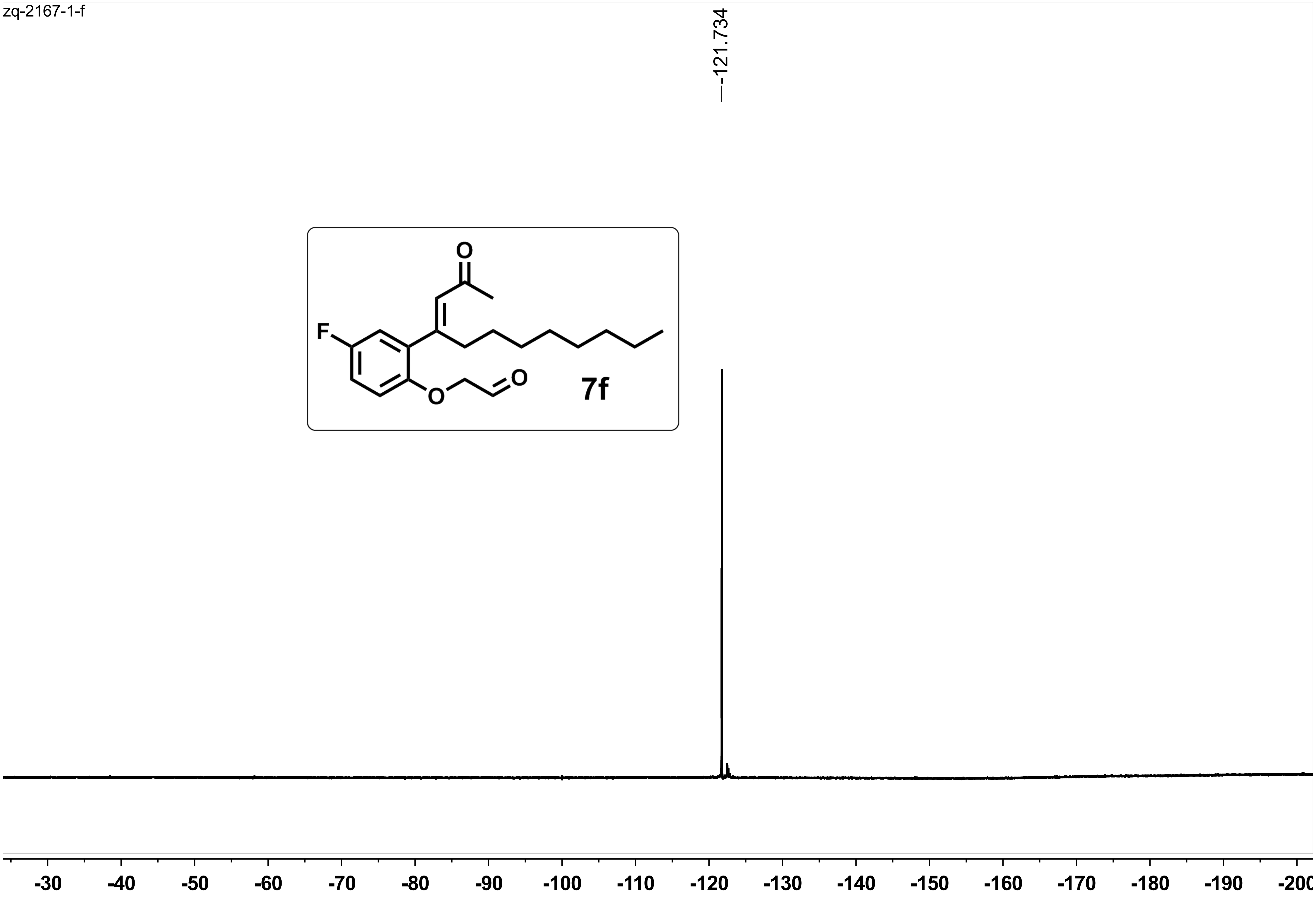

Supplementary Figure 77. ^{19}F NMR of **7f**

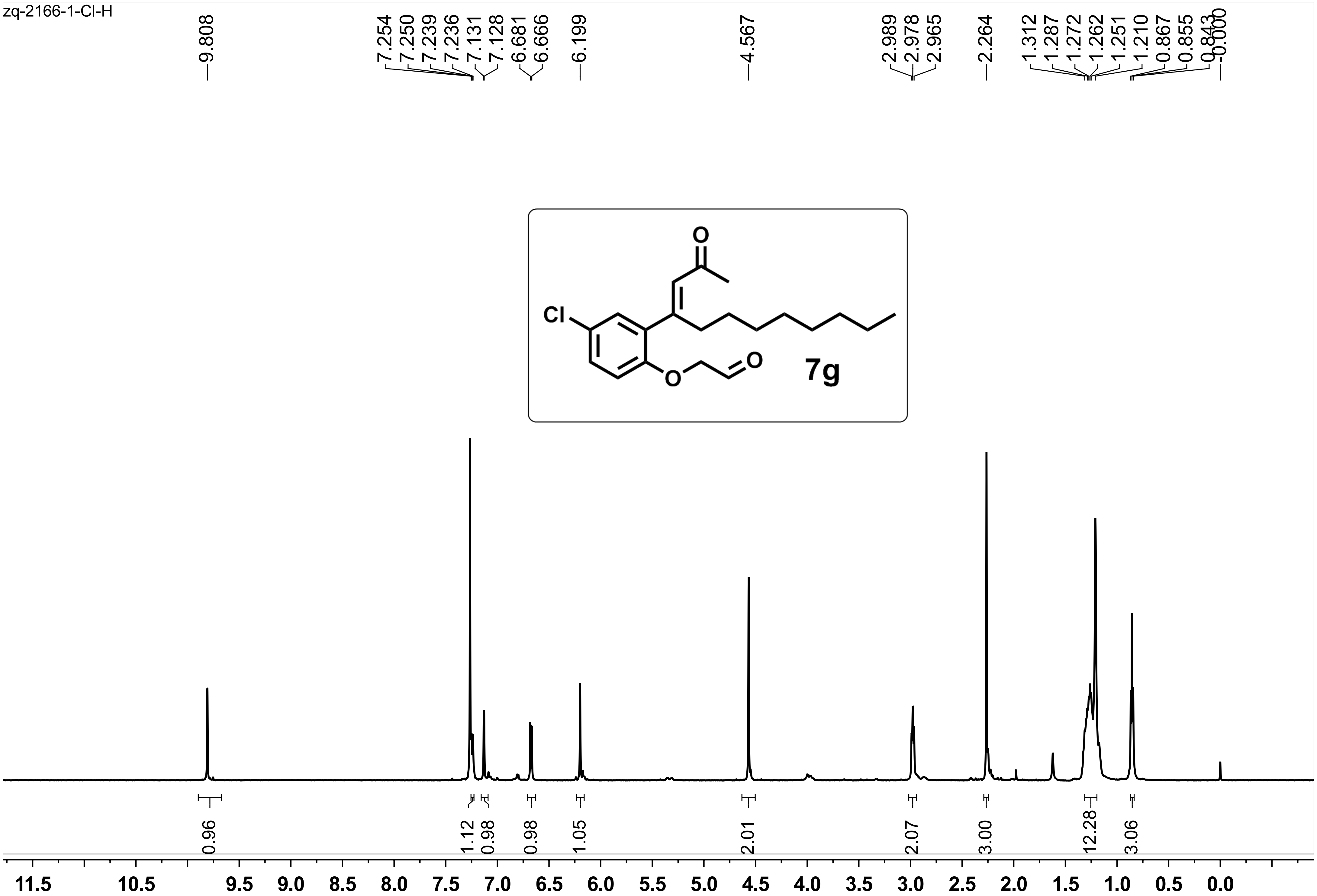

Supplementary Figure 78. ^{1}H NMR of **7g**

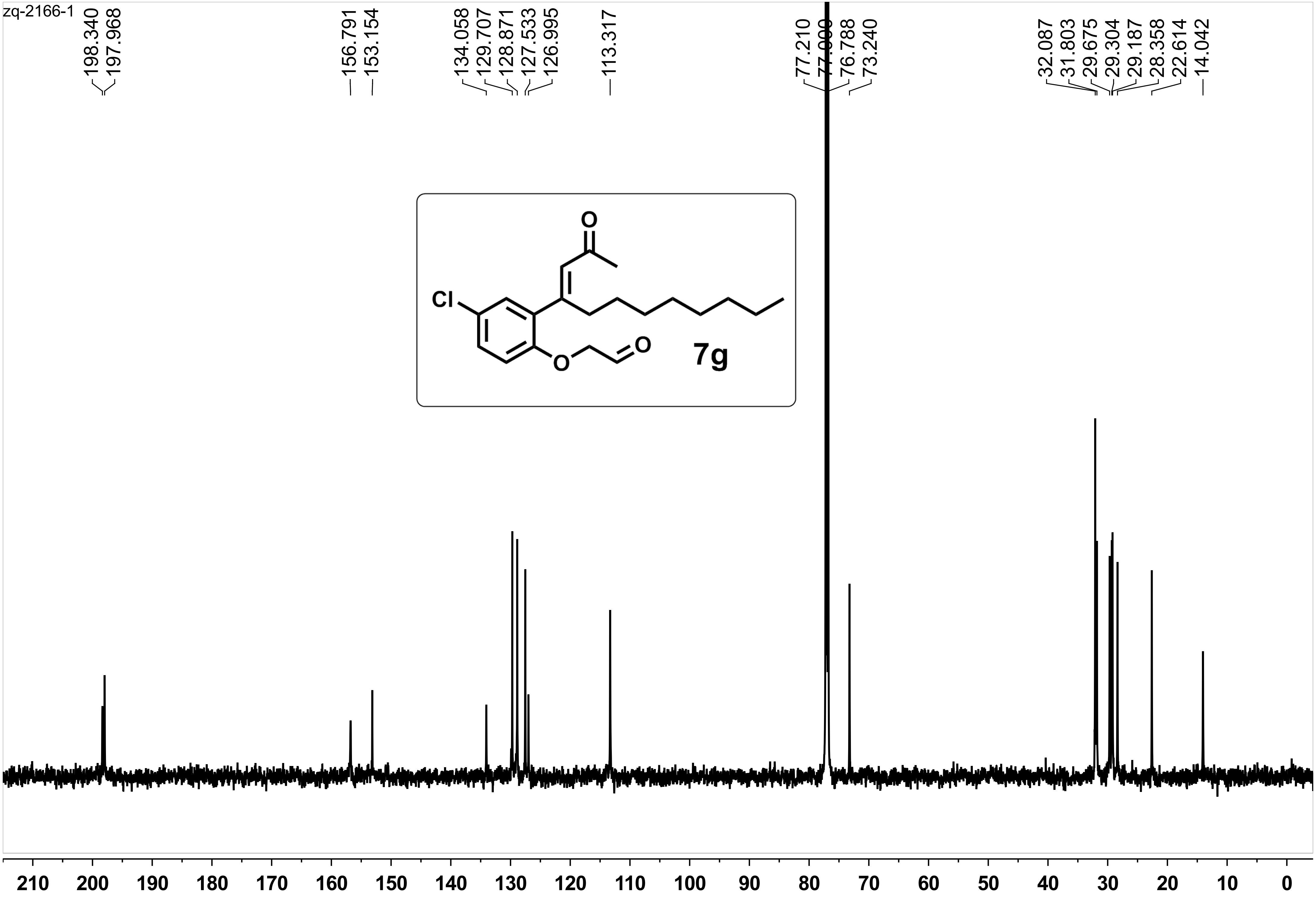

Supplementary Figure 79. ^{13}C NMR of **7g**

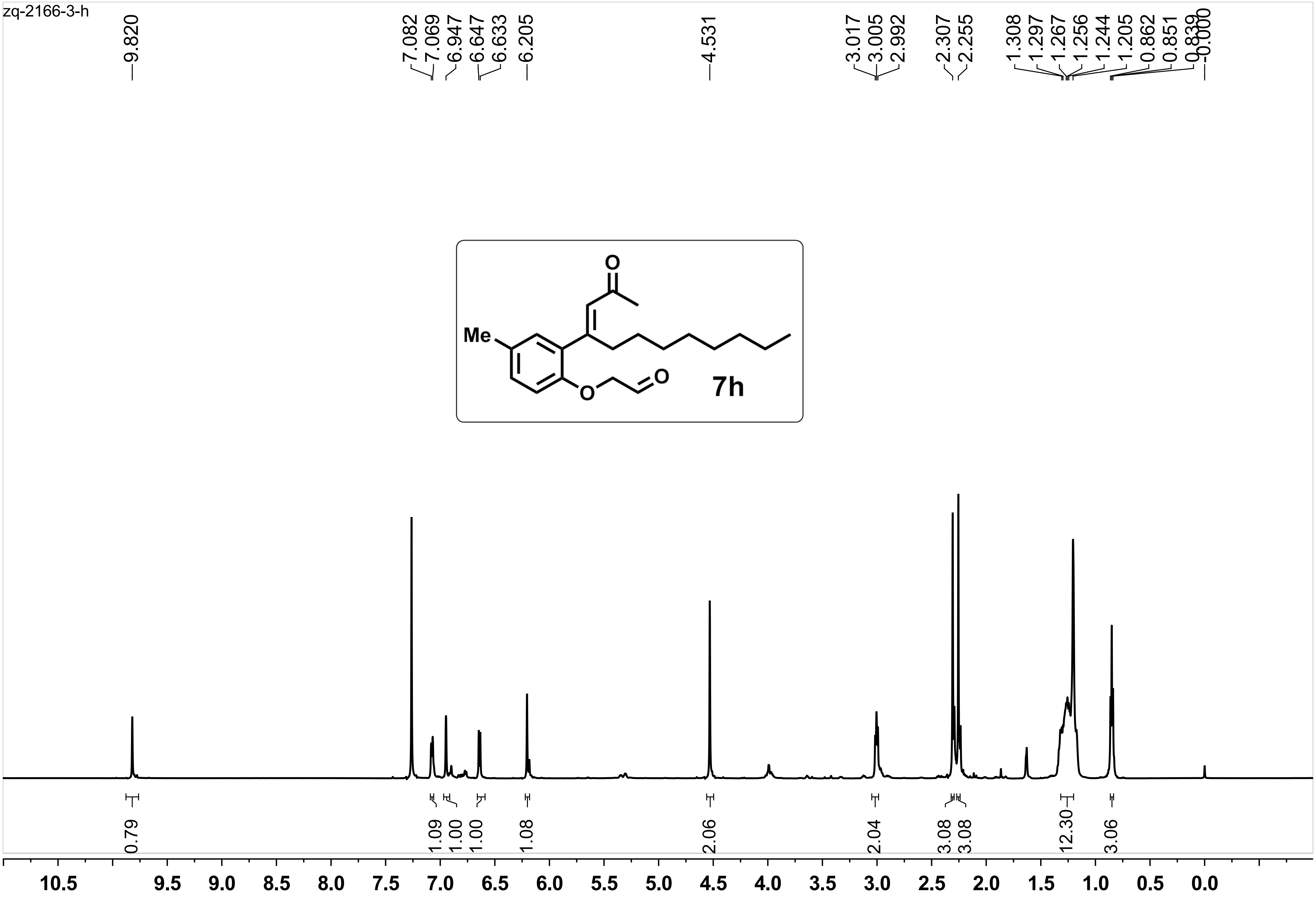

Supplementary Figure 80. ^{1}H NMR of **7h**

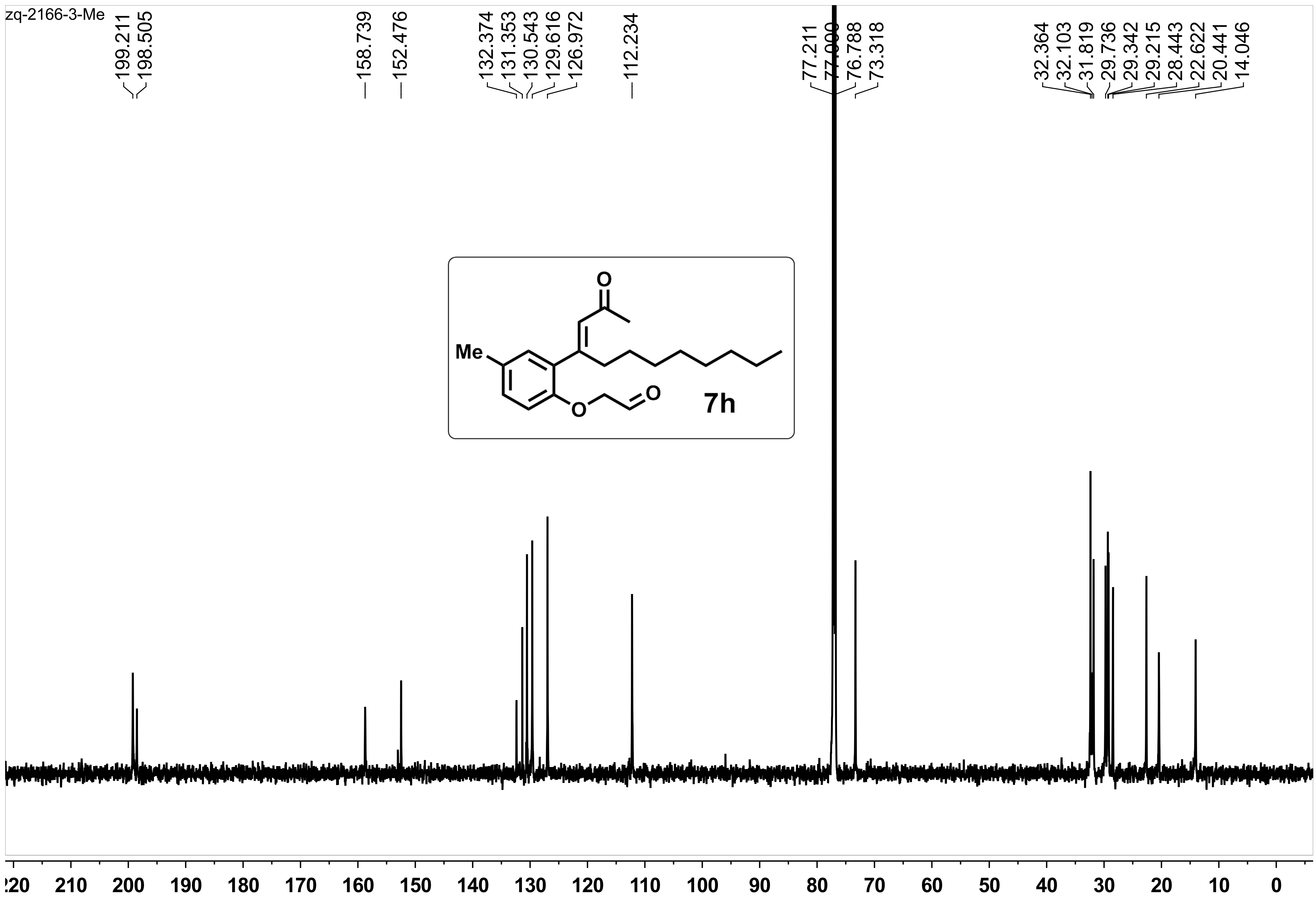

Supplementary Figure 81. ^{13}C NMR of **7h**

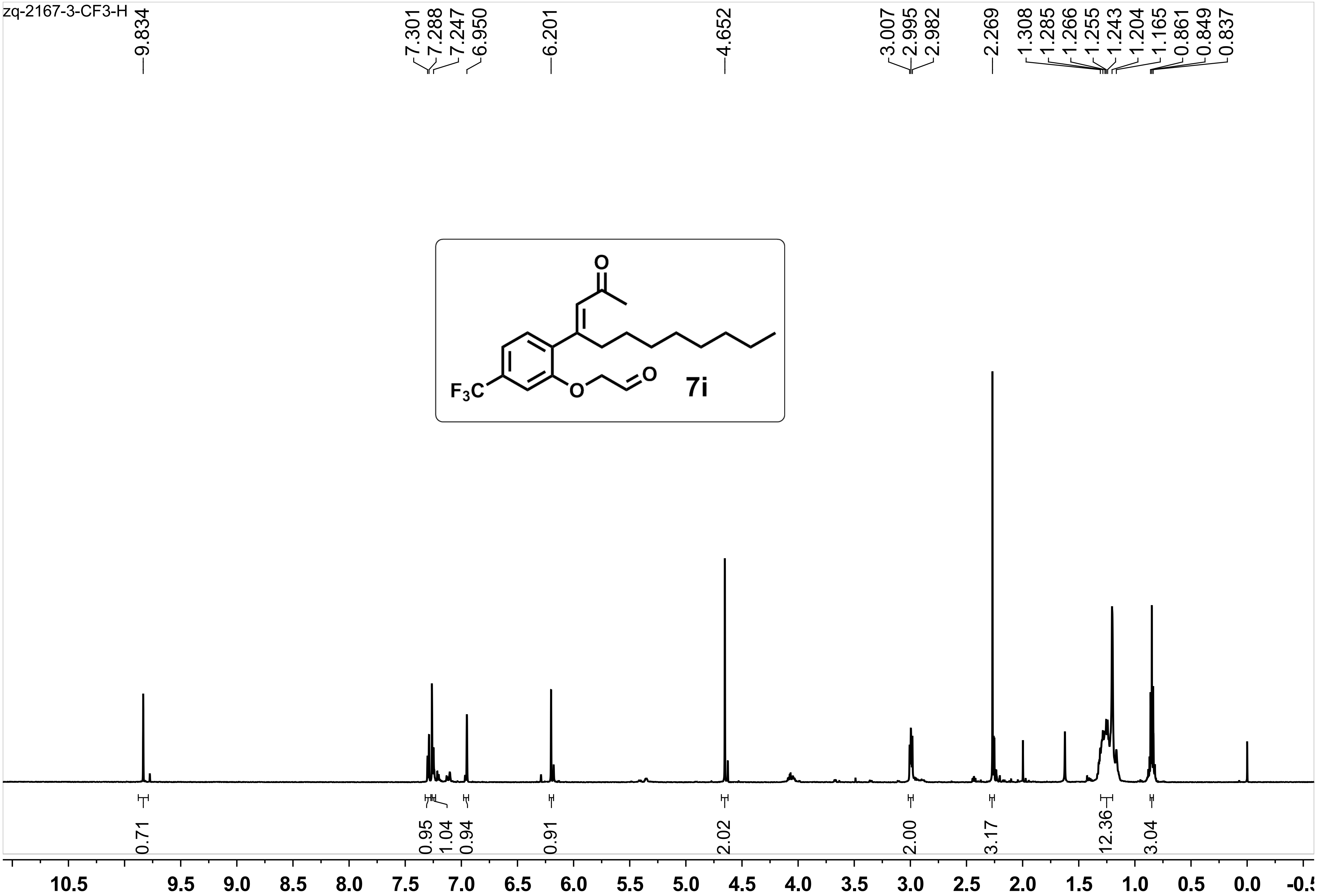

Supplementary Figure 82. ^{1}H NMR of **7i**

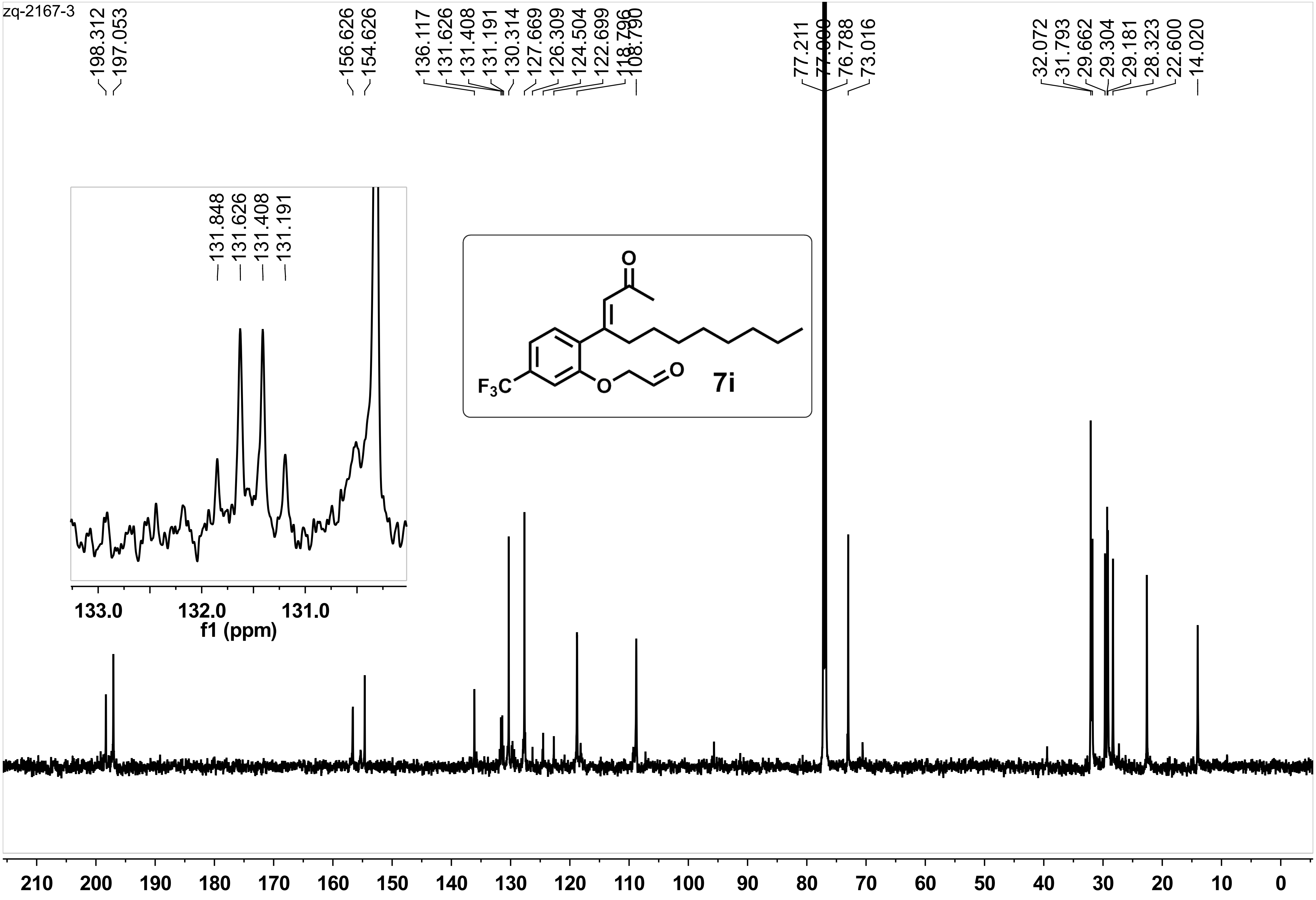

Supplementary Figure 83. ^{13}C NMR of **7i**

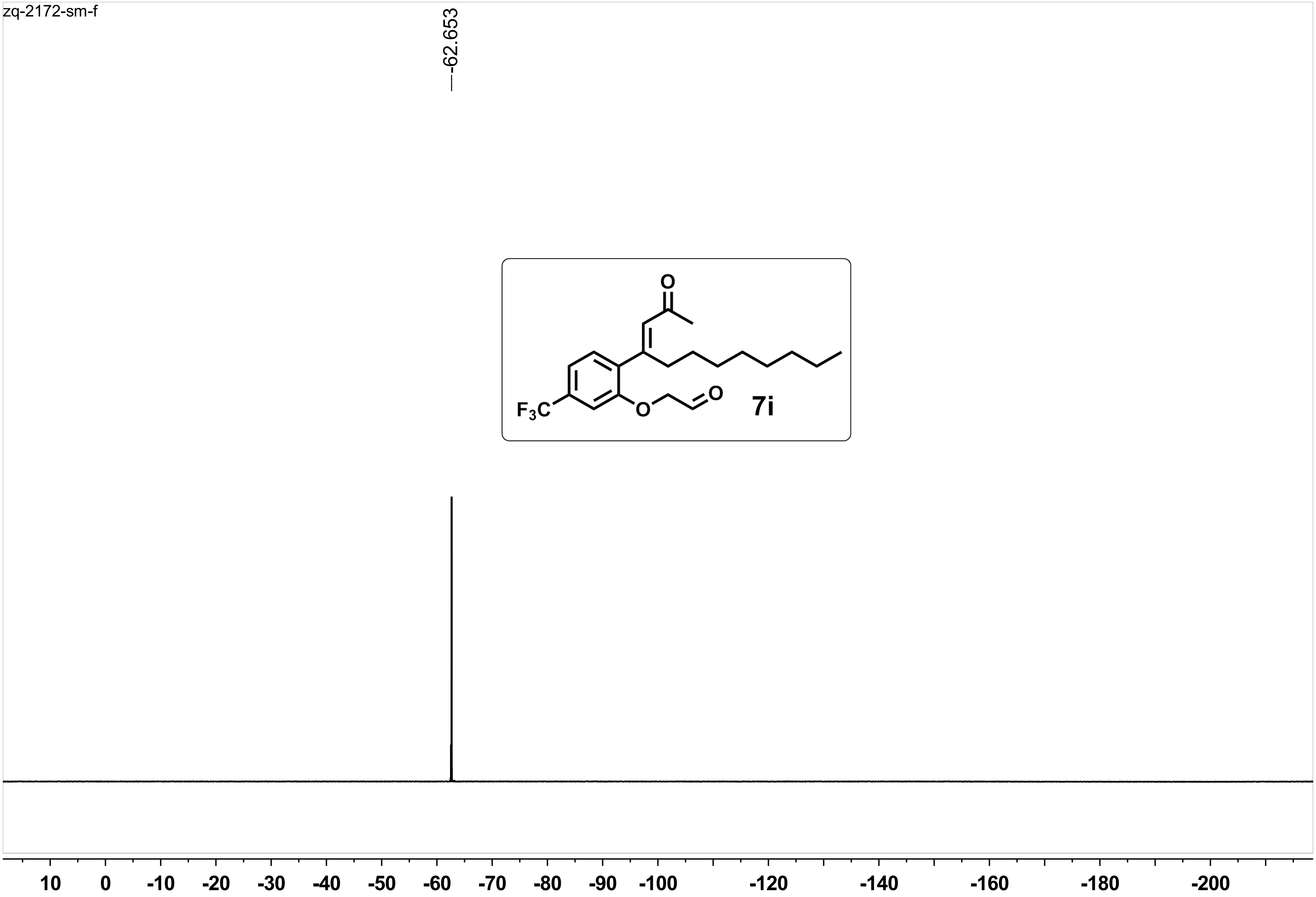

Supplementary Figure 84. ^{19}F NMR of **7i**

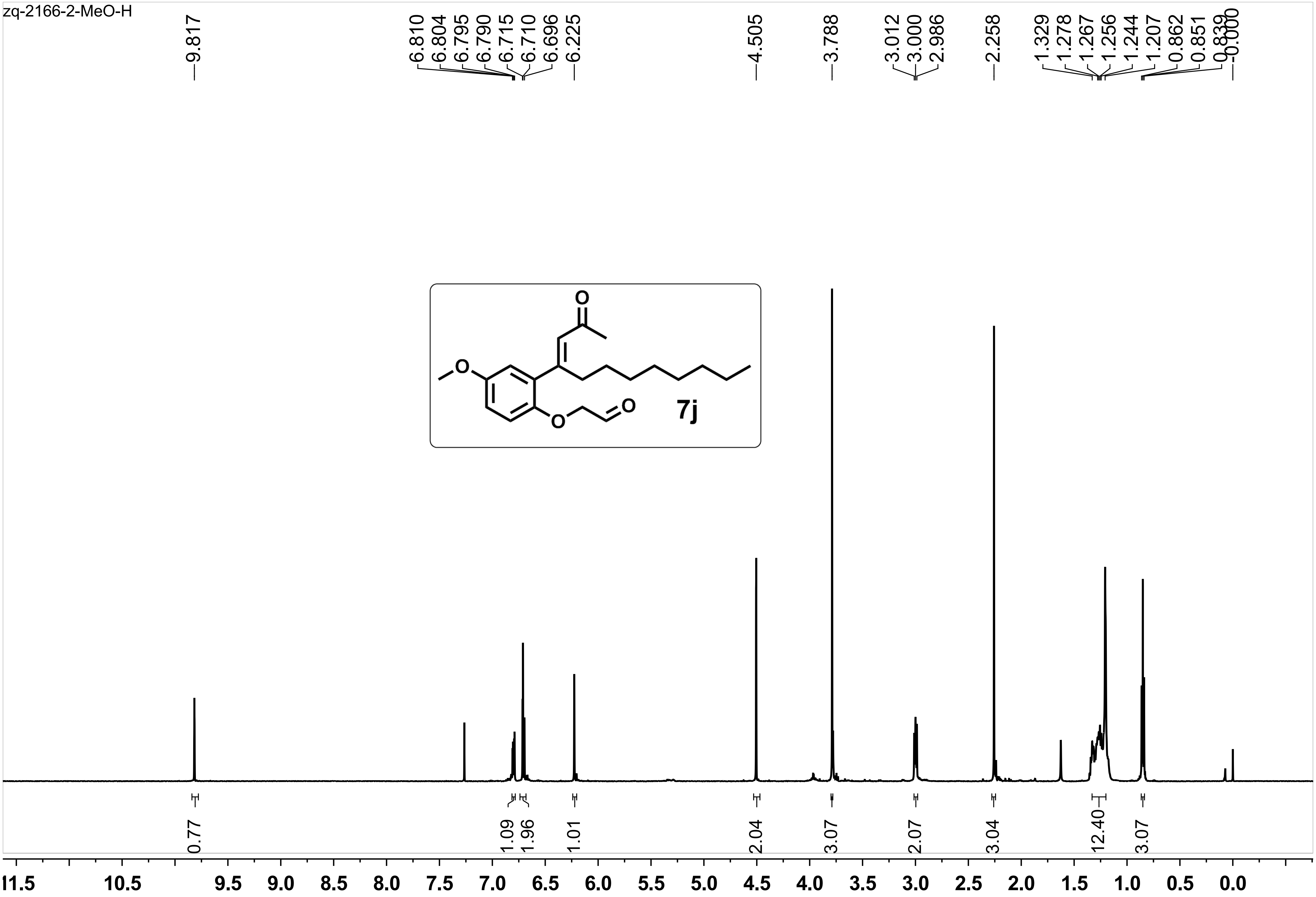

Supplementary Figure 85. ^{1}H NMR of **7j**

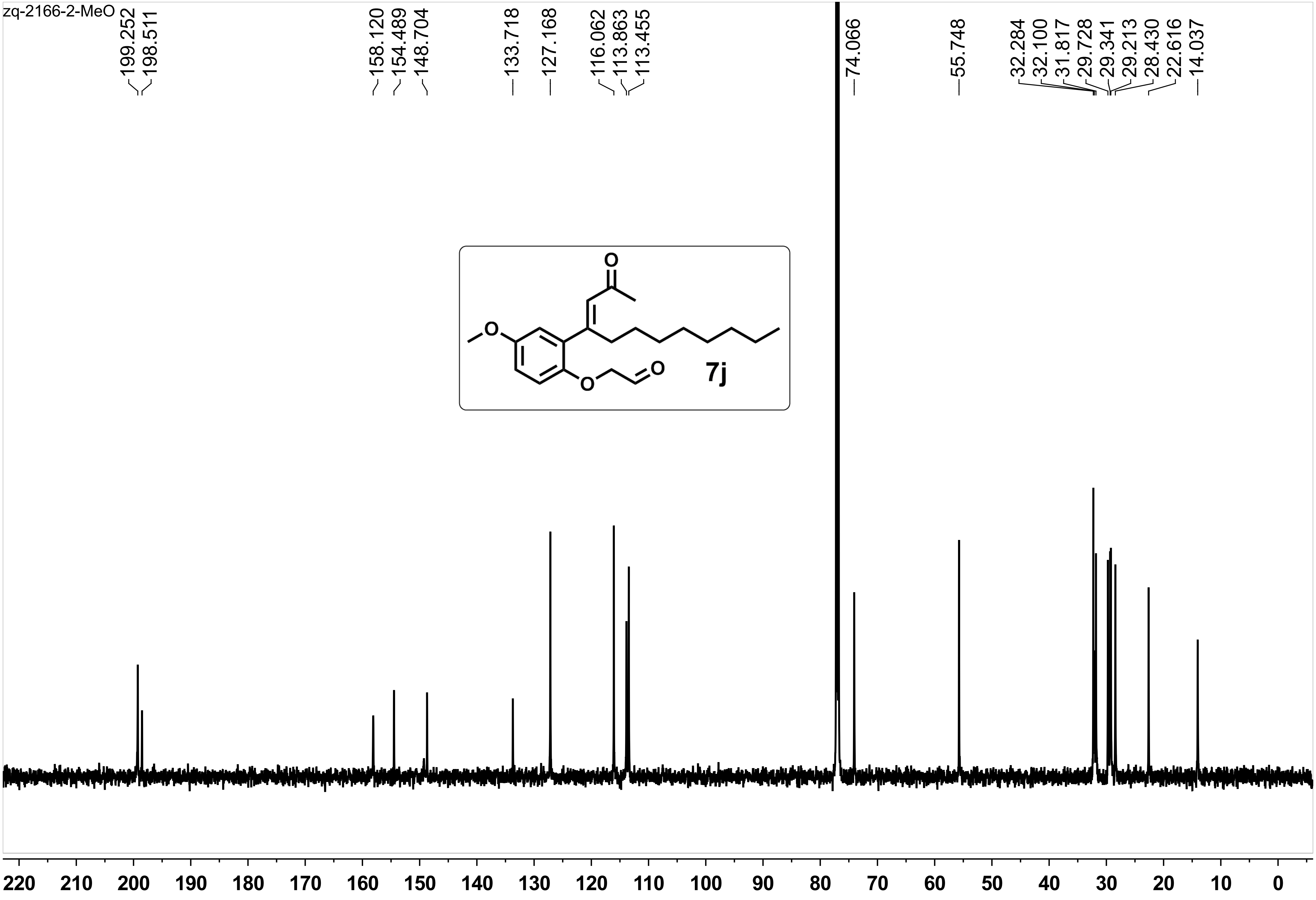

Supplementary Figure 86. ^{13}C NMR of **7j**

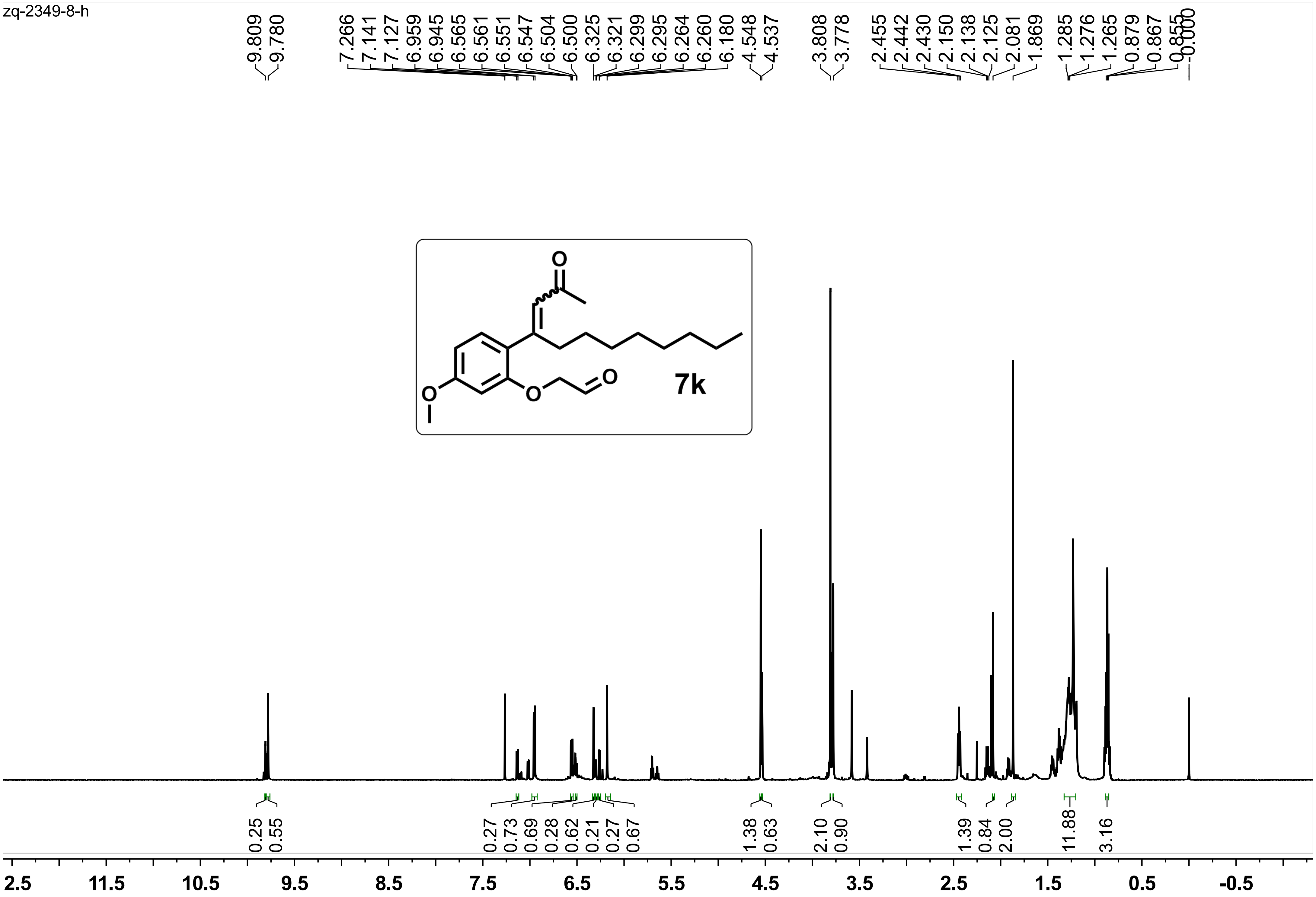

Supplementary Figure 87. ^{1}H NMR of **7k**

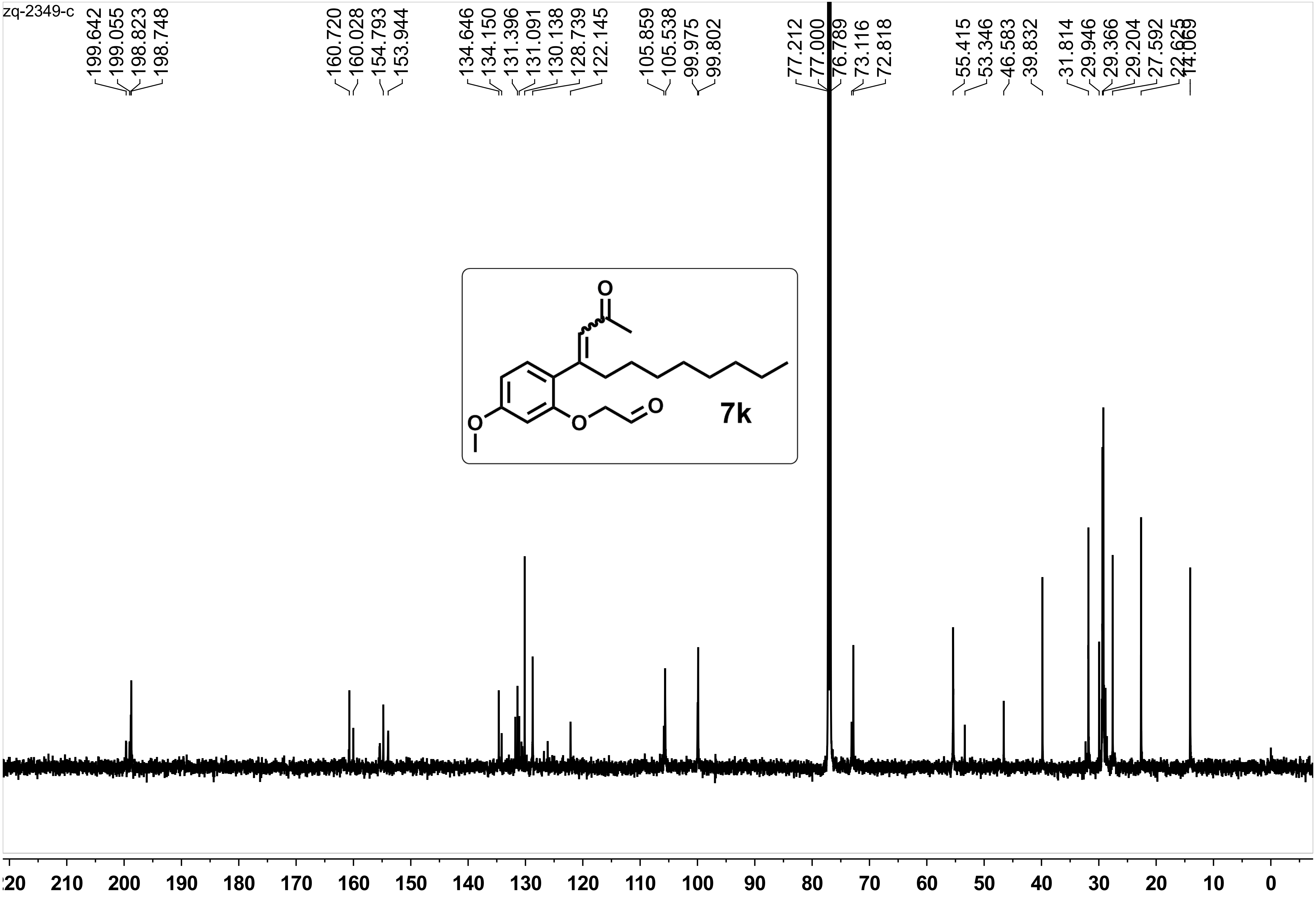

Supplementary Figure 88. ^{13}C NMR of **7k**

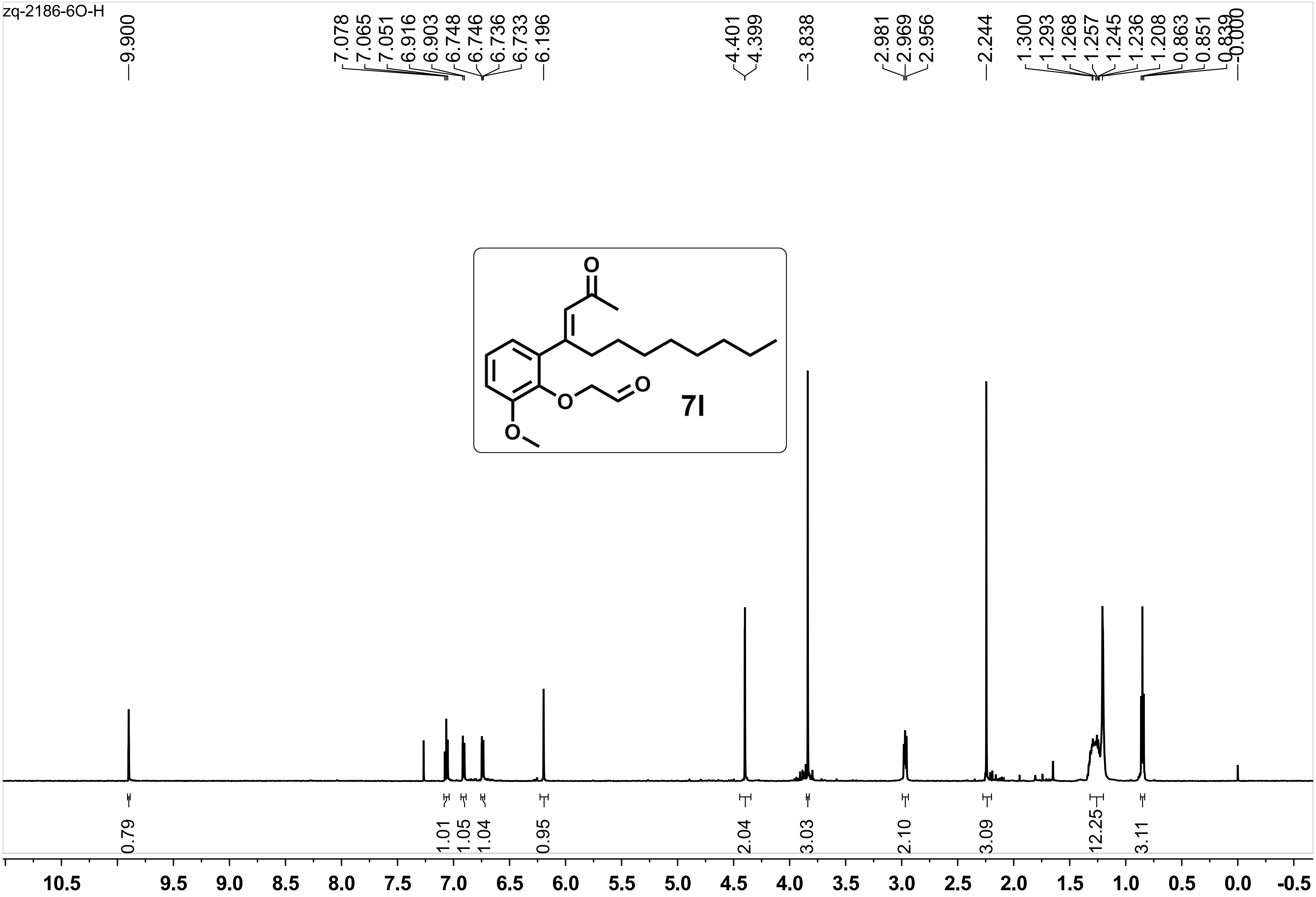

Supplementary Figure 89. ^{1}H NMR of **7l**

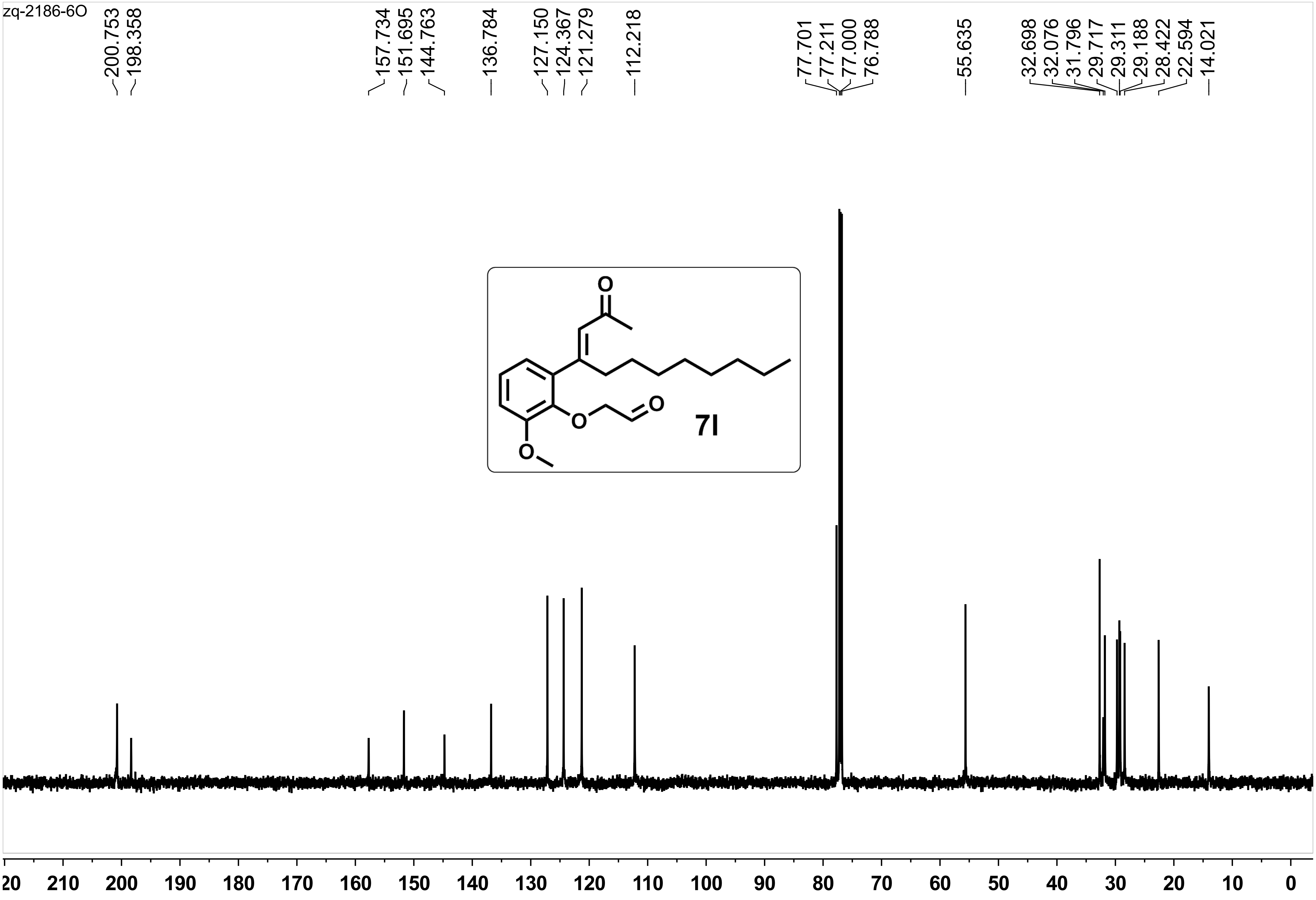

Supplementary Figure 90. ^{13}C NMR of **7l**

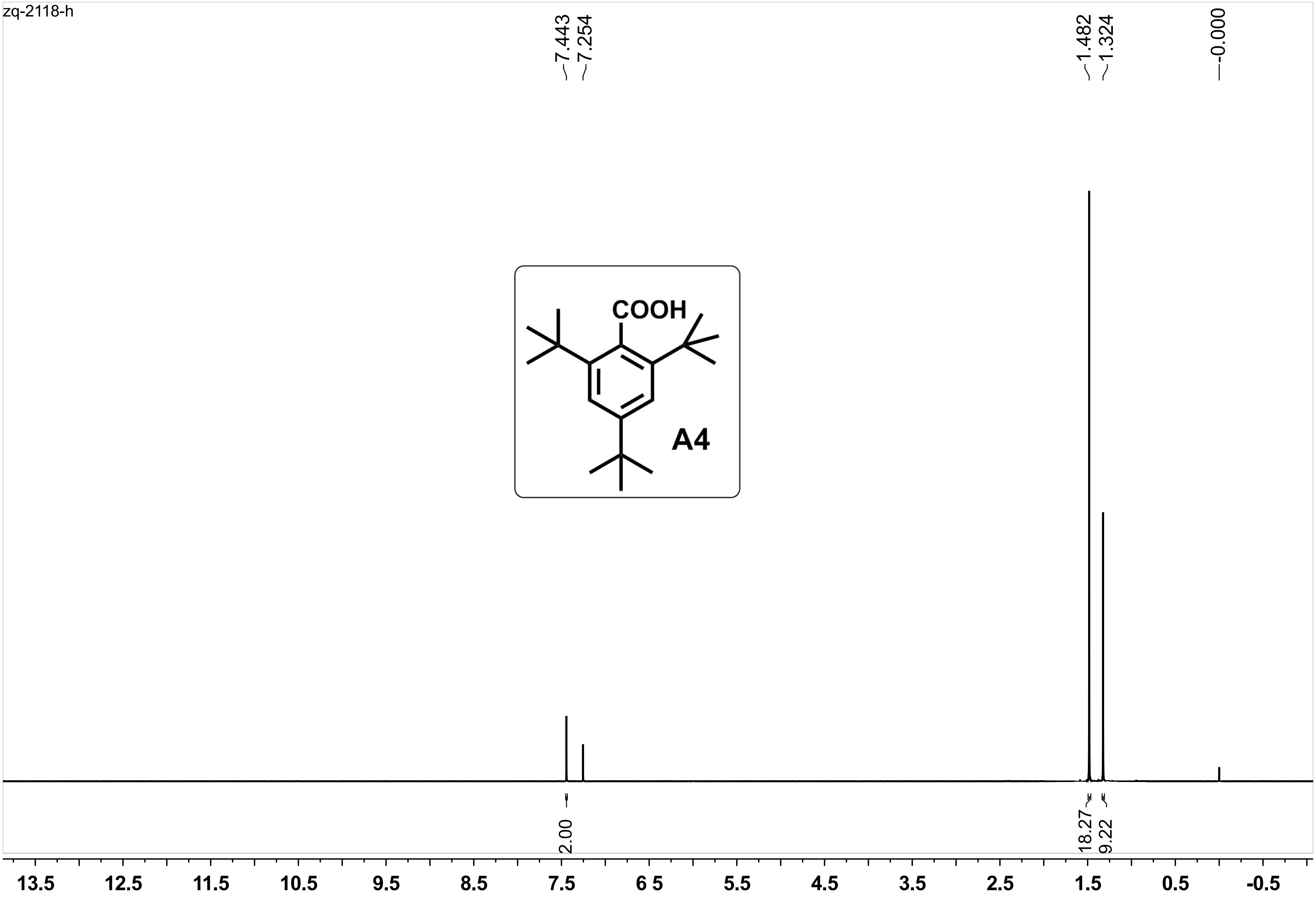

Supplementary Figure 91. ^{1}H NMR of **A4**

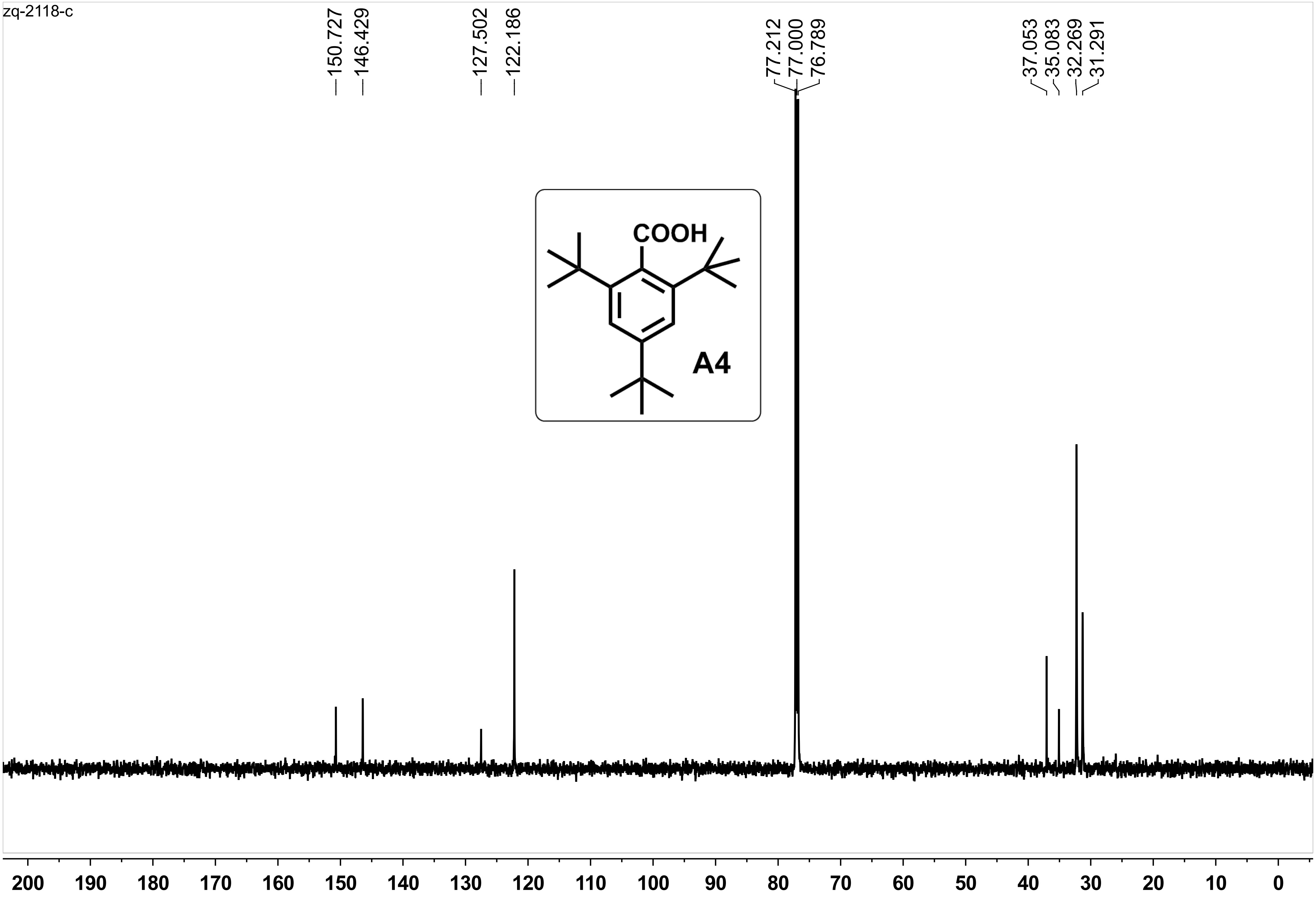

Supplementary Figure 92. ^{13}C NMR of **A4**

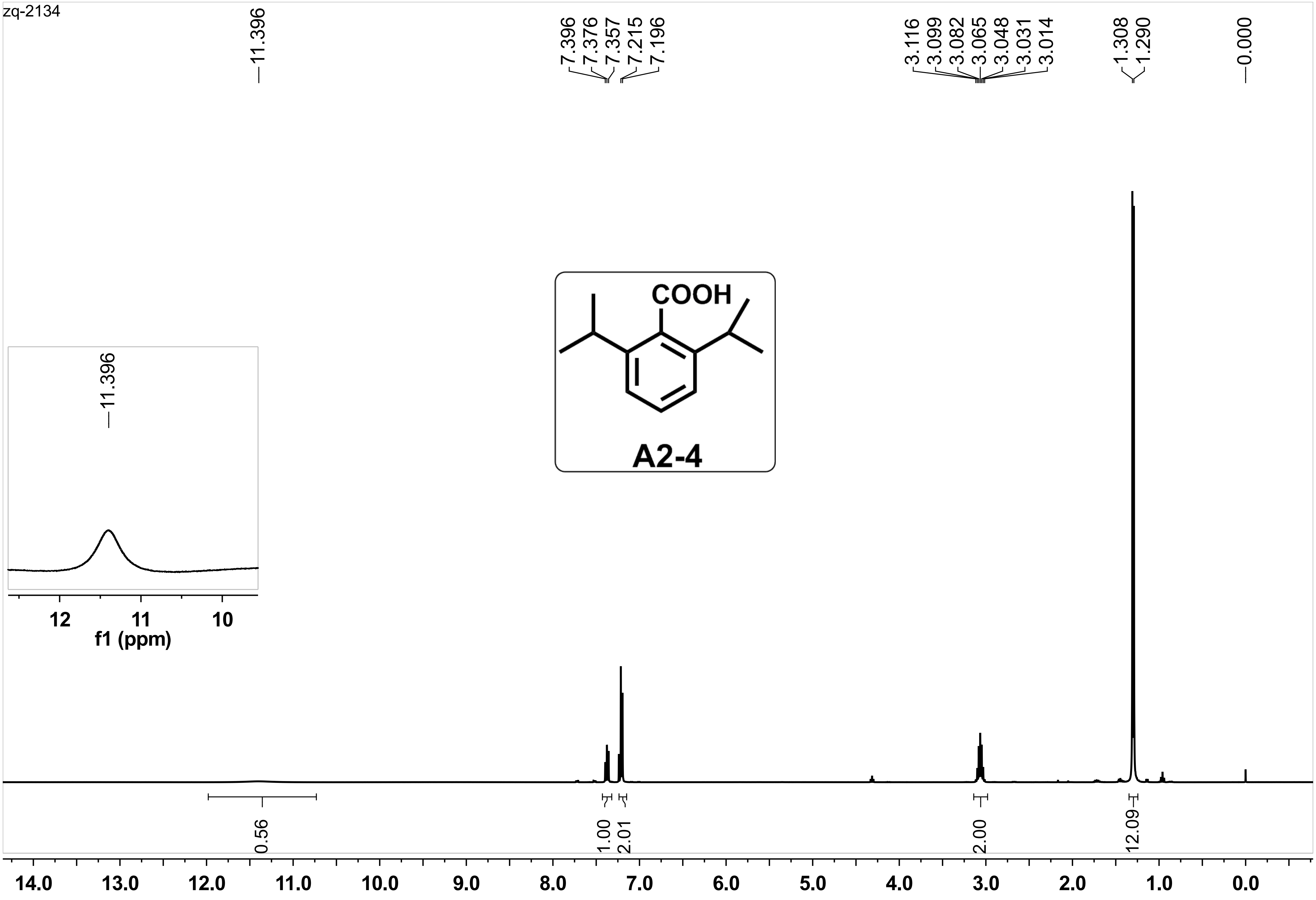

Supplementary Figure 93. ^{1}H NMR of **A2-4**

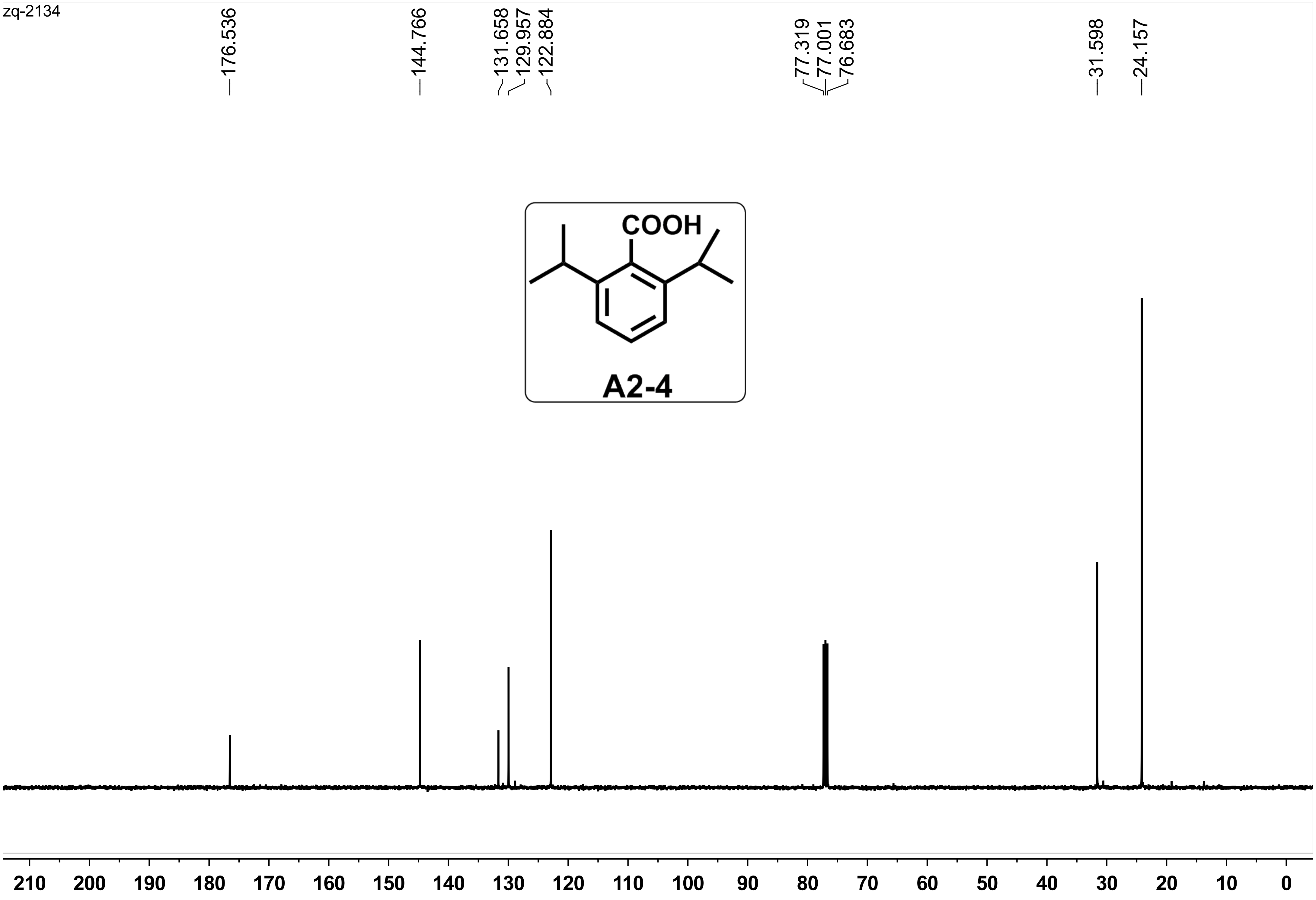

Supplementary Figure 94. ^{13}C NMR of **A2-4**

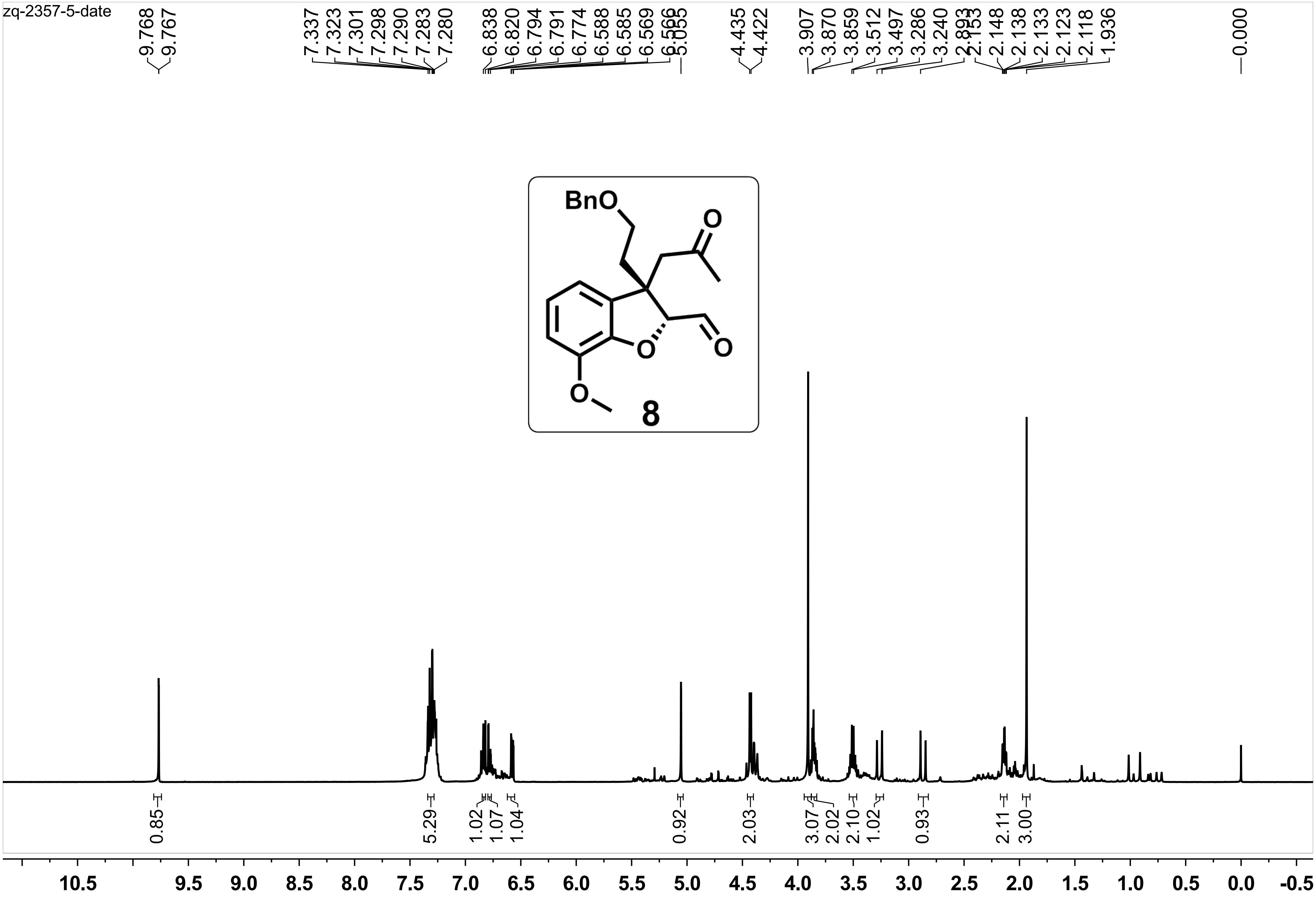

Supplementary Figure 95. ^{1}H NMR of **8**

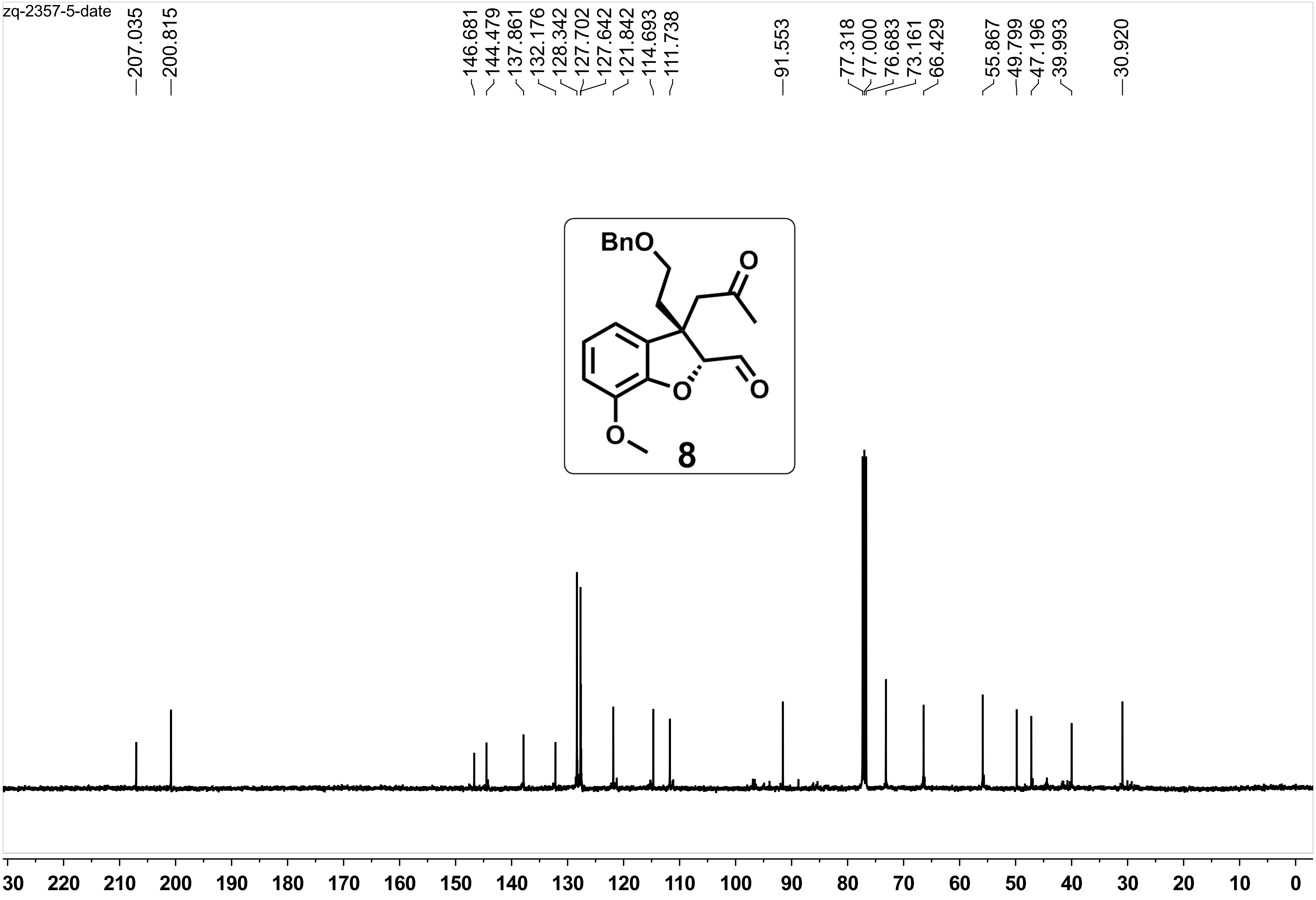

Supplementary Figure 96. ^{13}C NMR of **8**

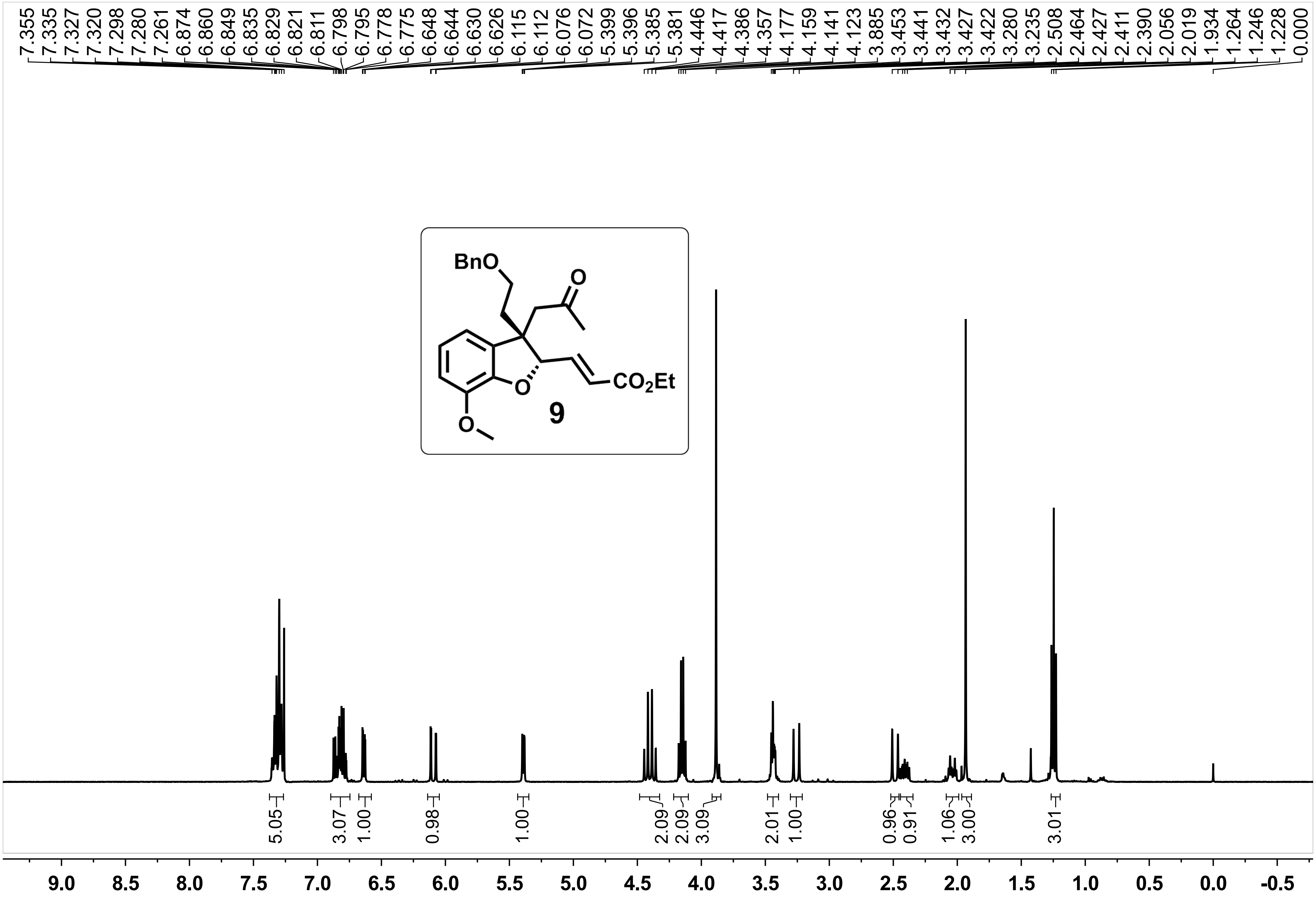

Supplementary Figure 97. ^{1}H NMR of **9**

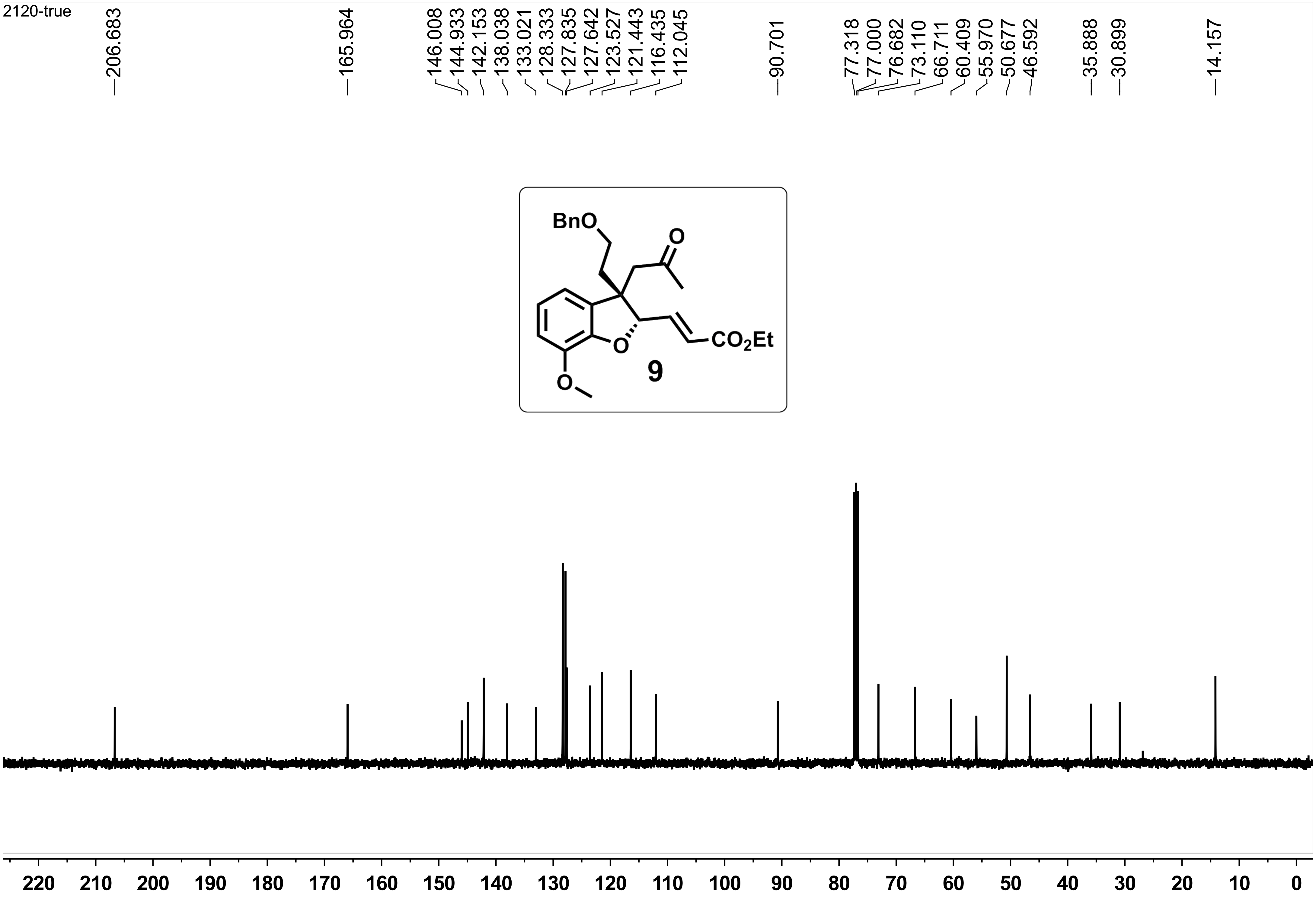

Supplementary Figure 98. ^{13}C NMR of **9**

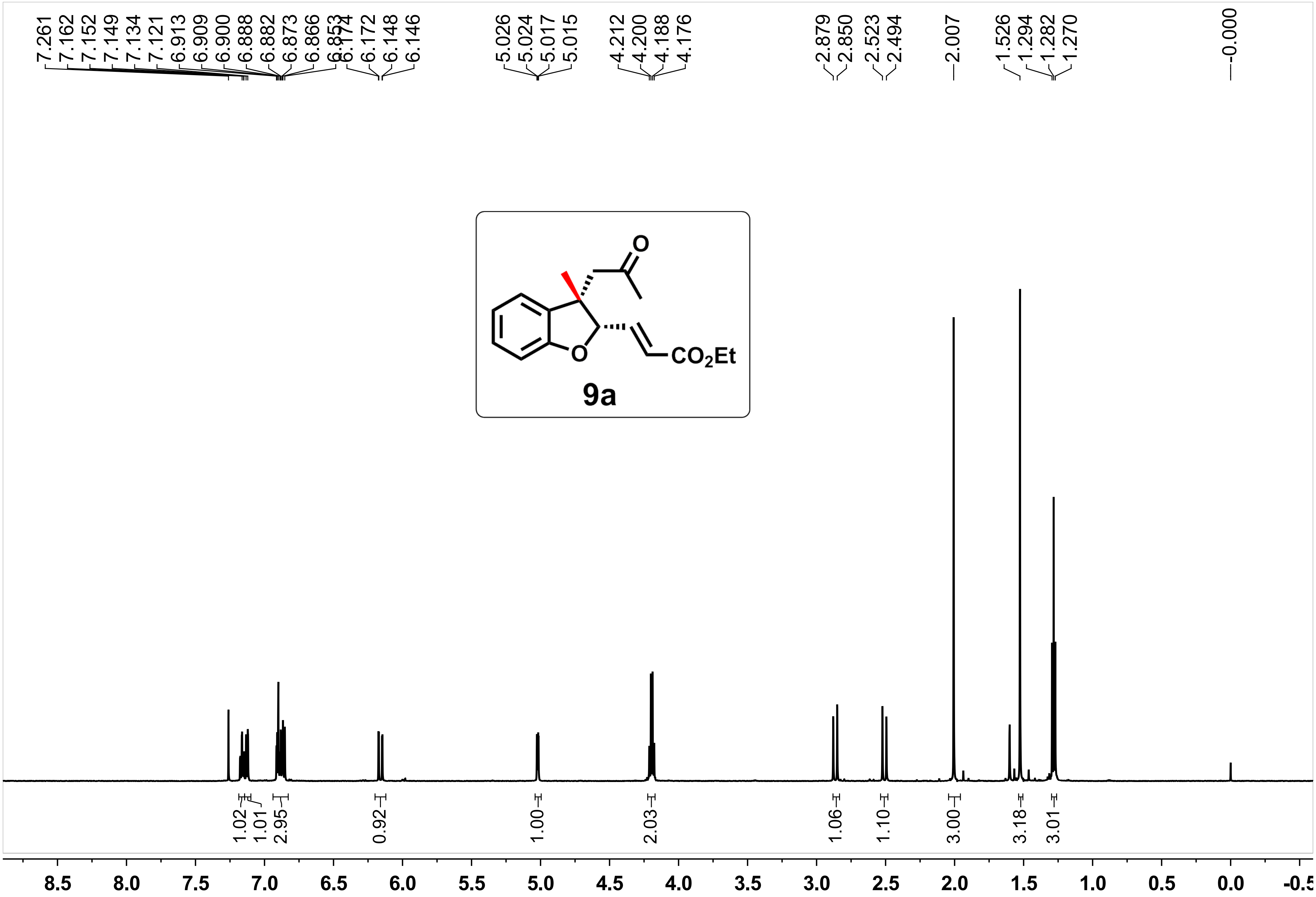

Supplementary Figure 99. ^{1}H NMR of **9a**

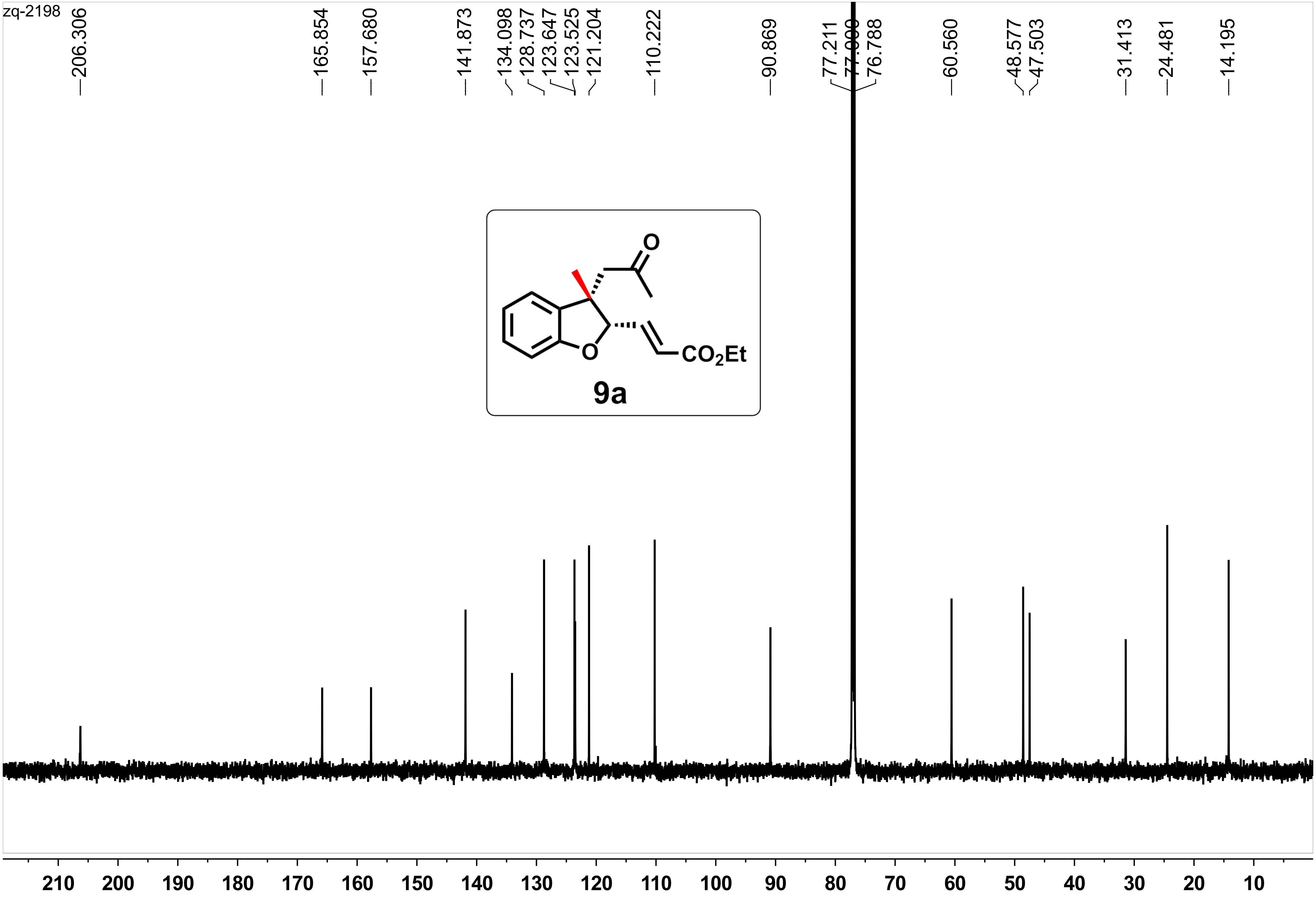

Supplementary Figure 100. ^{13}C NMR of **9a**

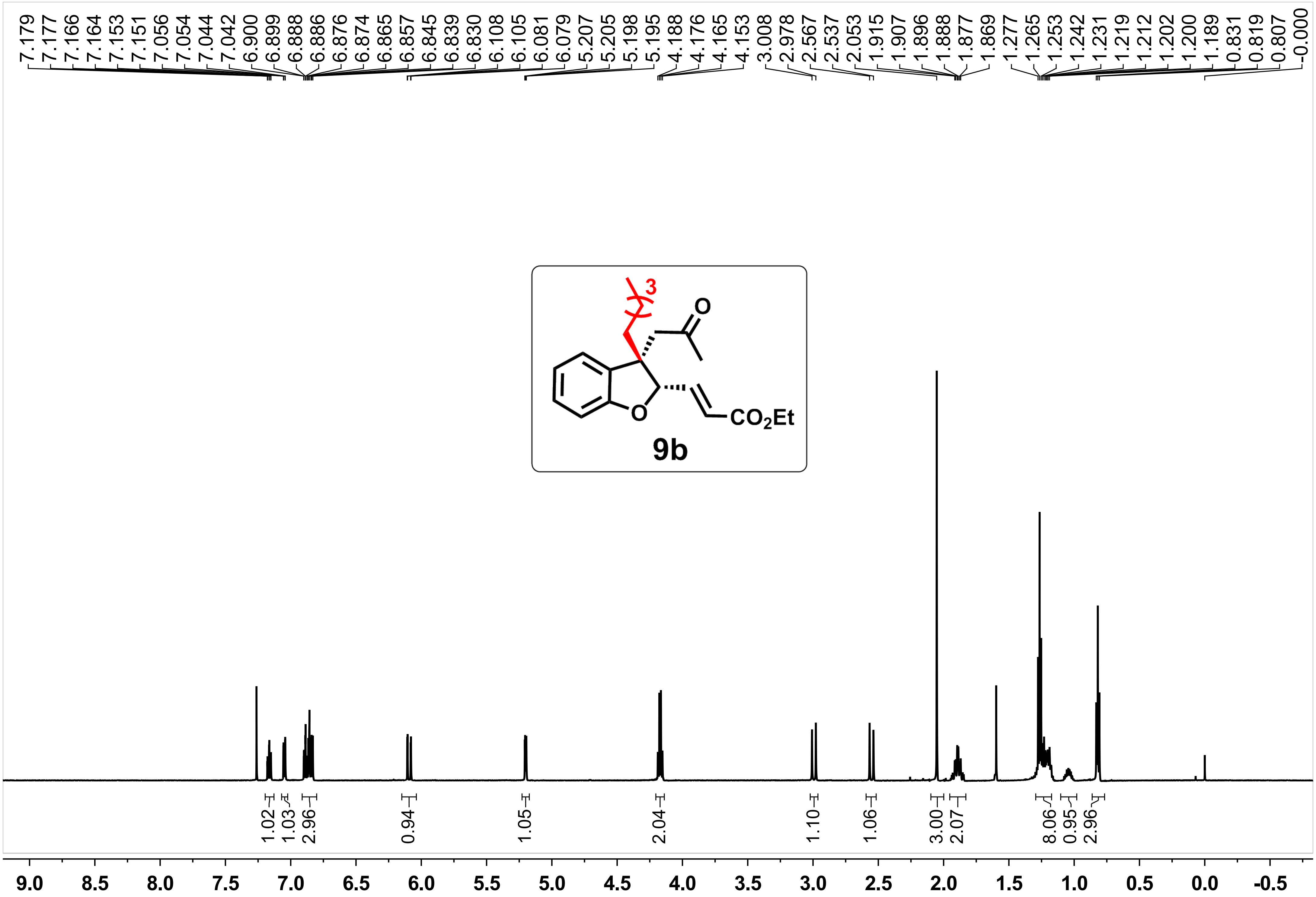

Supplementary Figure 101. ^{1}H NMR of **9b**

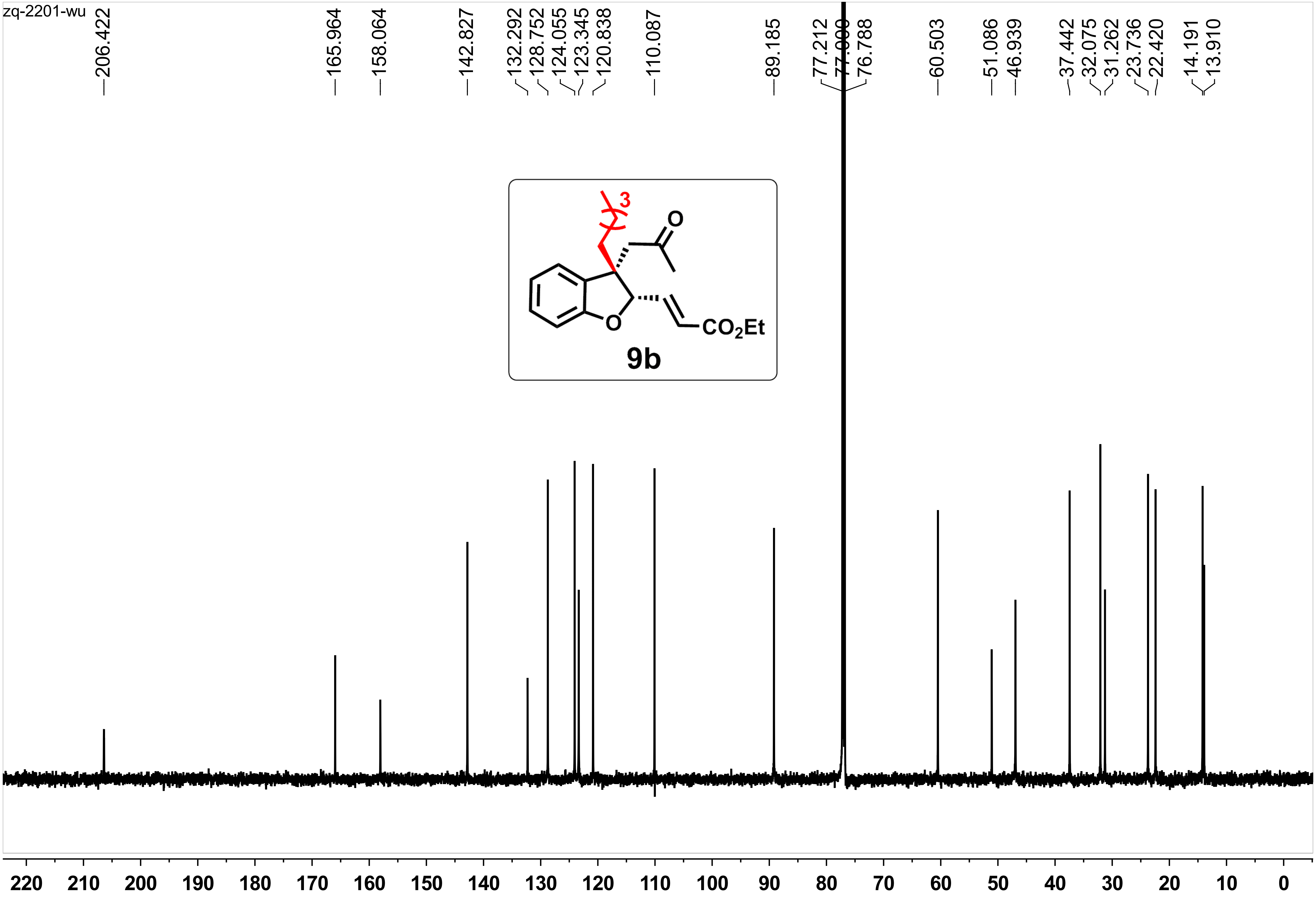

Supplementary Figure 102. ^{13}C NMR of **9b**

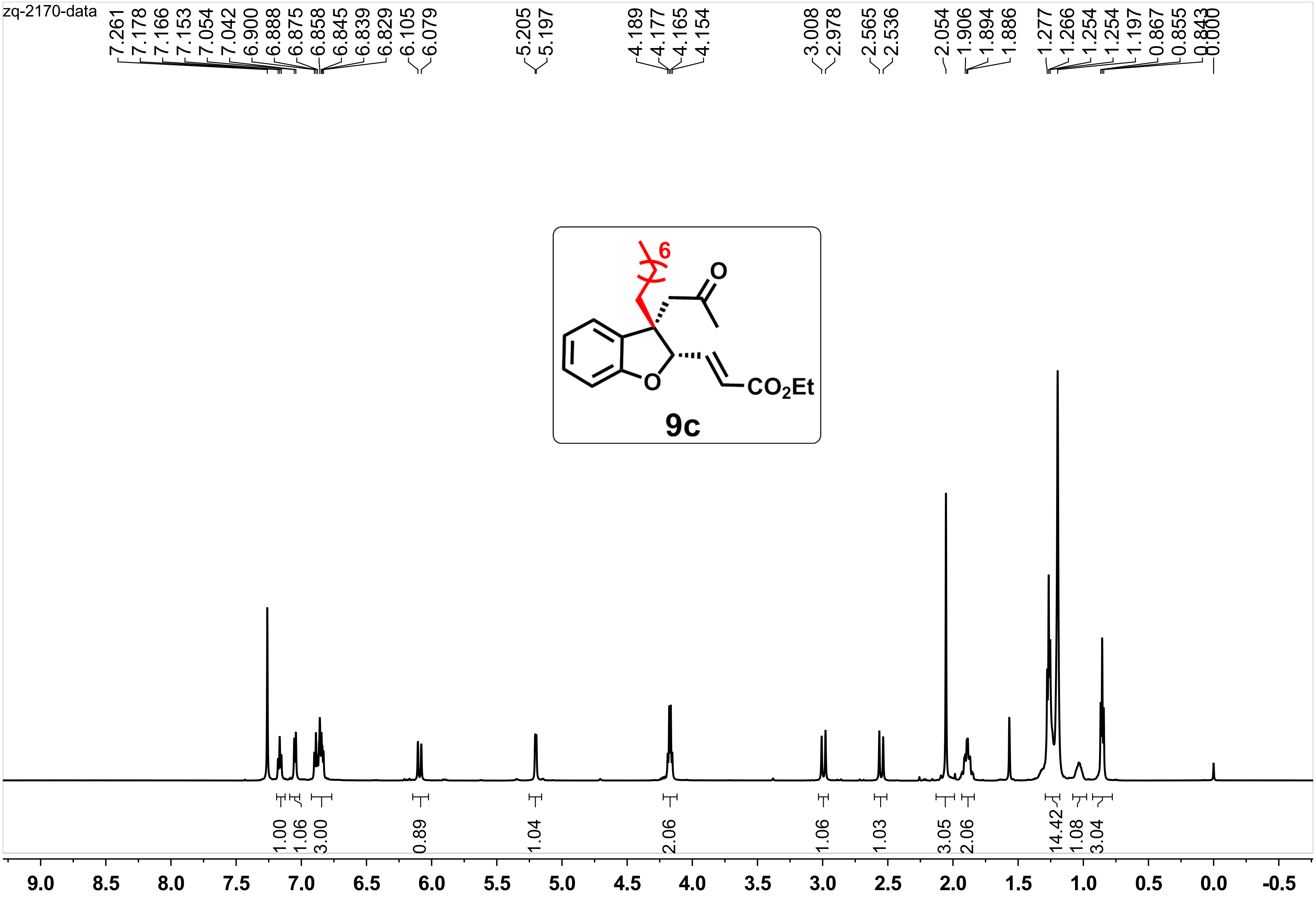

Supplementary Figure 103. ^{1}H NMR of **9c**

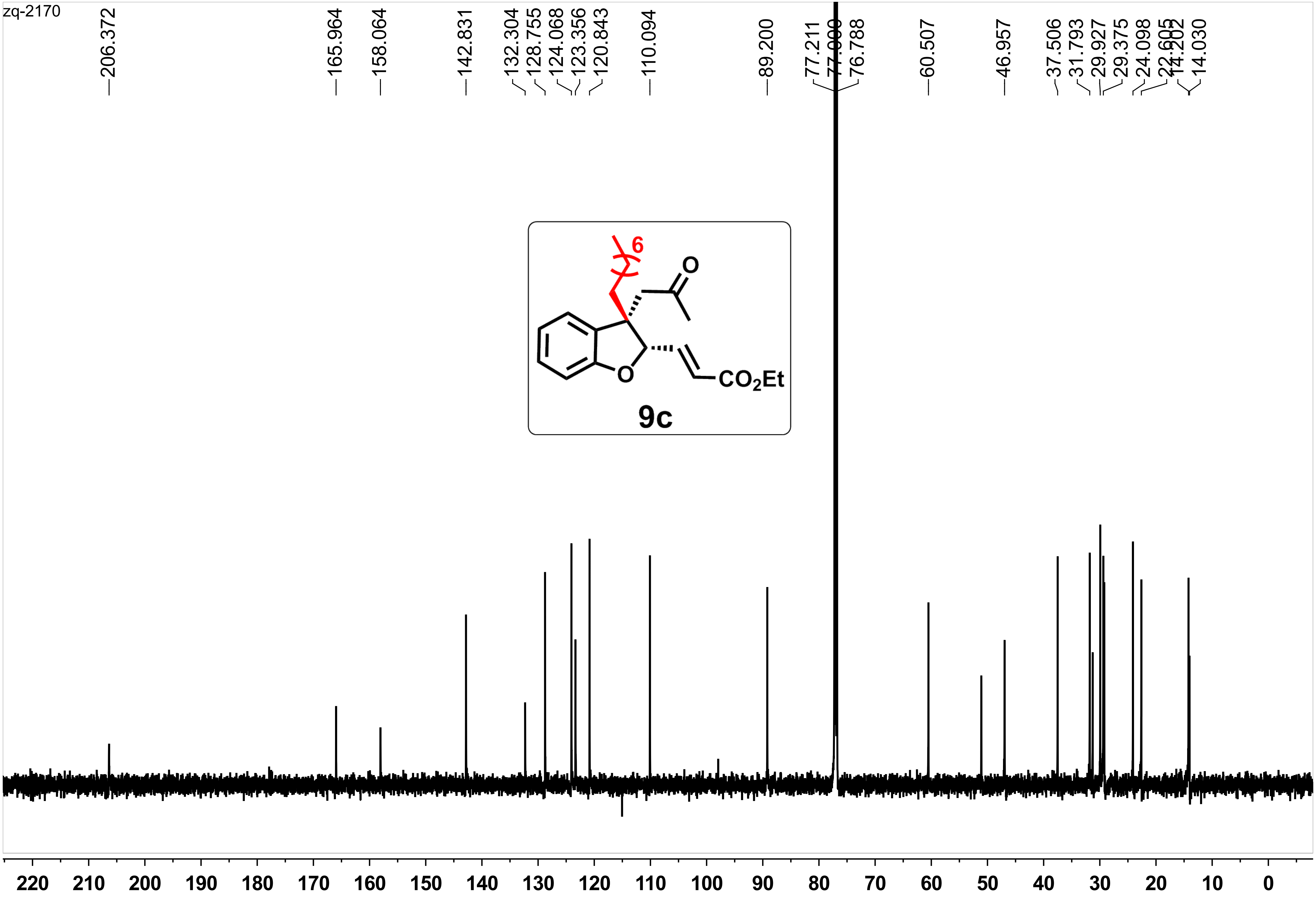

Supplementary Figure 104. ^{13}C NMR of **9c**

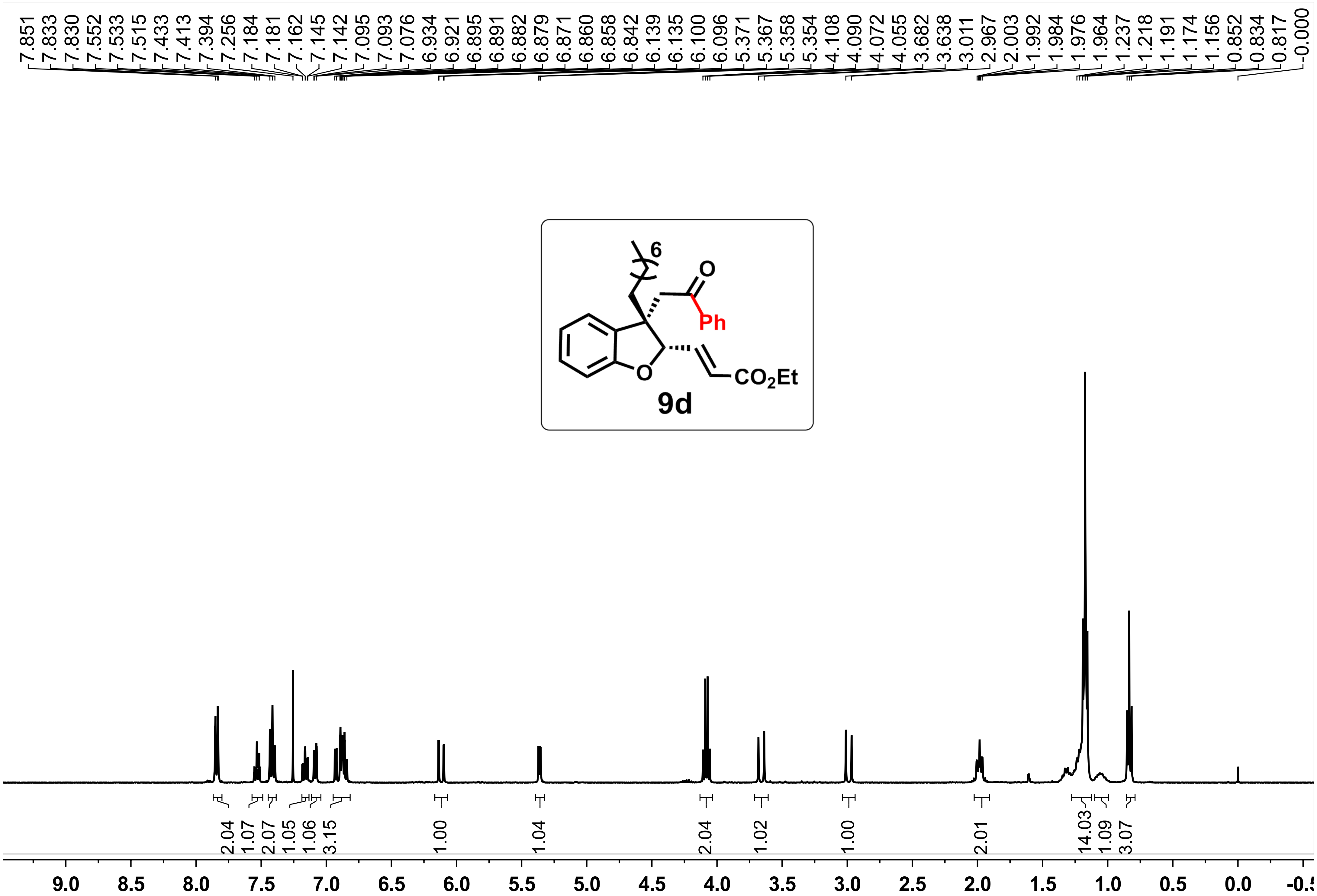

Supplementary Figure 105. ^{1}H NMR of **9d**

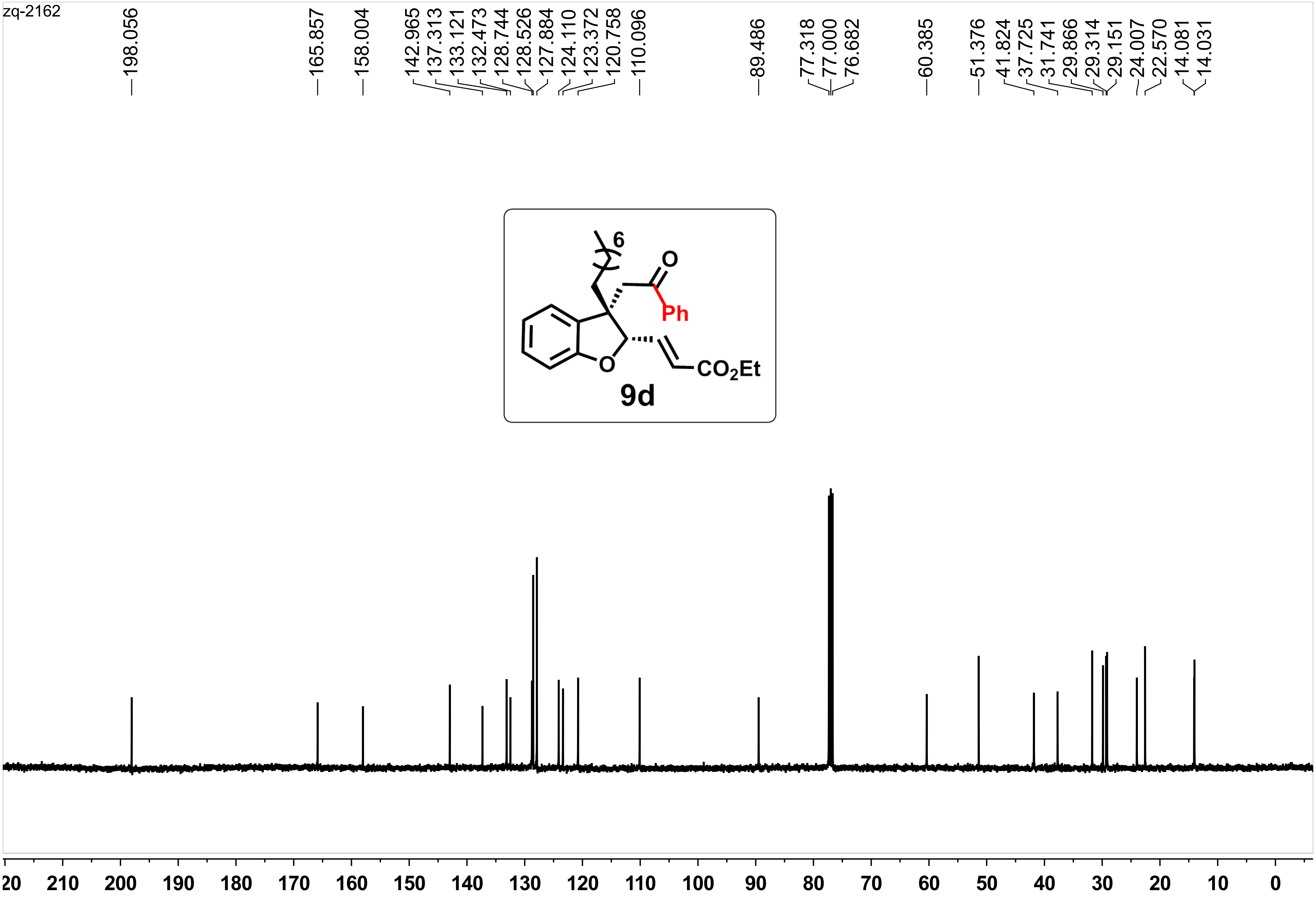

Supplementary Figure 106. ^{13}C NMR of **9d**

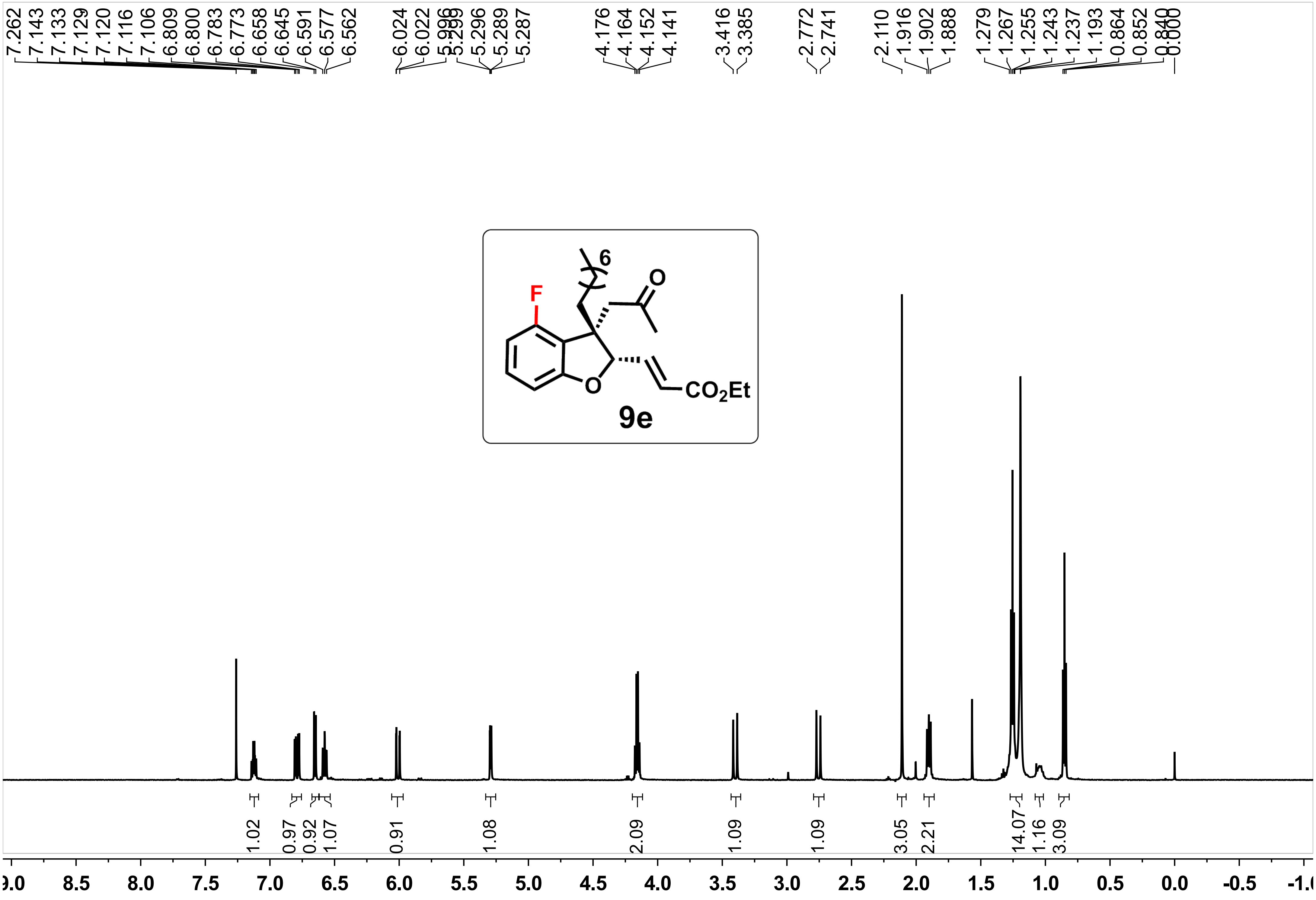

Supplementary Figure 107. ^{1}H NMR of **9e**

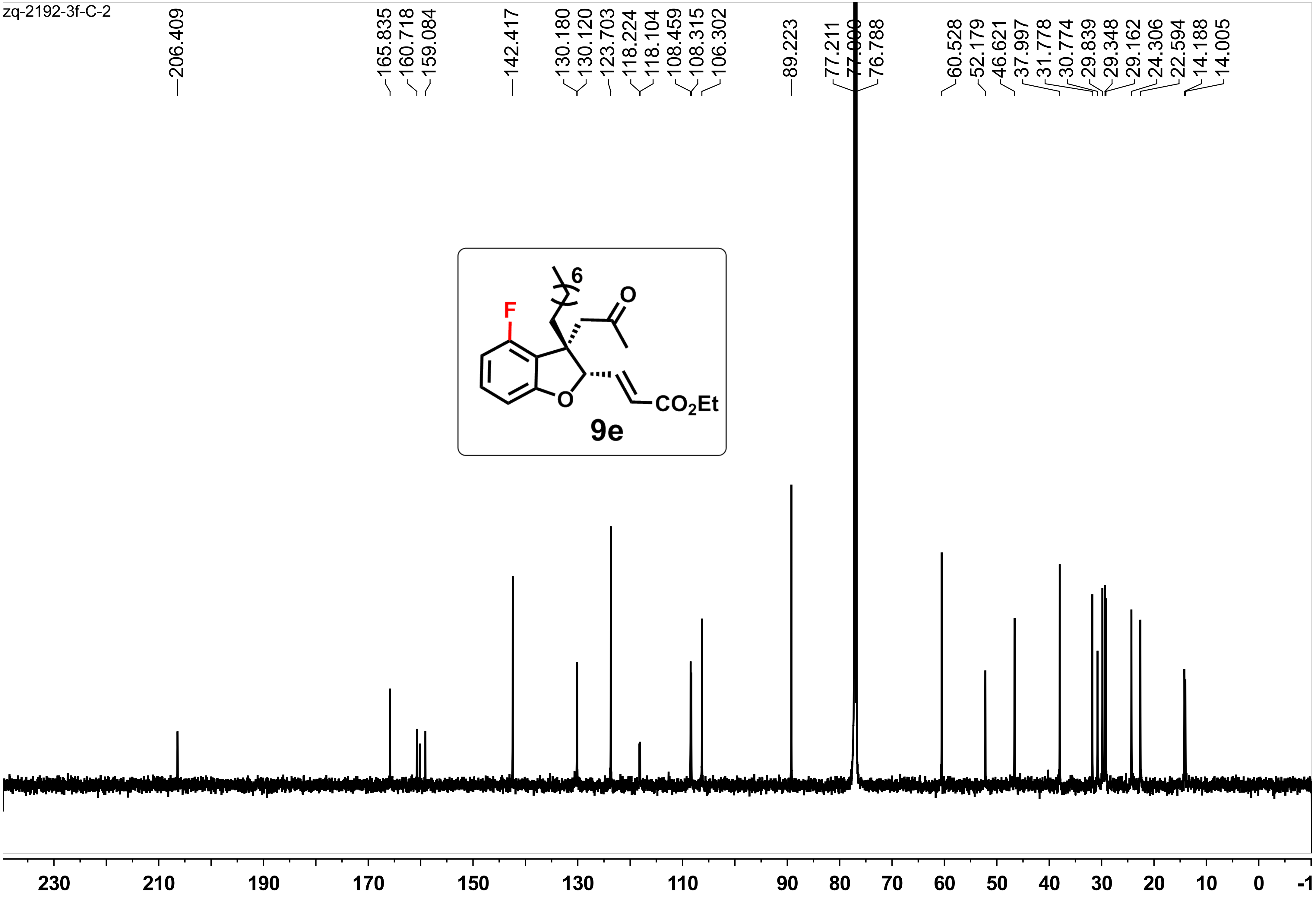

Supplementary Figure 108. ^{13}C NMR of **9e**

zq-2192-3f-F

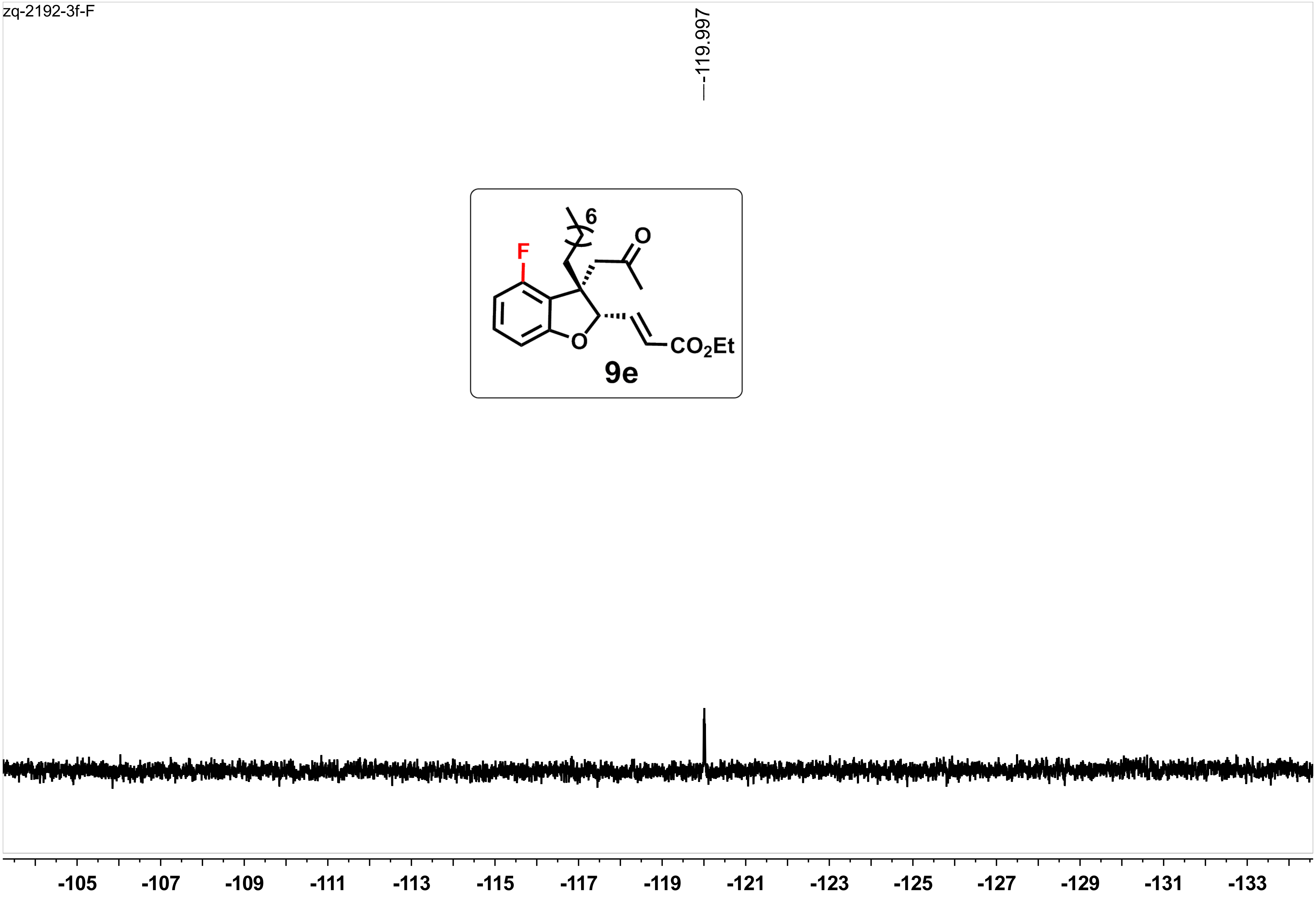

Supplementary Figure 109. ^{19}F NMR of **9e**

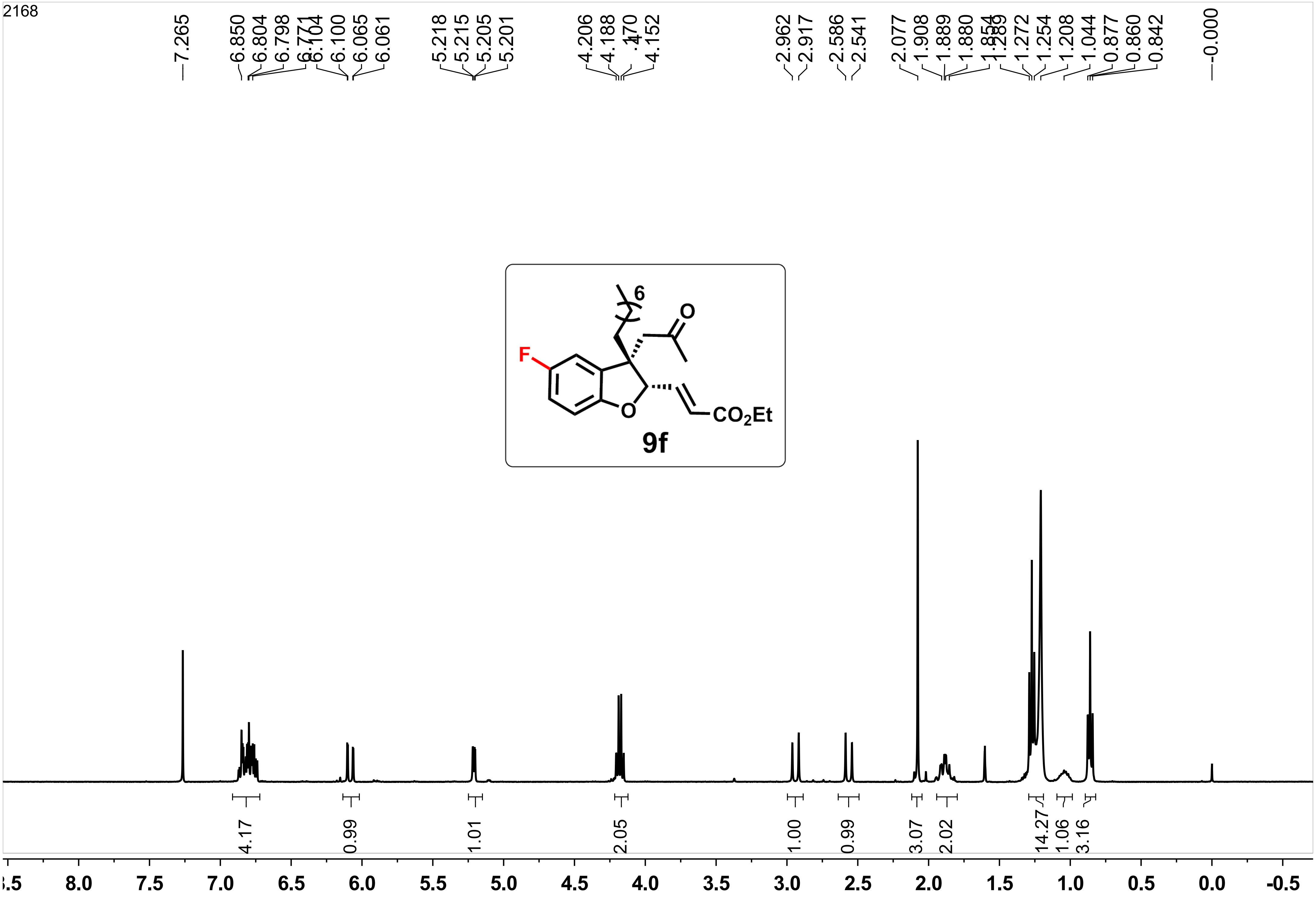

Supplementary Figure 110. ^{1}H NMR of **9f**

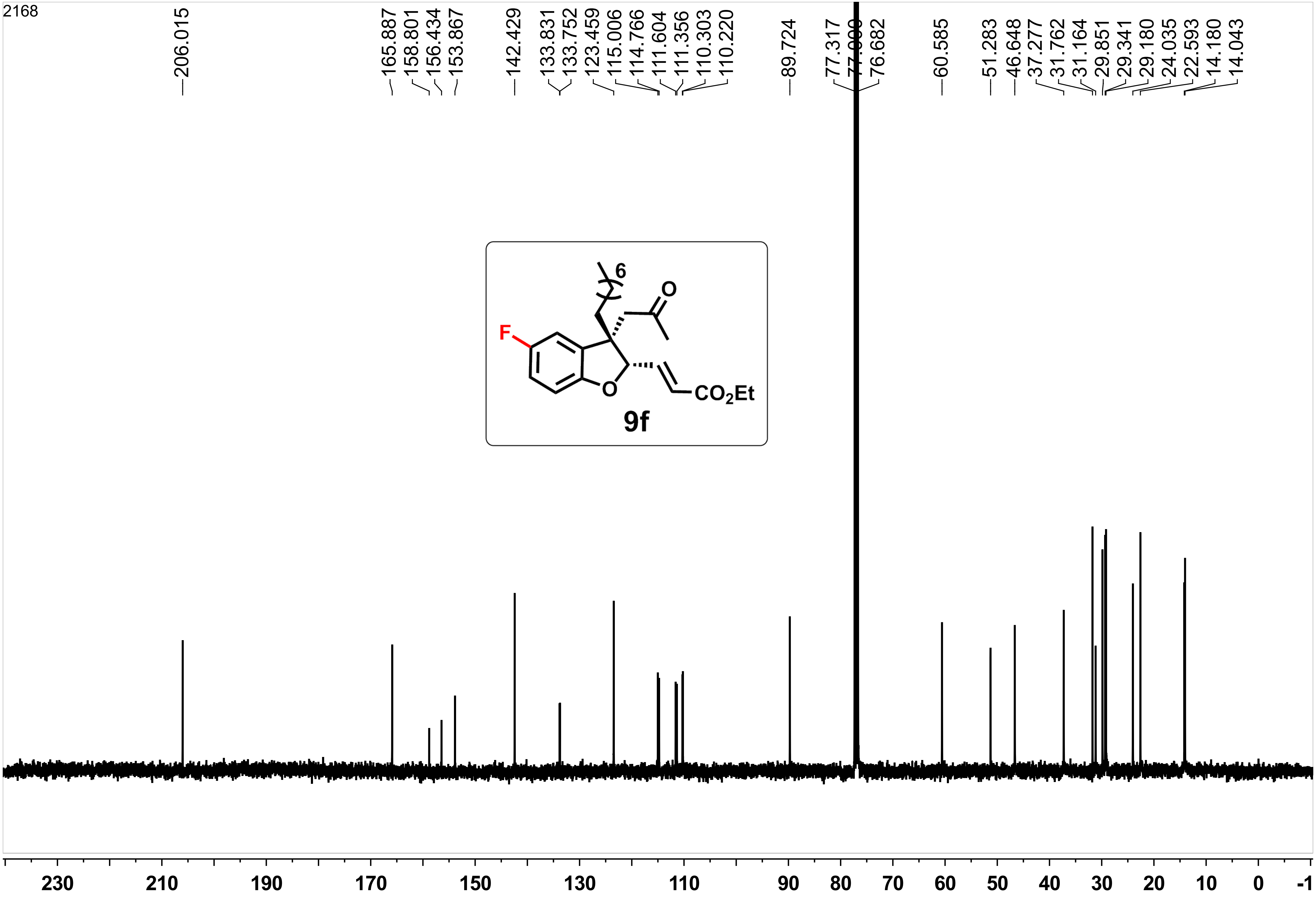

Supplementary Figure 111. ^{13}C NMR of **9f**

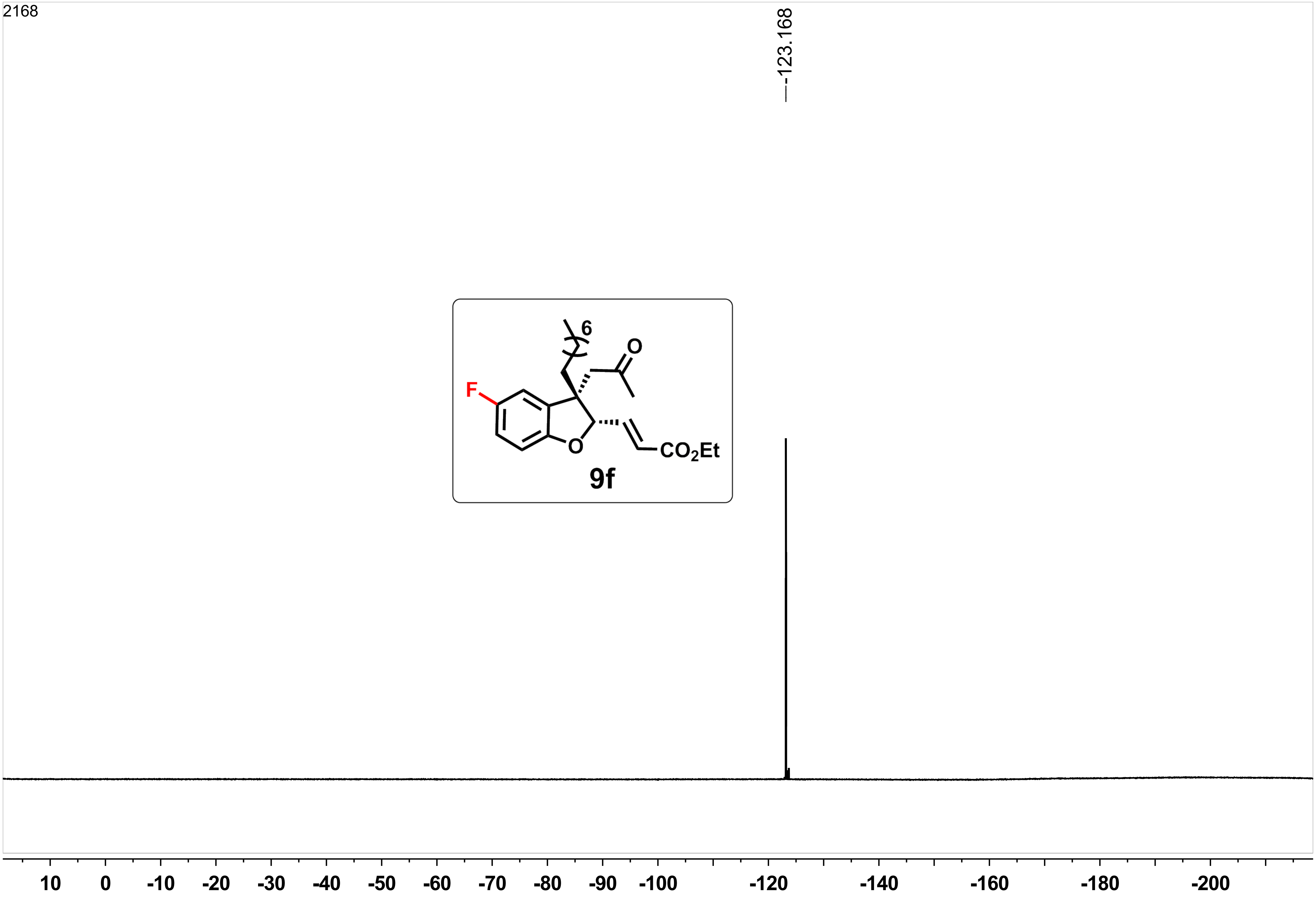

Supplementary Figure 112. ^{19}F NMR of **9f**

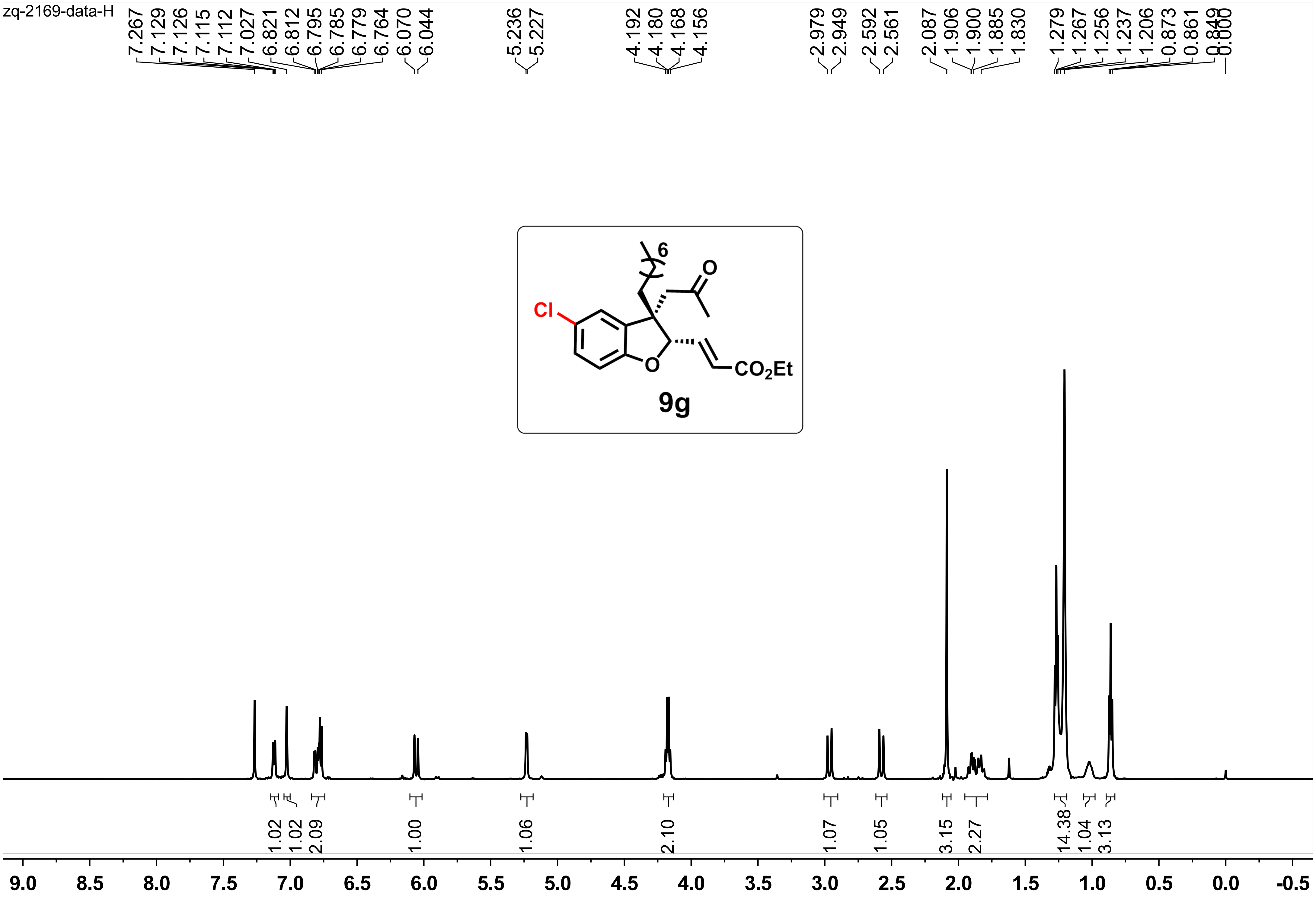

Supplementary Figure 113. ^{1}H NMR of **9g**

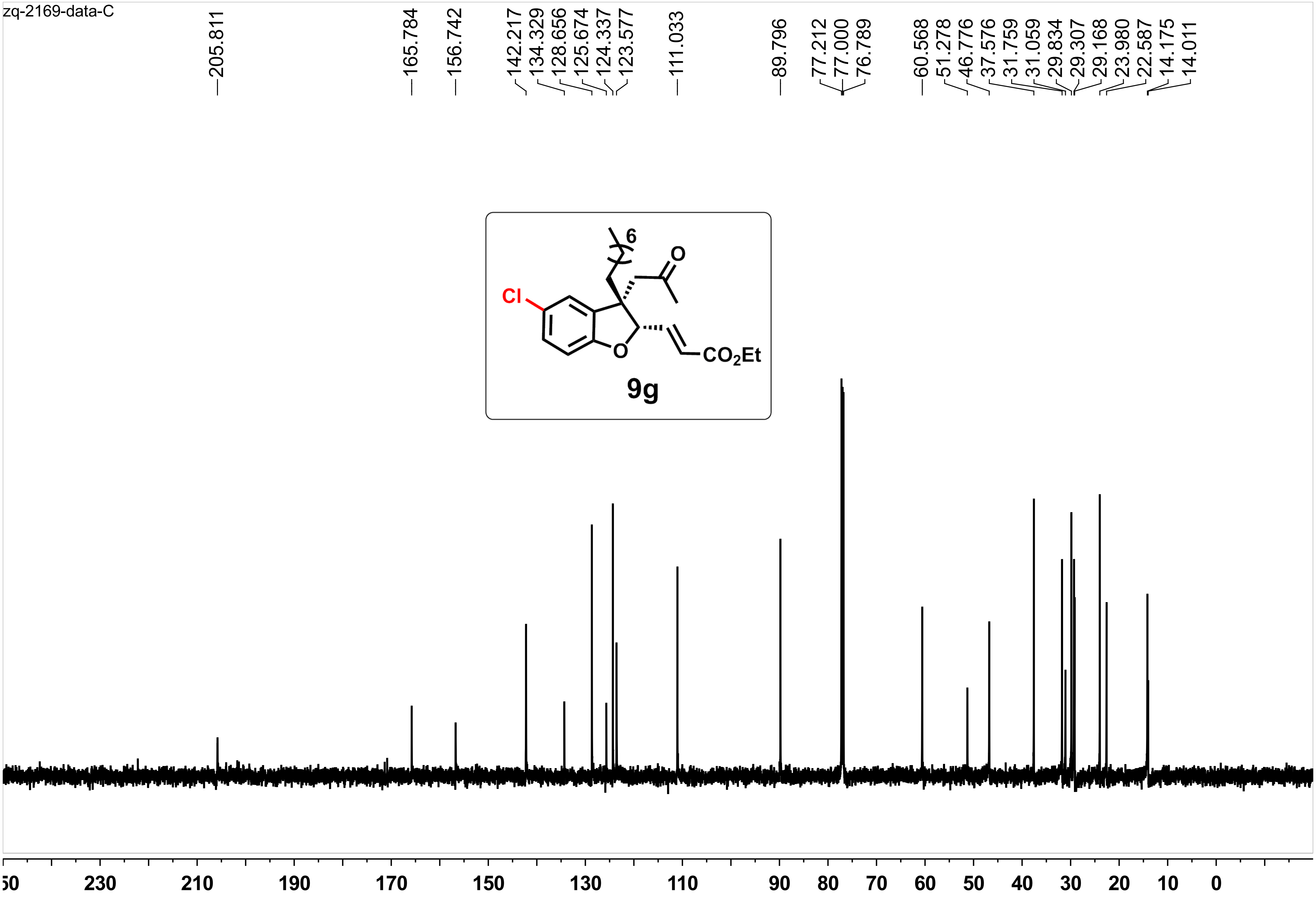

Supplementary Figure 114. ^{13}C NMR of **9g**

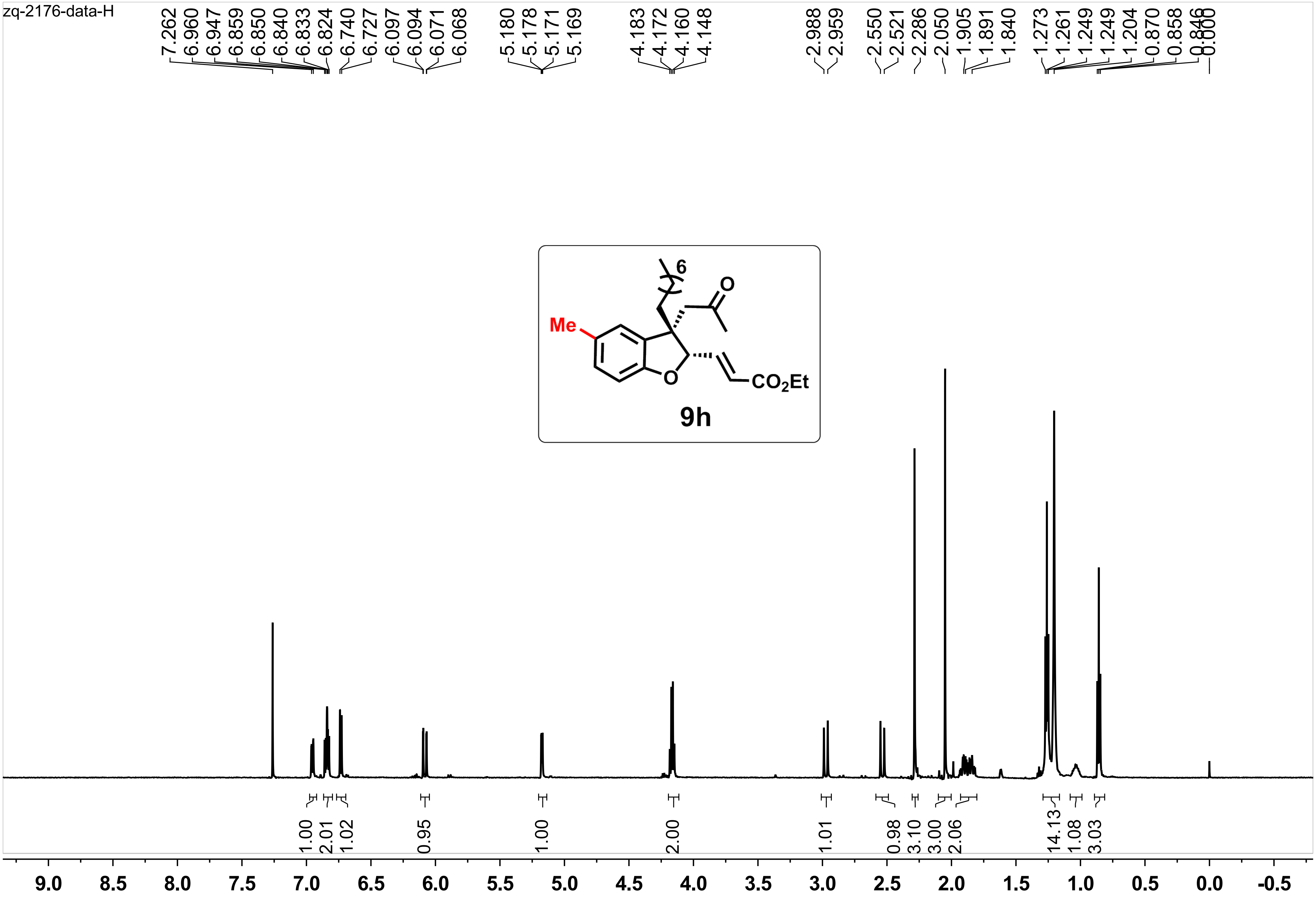

Supplementary Figure 115. ^{1}H NMR of **9h**

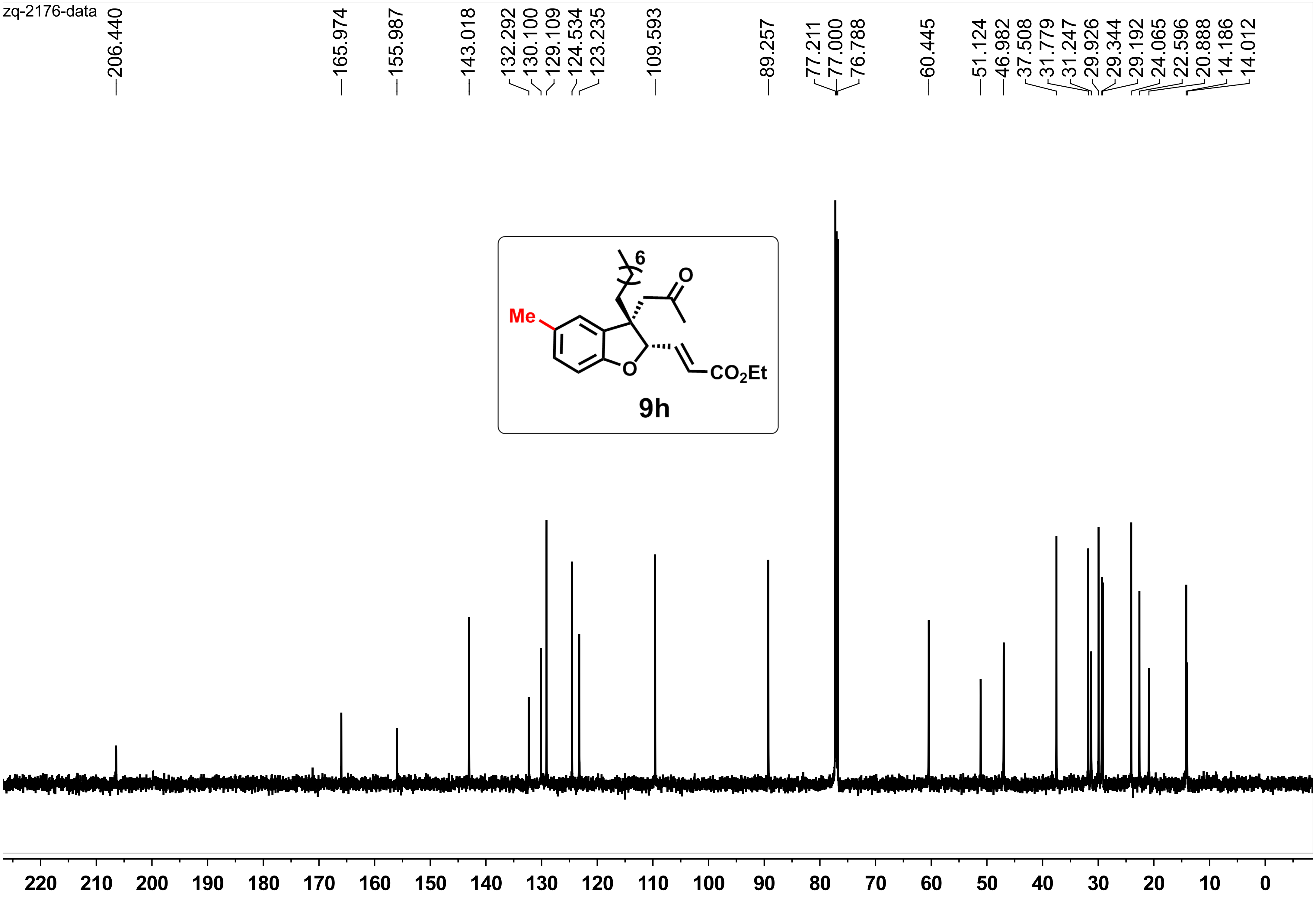

Supplementary Figure 116. ^{13}C NMR of **9h**

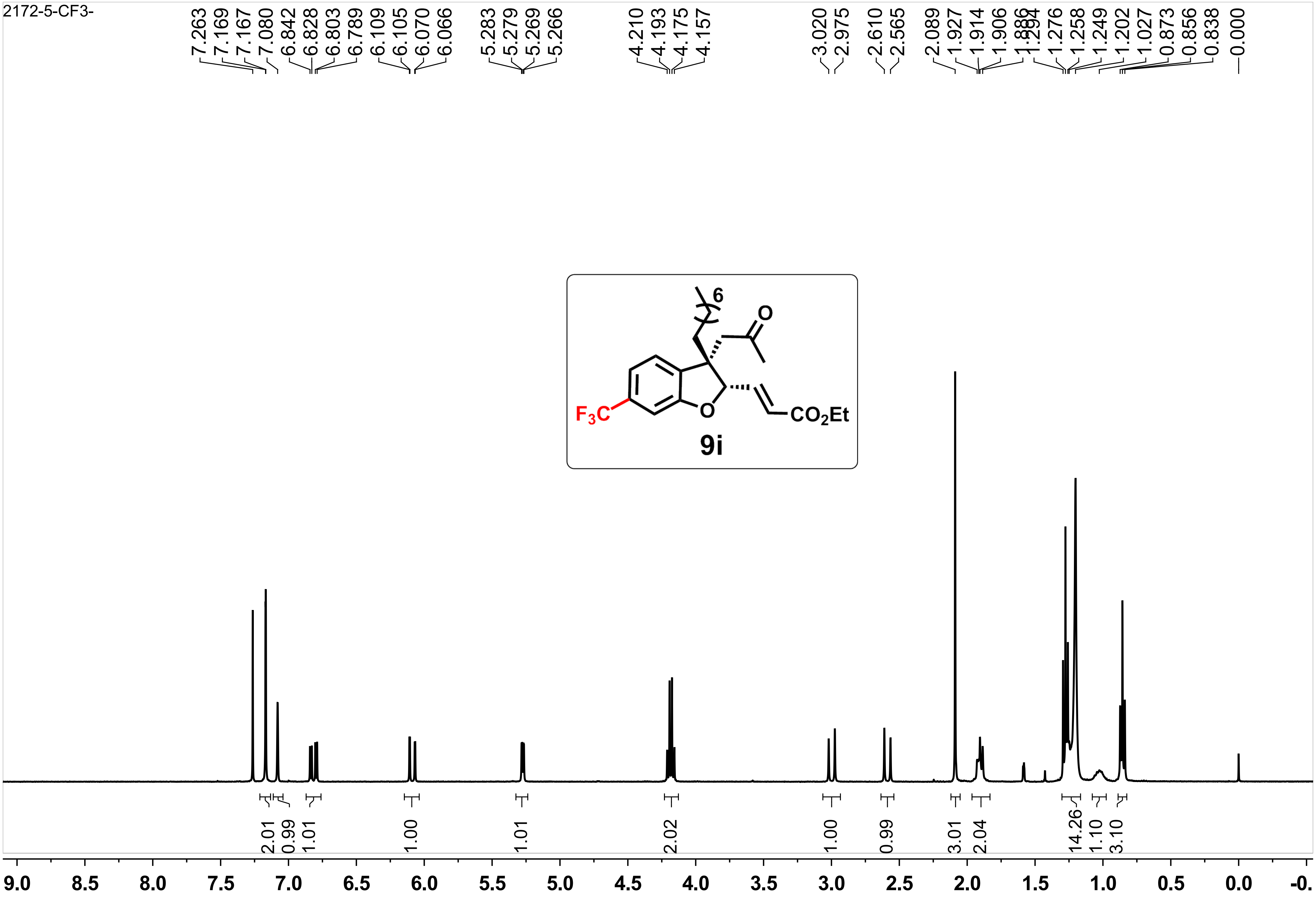

Supplementary Figure 117. ^{1}H NMR of **9i**

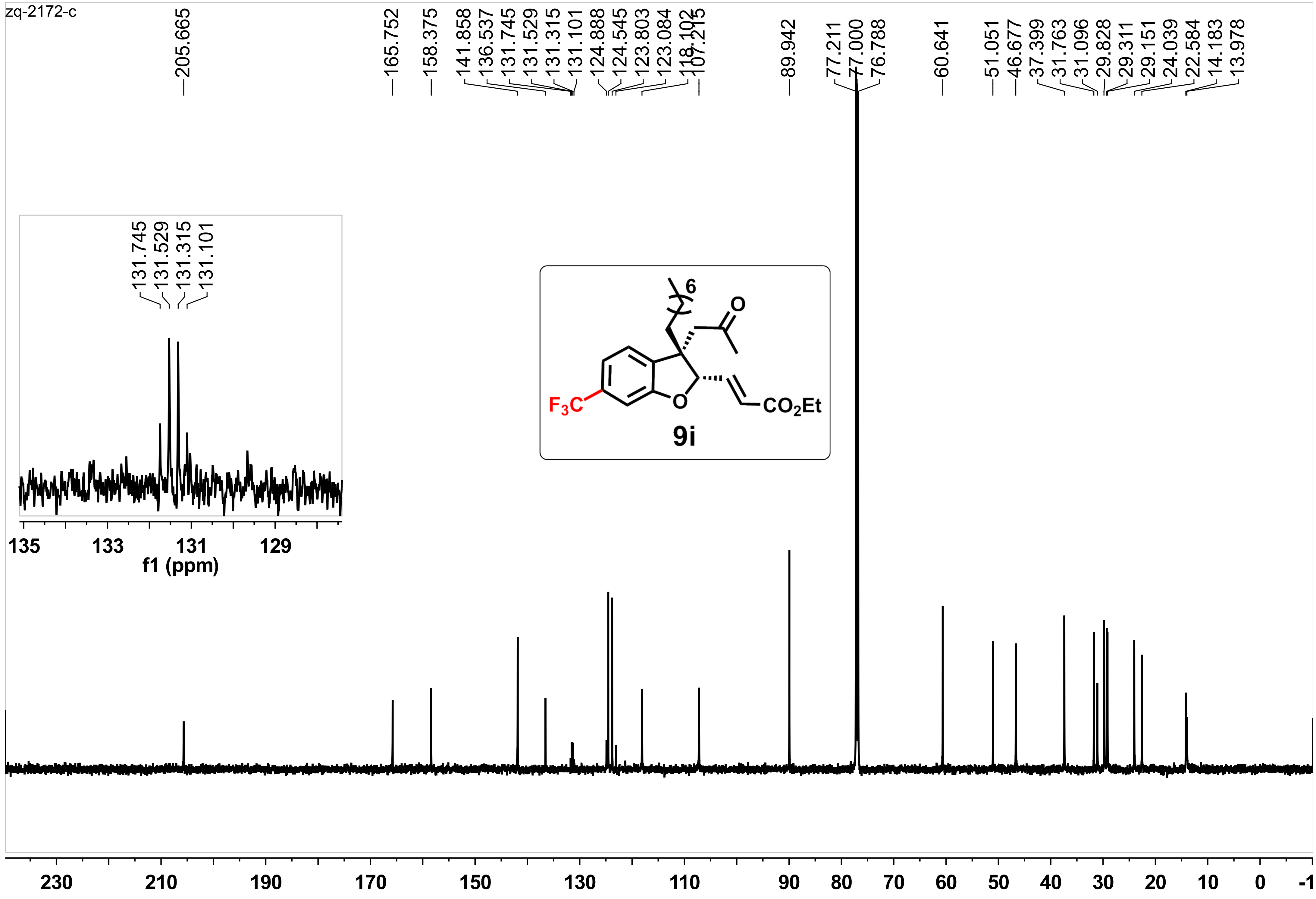

Supplementary Figure 118. ^{13}C NMR of **9i**

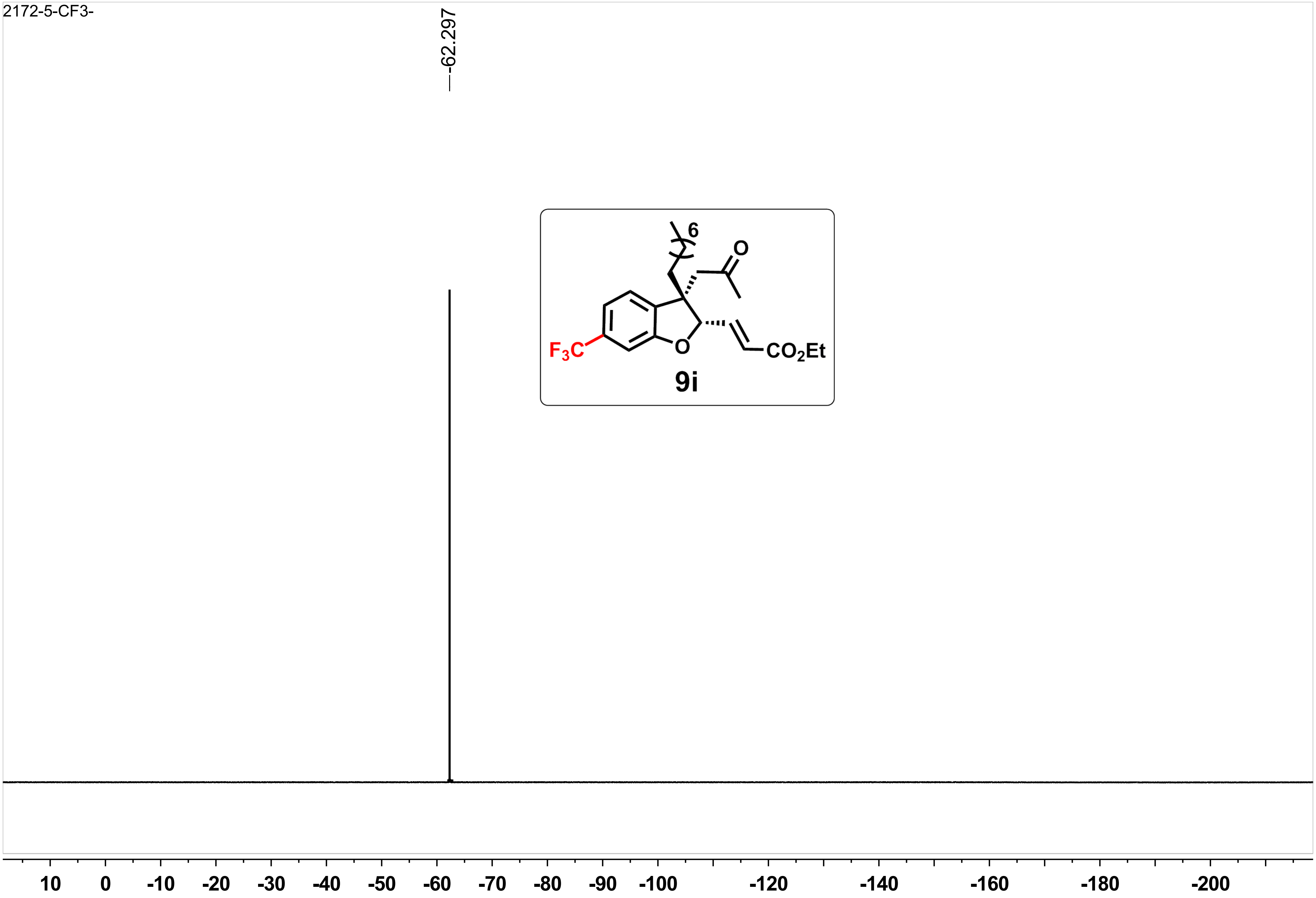

Supplementary Figure 119. ^{19}F NMR of **9i**

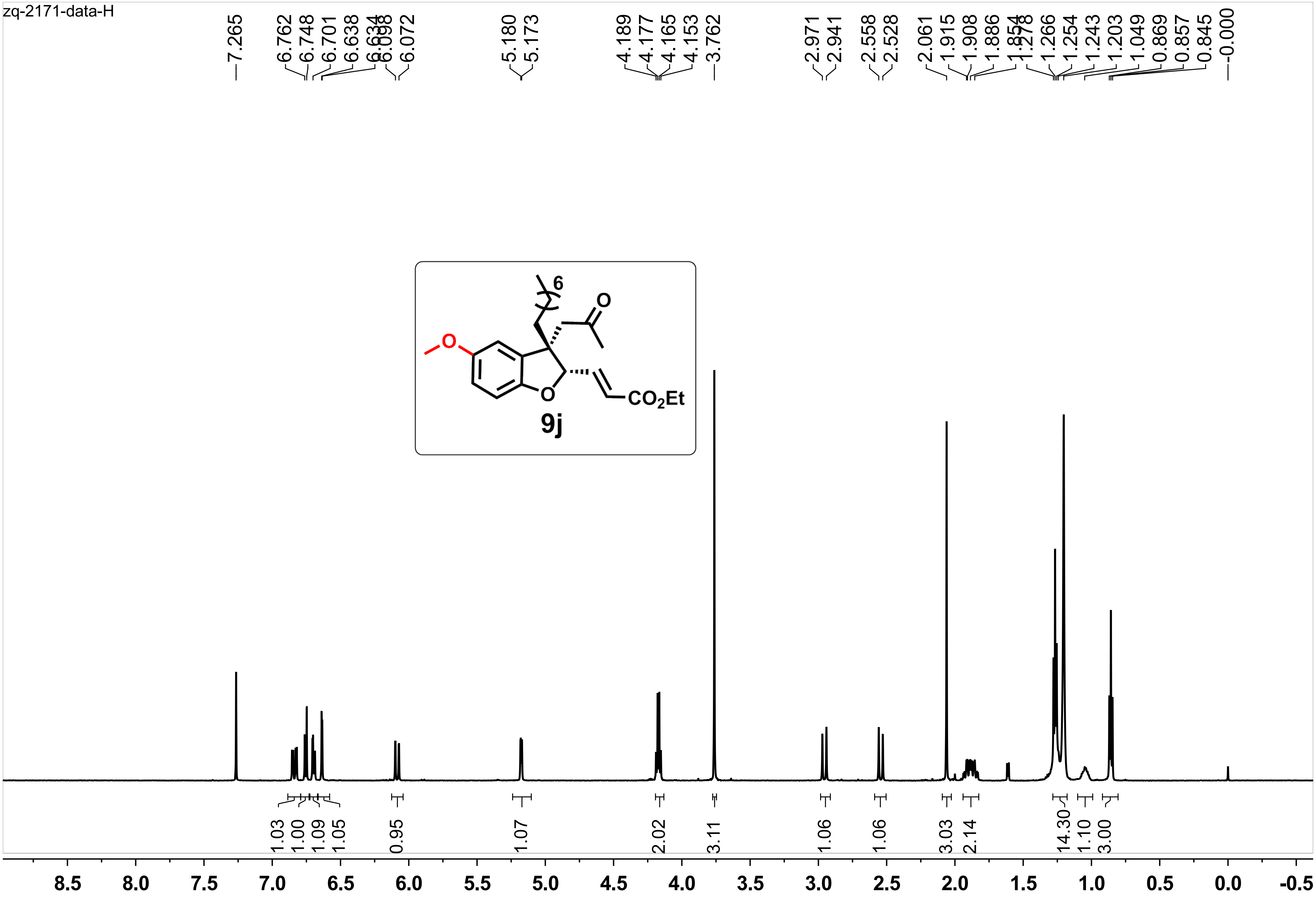

Supplementary Figure 120. ^{1}H NMR of **9j**

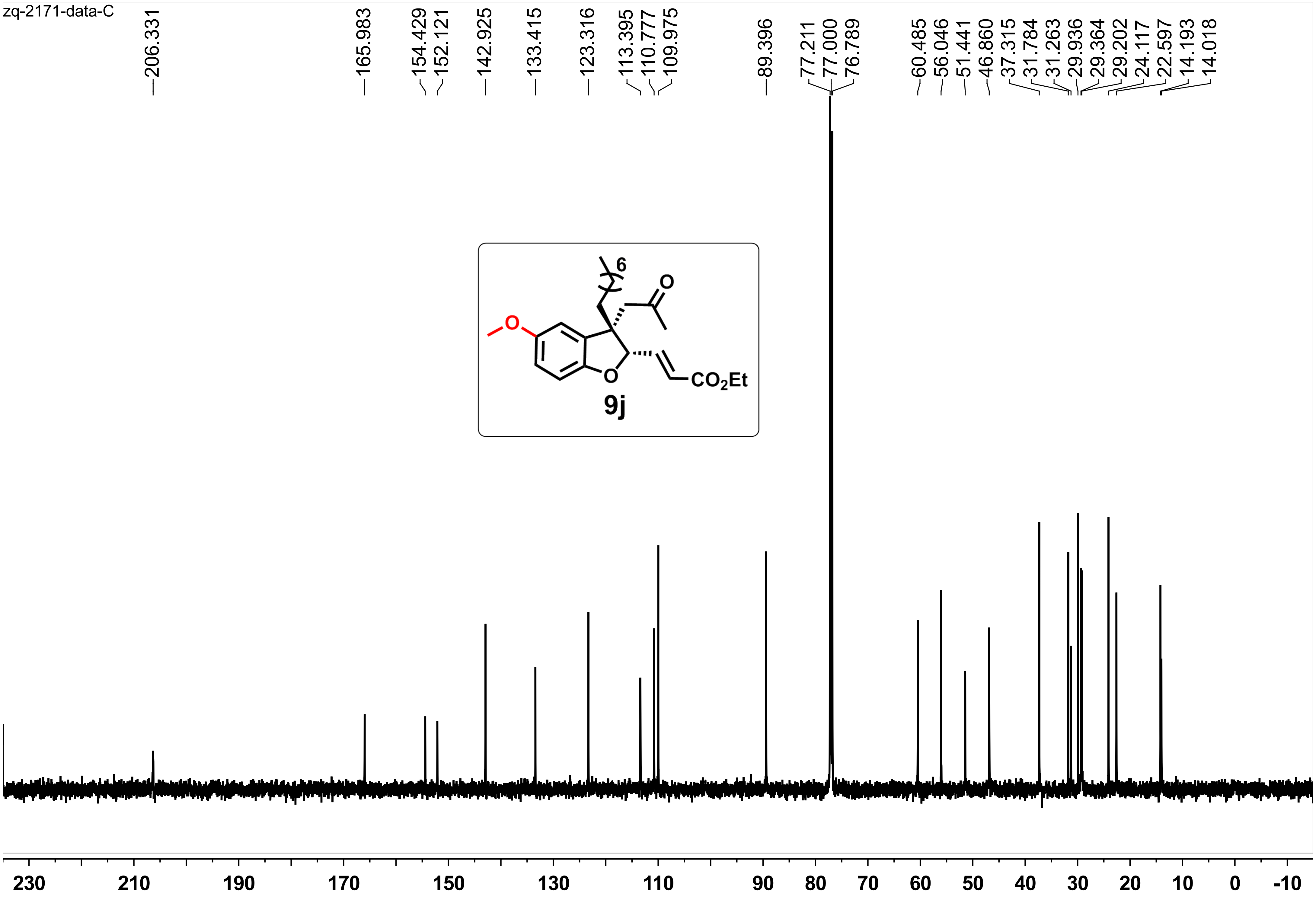

Supplementary Figure 121. ^{13}C NMR of **9j**

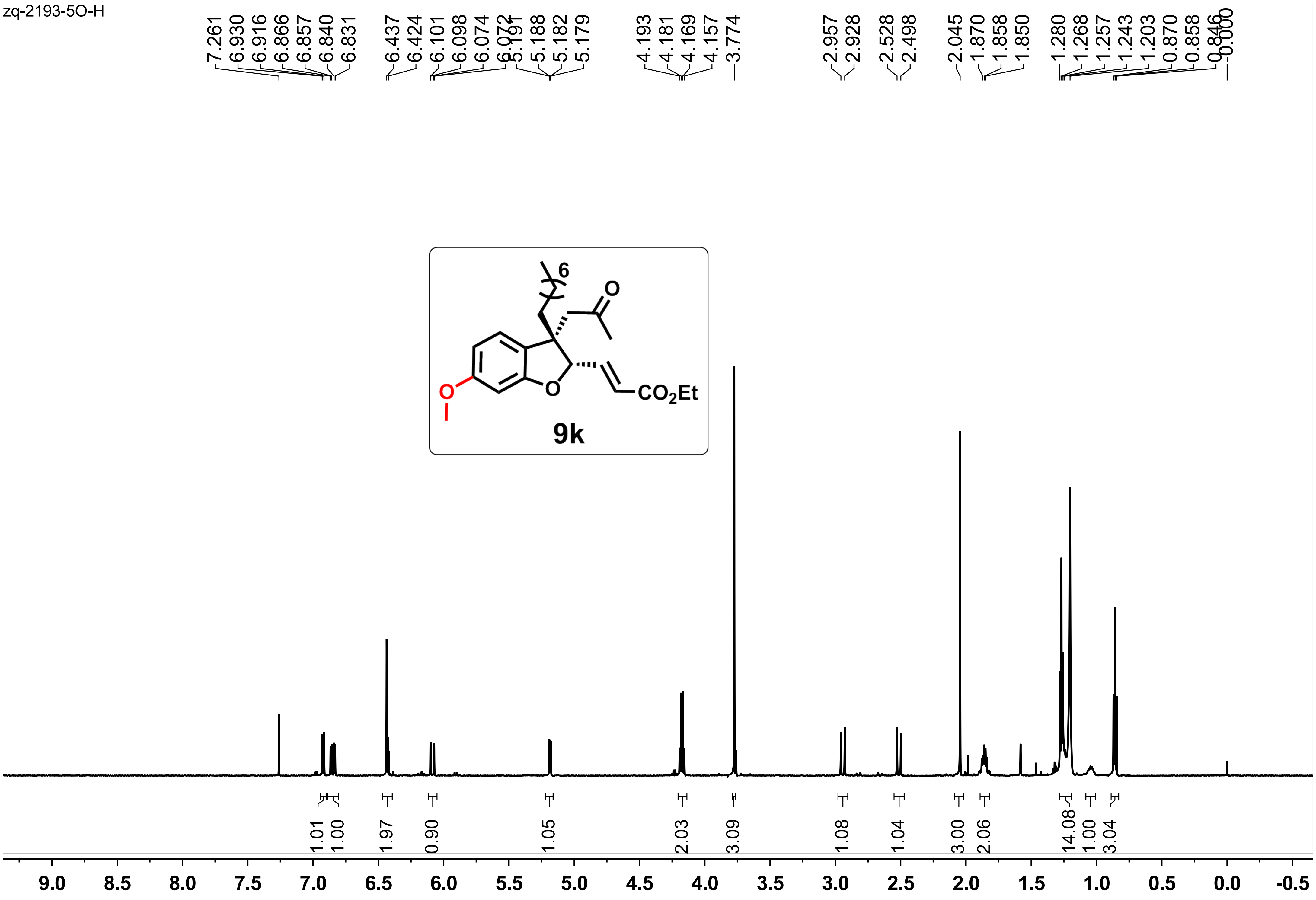

Supplementary Figure 122. ^{1}H NMR of **9k**

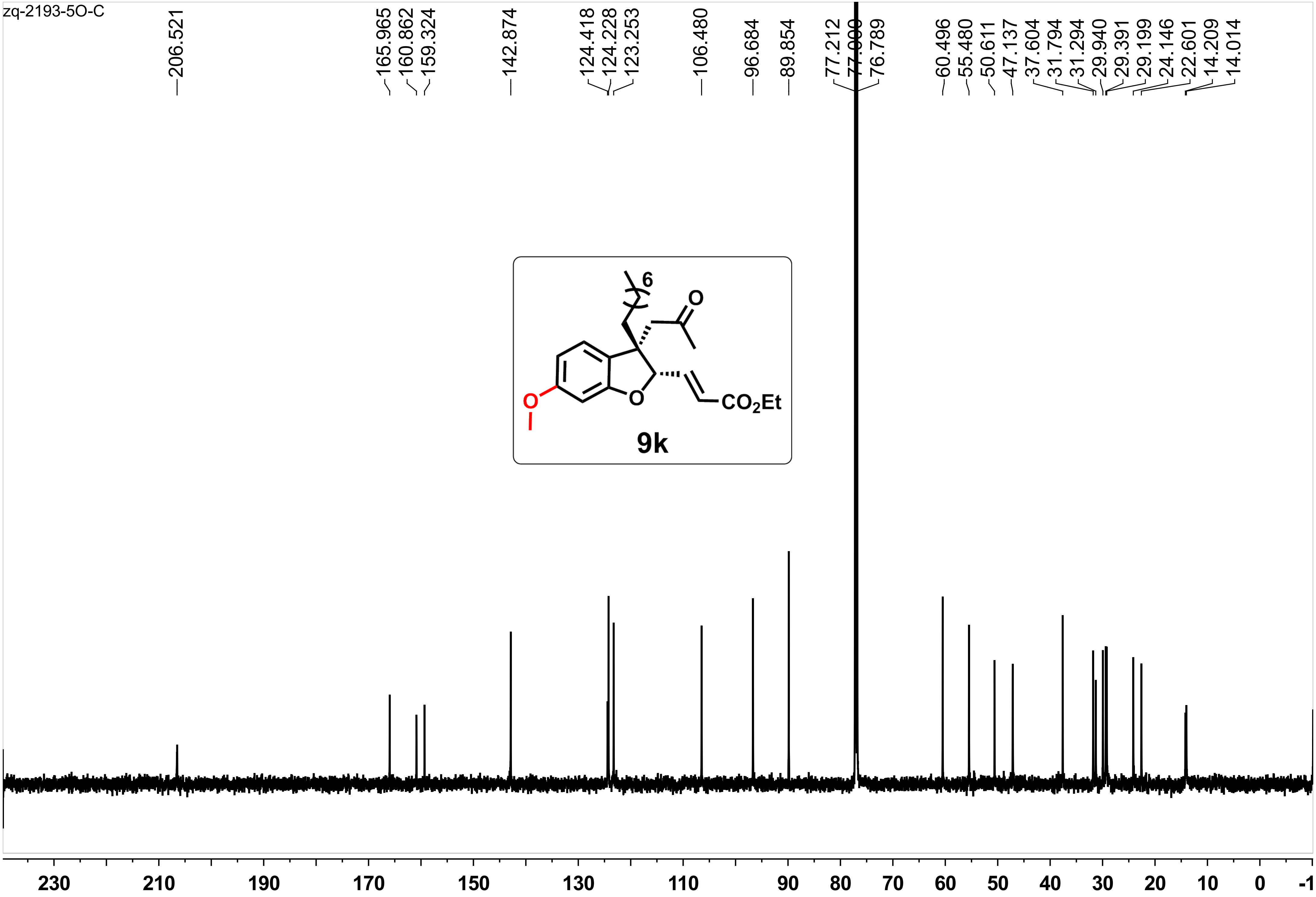

Supplementary Figure 123. ^{13}C NMR of **9k**

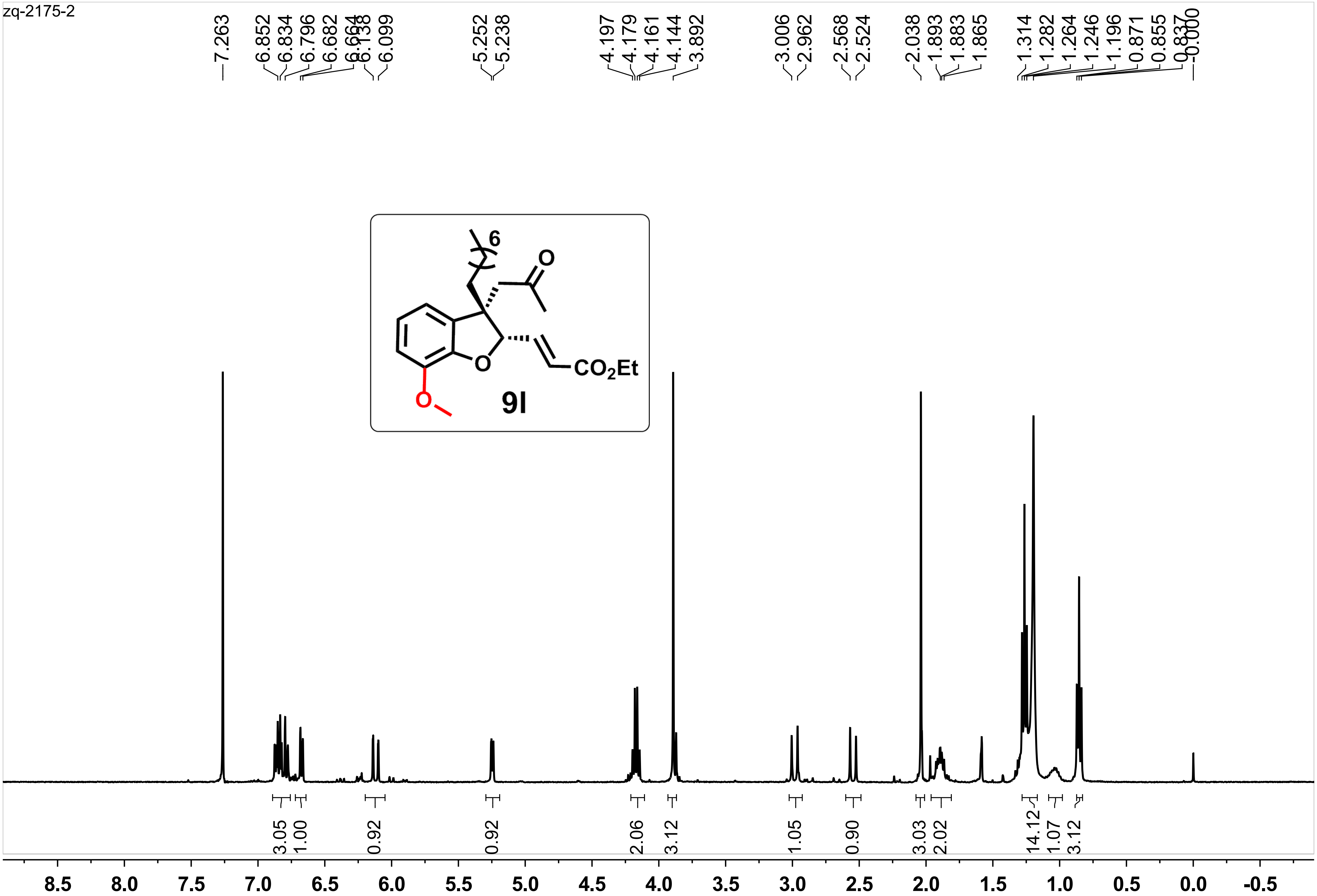

Supplementary Figure 124. ^{1}H NMR of **9l**

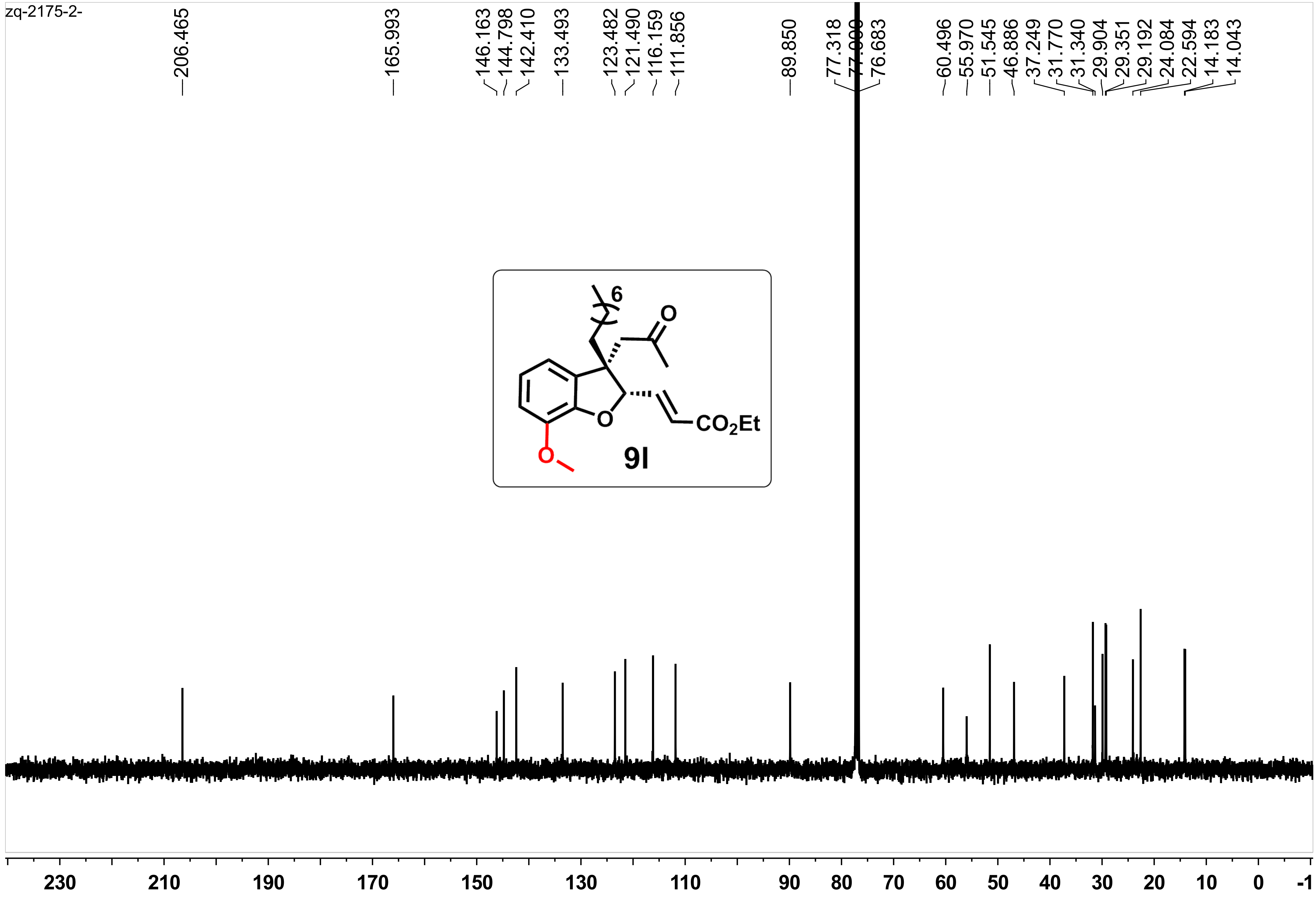

Supplementary Figure 125. ^{13}C NMR of **9l**

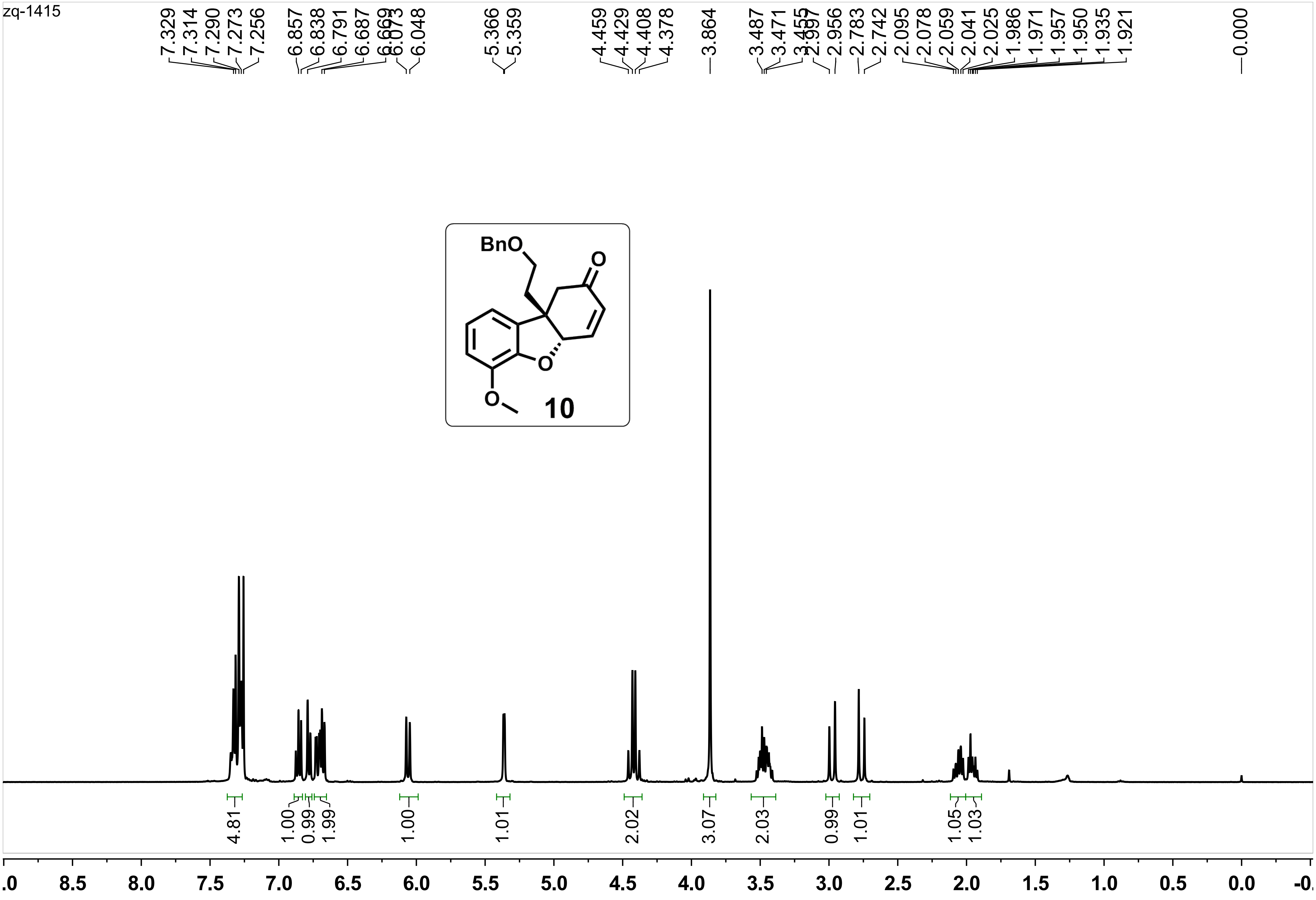

Supplementary Figure 126. ^{1}H NMR of **10**

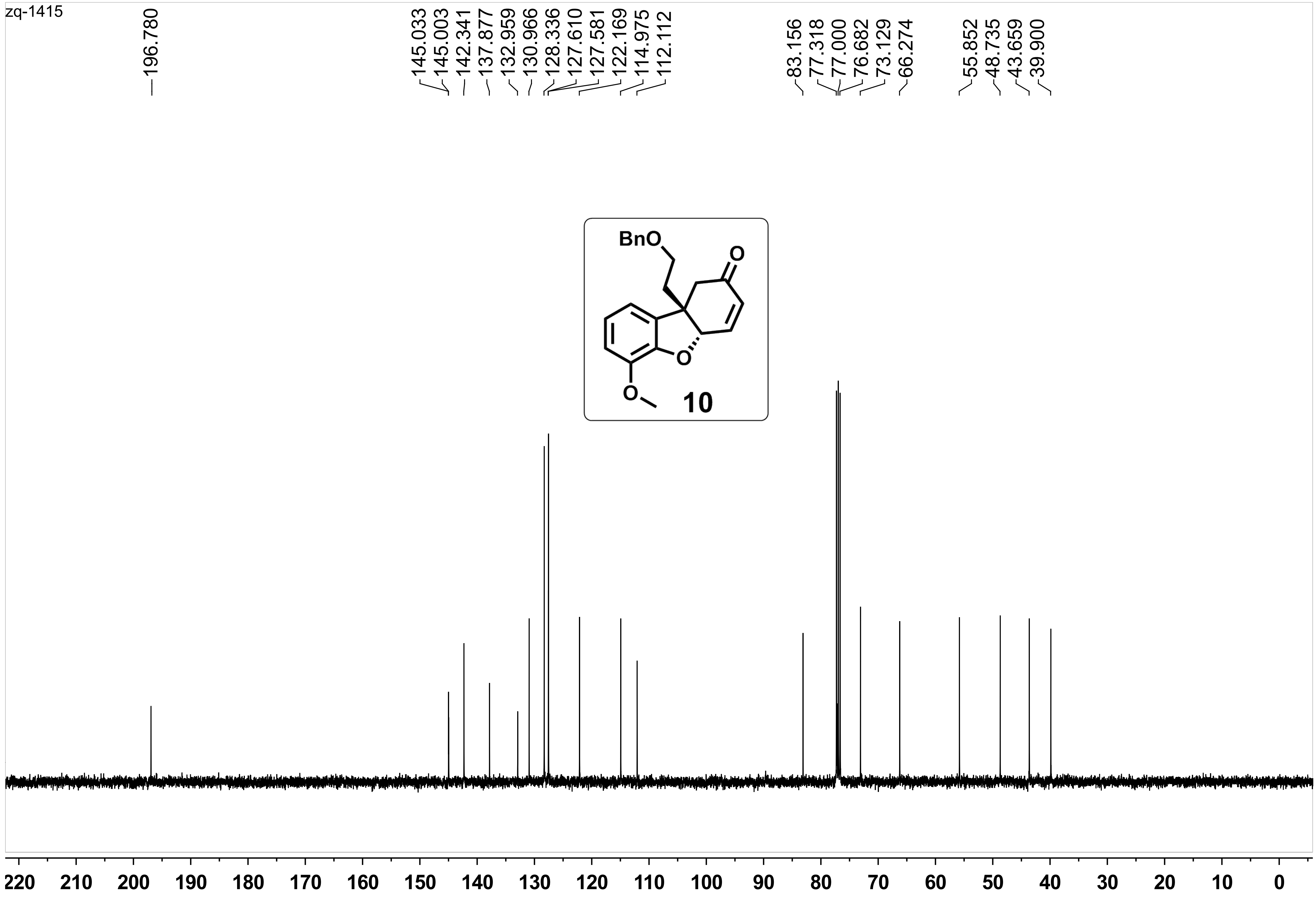

Supplementary Figure 127. ^{13}C NMR of **10**

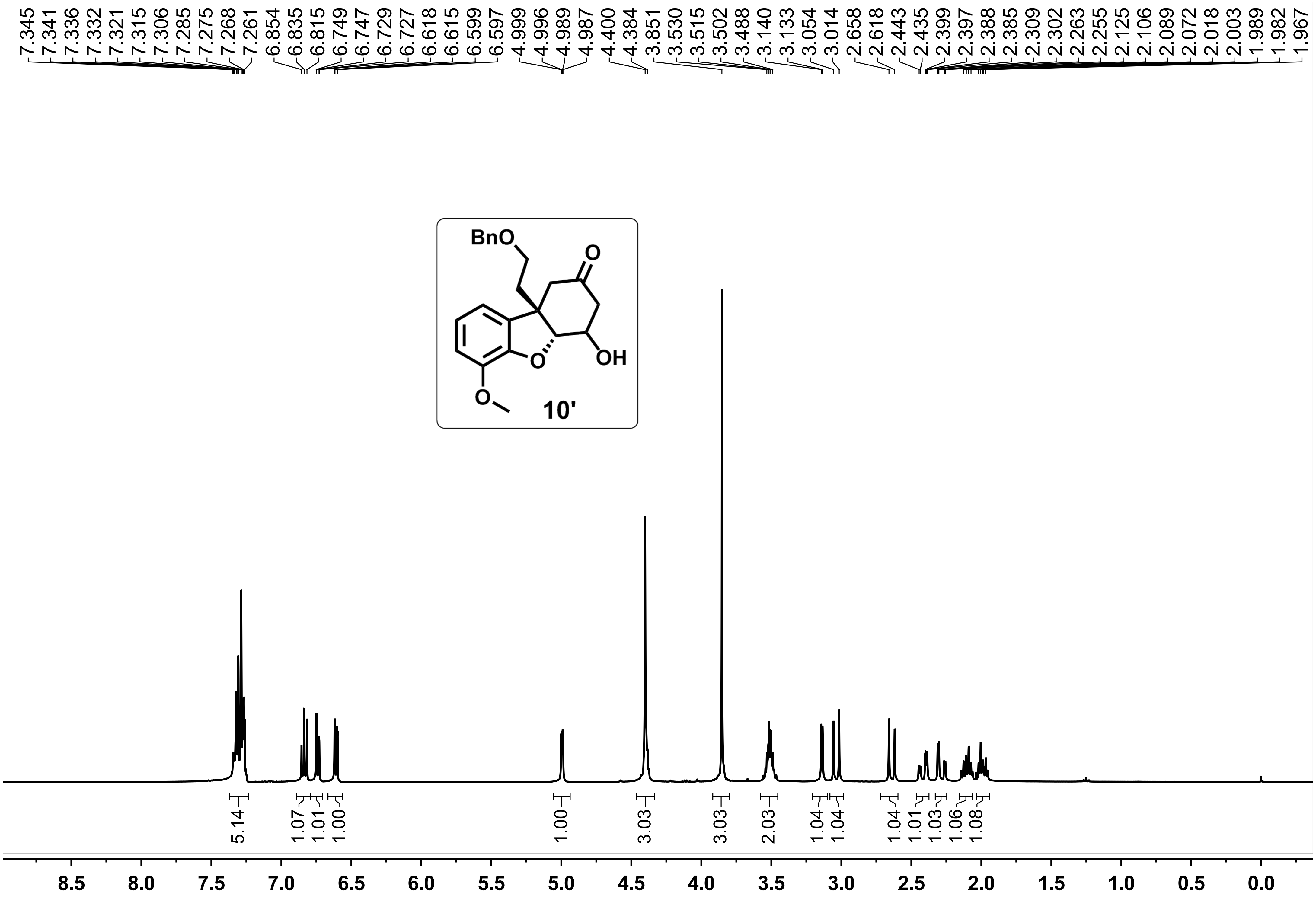

Supplementary Figure 128. ^{1}H NMR of **10'**

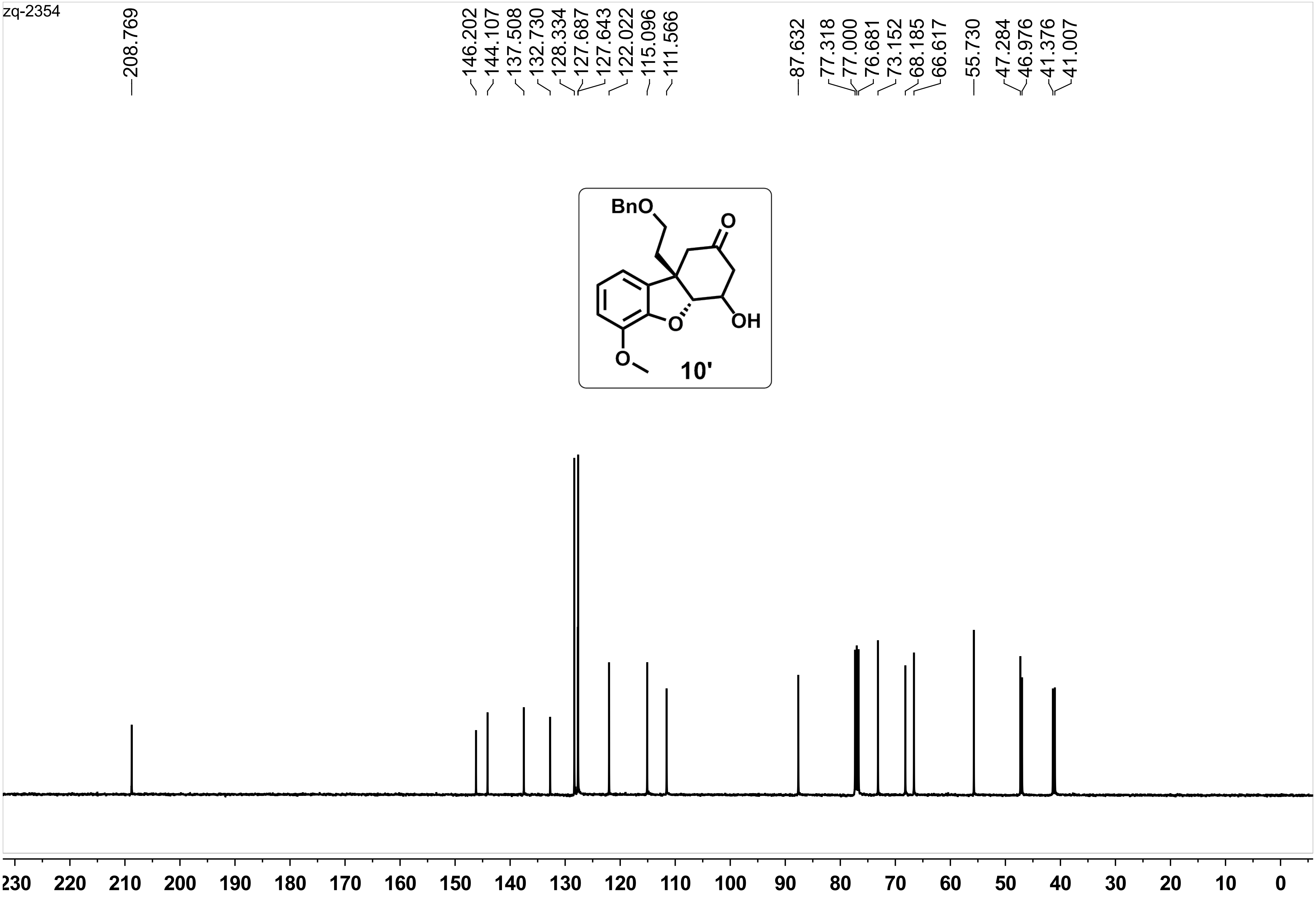

Supplementary Figure 129. ^{13}C NMR of **10'**

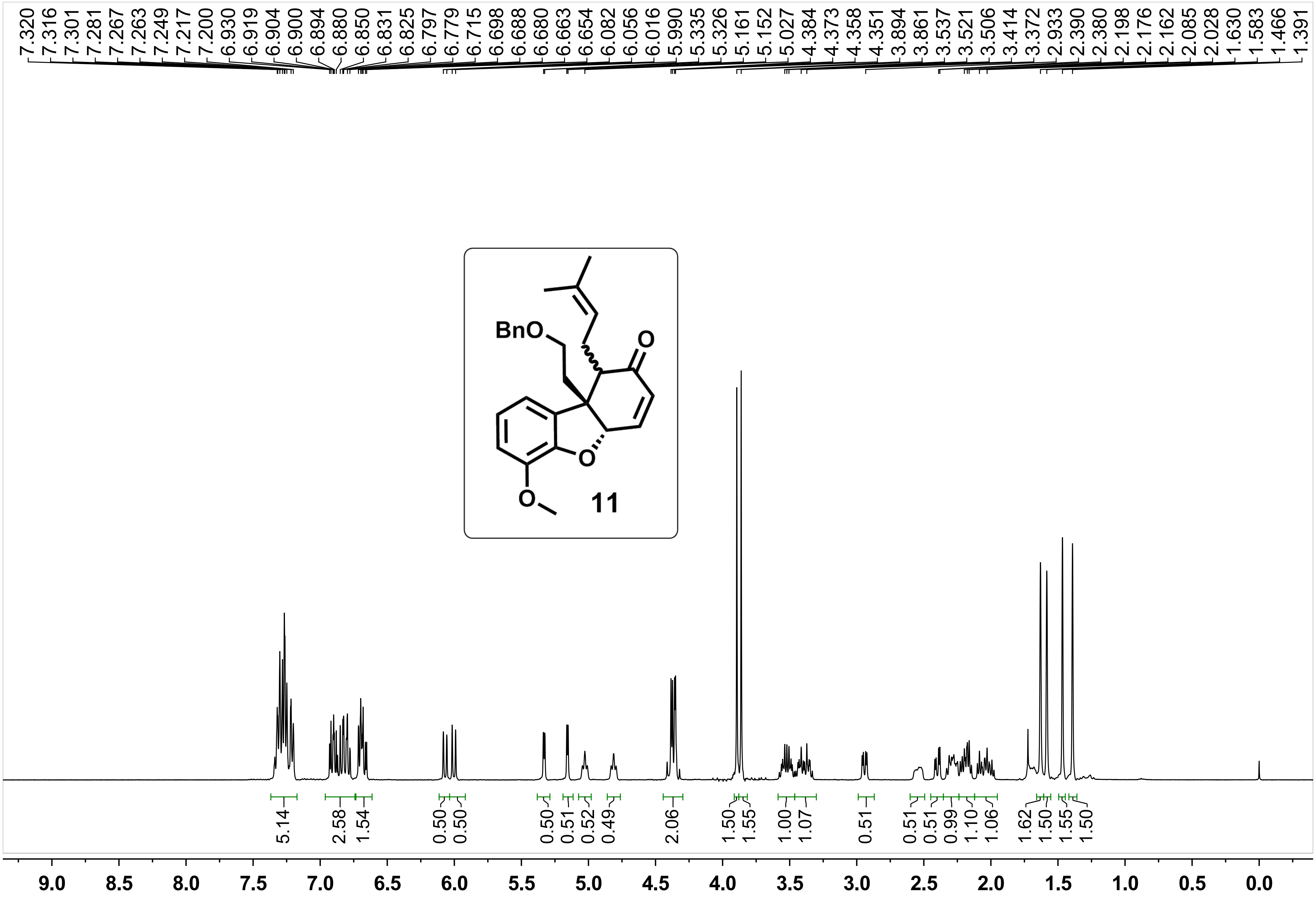

Supplementary Figure 130. ^{1}H NMR of **11**

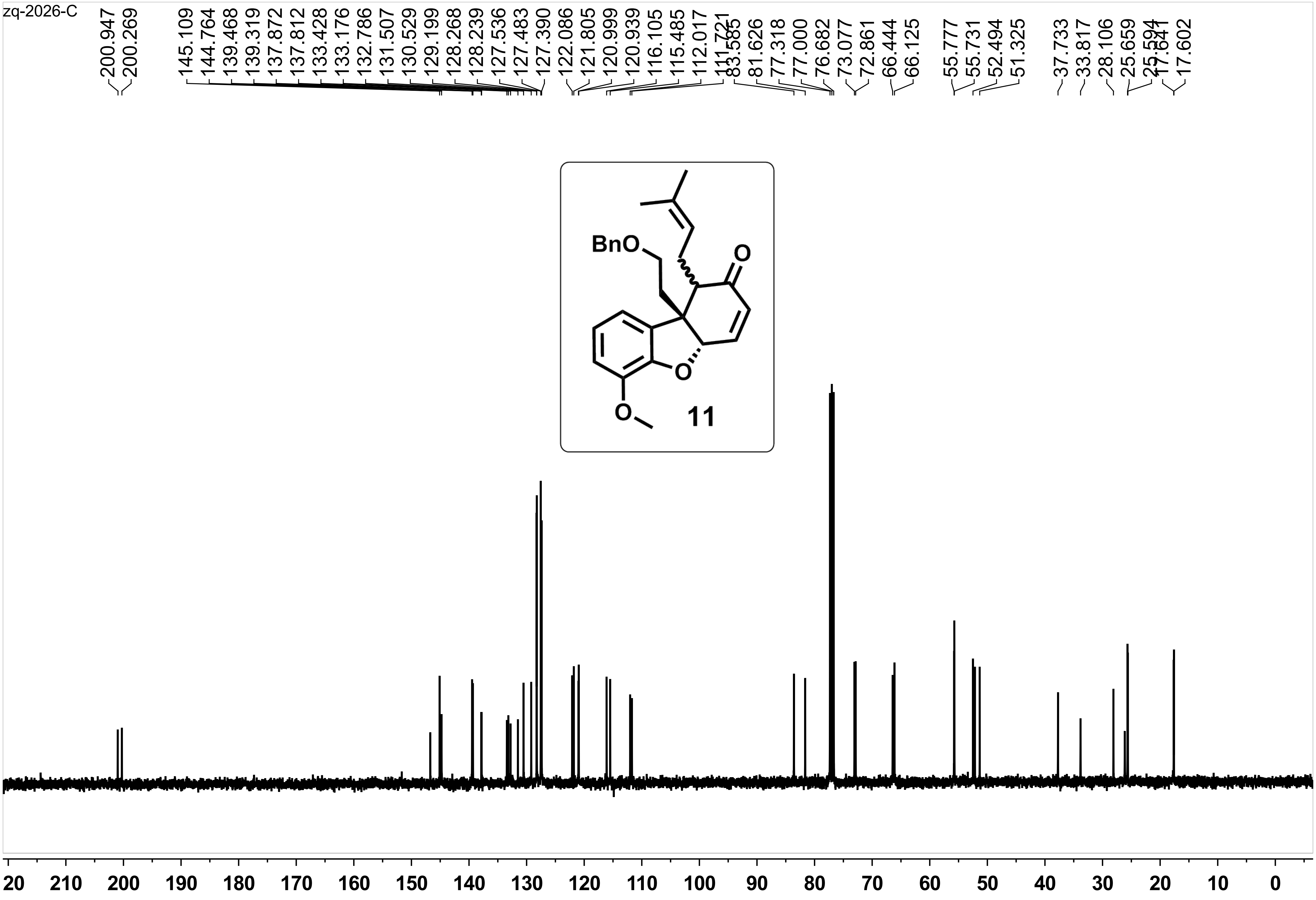

Supplementary Figure 131. ^{13}C NMR of **11**

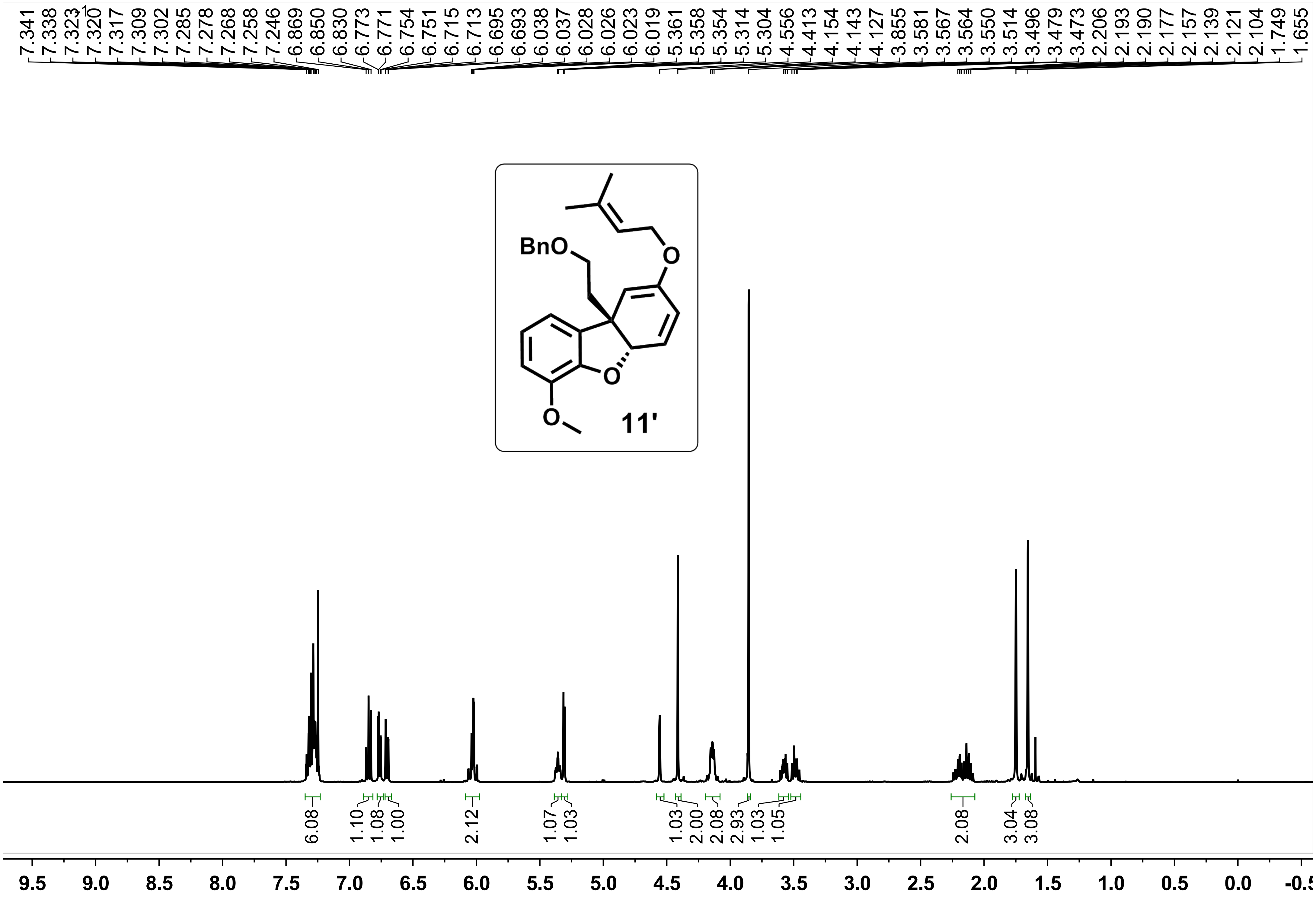

Supplementary Figure 132. ^{1}H NMR of **11'**

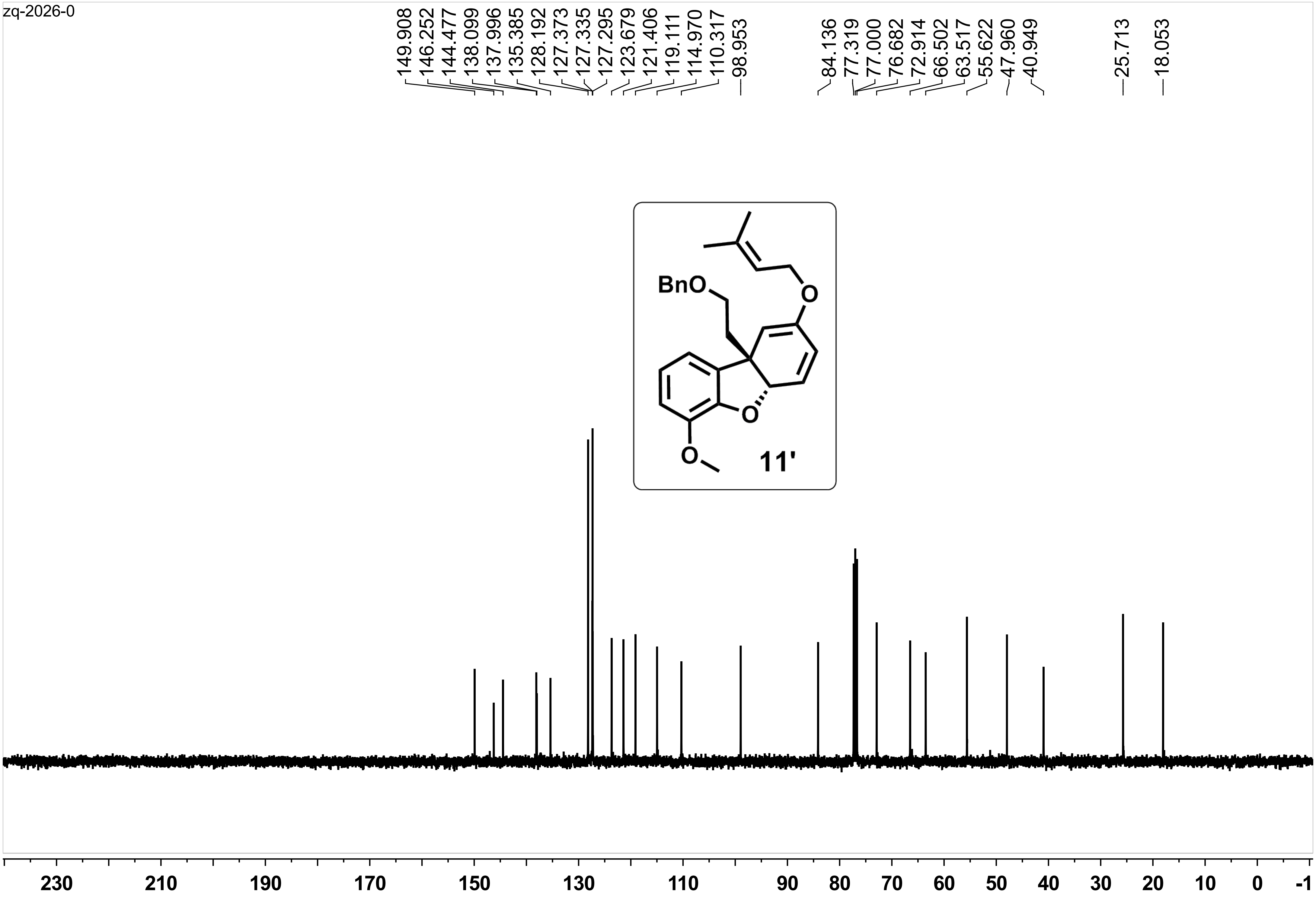

Supplementary Figure 133. ^{13}C NMR of **11'**

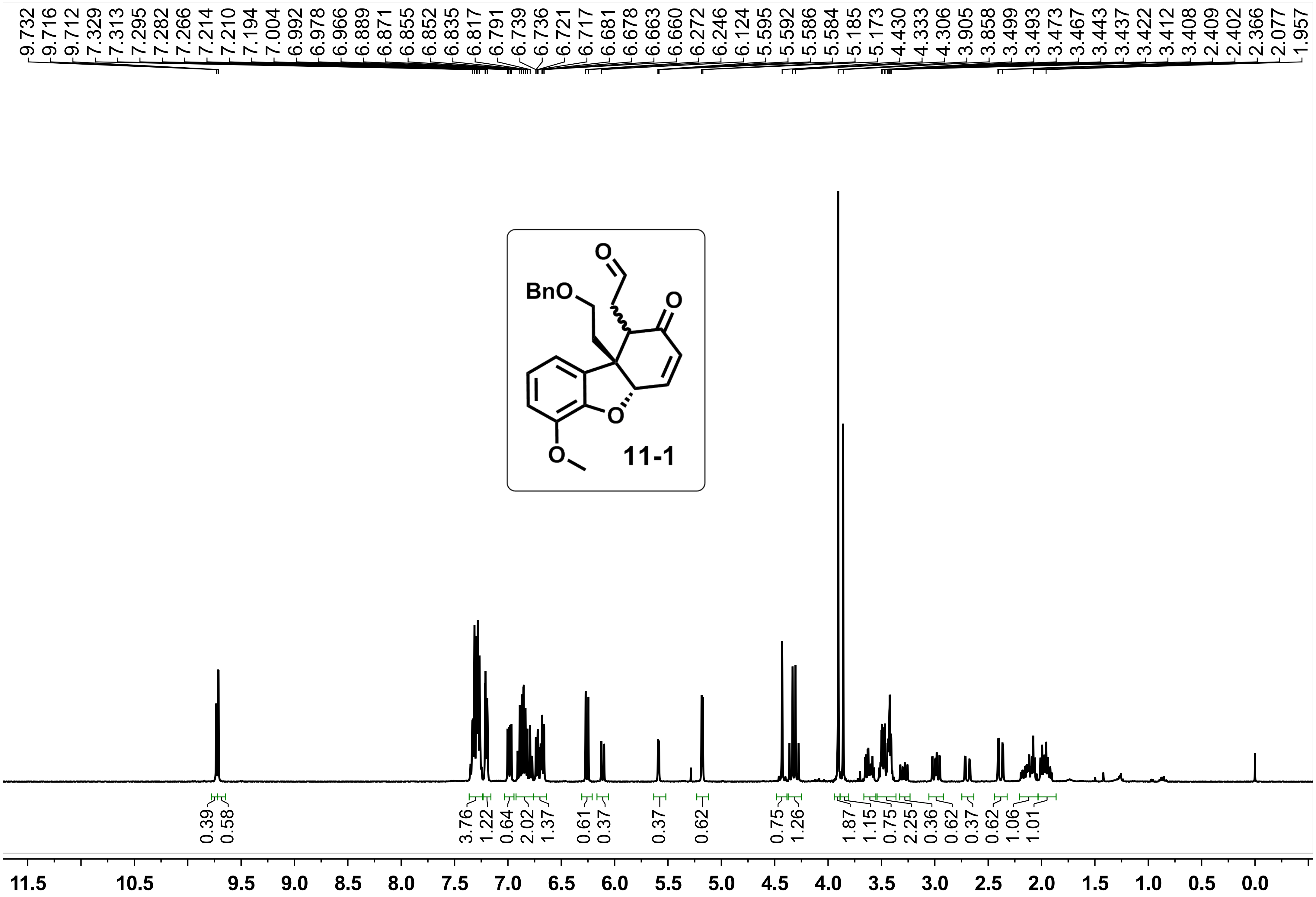

Supplementary Figure 134. ^{1}H NMR of **11-1**

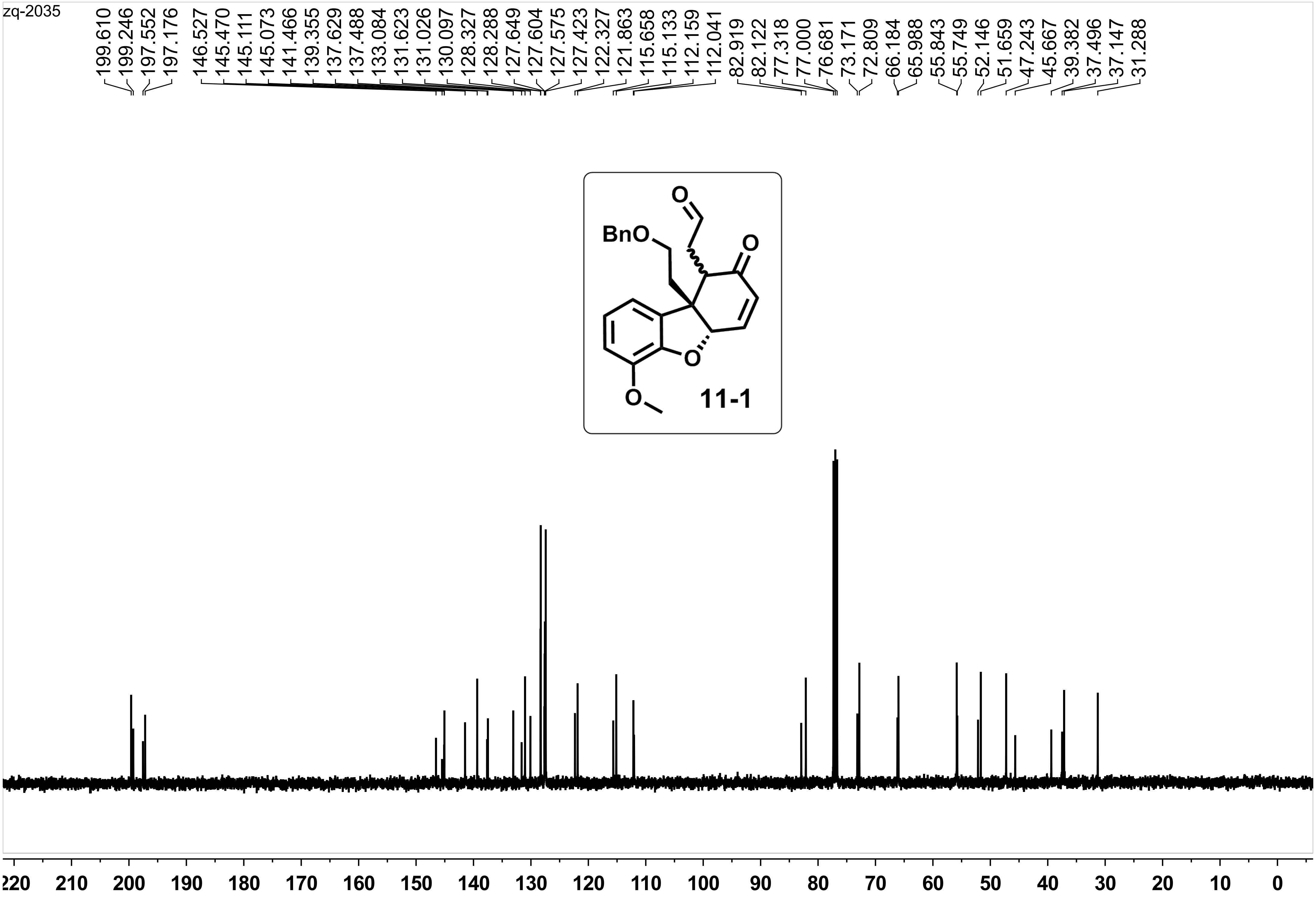

Supplementary Figure 135. ^{13}C NMR of **11-1**

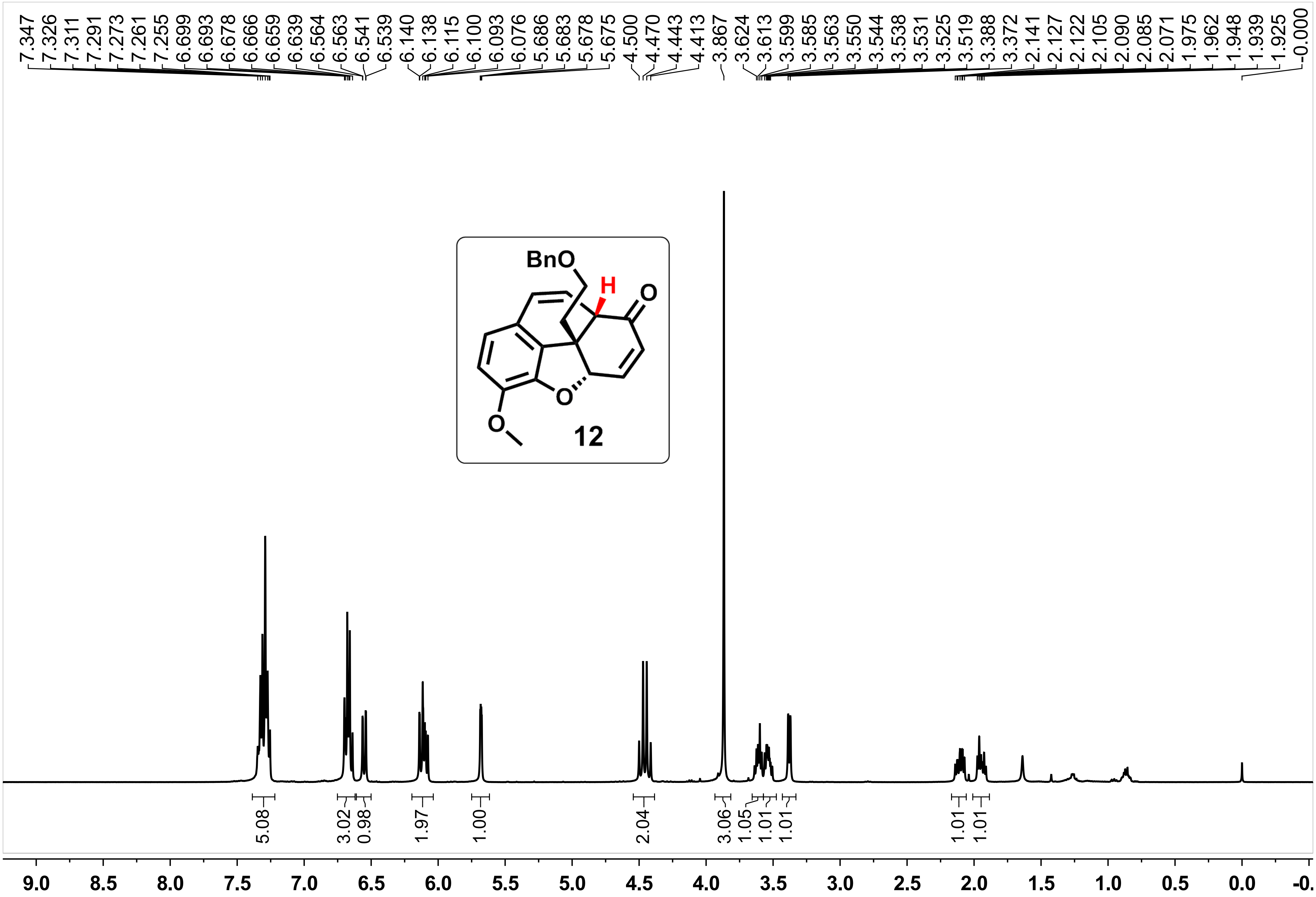

Supplementary Figure 136. ^{1}H NMR of **12**

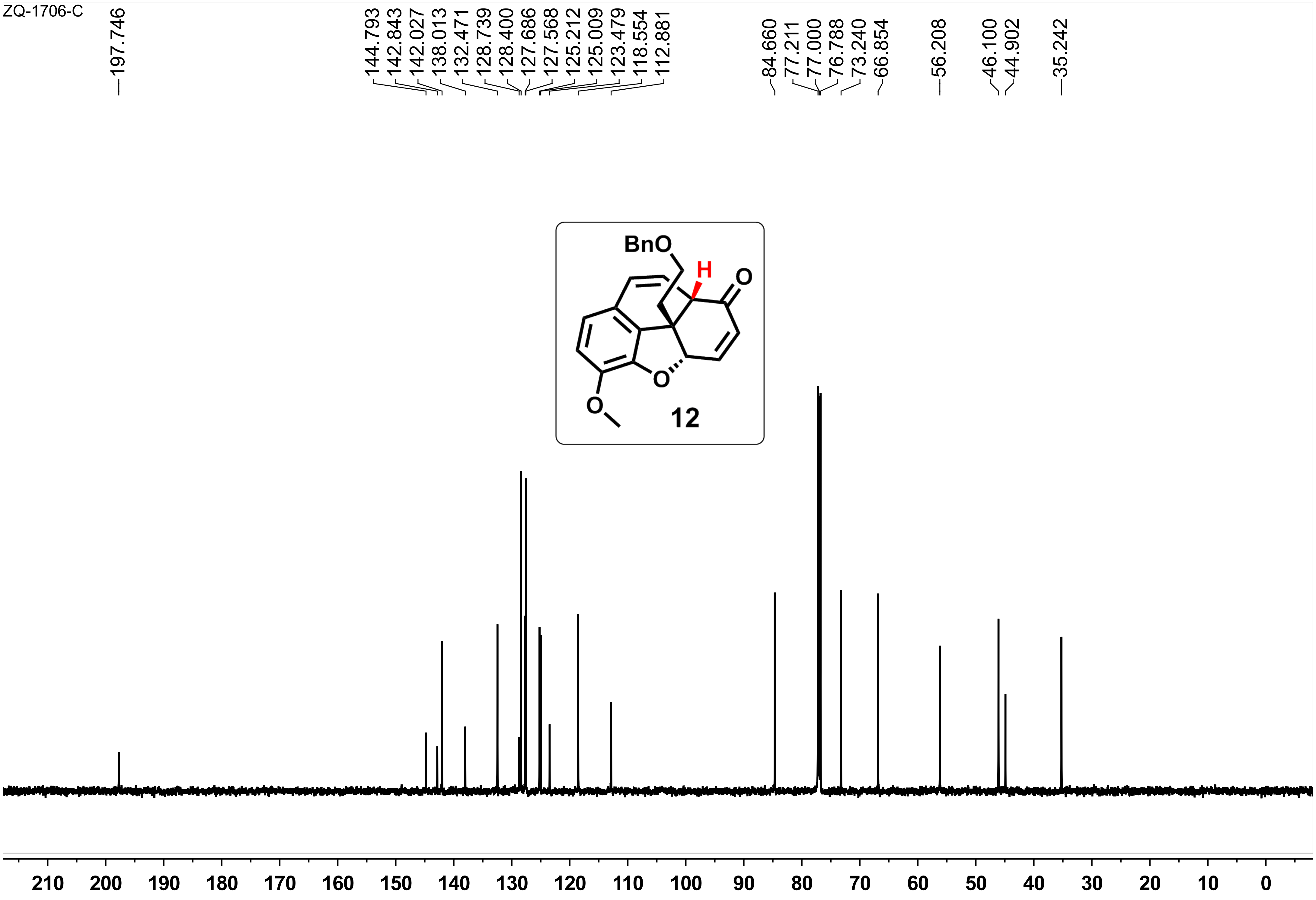

Supplementary Figure 137. ^{13}C NMR of **12**

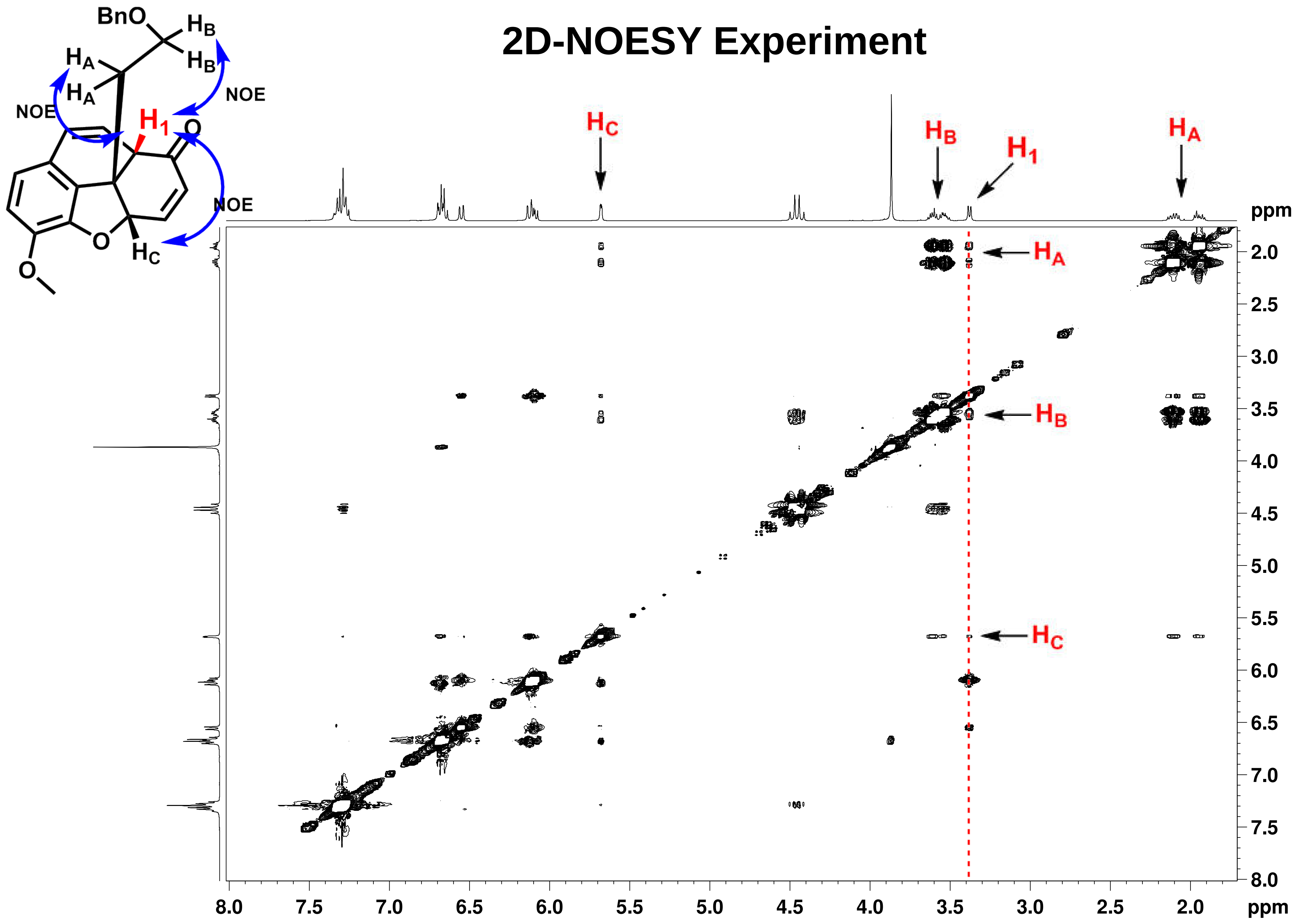

Supplementary Figure 138. 2D NOE of **12**

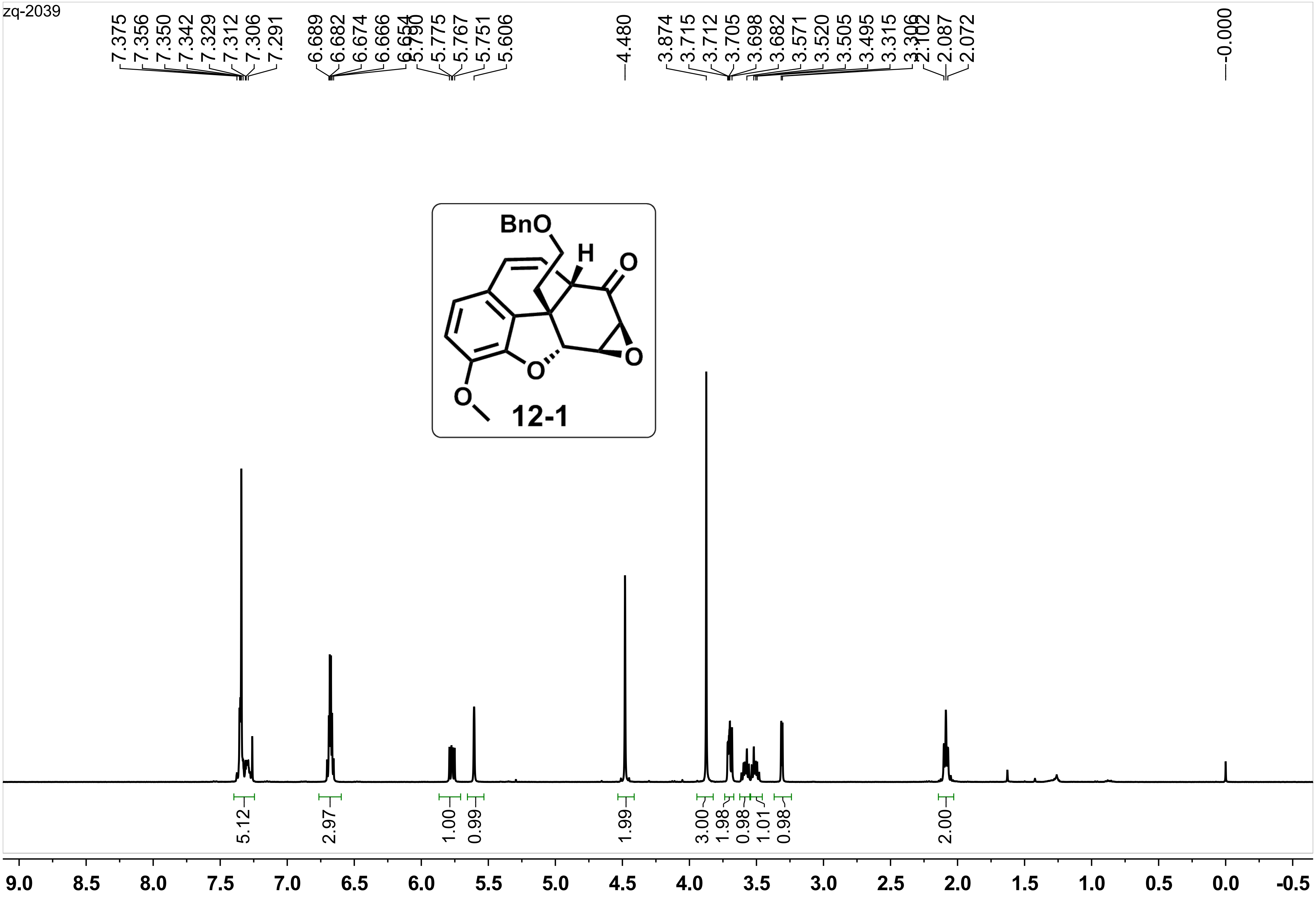

Supplementary Figure 139. ^{1}H NMR of **12-1**

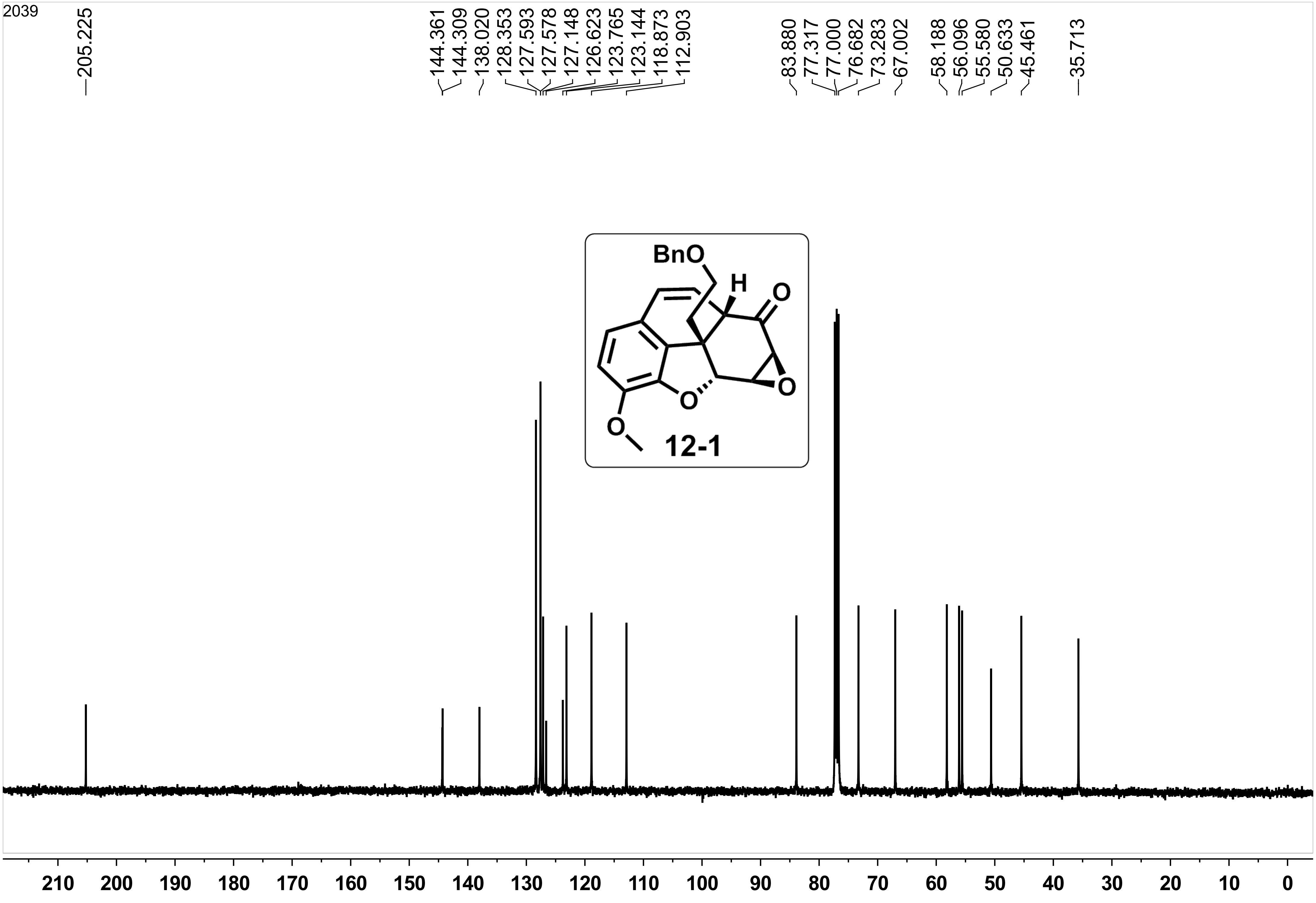

Supplementary Figure 140. ^{13}C NMR of **12-1**

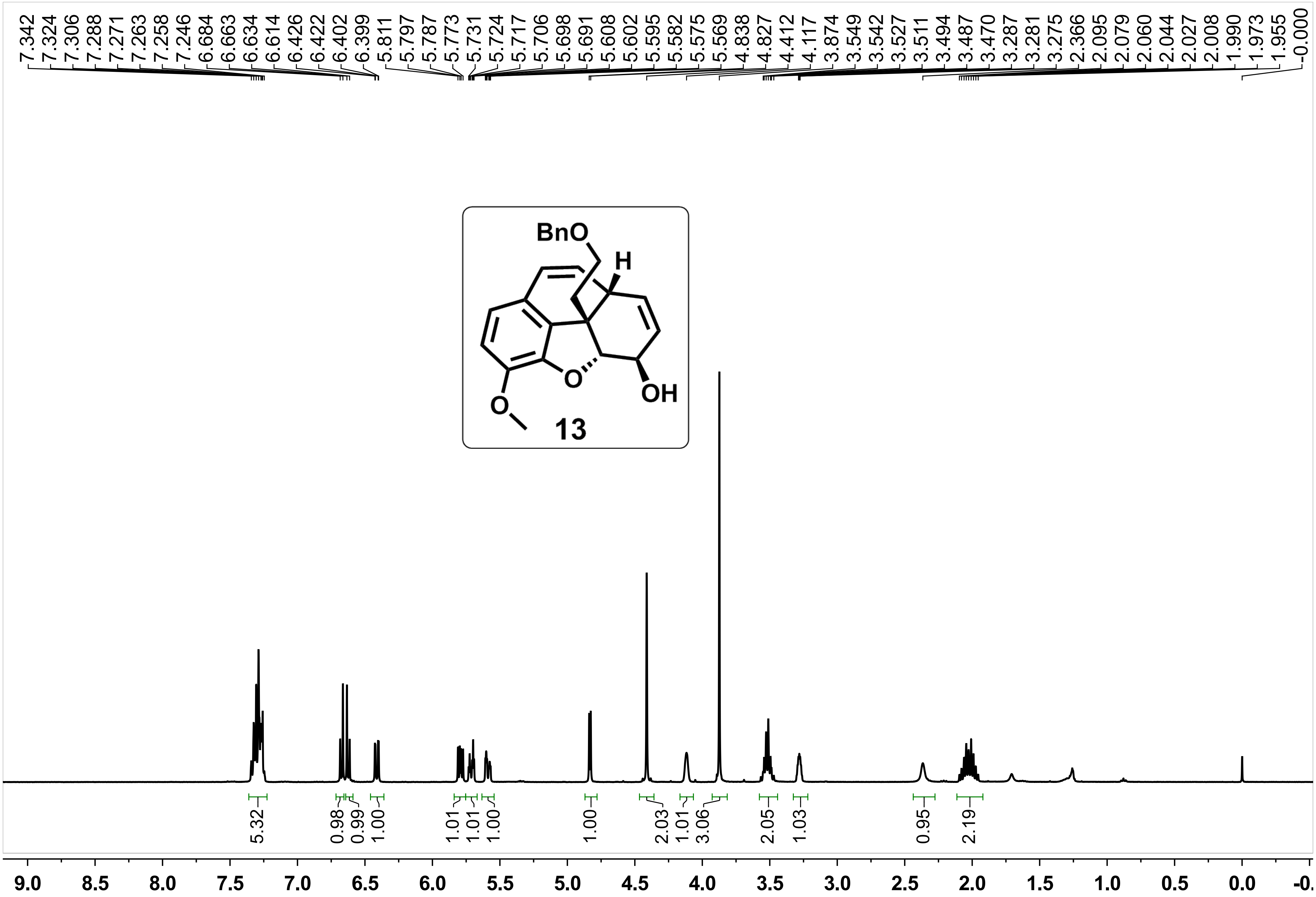

Supplementary Figure 141. ^{1}H NMR of **13**

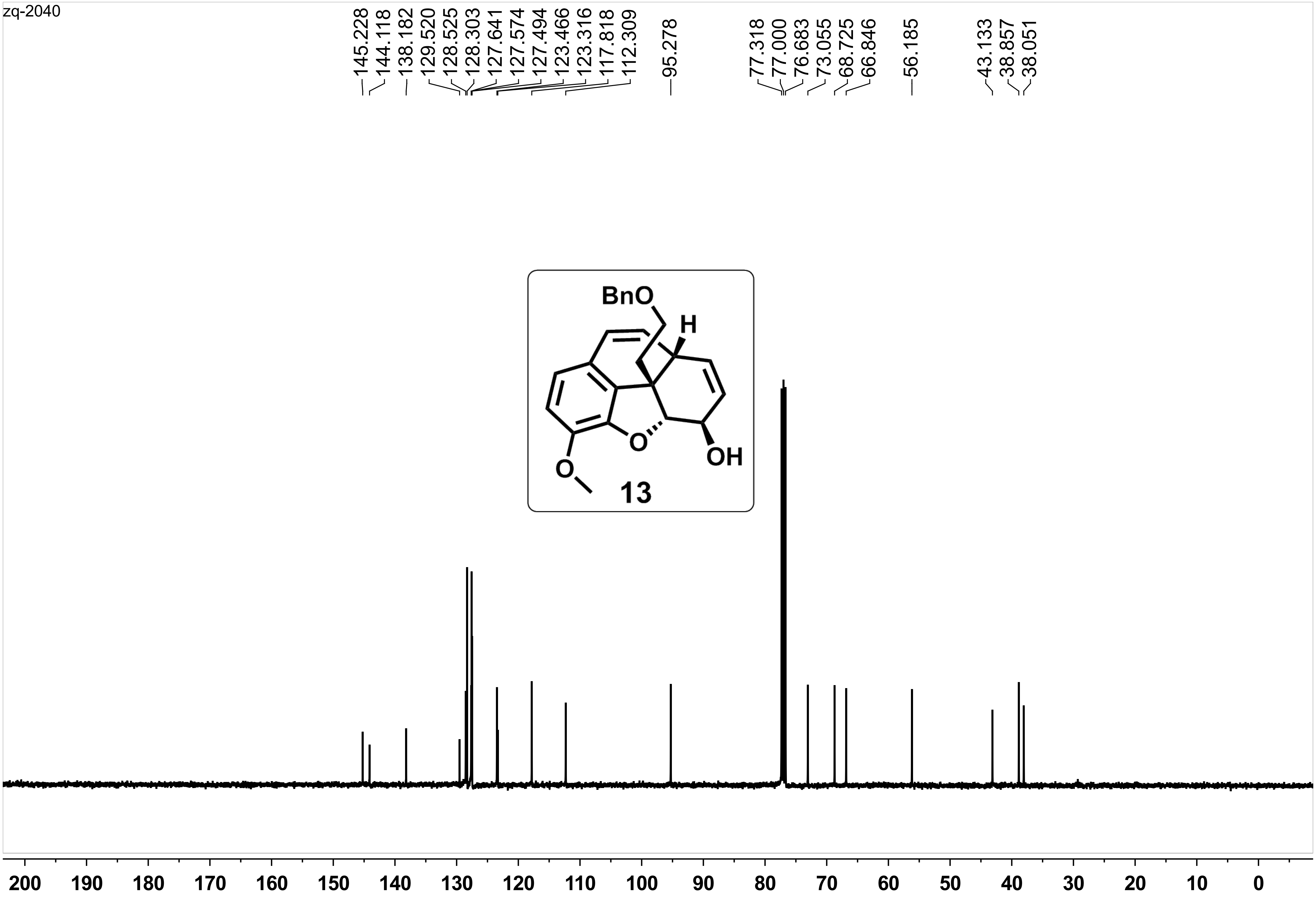

Supplementary Figure 142. ^{13}C NMR of **13**

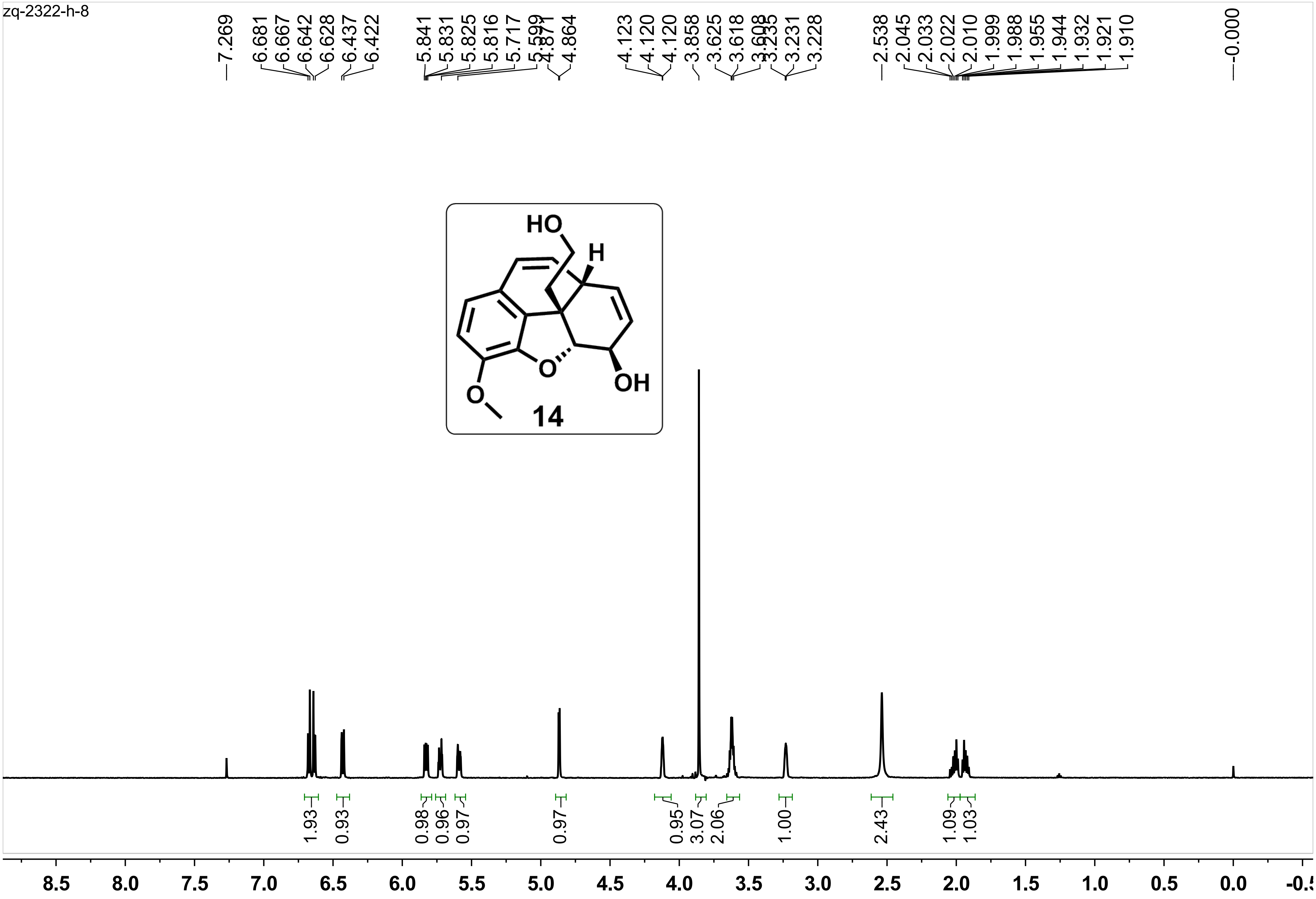

Supplementary Figure 143. ^{1}H NMR of **14**

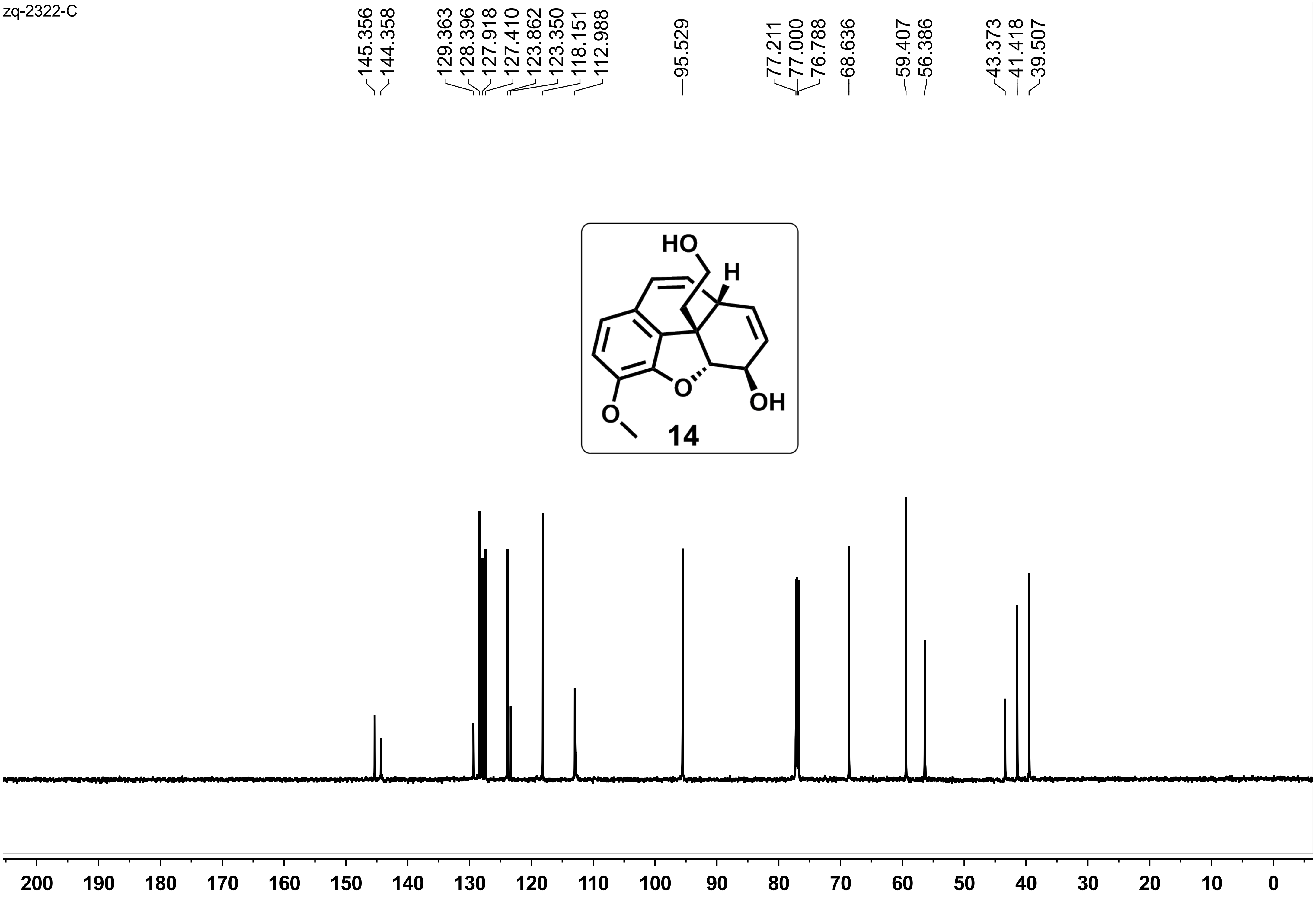

Supplementary Figure 144. ^{13}C NMR of **14**

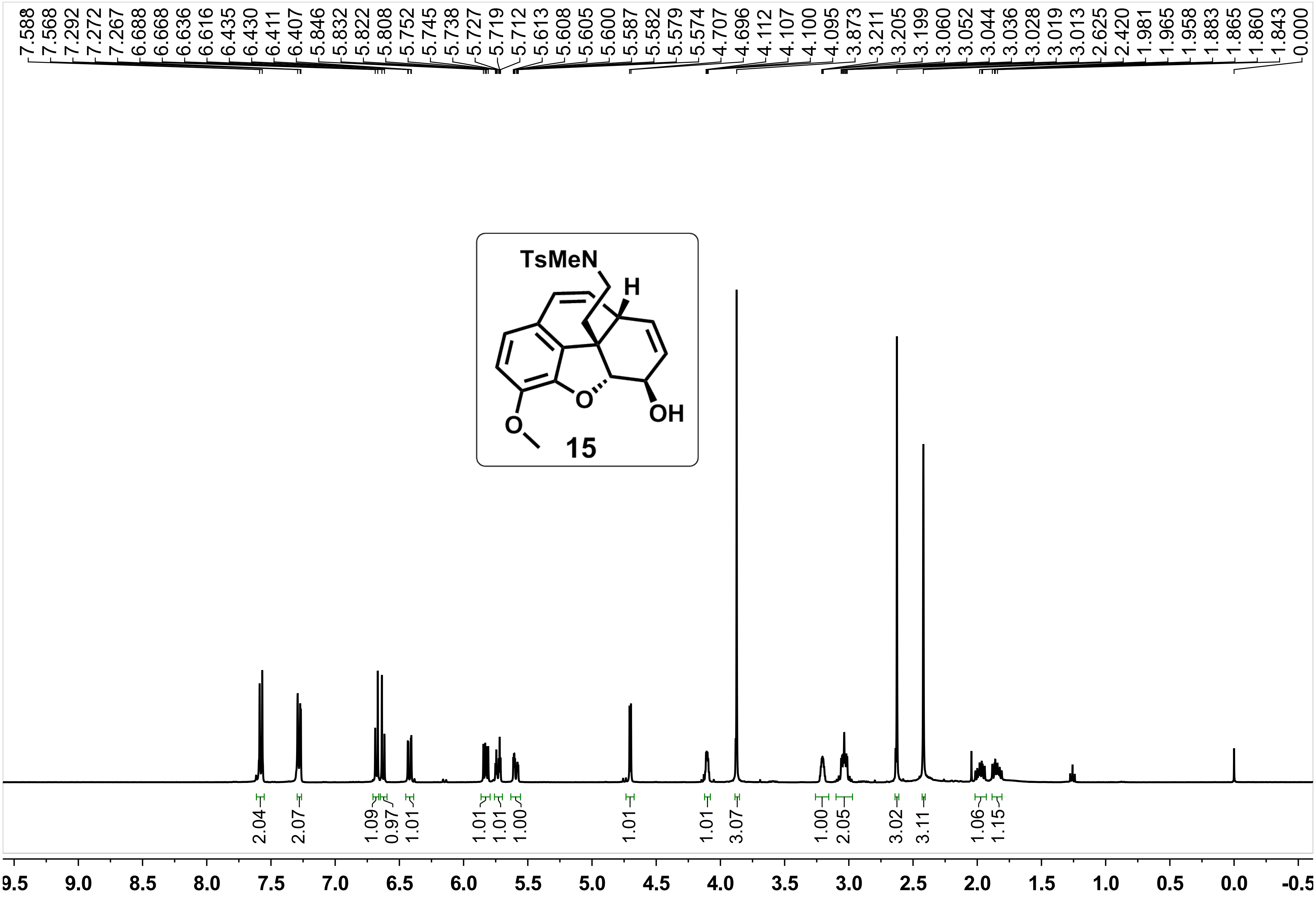

Supplementary Figure 145. ^{1}H NMR of **15**

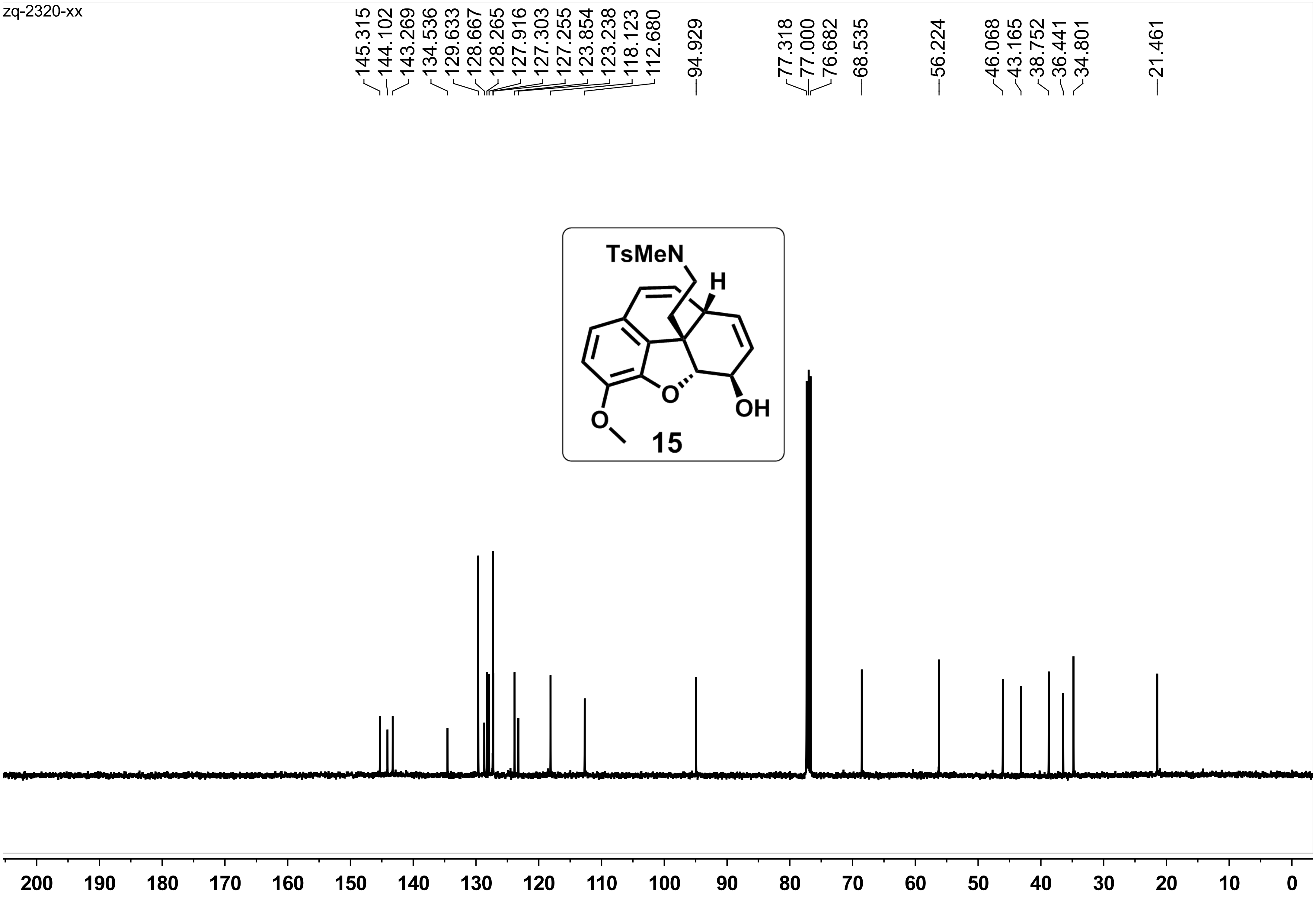

Supplementary Figure 146. ^{13}C NMR of **15**

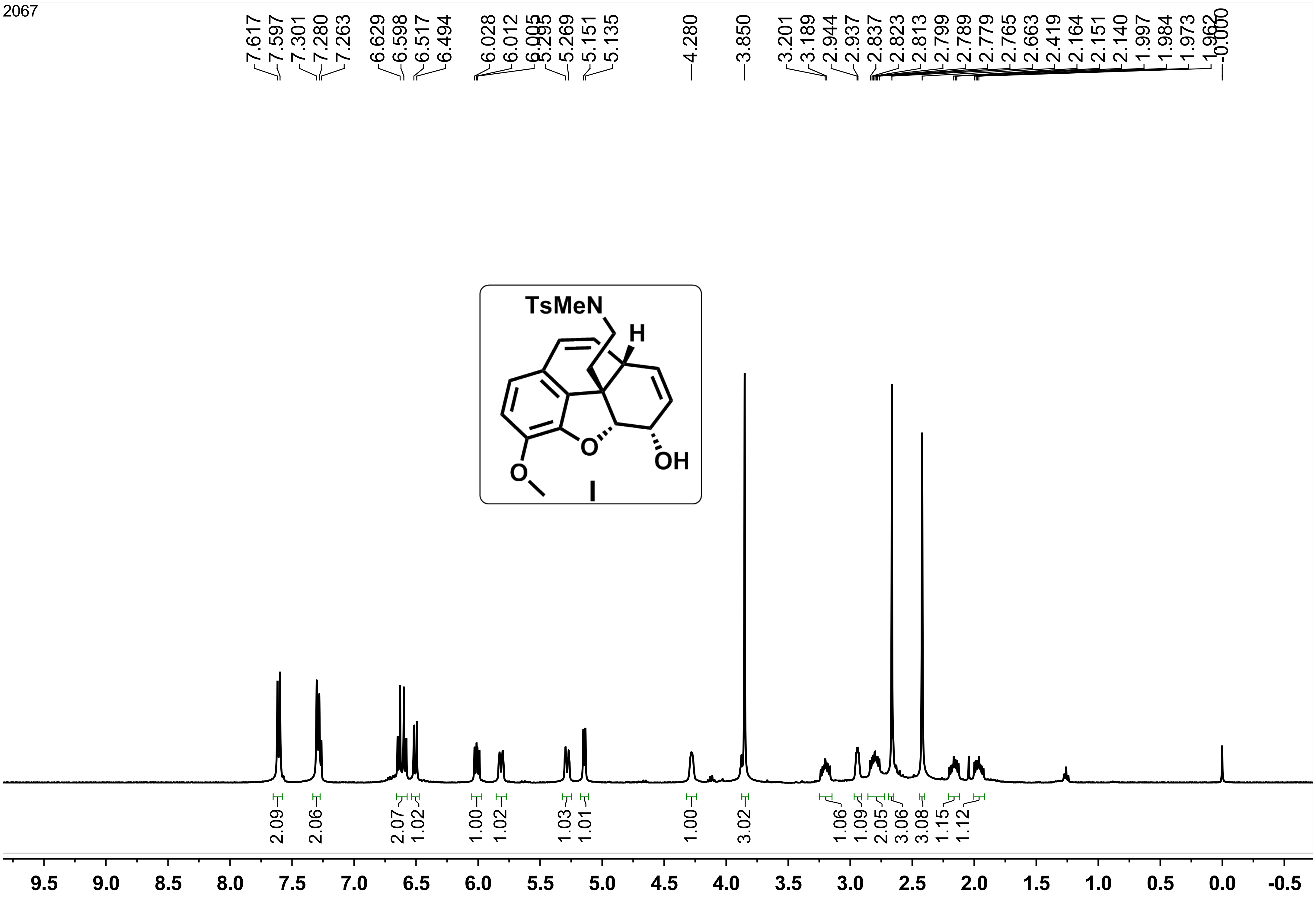

Supplementary Figure 147. ^{1}H NMR of **I**

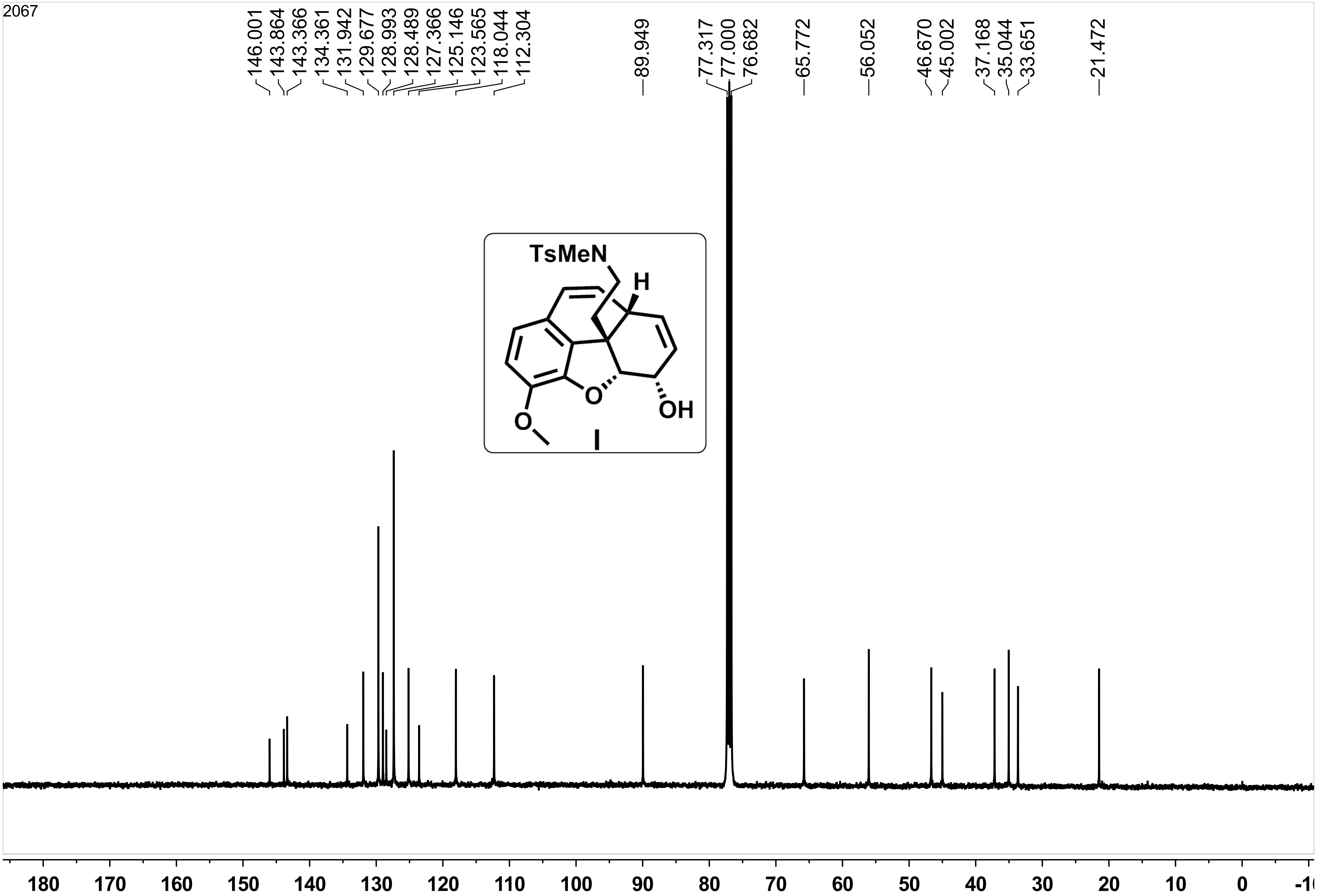

Supplementary Figure 148. ^{13}C NMR of **I**

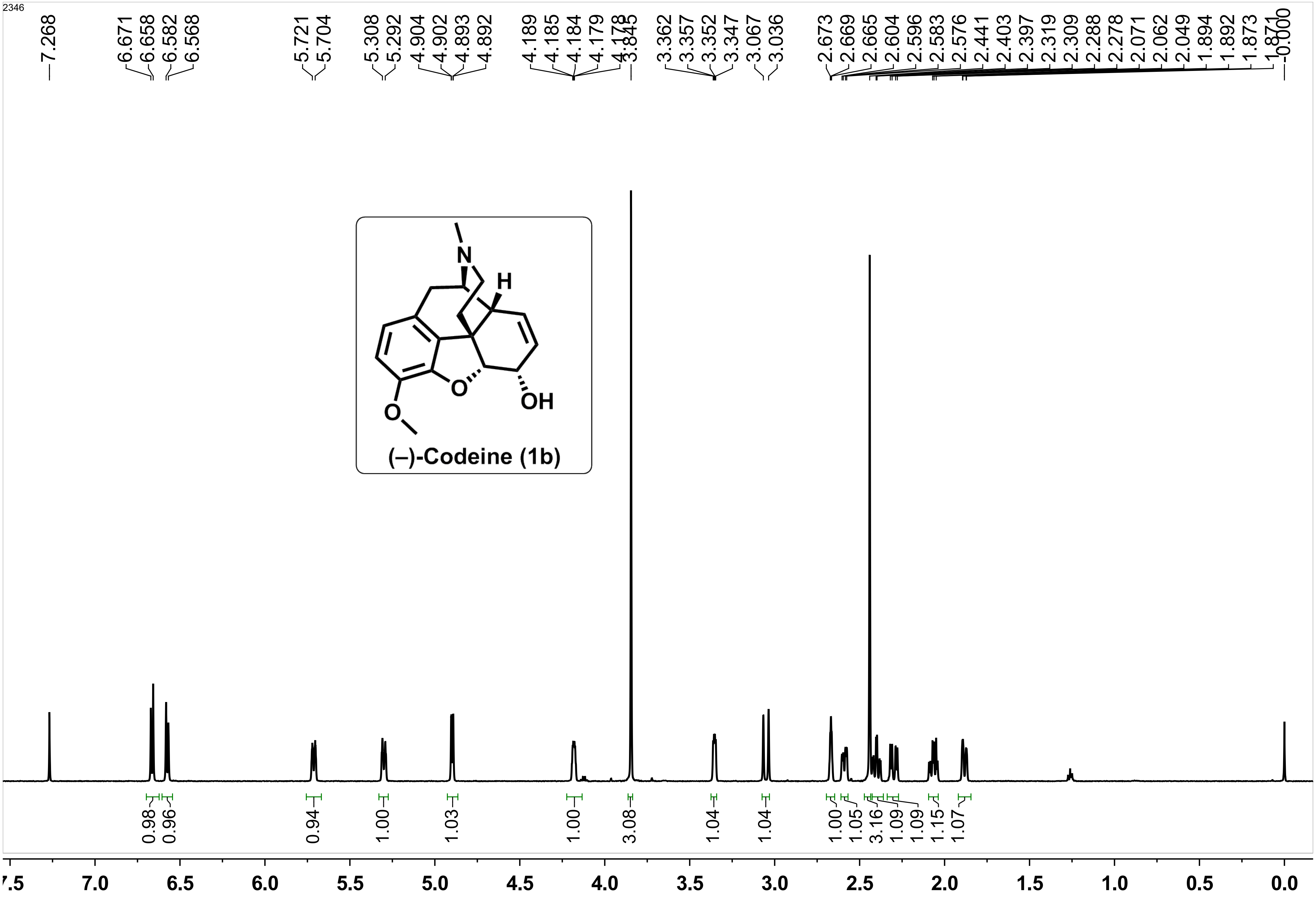

Supplementary Figure 149. ^{1}H NMR of **1b**

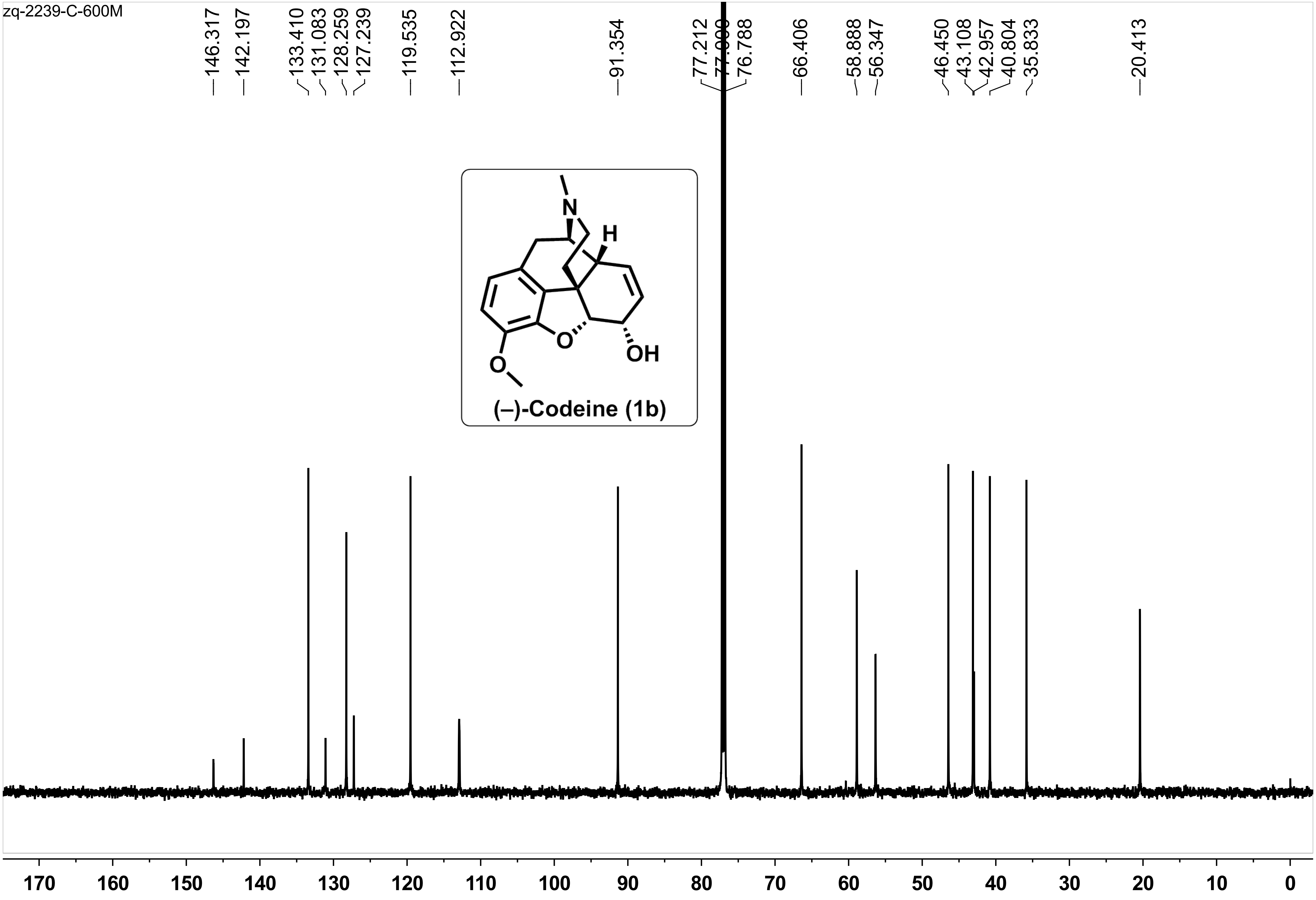

Supplementary Figure 150. ^{13}C NMR of **1b**

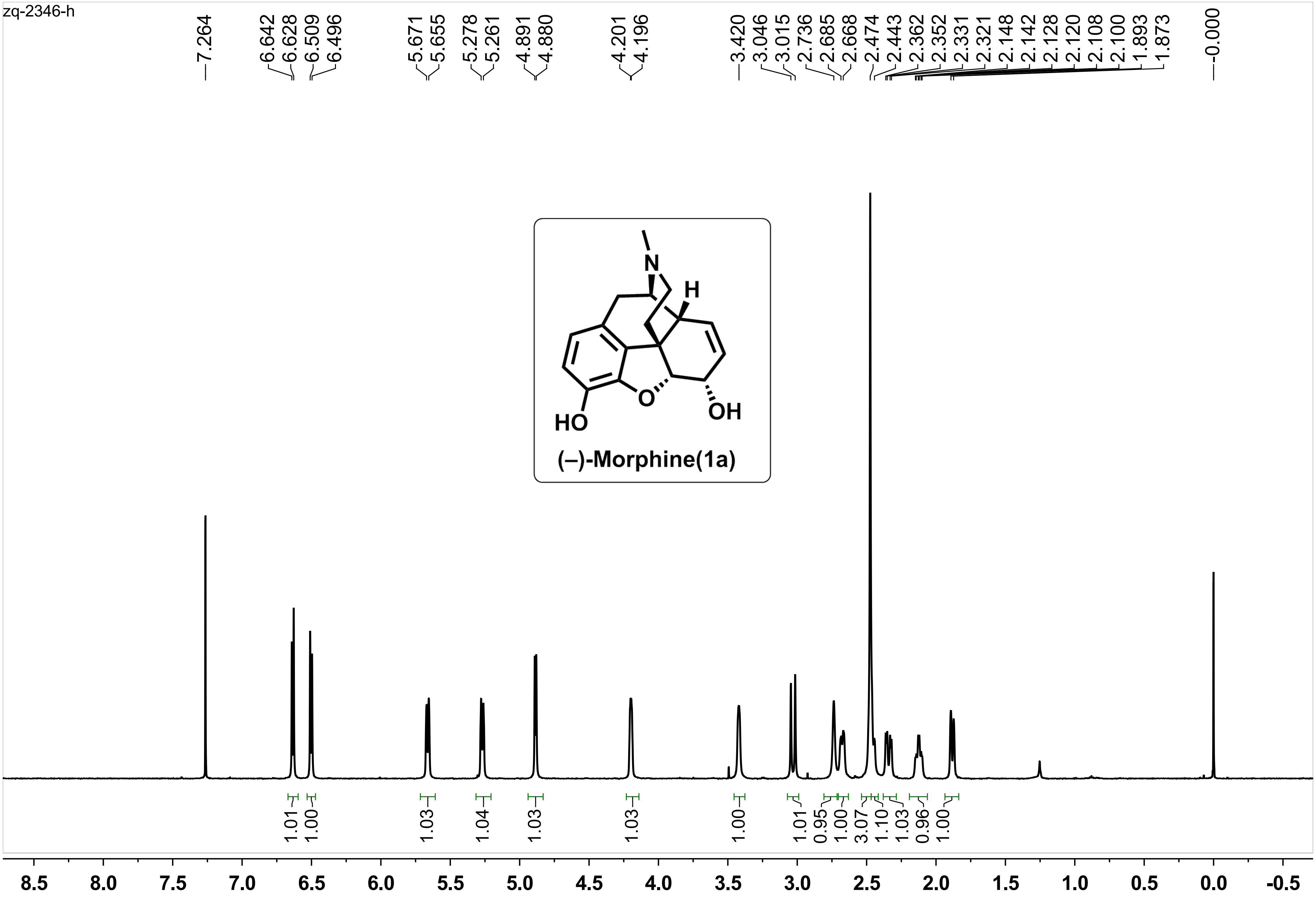

Supplementary Figure 151. ^{1}H NMR of **1a**

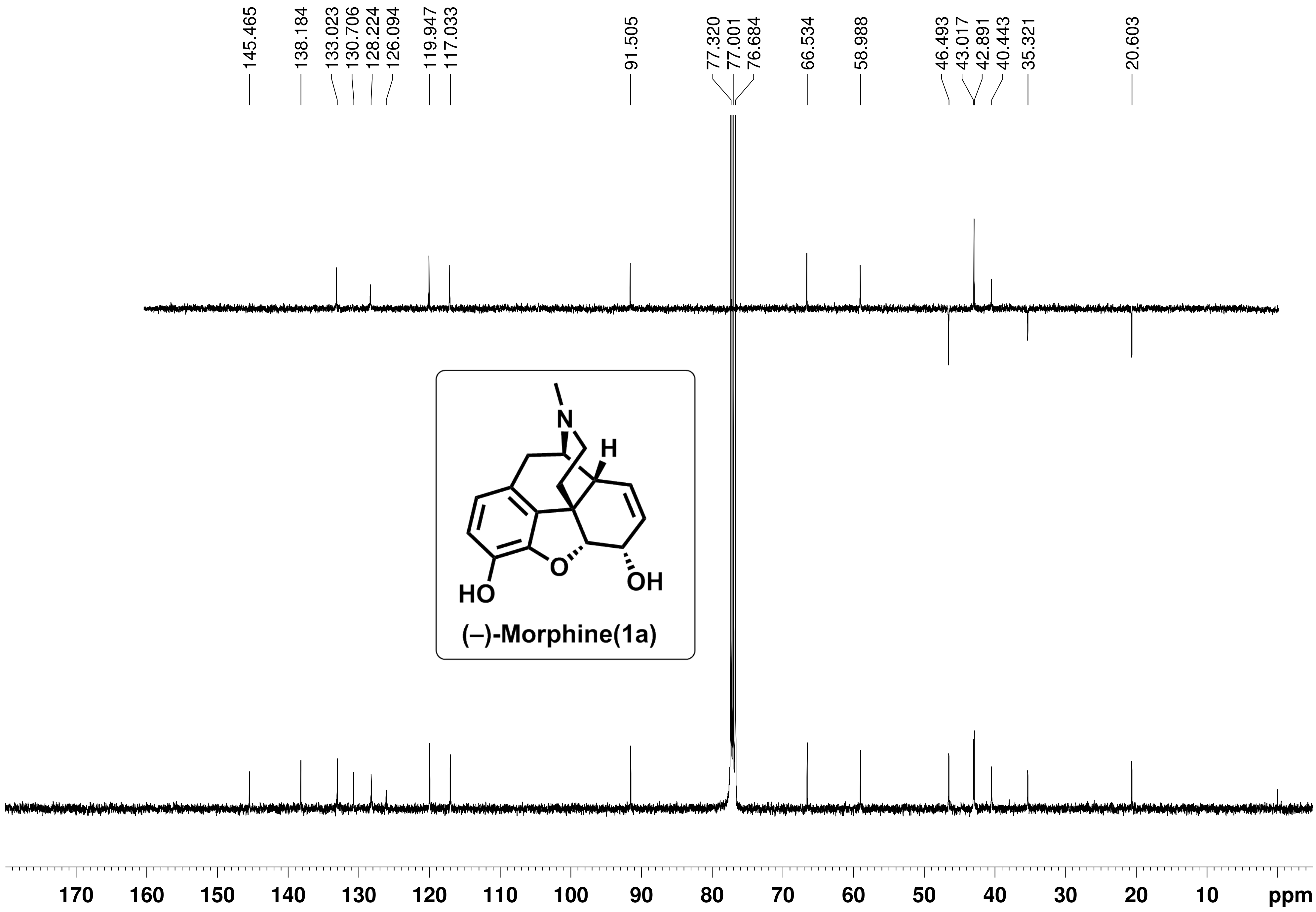

Supplementary Figure 152. ^{13}C NMR of **1a**

Sample Name: 2180-5-xx-80:20-OZ-H Wave Length: 2018/7/25 17:15:02 CST
Column: 10.00 ul

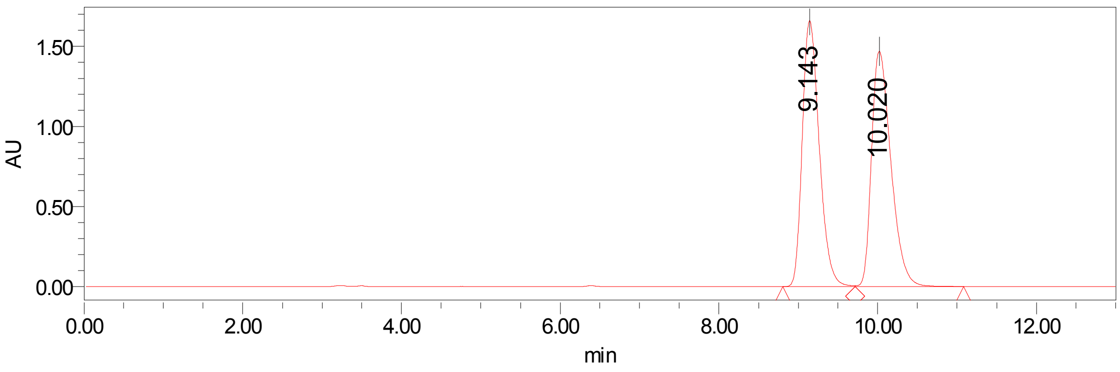

Peak Information:

	RetTime (min)	Area (uV*s)	Area (%)	Height (uV)
1	9.143	24591690.562	49.787	1658940
2	10.020	24801963.547	50.213	1466889

Sample Name: 2180-5-C-huan-OZ-H-80:20 Wave Length: 2018/6/23 15:23:56 CST
Column: 5.00 ul

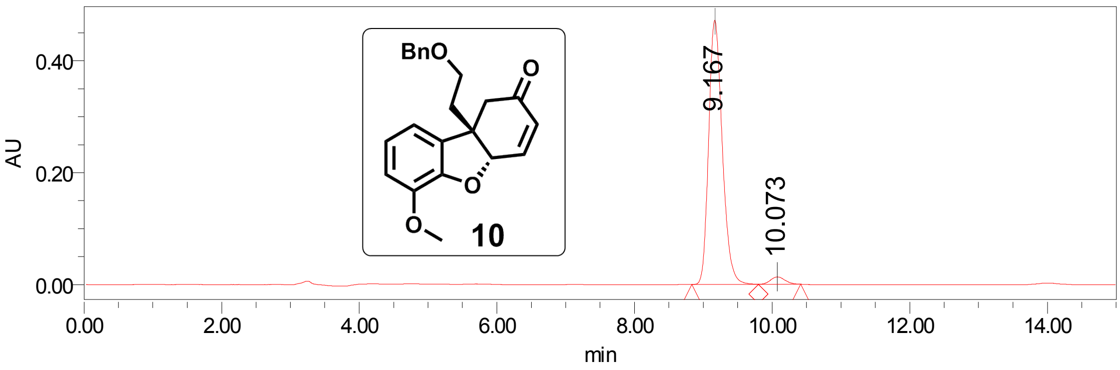

Peak Information:

	RetTime (min)	Area (uV*s)	Area (%)	Height (uV)
1	9.167	6486202.435	97.025	472590
2	10.073	198904.049	2.975	13429

Supplementary Figure 153. HPLC spectrum of **10**

Sample Name: 2180-5-xx-80:20-OZ-H Wave Length: 2018/7/25 17:15:02 CST
Column: 10.00 ul

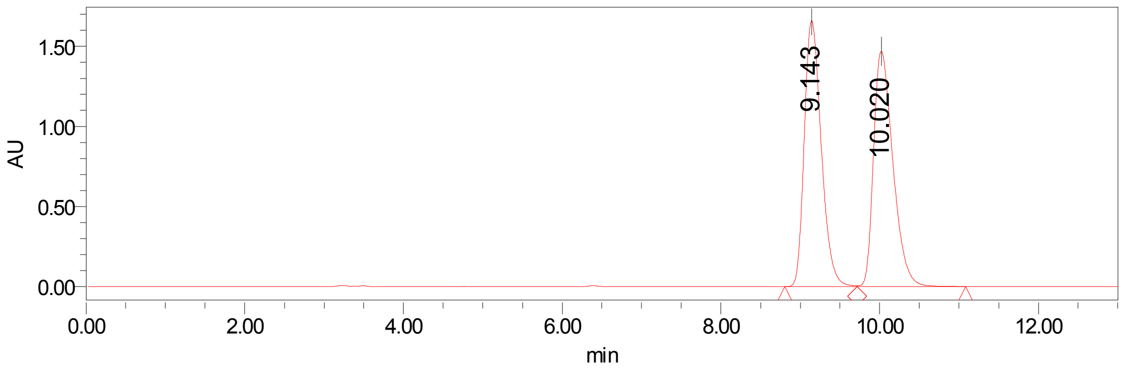

Peak Information:

	RetTime (min)	Area (uV*s)	Area (%)	Height (uV)
1	9.143	24591690.562	49.787	1658940
2	10.020	24801963.547	50.213	1466889

Sample Name: 2180-5-c-80:20-OZ-H-danjing Wave Length: 2018/7/25 17:01:20 CST
Column: 10.00 ul

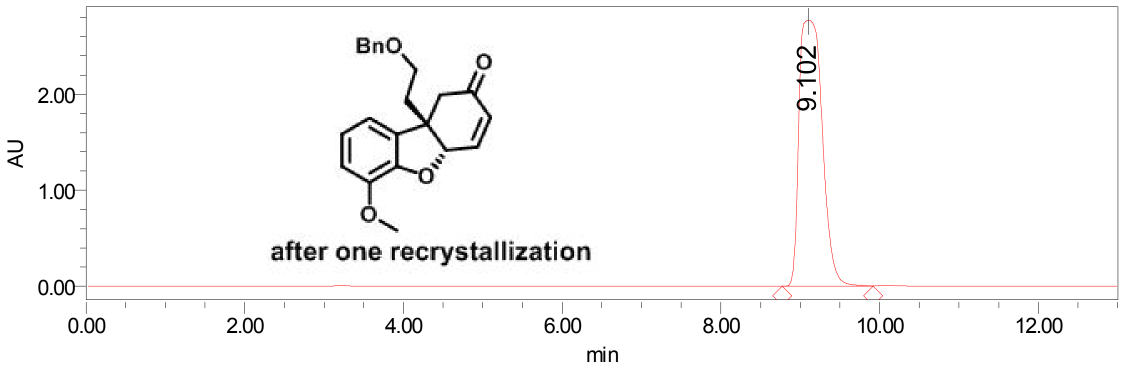

Peak Information:

	RetTime (min)	Area (uV*s)	Area (%)	Height (uV)
1	9.102	56308110.811	100.000	2771011

Supplementary Figure 154. HPLC spectrum of **10**

Sample Name: 2320-xx-IE-3-65:35 Wave Length: 2018/10/5 15:01:09 CST
Column: 10.00 ul

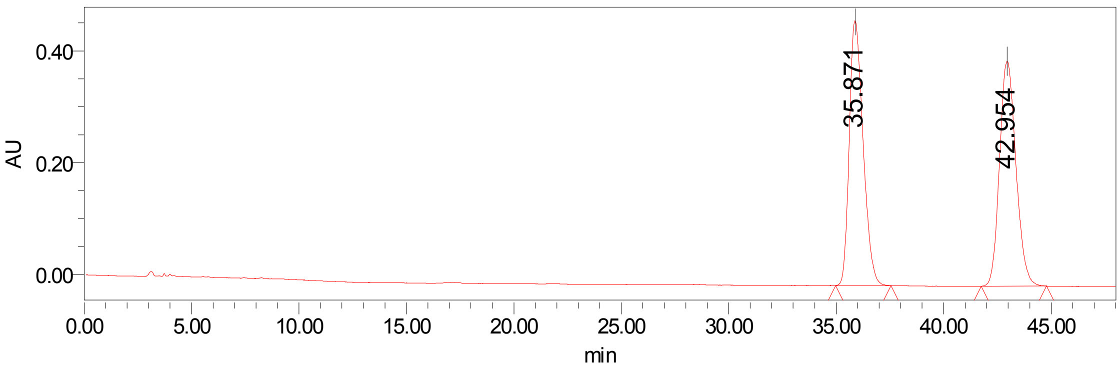

Peak Information:

	RetTime (min)	Area (uV*s)	Area (%)	Height (uV)
1	35.871	21230473.543	49.836	473976
2	42.954	21370133.096	50.164	402158

Sample Name: 2324-C-IE-3-65:35 Wave Length: 2018/10/5 12:51:50 CST
Column: 10.00 ul

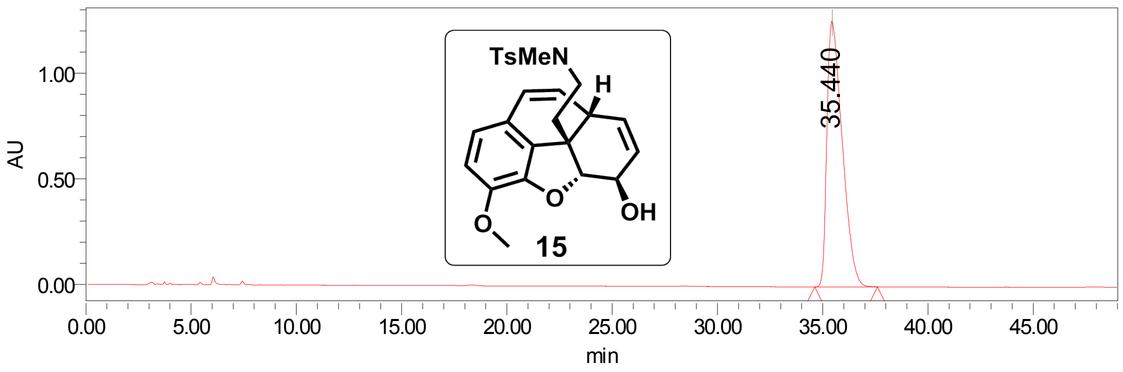

Peak Information:

	RetTime (min)	Area (uV*s)	Area (%)	Height (uV)
1	35.440	63520123.675	100.000	1256627

Supplementary Figure 155. HPLC spectrum of **15**

Sample Name: xx-p
Column: 10.00 ul

Wave Length: 2018/4/9 16:31:05 CST

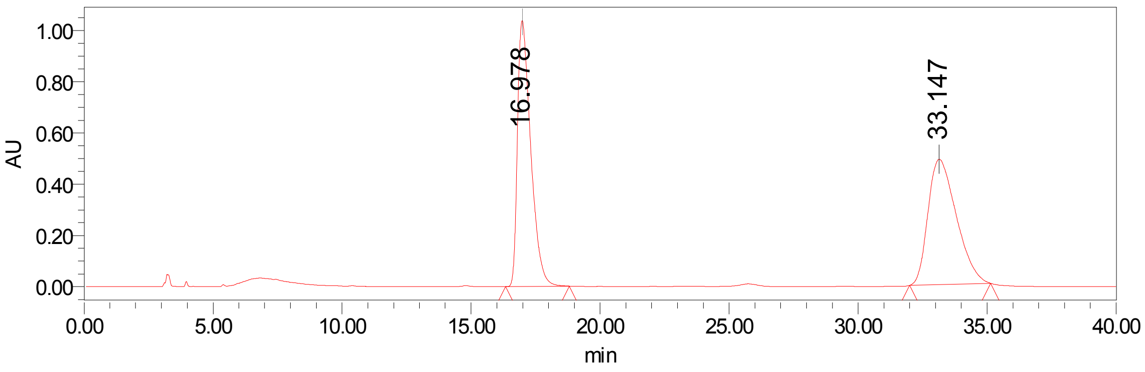

Peak Information:

	RetTime (min)	Area (uV*s)	Area (%)	Height (uV)
1	16.978	36971354.224	49.707	1038085
2	33.147	37407736.686	50.293	489620

Sample Name: 2114-5
Column: 10.00 ul

Wave Length: 2018/4/9 13:03:41 CST

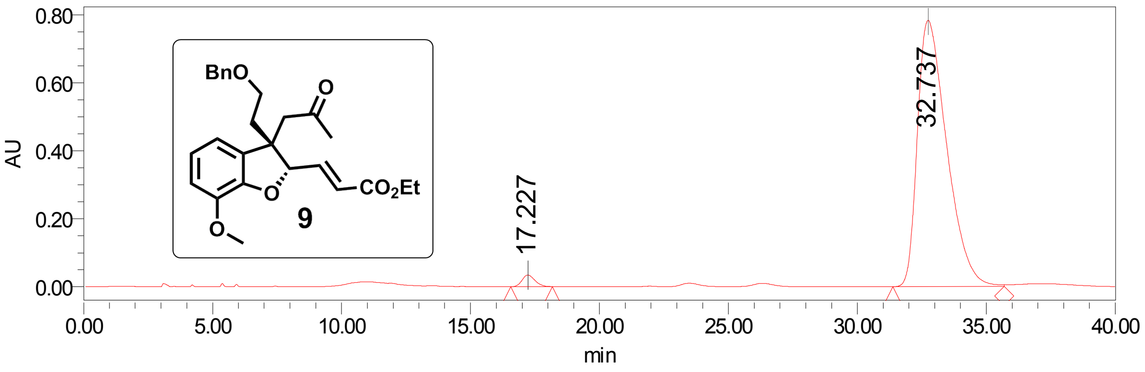

Peak Information:

	RetTime (min)	Area (uV*s)	Area (%)	Height (uV)
1	17.227	1197821.934	1.895	34294
2	32.737	62011287.214	98.105	783307

Supplementary Figure 156. HPLC spectrum of **9**

Sample Name: 2198-xx-IF-3-95:5 Wave Length: 2018/6/18 11:15:04 CST
Column: 5.00 ul

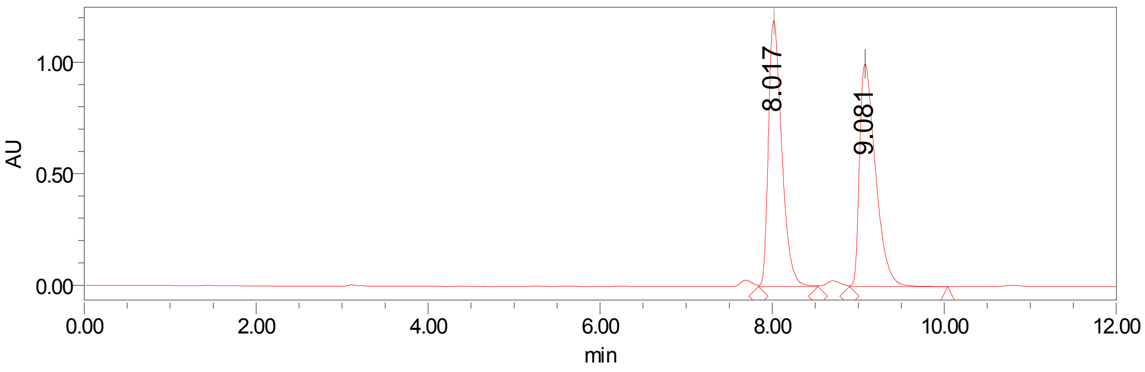

Peak Information:

	RetTime (min)	Area (uV*s)	Area (%)	Height (uV)
1	8.017	12826582.152	49.905	1193163
2	9.081	12875394.137	50.095	998695

Sample Name: 2198-c-IF-3-95:5 Wave Length: 2018/6/18 11:02:25 CST
Column: 5.00 ul

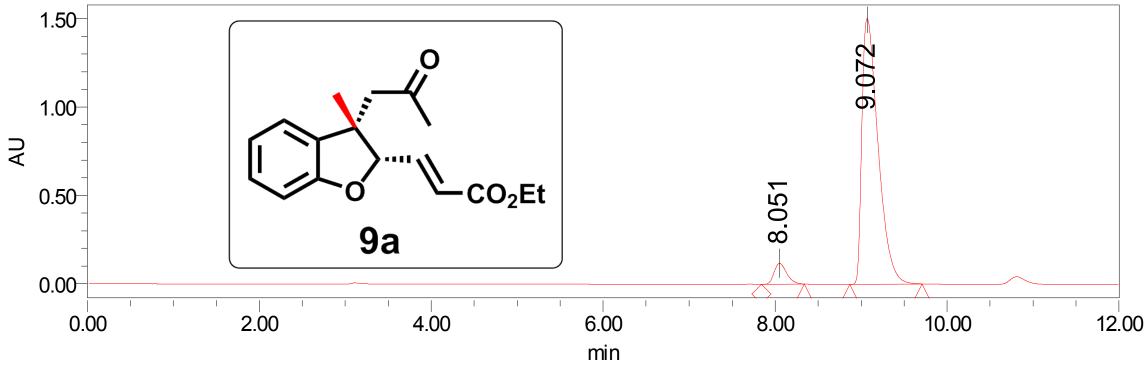

Peak Information:

	RetTime (min)	Area (uV*s)	Area (%)	Height (uV)
1	8.051	1191366.162	5.727	117902
2	9.072	19609546.720	94.273	1504634

Supplementary Figure 157. HPLC spectrum of **9a**

Sample Name: 2199-XX-IF-3-95:5 Wave Length: 2018/6/18 17:54:43 CST
Column: 5.00 ul

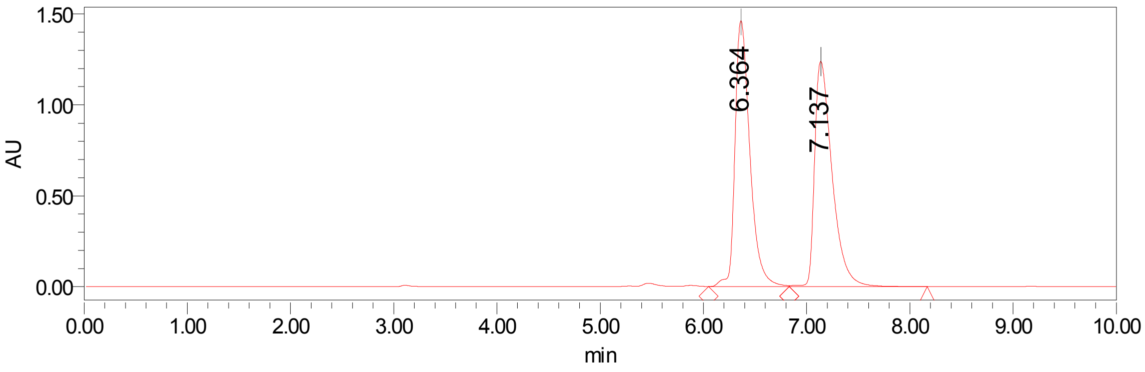

Peak Information:

	RetTime (min)	Area (uV*s)	Area (%)	Height (uV)
1	6.364	14687931.337	50.234	1463351
2	7.137	14551169.823	49.766	1239048

Sample Name: 2199-c-IF-3-95:5 Wave Length: 2018/6/18 17:44:05 CST
Column: 5.00 ul

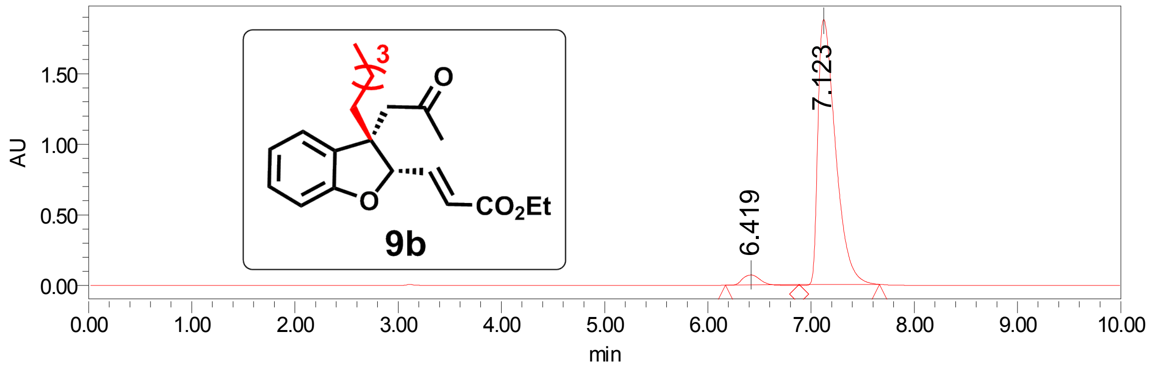

Peak Information:

	RetTime (min)	Area (uV*s)	Area (%)	Height (uV)
1	6.419	856925.001	3.727	69731
2	7.123	22134134.820	96.273	1877212

Supplementary Figure 158. HPLC spectrum of **9b**

Sample Name: 2170-xx-IF-3-98:2
Column: 3.00 ul
Wave Length: 2018/5/28 20:57:27 CST

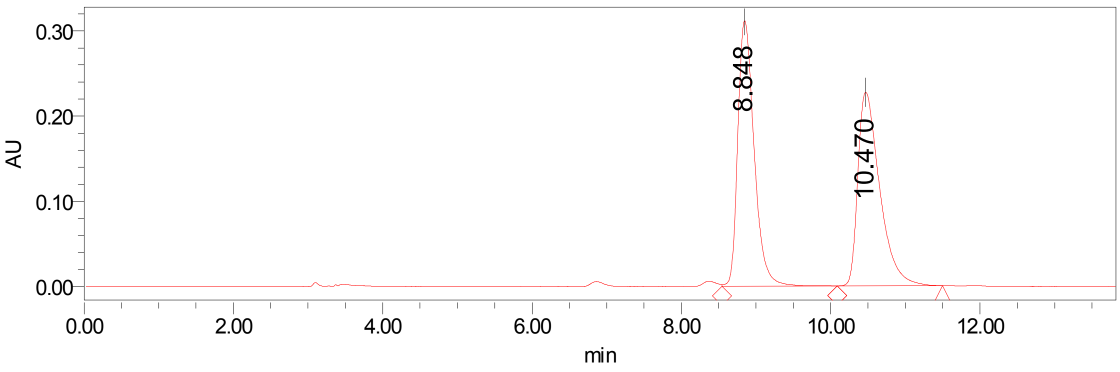

Peak Information:

	RetTime (min)	Area (uV*s)	Area (%)	Height (uV)
1	8.848	4723465.009	50.536	311624
2	10.470	4623239.340	49.464	227138

Sample Name: 2173-c-IF-3-95:5
Column: 5.00 ul
Wave Length: 2018/6/2 10:43:40 CST

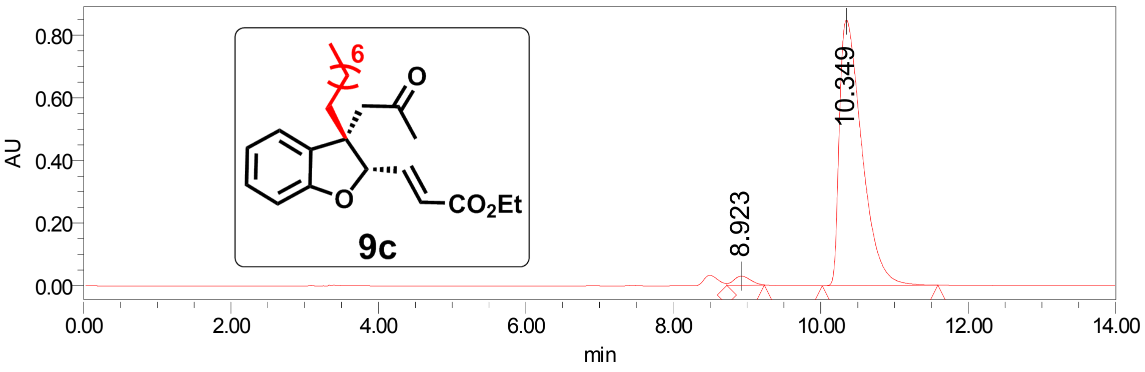

Peak Information:

	RetTime (min)	Area (uV*s)	Area (%)	Height (uV)
1	8.923	456586.073	2.476	28970
2	10.349	17985364.486	97.524	847845

Supplementary Figure 159. HPLC spectrum of **9c**

Sample Name: 2162-xx-IE-3-95:5
Column: 5.00 ul
Wave Length: 2018/5/24 8:54:44 CST

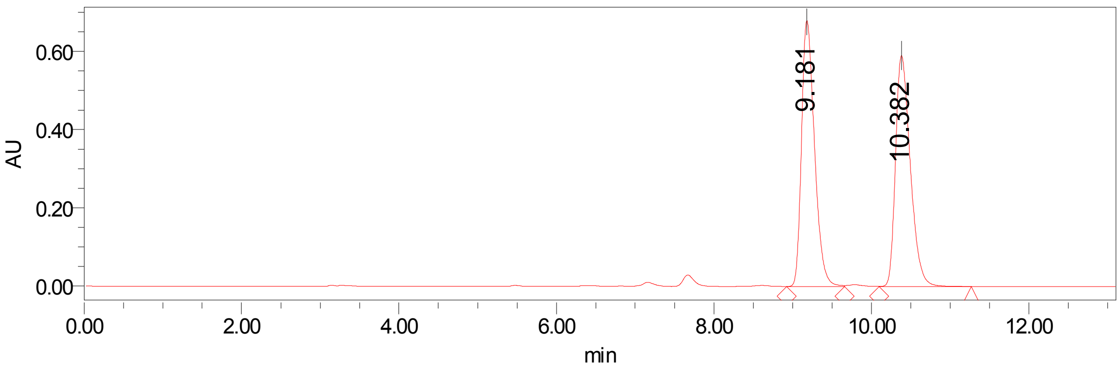

Peak Information:

	RetTime (min)	Area (uV*s)	Area (%)	Height (uV)
1	9.181	7771434.901	50.259	679950
2	10.382	7691256.623	49.741	590788

Sample Name: 2162-c-IE-3-95:5
Column: 10.00 ul
Wave Length: 2018/5/25 9:34:32 CST

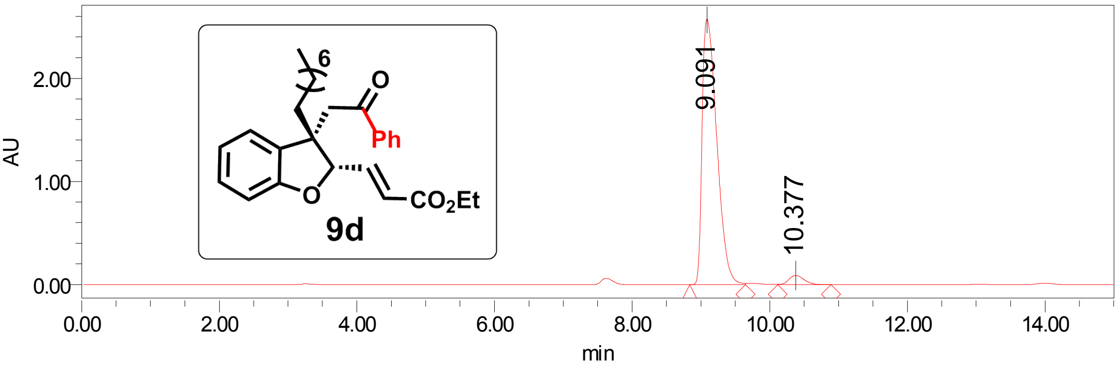

Peak Information:

	RetTime (min)	Area (uV*s)	Area (%)	Height (uV)
1	9.091	38178691.503	96.452	2579017
2	10.377	1404591.512	3.548	89646

Supplementary Figure 160. HPLC spectrum of **9d**

Sample Name: 2192-xx-ID-3-95:5 Wave Length: 2018/6/18 19:47:36 CST
Column: 5.00 ul

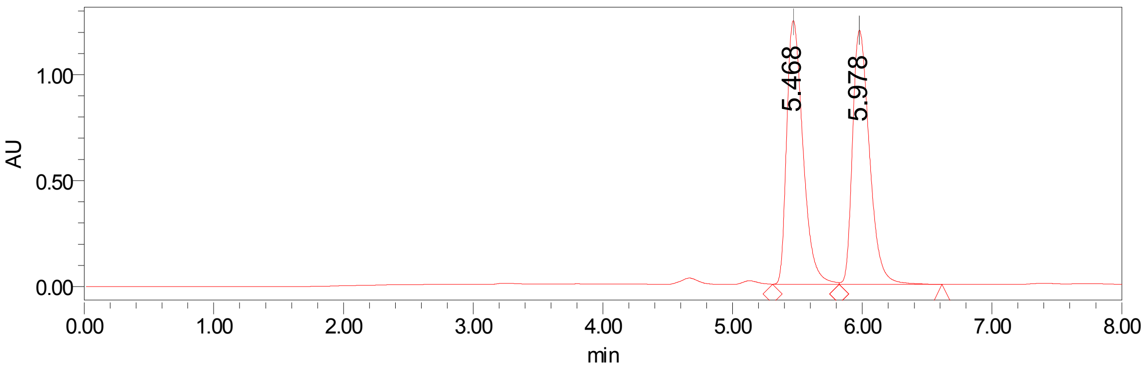

Peak Information:

	RetTime (min)	Area (uV*s)	Area (%)	Height (uV)
1	5.468	10834751.665	49.903	1244395
2	5.978	10876872.762	50.097	1198824

Sample Name: 2194-c-xiao-ID-3-95:5 Wave Length: 2018/6/18 20:08:40 CST
Column: 5.00 ul

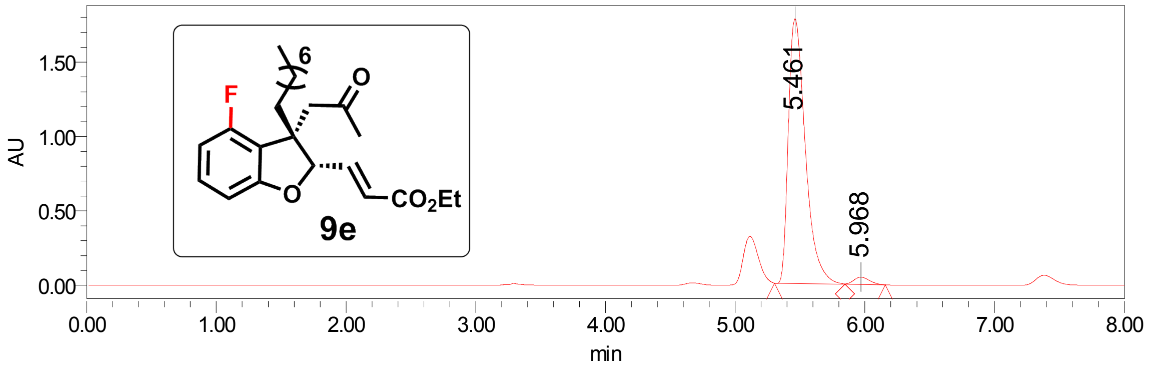

Peak Information:

	RetTime (min)	Area (uV*s)	Area (%)	Height (uV)
1	5.461	16201374.103	97.499	1777765
2	5.968	415595.755	2.501	49176

Supplementary Figure 161. HPLC spectrum of **9e**

Sample Name: 2168-xx-IF-3-95:5 Wave Length: 2018/5/28 18:03:07 CST
Column: 5.00 ul

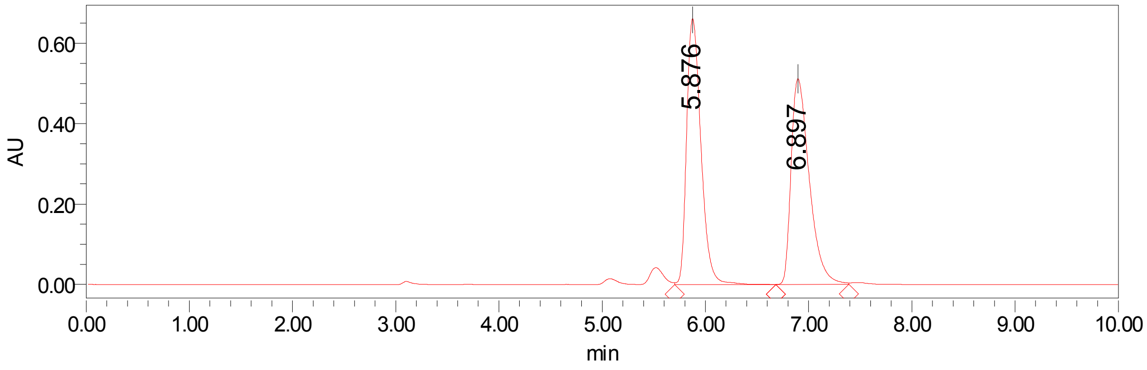

Peak Information:

	RetTime (min)	Area (uV*s)	Area (%)	Height (uV)
1	5.876	6374037.937	50.431	661610
2	6.897	6265009.821	49.569	511854

Sample Name: 2168-c-IF-3-95:5 Wave Length: 2018/5/28 17:52:27 CST
Column: 5.00 ul

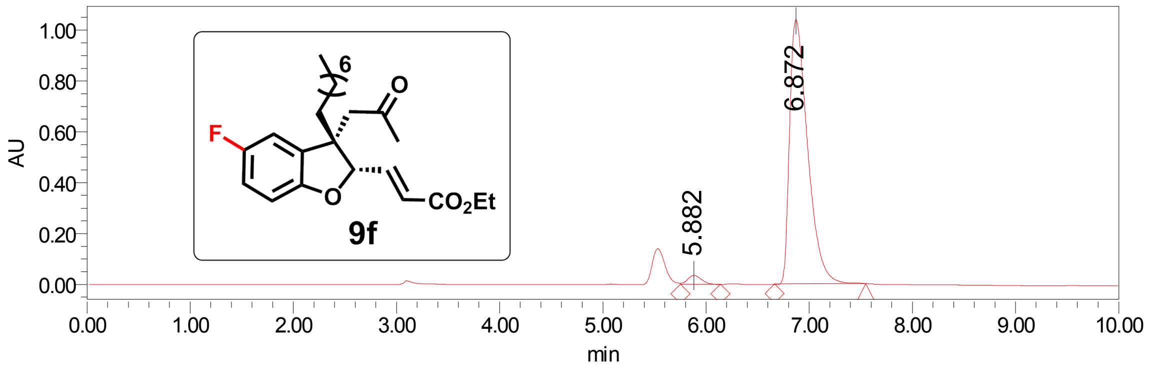

Peak Information:

	RetTime (min)	Area (uV*s)	Area (%)	Height (uV)
1	5.882	319327.227	2.472	34369
2	6.872	12597598.551	97.528	1038413

Supplementary Figure 162. HPLC spectrum of **9f**

Sample Name: 2169-xx-IF-3-98:2 Wave Length: 2018/5/28 20:22:43 CST
Column: 3.00 ul

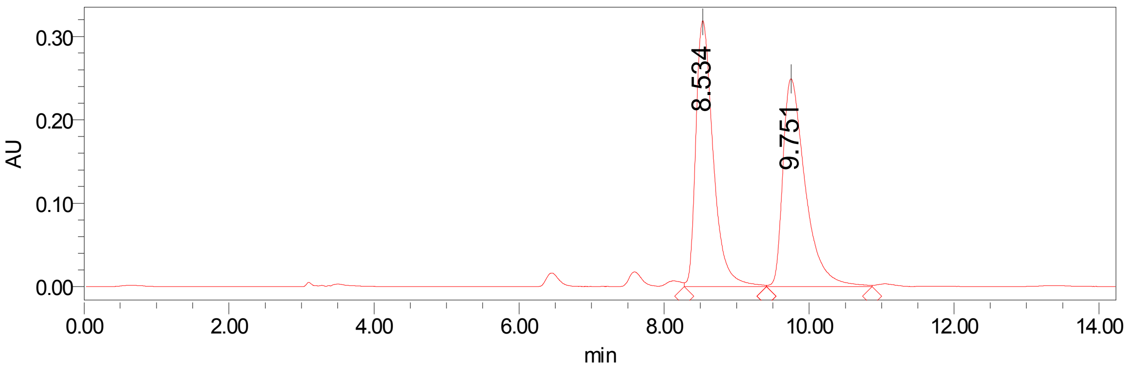

Peak Information:

	RetTime (min)	Area (uV*s)	Area (%)	Height (uV)
1	8.534	5302855.728	49.805	318725
2	9.751	5344314.934	50.195	249006

Sample Name: 2169-c-IF-3-98:2 Wave Length: 2018/5/28 20:07:29 CST
Column: 3.00 ul

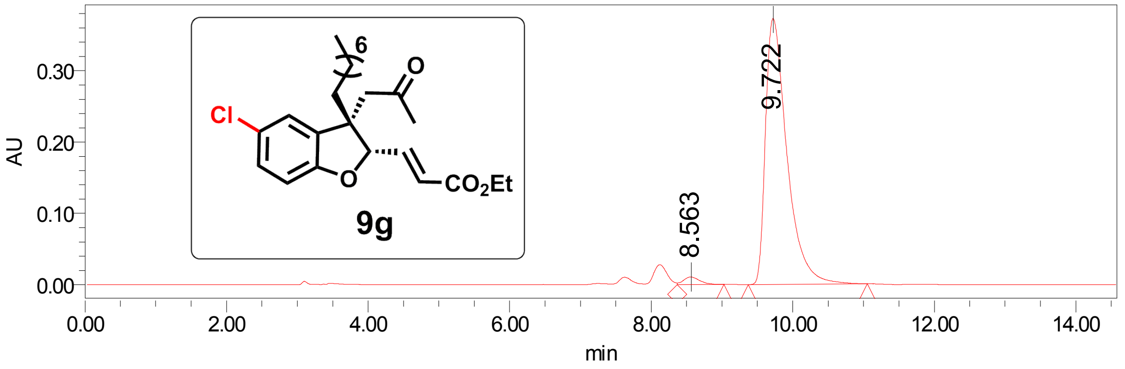

Peak Information:

	RetTime (min)	Area (uV*s)	Area (%)	Height (uV)
1	8.563	168408.648	2.098	10551
2	9.722	7860385.527	97.902	372871

Supplementary Figure 163. HPLC spectrum of **9g**

Sample Name: 2159-xx-IE-3-95-5 Wave Length: 2018/5/22 15:01:19 CST
Column: 5.00 ul

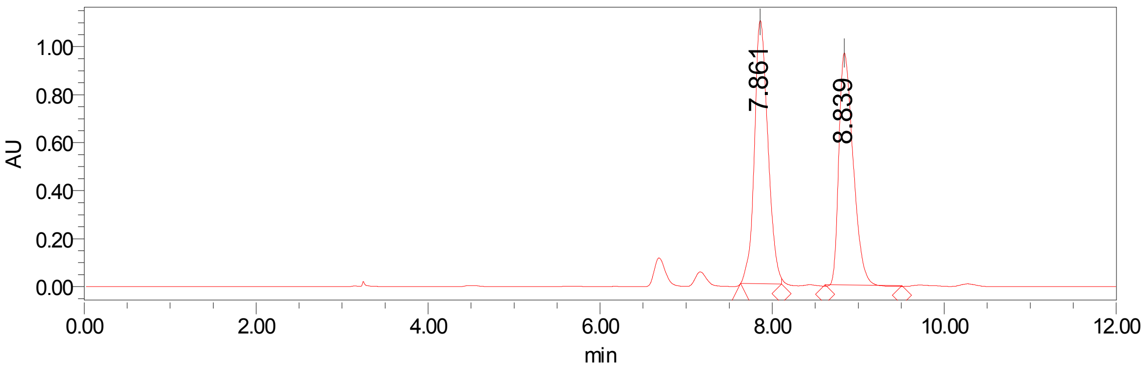

Peak Information:

	RetTime (min)	Area (uV*s)	Area (%)	Height (uV)
1	7.861	11906774.820	51.667	1096002
2	8.839	11138508.753	48.333	966121

Sample Name: 2159-c-IE-3-95-5 Wave Length: 2018/5/22 15:14:10 CST
Column: 5.00 ul

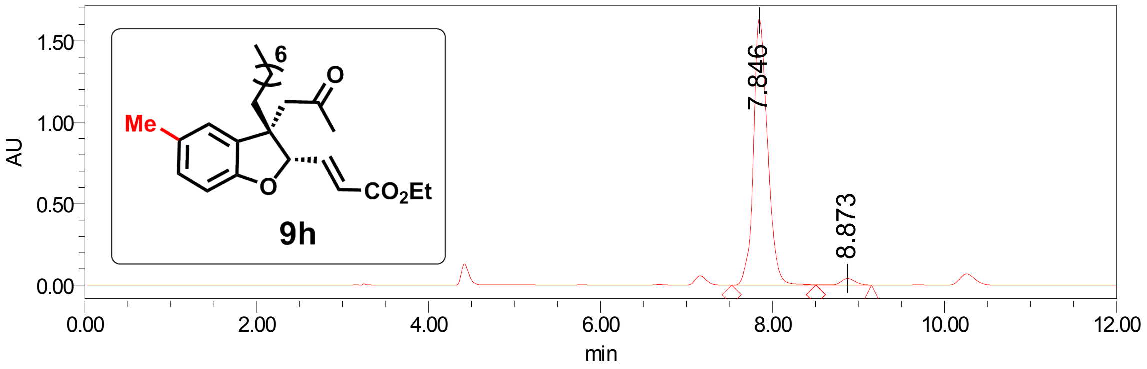

Peak Information:

	RetTime (min)	Area (uV*s)	Area (%)	Height (uV)
1	7.846	18621316.713	97.526	1631626
2	8.873	472340.262	2.474	40305

Supplementary Figure 164. HPLC spectrum of **9h**

Sample Name: 2172-xx-IF-3-95:5 Wave Length: 2018/5/29 12:34:28 CST
Column: 5.00 ul

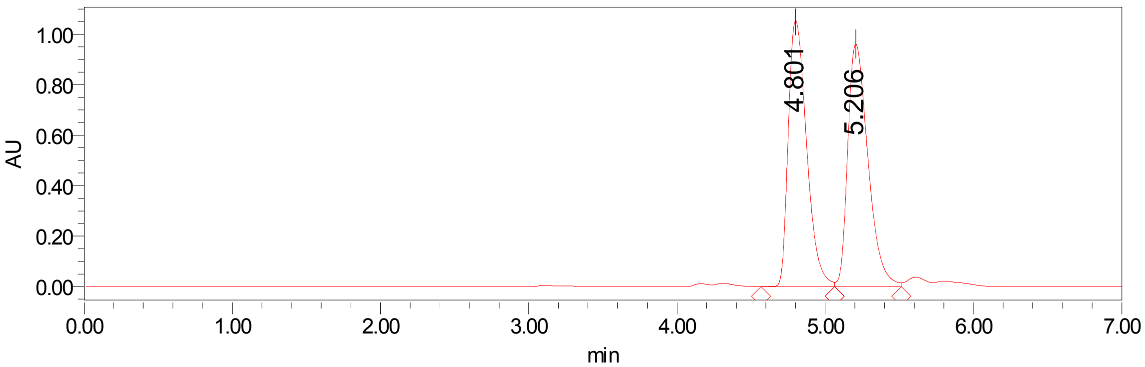

Peak Information:

	RetTime (min)	Area (uV*s)	Area (%)	Height (uV)
1	4.801	9069652.936	49.755	1054254
2	5.206	9158830.732	50.245	961358

Sample Name: 2160-c-IF-3-95:5 Wave Length: 2018/5/21 22:33:28 CST
Column: 5.00 ul

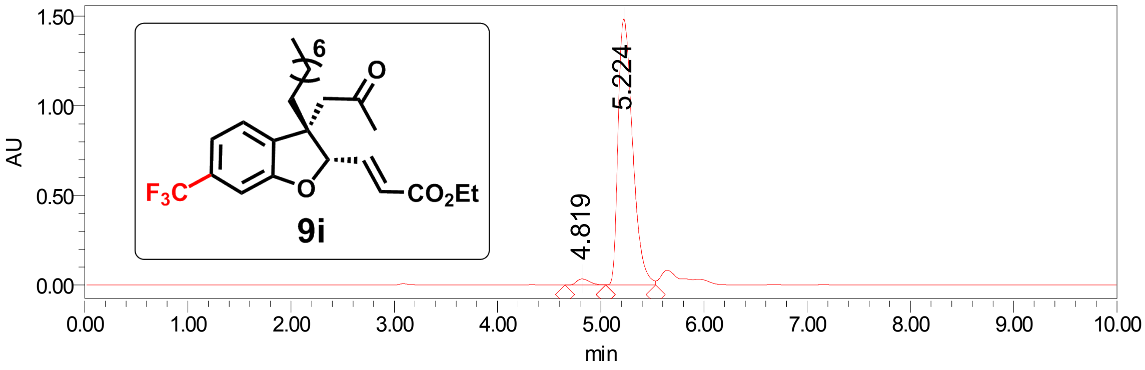

Peak Information:

	RetTime (min)	Area (uV*s)	Area (%)	Height (uV)
1	4.819	310093.921	2.088	34388
2	5.224	14541729.657	97.912	1484411

Supplementary Figure 165. HPLC spectrum of **9i**

Sample Name: 2171-xx-IF-3-95:5 Wave Length: 2018/5/29 12:23:48 CST
Column: 5.00 ul

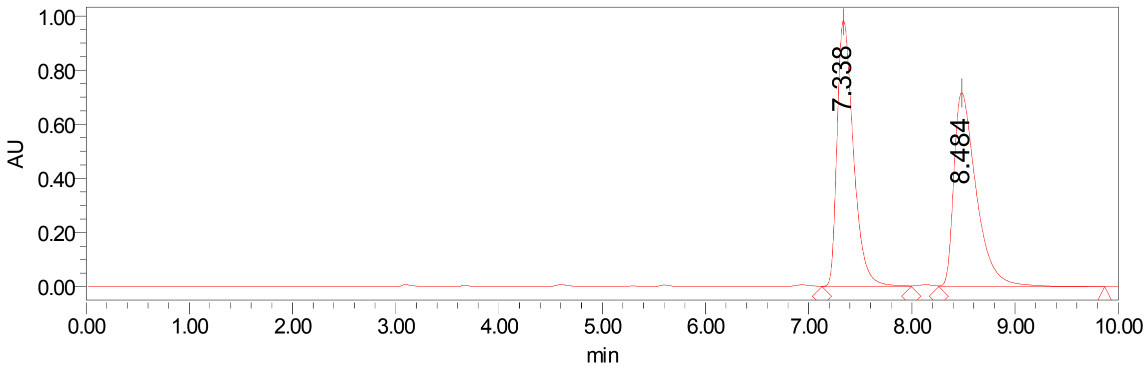

Peak Information:

	RetTime (min)	Area (uV*s)	Area (%)	Height (uV)
1	7.338	10835648.144	50.111	982695
2	8.484	10787442.106	49.889	716039

Sample Name: 2171-c-IF-3-95:5 Wave Length: 2018/5/29 12:05:27 CST
Column: 5.00 ul

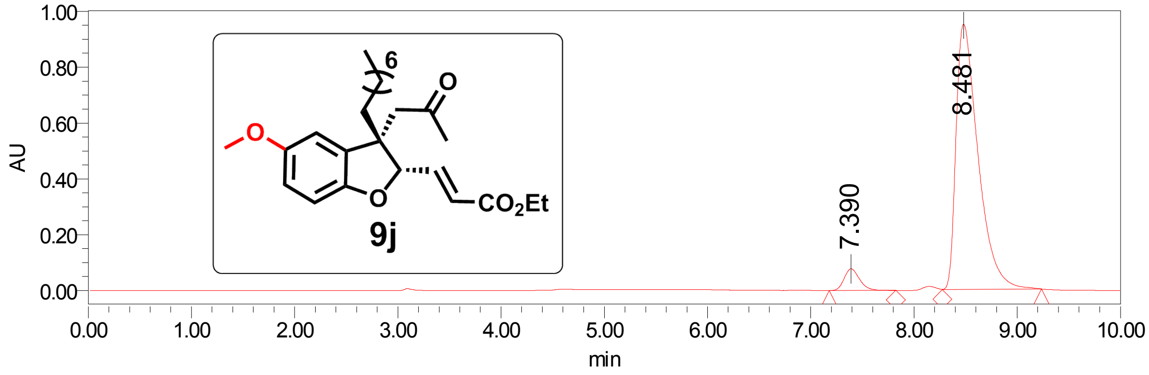

Peak Information:

	RetTime (min)	Area (uV*s)	Area (%)	Height (uV)
1	7.390	794956.344	5.349	76935
2	8.481	14067458.269	94.651	949822

Supplementary Figure 166. HPLC spectrum of **9j**

Sample Name: 2193-IF-3-xx-95:5 Wave Length: 2018/6/17 20:20:26 CST
Column: 5.00 ul

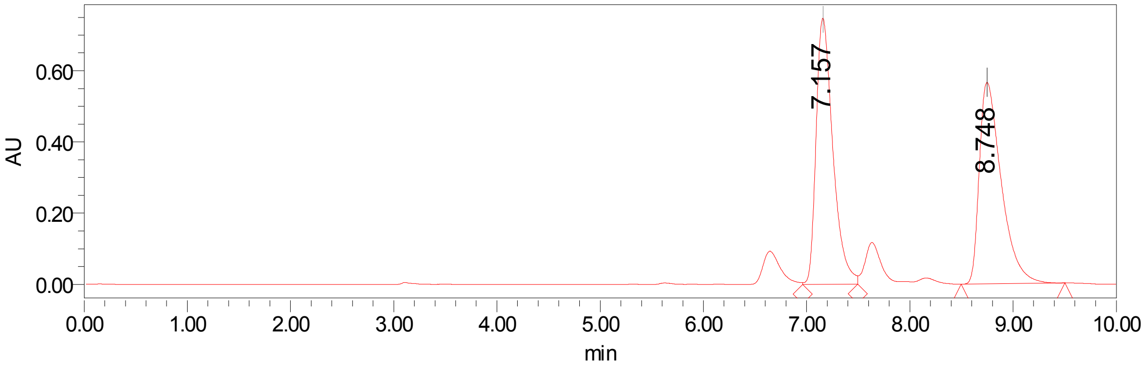

Peak Information:

	RetTime (min)	Area (uV*s)	Area (%)	Height (uV)
1	7.157	8441072.833	50.283	747831
2	8.748	8346089.172	49.717	565929

Sample Name: 2193-IF-3-c-95:5 Wave Length: 2018/6/17 20:09:44 CST
Column: 5.00 ul

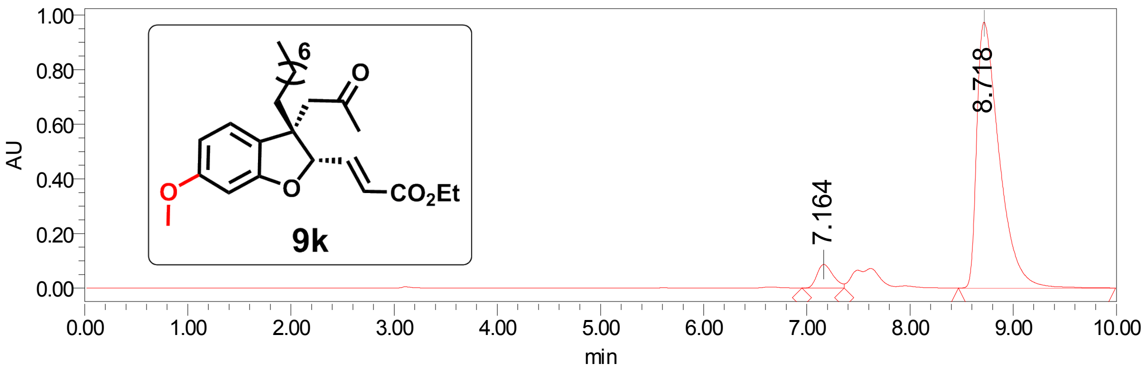

Peak Information:

	RetTime (min)	Area (uV*s)	Area (%)	Height (uV)
1	7.164	938115.321	6.016	86514
2	8.718	14654521.802	93.984	973332

Supplementary Figure 167. HPLC spectrum of **9k**

Sample Name: 2163-xx-IE-3-95:5 Wave Length: 2018/5/24 21:00:49 CST
Column: 5.00 ul

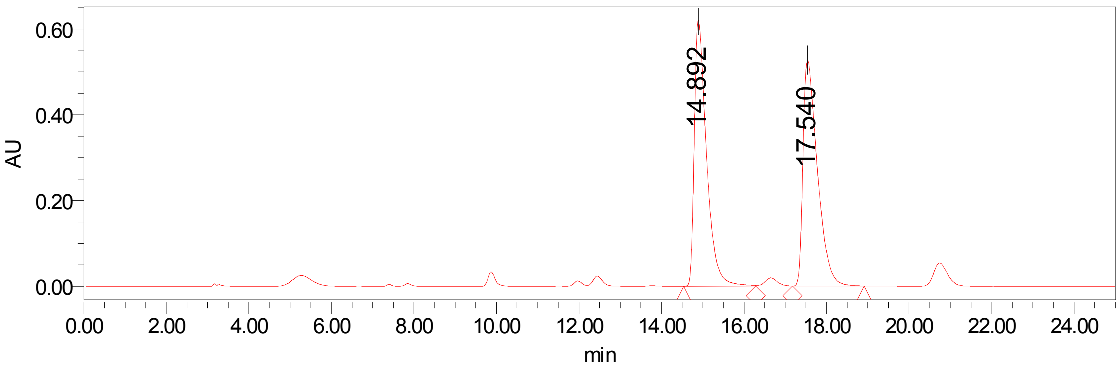

Peak Information:

	RetTime (min)	Area (uV*s)	Area (%)	Height (uV)
1	14.892	12768902.669	50.038	619173
2	17.540	12749504.005	49.962	526272

Sample Name: 2163-c-IE-3-95:5 Wave Length: 2018/5/24 20:35:11 CST
Column: 5.00 ul

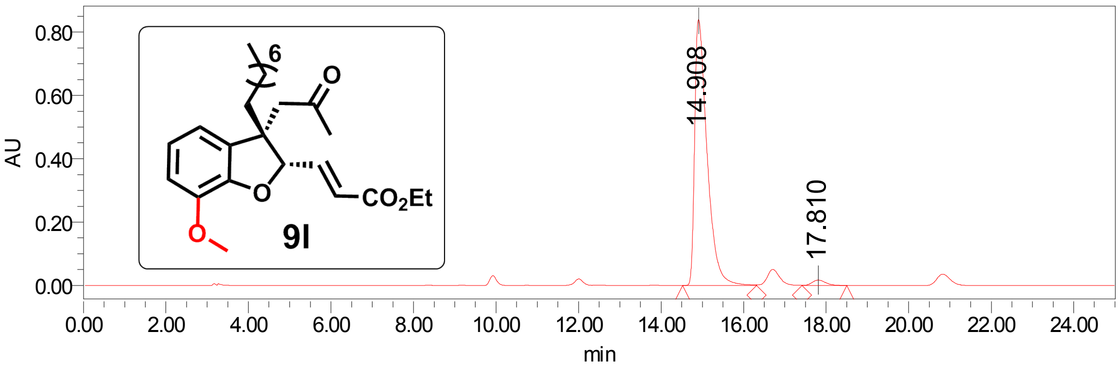

Peak Information:

	RetTime (min)	Area (uV*s)	Area (%)	Height (uV)
1	14.908	17561930.108	97.797	839760
2	17.810	395515.070	2.203	17280

Supplementary Figure 168. HPLC spectrum of **9l**

Supplementary References

1. Murakami, M., Hoshino, Y., Ito, H. & Ito, Y. Palladium-catalyzed coupling reactions of *N*-methoxy-*N*-methylcarbamoyl chloride for the synthesis of *N*-methoxy-*N*-methylamides. *Chem. Lett.* 163-164 (1998).
2. Krishnamoorthy, R., Lam, S. Q., Manley, C. M. & Herr, R. J. Palladium-catalyzed preparation of weinreb amides from boronic acids and *N*-methyl-*N*-methoxycarbamoyl chloride. *J. Org. Chem.* **75**, 1251-1258 (2010).
3. Smith, A. B., Beiger, J. J., Davulcu, A. H. & Cox, J. M. Preparation of [1-(methoxymethylcarbamoyl)ethyl] phosphonic acid bis-(2,2,2-trifluoroethyl) ester: A useful intermediate in the synthesis of Z-unsaturated *N*-methoxy-*N*-methylamides. *Org. Synth.* **82**, 147-156 (2005).
4. Nelson, A. K., Peck, C. L., Rafferty, S. M. & Santos, W. L. Chemo-, regio-, and stereoselective copper(II)-catalyzed boron addition to acetylenic esters and amides in aqueous media. *J. Org. Chem.* **81**, 4269-4279 (2016).
5. Tian, J.-M., *et al.* The design of a spiro-pyrrolidine organocatalyst and its application to catalytic asymmetric Michael addition for the construction of all-carbon quaternary centers. *Chem. Commun.* **51**, 9979-9982 (2015).
6. Tian, J.-M., *et al.* Catalytic asymmetric cascade using spiro-pyrrolidine organocatalyst: efficient construction of hydrophenanthridine derivatives. *Org. Lett.* **19**, 6618-6621 (2017).
7. Hunter, C. A., Jones, P. S., Tiger, P. M. N. & Tomas, S. New building blocks for the assembly of sequence selective molecular zippers. *Chem. Commun.* 1642-1643 (2003).
8. Knorr, R., Rossmann, E. C., Knittl, M. & Boehrer, P. Highly syn selective addition of aqueous HBr to hydrophobically shielded arylalkynes. *Tetrahedron* **70**, 5332-5338 (2014).
9. Mielgo, A. & Palomo, C. α,α-Diarylprolinol ethers: new tools for functionalization of carbonyl compounds. *Chem. Asian. J.* **3**, 922-948 (2008).
10. Jensen, K. L., *et al.* The diarylprolinol silyl ether system: a general organocatalyst. *Acc. Chem. Res.* **45**, 248-264 (2012).
11. Mahlau, M. & List, B. Asymmetric counteranion-directed catalysis: concept, definition, and applications. *Angew. Chem. Int. Ed.* **52**, 518-533 (2013).
12. Xu, B. & Tambar, U. K. Ligand-controlled regiodivergence in the copper-catalyzed [2,3]- and [1,2]-rearrangements of iodonium ylides. *J. Am. Chem. Soc.* **138**, 12073-12076 (2016).
13. Dupuy, C. & Luche, J. L. New developments of the Wharton transposition. *Tetrahedron* **45**, 3437-3444 (1989).
14. Freeman, P. K. & Hutchinson, L. L. Alkyllithium reagents from alkyl halides and lithium radical anions. *J. Org. Chem.* **45**, 1924-1930 (1980).
15. Hill, R. R. & Rychnovsky, S. D. Generation, stability, and utility of lithium 4,4′-di-tert-butylbiphenylide (LiDBB). *J. Org. Chem.* **81**, 10707-10714 (2016).
16. Trost, B. M. & Tang, W. Enantioselective synthesis of (−)-codeine and (−)-morphine. *J. Am. Chem. Soc.* **124**, 14542-14543 (2002).
17. Trost, B. M., Tang, W. P. & Toste, F. D. Divergent enantioselective synthesis of (-)-galanthamine and (-)-morphine. *J. Am. Chem. Soc.* **127**, 14785-14803 (2005).

18. Li, Q. & Zhang, H. Total synthesis of codeine. *Chem. Eur. J.* **21**, 16379-16382 (2015).

19. Varin, M., Barré, E., Iorga, B. & Guillou, C. Diastereoselective total synthesis of (±)-codeine. *Chem. Eur. J.* **14**, 6606-6608 (2008).

20. Omori, A. T*., et al.* Chemoenzymatic total synthesis of (+)-codeine by sequential intramolecular Heck cyclizations via C-B-D ring construction. *Synlett*, 2859-2862 (2007).

21. Leisch, H*., et al.* Chemoenzymatic enantiodivergent total syntheses of (+)- and (−)-codeine. *Tetrahedron* **65**, 9862-9875 (2009).

22. Uchida, K., Yokoshima, S., Kan, T. & Fukuyama, T. Total synthesis of (+/-)-morphine. *Org. Lett.* **8**, 5311-5313 (2006).

23. Chu, S., Munster, N., Balan, T. & Smith, M. D. A Cascade strategy enables a total synthesis of (+/-)-morphine. *Angew. Chem. Int. Ed.* **55**, 14304-14307 (2016).
